# Refining the Global Spatial Limits of Dengue Virus Transmission by Evidence-Based Consensus

**DOI:** 10.1371/journal.pntd.0001760

**Published:** 2012-08-07

**Authors:** Oliver J. Brady, Peter W. Gething, Samir Bhatt, Jane P. Messina, John S. Brownstein, Anne G. Hoen, Catherine L. Moyes, Andrew W. Farlow, Thomas W. Scott, Simon I. Hay

**Affiliations:** 1 Spatial Ecology and Epidemiology Group, Department of Zoology, University of Oxford, Oxford, United Kingdom; 2 Oxitec Ltd., Abingdon, United Kingdom; 3 Department of Pediatrics, Harvard Medical School and Children's Hospital Informatics Program, Boston Children's Hospital, Boston, Massachusetts, United States of America; 4 Department of Community and Family Medicine, Dartmouth College, Hanover, New Hampshire, United States of America; 5 Department of Entomology, University of California Davis, Davis, California, United States of America; 6 Fogarty International Center, National Institutes of Health, Bethesda, Maryland, United States of America; George Washington University, United States of America

## Abstract

**Background:**

Dengue is a growing problem both in its geographical spread and in its intensity, and yet current global distribution remains highly uncertain. Challenges in diagnosis and diagnostic methods as well as highly variable national health systems mean no single data source can reliably estimate the distribution of this disease. As such, there is a lack of agreement on national dengue status among international health organisations. Here we bring together all available information on dengue occurrence using a novel approach to produce an evidence consensus map of the disease range that highlights nations with an uncertain dengue status.

**Methods/Principal Findings:**

A baseline methodology was used to assess a range of evidence for each country. In regions where dengue status was uncertain, additional evidence types were included to either clarify dengue status or confirm that it is unknown at this time. An algorithm was developed that assesses evidence quality and consistency, giving each country an evidence consensus score. Using this approach, we were able to generate a contemporary global map of national-level dengue status that assigns a relative measure of certainty and identifies gaps in the available evidence.

**Conclusion:**

The map produced here provides a list of 128 countries for which there is good evidence of dengue occurrence, including 36 countries that have previously been classified as dengue-free by the World Health Organization and/or the US Centers for Disease Control. It also identifies disease surveillance needs, which we list in full. The disease extents and limits determined here using evidence consensus, marks the beginning of a five-year study to advance the mapping of dengue virus transmission and disease risk. Completion of this first step has allowed us to produce a preliminary estimate of population at risk with an upper bound of 3.97 billion people. This figure will be refined in future work.

## Author Summary

Previous attempts to map the current global distribution of dengue virus transmission have produced variable results, particularly in Africa, reflecting the lack of accuracy in both diagnostic and locational information of reported dengue cases. In this study, instead of excluding these less informed points we included them with appropriate uncertainty alongside other diverse evidence forms. After assembling a comprehensive database of different evidence types, a weighted scoring system calculated “evidence consensus” for each country a continuous measure of the certainty of dengue presence or absence when considering the full aggregate of evidence. The resulting map and analysis helped highlight important evidence gaps that underlie uncertainties in the current distribution of dengue. We also show the importance of local knowledge through incorporating questionnairebased responses that can help add clarity in uncertain regions. This analysis showed that presence/absence maps do not sufficiently highlight the uncertainties in the evidence base used to construct them. Mapping by evidence consensus not only encourages greater data inclusion, but it also better illustrates the current global distribution of dengue. Consensus mapping is thus ideal for a range of neglected tropical diseases where the evidence base is incomplete or less diagnostically reliable.

## Introduction

Despite increased interest in dengue in recent years, the global distribution of dengue remains highly uncertain. Estimates for the population at risk range from 30% [1] to 54.7% [2] of the world's population (2.05–3.74 billion) while the Centers for Disease Control (CDC) and the World Health Organization (WHO) currently disagree on dengue presence in 34 countries across five continents (Table S1). Clinical features of dengue virus infection include fever, rash and joint pain [3], which ensure the disease's misdiagnosis and mis-reporting among many other febrile illnesses. The diagnostic methods available also have limitations and a full complement of tests is not feasible in many healthcare settings. There is consensus, however, that dengue is a growing problem both geographically and in its intensity [4], [5], [6].

There is an urgent need to compile more extensive occurrence records of dengue virus transmission and assess them for contemporariness and accuracy. Evidence on dengue transmission comes in a wide variety of forms, with varying levels of spatial coverage and reliability. A global audit of dengue distribution therefore requires a transparent methodology to compile these disparate data types and synthesise an output map summarising the current consensus for each country. Such a methodology for compiling and assessing evidence must be robust, repeatable, able to evaluate a large variety of evidence types and incorporate expert opinion. An ideal output metric is a summary statistic (hereafter referred to as evidence consensus) that quantifies certainty on dengue virus transmission presence or absence given the accuracy and contemporariness of the evidence available. An evidence-based map of the current distribution of dengue virus transmission will have direct implications for design and implementation of dengue surveillance and, by showing gaps in contemporary knowledge, provide an advocacy platform for improved data.

Existing approaches to mapping the global limits of vector-borne diseases have used estimates of biological suitability of local environments, which have proved informative in the cases of some pathogens, such as *Plasmodium falciparum* [7], [8] and *P. vivax* [9]. Several approaches have been used to map biological suitability for dengue using non-dengue-specific variables such as temperature, rainfall and satellite-derived environmental variables [1], [10], [11]. Although successive attempts have each increased predictive capacity and resolution, this approach produces variable results in Africa due to a scarcity of confirmed occurrence points across extensive geographic areas. An alternative approach has been to map evidence of dengue occurrence making no assumptions about biological suitability, as in Van Kleef *et al.*, who reviewed published literature to contrast historic, current and future limits of dengue [5]. To date dengue mapping has focussed on future scenarios, yet understanding of the current distribution of dengue virus transmission is far from complete and needs to be better evaluated before we can make predictions about forthcoming patterns and trends. In this study we combine evidence from large occurrence-point style databases used in biological suitability mapping approaches with a wider systematic review of various sources of evidence to create a more comprehensive dengue database. Using this database we then use the novel method of defining evidence consensus to evaluate the current level of certainty on dengue virus transmission presence or absence at national (and some sub-national) levels using a weighted evidence scoring system. Finally, we present these results as a series of global maps that explicitly identify surveillance gaps.

This study is the initial part of a five year project to collect, analyse and publicise global dengue virus transmission data. While the map presented here is the most extensive display of current dengue evidence available, we hope that continual data acquisition will result in more evidence from uncertain areas, increasing the resolution at which we can map evidence consensus in future advances.

## Methods

### Collection of dengue virus transmission evidence

**Figure 1:**
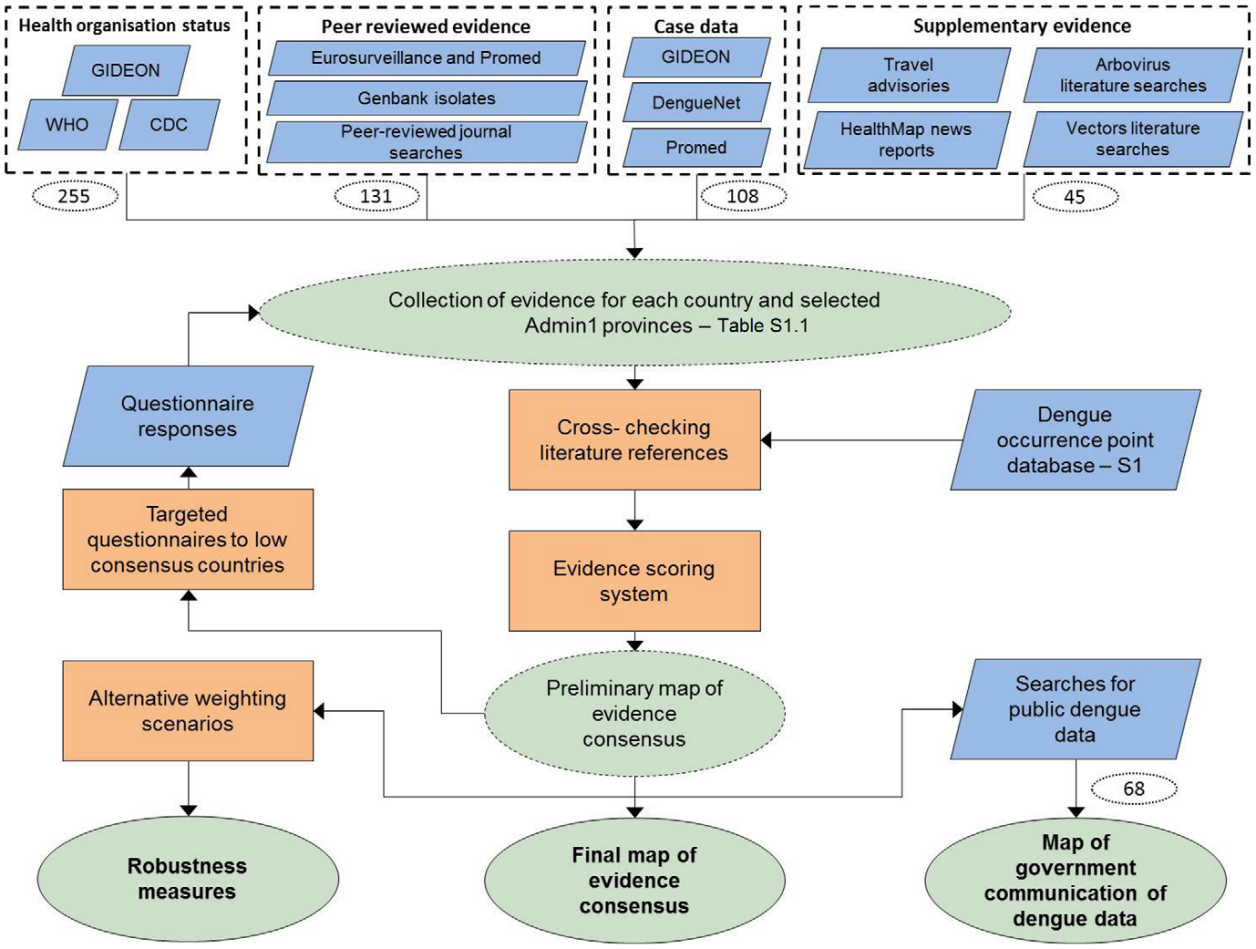
Schematic overview of the methods. Blue diamonds describe input data; orange boxes denote experimental procedures; green ovals indicate output data; dashed lines represent intermediate outputs and solid lines final outputs; dotted white ovals denote the number of countries for which data was available and added to the final output. Dotted rectangles identify the different evidence categories and their main data sources. S1 = Protocol S1.

Evidence for indigenous dengue virus transmission was obtained from four evidence categories: health organisations, peer-reviewed evidence, case data and supplementary evidence (Figure 1). The first three categories were used for all countries. For countries where some of these categories were not available and/or did not provide good consensus, the fourth category of supplementary evidence was used. Evidence was initially collected at a country level (Admin0), but resolution was improved to a state/province level (Admin1) or district level (Admin2) at the fringes of the distribution of detectable virus transmission when sufficient data were available.

Country dengue status as defined by health organisations was determined by consulting the WHO [12] and CDC [13] dengue distribution maps as well as the Global Infectious Diseases and Epidemiology Online Network (GIDEON) database [14]. GIDEON provides a collection of literature and case reports for a range of tropical and infectious diseases in 224 countries. Dengue status by country was recorded as present or absent.

The peer-reviewed evidence category contained evidence of dengue occurrence as determined by peer-reviewed sources where details of diagnostic techniques were given. Peer-reviewed journal (Google Scholar, PubMed, ISI Web of Science) and disease surveillance network (ProMED archives, Eurosurveillance archives) searches were conducted with search terms “country” or “Admin1/2” and “dengue”. Sources were included for the period 1960–2012 and only if cases were confirmed as resulting from indigenous (i.e. not imported) transmission. The specialist regional journal collections African Journals Online (http://www.ajol.info/) and China National Knowledge Infrastructure (http://en.cnki.com.cn/) were also searched. Extra publications were found by searching using the location term in Genbank nucleotide records for dengue viruses isolated from human hosts. The search of peer-reviewed sources of evidence resulted in a total of 285 articles being selected for 123 countries where positive dengue occurrence records were identified. This included evidence from returning travellers who were diagnosed upon return to their often non-endemic home countries as opposed to the transmission setting. For these cases, evidence was attributed to the place to which they had travelled. The added value of returning traveller reports is that the travellers are often more immunologically naïve to dengue infections, and also that diagnosis is often pursued more rigorously. Therefore, the sensitivity of detecting an infection is increased. The results of our search were then cross-referenced against a dengue occurrence-point database compiled internally, in a separate exercise. Unlike our country-specific searches, this database of 2836 articles results from searches simply for “dengue”, which were then geo-referenced using the article text. Full details are available in Protocol S1 and the geographic location of the occurrence points are displayed in Figures S1, S2, S3, S4, S5, S6. This cross-referencing resulted in the inclusion of an additional 16 articles in the current analysis and also provided increased justification for our choice of countries to evaluate at Admin1 level.

The case data category contained evidence of dengue outbreaks (minimum 50 infections) where evidence contained less diagnostic detail, but was more informative about the magnitude of dengue transmission occurring. Case data from the most recent outbreak were obtained from the Program for Monitoring Emerging Diseases (ProMED) archive search, WHO DengueNet data query [15] and from GIDEON which holds a detailed record of government-reported case numbers. This resulted in 100 countries with useful dengue case data.

In many resource-poor countries, both surveillance and researcher-generated reports are rare. Therefore, in countries where other evidence categories were sparse, we looked for supplemental evidence that suggested possible dengue virus presence. Supplemental evidence types included: presence of an established mosquito vector population of public health significance (*Aedes aegypti*, *Ae. albopictus* or *Ae. polynesiensis*) as documented by peer-reviewed literature, confirmed presence of multiple other rarely diagnosed arboviral diseases as documented by peer-reviewed literature, news reports of dengue epidemics found using GoogleNews archives (http://news.google.co.uk/archivesearch) and travel advisories from the National Travel Health Network and Centre (http://www.nathnac.org/ds/map_world.aspx) issued at a country-level. We included evidence of multiple other rarely diagnosed arboviral diseases, as these are informative about the ability of a country to detect any possible dengue infection. If other arboviral diseases are poorly reported, but documented by peer-reviewed literature as present, then it is possible that dengue is also underreported. In addition to this, we cross-referenced our dataset with the HealthMap database (www.healthmap.org/dengue/). This website-based application automatically geo-positions cases from websites with news reports and outbreak alerts related to dengue and contains data from a wide variety of sources dating back to 2007 [16], [17]. This extensive database contributed important evidence especially at smaller spatial scales and in areas where translated articles are not so easily obtained. Supplementary evidence was used in evaluating dengue consensus in 45 countries.

While the categories are clearly defined here and in Figure 1, some overlap of evidence sources did occur, depending on the information content of each source. This meant evidence sources such as ProMED reports could be included twice, in both the peer-reviewed evidence and case data categories, if they contained information about diagnostic tests used for confirmation as well as overall outbreak case numbers. In this section we outline the main sources used for each category, but it should be noted that if evidence from a particular source fitted the criteria for a different evidence category, it was not excluded, but rather included in that category.

### Quantifying evidence with a weighted scoring system

**Figure 2:**
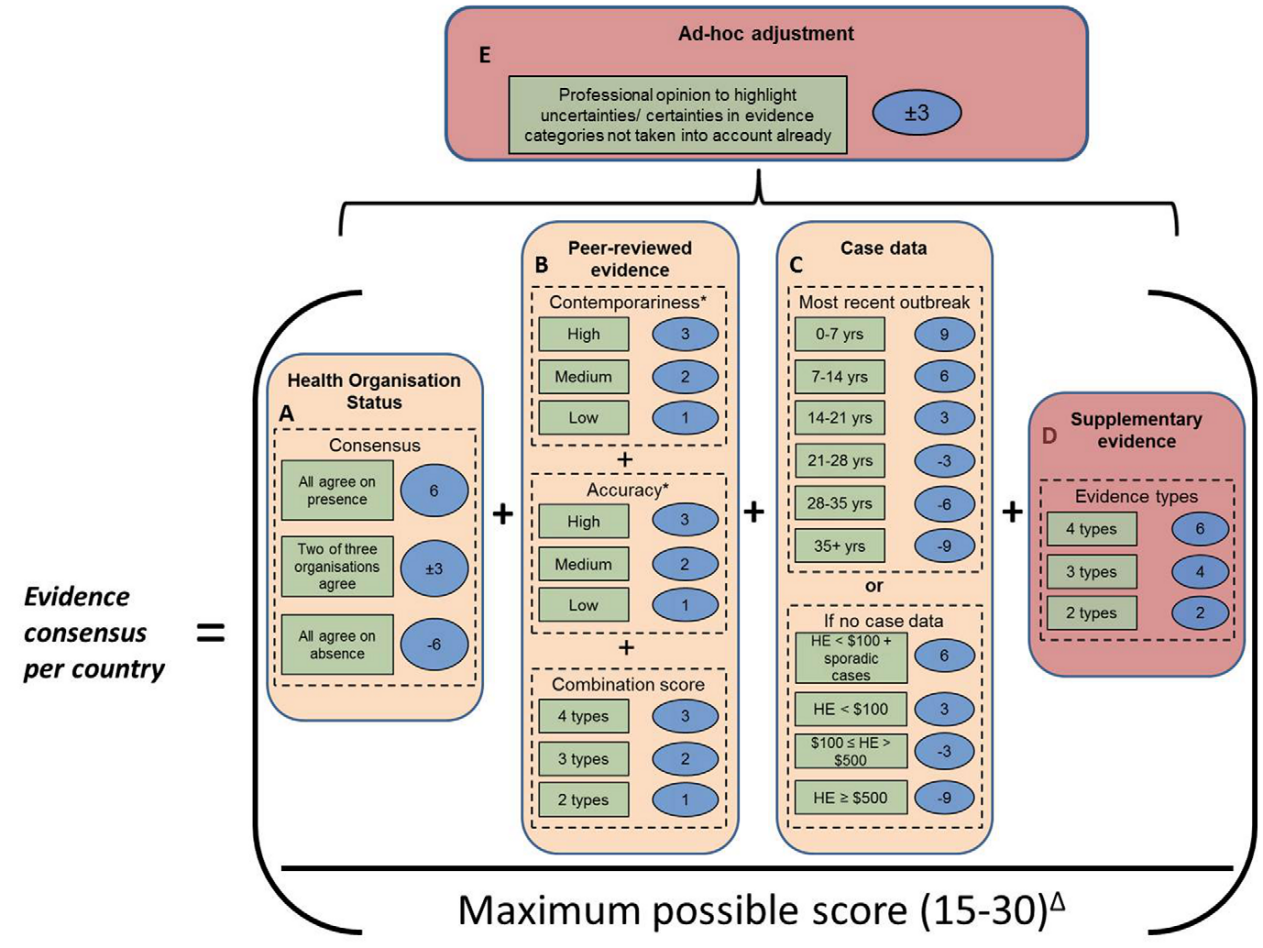
Overview of the evidence scoring system. Cream boxes represent mandatory categories while red boxes represent optional categories that are only used where required (see Methods). Dashed lines surround individual parameters that are assessed and totalled in the scoring system. Green boxes describe the level of evidence, with a given score in the blue oval. * Each individual piece of literary evidence is scored for contemporariness and accuracy before taking an average of the whole set then adding the combination score. Evidence consensus is calculated as the proportion of the maximum possible score from the dashed lined characteristics that are used. Δ Maximum possible score depends on which categories are included and can vary from 15 (Case data and Health organisation status, but no peer-reviewed evidence available) to 30 (all evidence categories included). Yrs = years. HE = total healthcare expenditure per capita at average U.S. $ exchange rates.

In order to quantify evidence consensus, a weighted scoring system was developed that attributed positive values to evidence of presence and negative values to evidence of a lack of presence. The aim here was to use an optimal subset of evidence to accurately assess dengue status within a given area. By scoring the evidence categories mentioned above individually and then combining their respective scores, we were able to calculate “evidence consensus,” a measure of how strongly the combined evidence collection supports a dengue-present or dengue-absent status (Figure 2). We defined a country as having “complete consensus” on dengue presence when the evidence base was comprised of contemporary forms of most or all of the following evidence types: 1) unanimous health organisations agreement, 2) a seroprevalence survey, 3) Polymerase Chain Reaction (PCR) typing of dengue virus or dengue viral RNA, 4) a foreign visitor to the area with a confirmed dengue infection upon returning to their home country, and 5) records of an epidemic of greater than 50 infections. Such a country has a consensus score of between 80% and 100%. A country with a complete consensus on dengue virus absence is characterised by all health organisations agreeing on dengue absence and high healthcare expenditure (as an approximate proxy for surveillance capability), therefore accounting for both the observed absence of dengue and the minimised possibility of any undetected dengue infections. Such a country scores between −80% and −100% on our scale. A country with no consensus on dengue virus status is characterised by conflicting evidence from different categories and scores close to 0%. Each evidence category was scored independently and category weights applied to reflect the level of detail each category provides: health organisation status (maximum score 6), peer-reviewed evidence (maximum 9), case data (maximum 9) and supplementary evidence (maximum 6). To support the choice of assigned category weights we performed a sensitivity analysis in which two alternative evidence weighting scenarios were applied to the same sources of data: 1) neutral (all categories hold the same weight) and 2) reversed (health organisation status and supplementary evidence hold weight 9, peer-reviewed evidence and case data hold weight 6). We then checked for any major deviations in overall country score resulting from such alternative scenarios.

#### Health organisation evidence

The data from the three health organisations (WHO, CDC and GIDEON) comprised discrete presence or absence answers. A consensus (+++ or −−−) scored 6 or −6 respectively, while a lack of consensus (++− or −−+) scored 3 or −3 respectively (Figure 2A). This gave a maximum score for this category of ±6.

#### Peer-reviewed evidence and returning traveller reports

These forms of evidence were each scored independently for contemporariness and accuracy. The date of occurrence was used for scoring as follows: between 2012–2005 = 3, 2004–1997 = 2 and pre-1997 = 1 (Figure 2B). This corresponded to a conservative estimate of the inter-epidemic period for dengue of three to five years [18]. This score was then added to a score for accuracy, whereby high accuracy, and a score of 3, was characterised by PCR methods, a Plaque Reduction Neutralization Test (PRNT), or a detailed case description of a complication of the disease. Complications of the disease were either dengue haemorrhagic fever (DHF) grades 1 and 2 or dengue shock syndrome (DSS) grades 3 and 4 under the old classification scheme [19] or severe dengue under the new classification scheme [3]. Medium accuracy methods including IgM- and IgG- based ELISA and Hemagglutination Inhibition (HI) assay approaches scored 2 because their calibration is sensitive to background immune responses [20], antibody response is variable over the course of an infection [21] and the test can cross-react with other non-dengue arboviruses [20]. A low accuracy score of 1 was used for articles that only reported case numbers with a non-dengue-specific case definition or a low participant number. Each included article was scored separately and then an average score was taken from all articles. This presented the possibility of devaluing the score of the most accurate and contemporary piece of evidence, so an extra score was added to reflect increased certainty provided by multiple forms of evidence. Evidence types 2) through 5) described above contributed to this extra score as such: if two types of evidence were present a score of 1 was added, three types = 2, four types = 3. This resulted in a maximum available score of 9 for peer-reviewed evidence.

#### Case data

This category was scored by contemporariness in eight-year intervals. The most frequent year in which an outbreak (over 50 cases or over 15 cases if the population is below 100,000) occurred was again scored in average inter-epidemic period intervals: 0–7 years since the last outbreak scored 9, 7–14 years = 6, 14–21 = 3, 21–28 = −3, 28–35 = −6, 35+ = −9 (Figure 2C). Where case data were unavailable, the distinction between true absences and inadequate surveillance was made using total annual healthcare expenditure (HE) per capita at average U.S. Dollar exchange rates (2011 WHO health statistics) [22]. Higher HE has been linked to better overall public health infrastructure, which includes high-quality diagnostic resources, greater healthcare coverage and higher levels of expertise, all of which may result in a more thorough characterisation of dengue status at the country-level [23], [24], [25]. Therefore, the lower the HE, the less certain we can be that an absence of case data accurately reflects an absence of dengue transmission. Class intervals for HE were chosen to reflect regional differences both within and between continents. Where information on HE was unavailable (Somalia, North Korea and Zimbabwe), low HE status was assigned. All overseas territories were assumed to have the same HE as their parent nations. The following criteria were used to derive the case scores in the absence of dengue case data: HE<$100 and reports of sporadic unconfirmed cases gave a score of 6, HE<$100 = low HE = 3, $100≤HE<$500 = medium HE = −3, HE≥$500 = high HE = −9 (Figure 2C). The maximum score for the case data category was ±9.

#### Supplementary evidence

This formed part of the evidence base if there was some suggestion of dengue presence, but the above three categories were insufficient to provide certainty on dengue status. If only two evidence types were available (see above), a score of 2 was given, three types = 4, four types = 6 (Figure 2D). Supplementary evidence carried a maximum score of 6.

Where a national score showed some uncertainty and an additional factor existed that was not captured by the default scoring system, an adjustment of up to ±3 was applied. For example, if multiple evidence categories suggested dengue presence in a country with high HE, but there was no case data, then the case data score was adjusted so as not to hold a disproportionate weight in deciding overall dengue status. This is termed the “ad hoc adjustment” (Figure 2E).

To derive an overall country evidence consensus score, the scores for all evidence categories were summed, and then divided by the maximum possible score and multiplied by 100. Evidence consensus was then mapped according to nine equal interval categories from 100% to −100% that differentiated evidence consensus worldwide, with evidence consensus being defined as complete (±79% to ±100%), good (±57% to ±78%), moderate (±34% to ±56%), poor (±12% to 33%) or indeterminate (−11% to 11%). An odd number of intervals was chosen so as to highlight places where consensus is very low (indeterminate) and where improved surveillance is particularly needed. As such, the resulting classification of consensus scores should not be strictly interpreted, but rather taken as a general indication of the quality of dengue evidence in a given location. A full breakdown of the exact evidence included, individual scores and overall consensus percentages are given for each country in Table S1 and Figure S7.

### Refining the evidence base and map with questionnaires targeted to consensus poor countries

In countries where evidence consensus was at best moderate, we attempted to increase consensus through targeted questionnaires. The questionnaire asked about endogenous surveillance and data collection. If available, diagnostic method(s) and summary results were requested. Any returned data or reports were then entered into their relevant evidence categories and scored in combination with existing evidence. Questionnaires were distributed to healthcare officials in the country of interest as well as selected offices of the Institut Pasteur. Questionnaire responses and expert comments are part of an on-going process that will lead to future modifications of this map.

### Identification of countries that publically distribute dengue case data

To map public awareness of dengue worldwide, we searched the ministry of health websites of each of the 128 countries identified as dengue-present (evidence consensus positive but not indeterminate). A country was indicated as publicly displaying dengue data if national dengue case numbers were displayed annually or during epidemic years at a minimum.

### Population at risk calculations

To calculate the maximum possible population at risk for dengue virus transmission we obtained total population counts from the Global Rural Urban Mapping Project (GRUMP) for the 128 countries identified as dengue-present. The GRUMP *beta* version provides gridded population count estimates at a 1×1 km spatial resolution for the year 2000 [26], [27]. Population counts for the year 2000 were projected to 2010 by applying country-specific urban and rural national growth rates [28] using methods described previously [29]. As 2010 forms a landmark year for many national censuses, we were able to adjust these expanded population counts using the United Nations 2010 population estimates [30].

## Results

### Global distribution of dengue virus transmission based on evidence consensus

**Figure 3:**
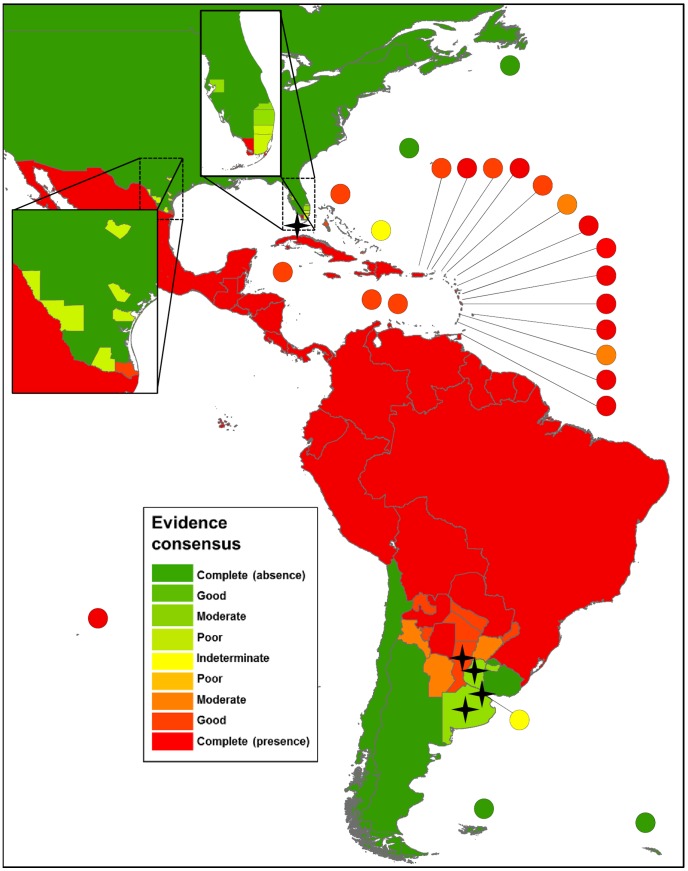
Evidence consensus on dengue virus presence and absence in the Americas. Figure 3 shows the areas categorised as complete evidence consensus on dengue absence in dark green, through to areas with indeterminate evidence consensus on dengue status in yellow, then up to areas with complete evidence consensus on dengue presence in dark red. Stars indicate one off indigenous transmission events with fewer than 50 cases. The map displays evidence consensus at Admin1 (state) level for Argentina and Uruguay, Admin2 (county) level for the United States of America and Admin0 (country) level for all other countries.

**Figure 4:**
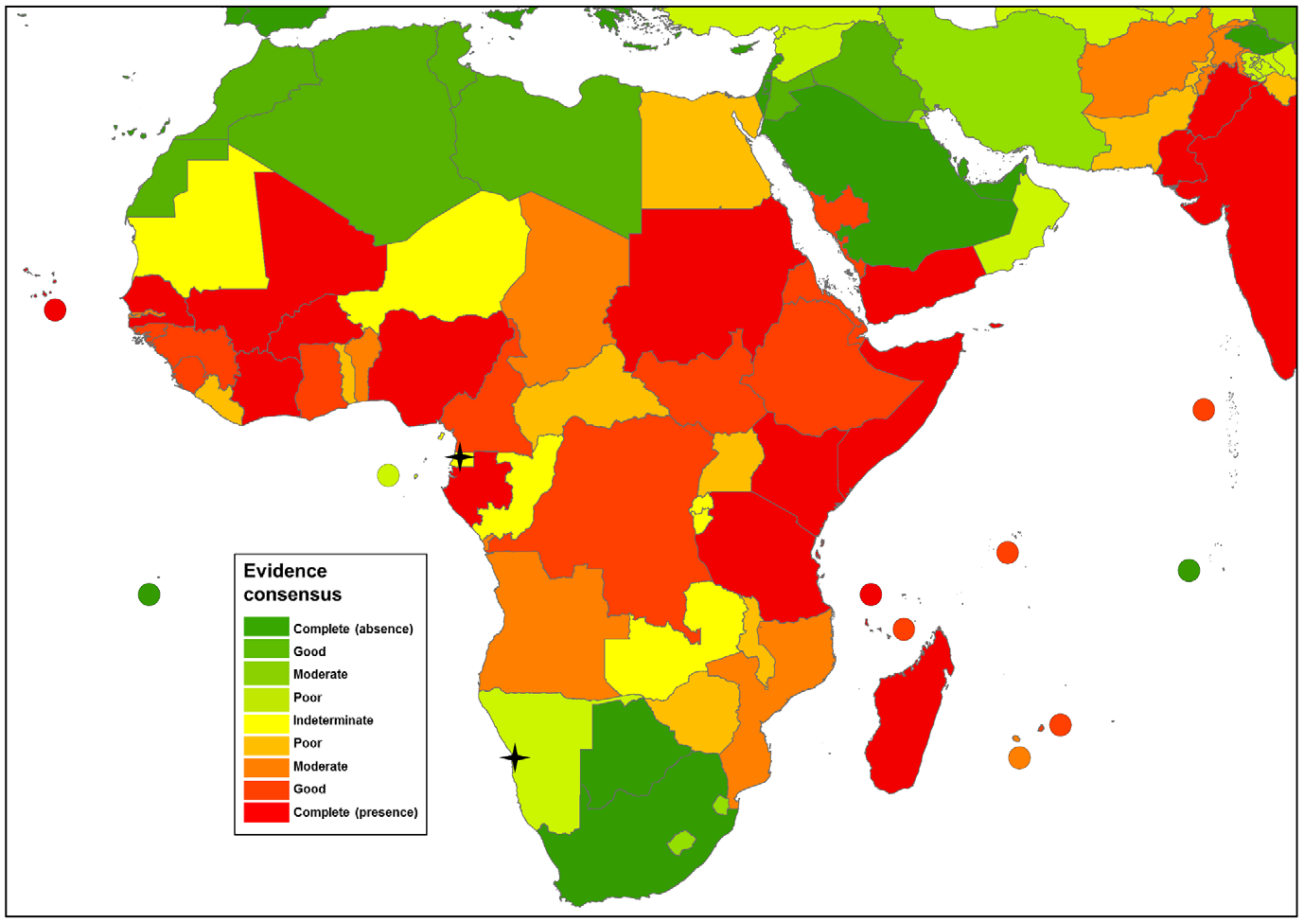
Evidence consensus on dengue virus presence and absence in Africa. Figure 4 shows the areas categorised as complete evidence consensus on dengue absence in dark green, through to areas with indeterminate evidence consensus on dengue status in yellow, then up to areas with complete evidence consensus on dengue presence in dark red. Stars indicate one off indigenous transmission events with fewer than 50 cases. The map displays evidence consensus at Admin1 (state) level for Saudi Arabia and Pakistan and Admin0 (country) level for all other countries.

**Figure 5:**
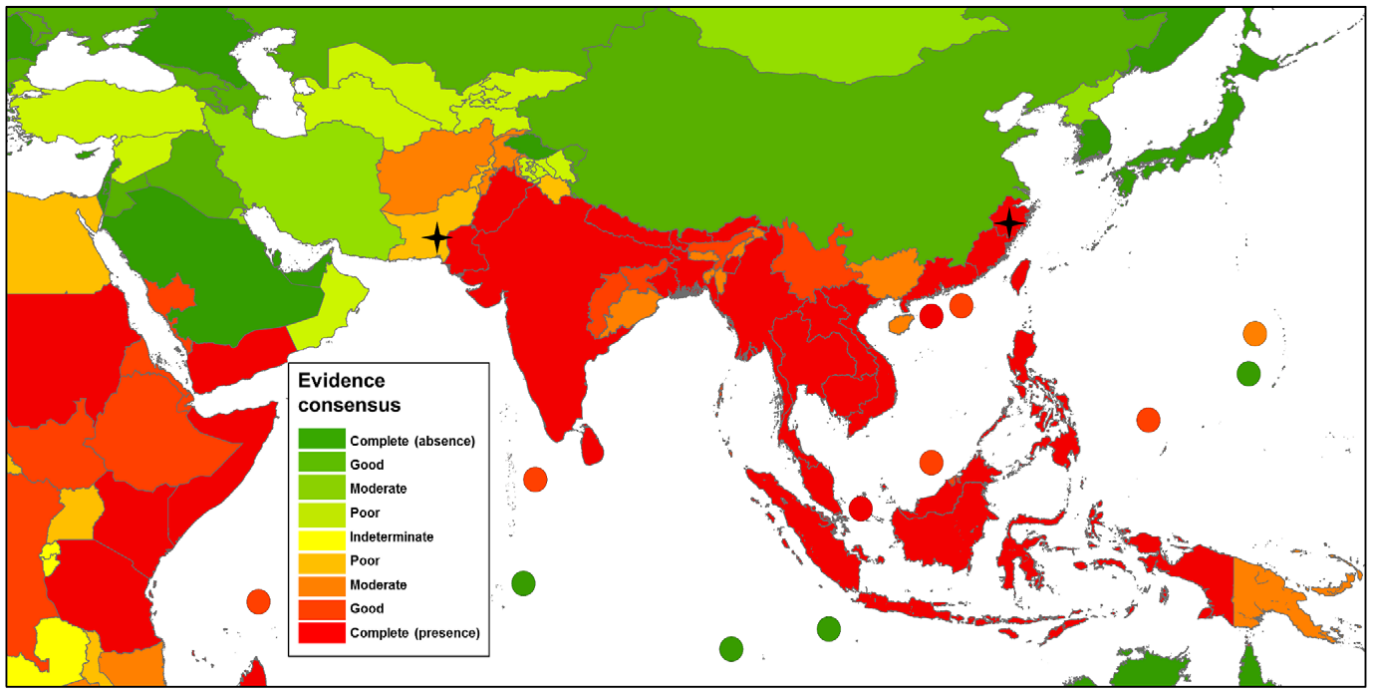
Evidence consensus on dengue virus presence and absence in Asia. Figure 5 shows the areas categorised as complete evidence consensus on dengue absence in dark green, through to areas with indeterminate evidence consensus on dengue status in yellow, then up to areas with complete evidence consensus on dengue presence in dark red. Stars indicate one off indigenous transmission events with fewer than 50 cases. The map displays evidence consensus at Admin1 (state) level for Saudi Arabia, Pakistan, India, China and South Korea and Admin0 (country) level for all other countries.

**Figure 6:**
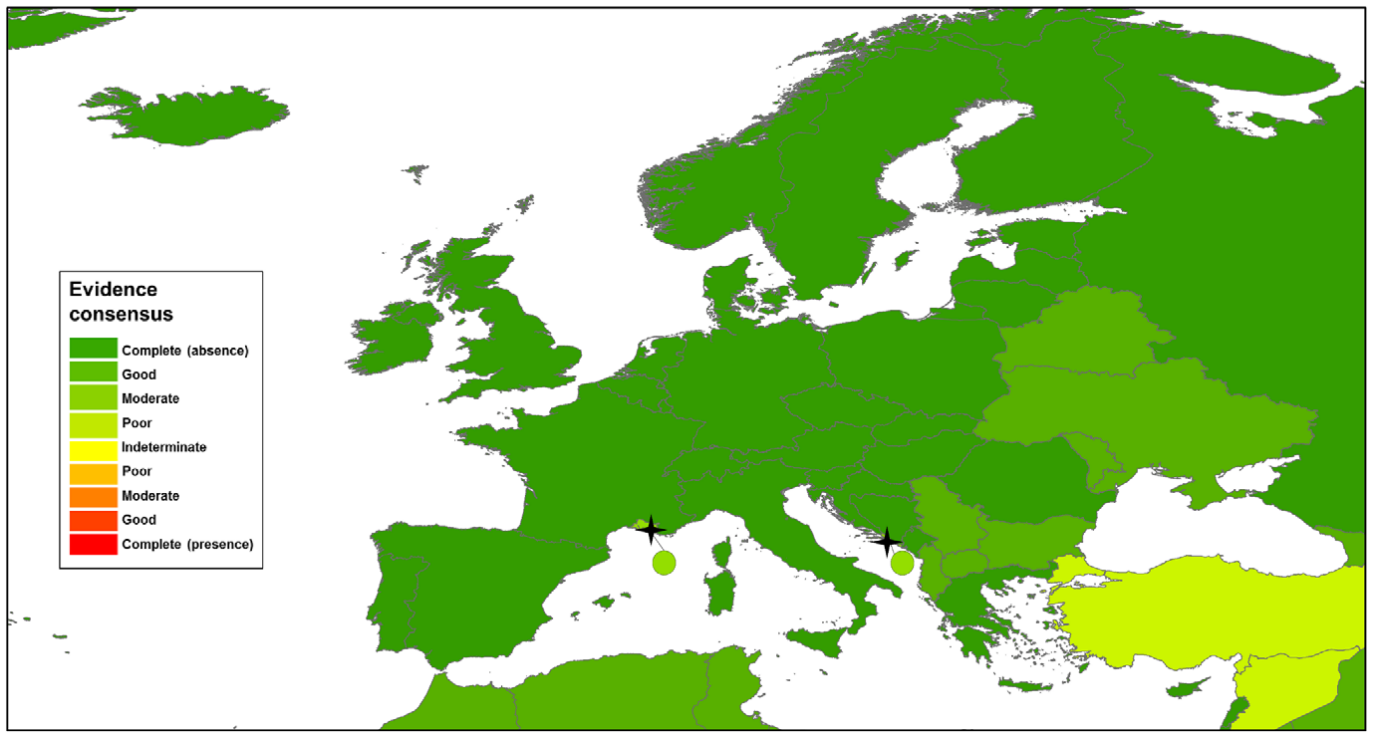
Evidence consensus on dengue virus presence and absence in Europe. Figure 6 shows the areas categorised as complete evidence consensus on dengue absence in dark green, through to areas with indeterminate evidence consensus on dengue status in yellow. Stars indicate one off indigenous transmission events with fewer than 50 cases. The map displays evidence consensus at Admin2 (county) level for France and Croatia and Admin0 (country) level for all other countries.

**Figure 7:**
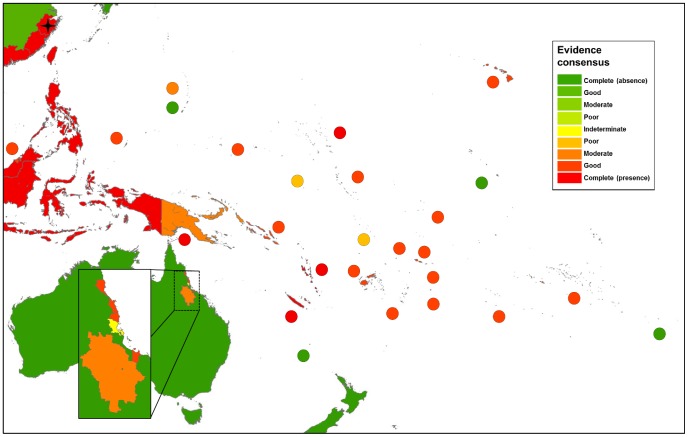
Evidence consensus on dengue virus presence and absence in Australasia. Figure 7 shows the areas categorised as complete evidence consensus on dengue absence in dark green, through to areas with indeterminate evidence consensus on dengue status in yellow, then up to areas with complete evidence consensus on dengue presence in dark red. Stars indicate one off indigenous transmission events with fewer than 50 cases. The map displays evidence consensus at Admin1 (state) level China, Admin2 (county) level for Australia and Admin0 (country) level for all other countries.

The global distribution of dengue virus transmission as defined by evidence consensus is shown in Figures 3–7. The mapped colour scale ranges from complete consensus on dengue presence (dark red) to indeterminate consensus on dengue status (yellow) then through to complete consensus on dengue absence (dark green). A full list of the evidence used for each area and their scoring is available in Table S1 and Figure S7. In total we identified 128 countries as dengue-present (i.e. positive values outside the indeterminate range), compared to 100 from the WHO, 104 from the CDC and 118 from GIDEON. Compared to the lists produced by the WHO and CDC, we identified 41 additional countries where evidence consensus for presence was outside the indeterminate range yet dengue-absent status was assigned by at least one of these health organisations.

Even after performing the sensitivity analysis described earlier, the number of countries defined by our methodology as dengue-present but defined by WHO/CDC as absent never dropped below 36 (Table 1). We therefore suggest that this list of 36 countries be subject to a review regarding their current health organisation dengue-absent classification. Of these countries, 31 had at least moderate consensus on dengue presence in our final analysis.

**Table 1:** Countries that require a reassessment of dengue status by health organisations.

Country	Evidence consensus (%)	Health organisations with dengue-absent status	Evidence included
**American Samoa**	Good (76)	CDC	2007 outbreak and SE
**Aruba**	Good (67)	WHO	2005 outbreak and PCR virus typing
**Bahamas**	Good (67)	WHO	2011 outbreak
**Benin**	Moderate (40)	WHO, CDC	Returning traveller reports, PCR virus typing and SE
**Brunei**	Good (75)	WHO	2010 outbreak, PCR virus typing
**Cameroon**	Good (76)	WHO	Seroprevalence surveys, returning traveller reports and questionnaire responzse
**Cayman Islands**	Good (69)	WHO	2010 outbreak and SE
**Chad**	Moderate (40)	WHO, CDC	Returning traveller reports and SE
**Comoros**	Complete (81)	WHO	2010 outbreak, seroprevalence survey and returning traveller reports
**Cook Islands**	Good (60)	WHO, CDC	2009 outbreak, PCR virus typing and SE
**Djibouti**	Good (75)	WHO	2005 outbreak, returning traveller reports and PCR virus typing
**Eritrea**	Good (63)	WHO	Returning traveller reports
**Fiji**	Good (69)	CDC	2012 outbreak and description of DHF
**French Polynesia**	Good (75)	CDC	2009 outbreak, PCR virus typing and description of DHF
**Guinea-Bissau**	Good (60)	WHO, CDC	Returning traveller reports, questionnaire response and SE
**Kiribati**	Good (71)	CDC	2008 outbreak and PCR virus typing
**Liberia**	Poor (29)	WHO, CDC	Reports of sporadic outbreaks and SE
**Maldives**	Good (71)	WHO	2011 outbreak and seroprevalence survey
**Marshall Islands**	Complete (80)	CDC	2011 outbreak
**Mauritius**	Good (65)	WHO	2009 outbreak, seroprevalence survey and PCR virus typing
**Mayotte**	Good (75)	WHO	2005 outbreak, seroprevalence survey and PCR virus typing
**Micronesia**	Good (69)	WHO, CDC	2011 outbreak, returning traveller reports, PCR virus typing and description of DHF
**Netherlands Antilles**	Good (75)	WHO	2008 outbreak and seroprevalence survey
**Nauru**	Poor (20)	CDC	PCR virus typing and SE
**Niue**	Good (65)	CDC	On-going-low level indigenous transmission with reports of sporadic outbreaks and PCR virus typing
**Northern Mariana Islands**	Moderate (54)	CDC	2001 outbreak and seroprevalence survey
**Reunion**	Moderate (43)	WHO, CDC	2010 outbreak, PCR virus typing and SE
**Samoa**	Good (68)	CDC	2001 outbreak, Returning traveller reports, PCR virus typing
**Seychelles**	Good (63)	WHO	2004 outbreak
**South Sudan**	Good (67)	WHO	PCR virus typing
**Togo**	Poor (30)	CDC	Returning traveller reports and SE
**Tokelau**	Good (60)	CDC	2001 outbreak
**Tonga**	Good (71)	CDC	2007 outbreak and returning traveller reports
**Turks and Caicos Islands**	Indeterminate (10)	WHO	Low level background case data, reported cases in peer-reviewed articles and SE
**Tuvalu**	Poor (30)	CDC	1998 outbreak, description of DHF and SE
**Wallis and Futuna**	Good (67)	CDC	1998 outbreak, PCR virus typing and SE

Table 1 shows countries for which we identified a consensus better than indeterminate on dengue-presence, but was listed as dengue-absent by the WHO or the CDC. WHO = World Health Organization, CDC = Centers for Disease Control, SE = supplementary evidence, PCR = polymerase chain reaction, DHF = dengue haemorrhagic fever.

The majority of these newly identified dengue-present countries were in Africa and the evidence type that allowed greatest identification was returning traveller reports. These sporadic reports established preliminary evidence, which we improved with supplementary evidence and questionnaire retrieval to clarify dengue status if possible (Table 2). Outside of Africa, the remaining newly identified countries were almost exclusively islands in the Indian and Pacific Oceans and in the Caribbean. The reason for a lack of dengue presence identification by health organisations here is likely the longer interval between epidemics in small isolated nations, resulting in sparse data which different health organisations have interpreted inconsistently. Inclusion of less official surveillance evidence, such as ProMED reports, that detected background case loads alongside officially reported outbreaks allowed our distinction of these areas as in fact dengue-present.

**Table 2:** Evidence consensus class changes in Africa as a result of including supplementary evidence and questionnaire responses.

Country	Evidence consensus class excluding questionnaires and supplementary evidence	Evidence consensus class including questionnaires and supplementary evidence
**Equatorial Guinea**	Poor (absence)	Indeterminate
**Mauritania**	Poor (absence)	Indeterminate
**Niger**	Poor (absence)	Indeterminate
**Central African Republic**	Indeterminate	Poor
**Liberia**	Indeterminate	Poor
**Malawi**	Indeterminate	Poor
**Uganda**	Indeterminate	Poor
**Zimbabwe**	Indeterminate	Poor
**Angola**	Poor	Moderate
**Benin**	Poor	Moderate
**Chad**	Poor	Moderate
**Guinea-Bissau**	Poor	Good
**Cameroon**	Moderate	Good
**Côte d'Ivoire**	Good	Complete
**Nigeria**	Good	Complete
**Sierra Leone**	Good	Complete

All classes refer to consensus on dengue presence unless otherwise stated. Supplementary evidence was available for all countries in this table, while questionnaire responses were received from Cameroon, Burkina Faso, Malawi, Guinea-Bissau, Gabon and Côte d'Ivoire.

A total of 3.97 billion people live in these 128 countries outside the indeterminate consensus class. Of these, 824 million live in urban and 763 million in peri-urban areas. These numbers therefore constitute plausible preliminary estimates for the maximum possible population at any risk of dengue transmission. We expect more comprehensive population at risk calculations to refine this figure and quantify levels of risk in our future work, allowing us to give a more accurate estimate.

**Figure 8:**
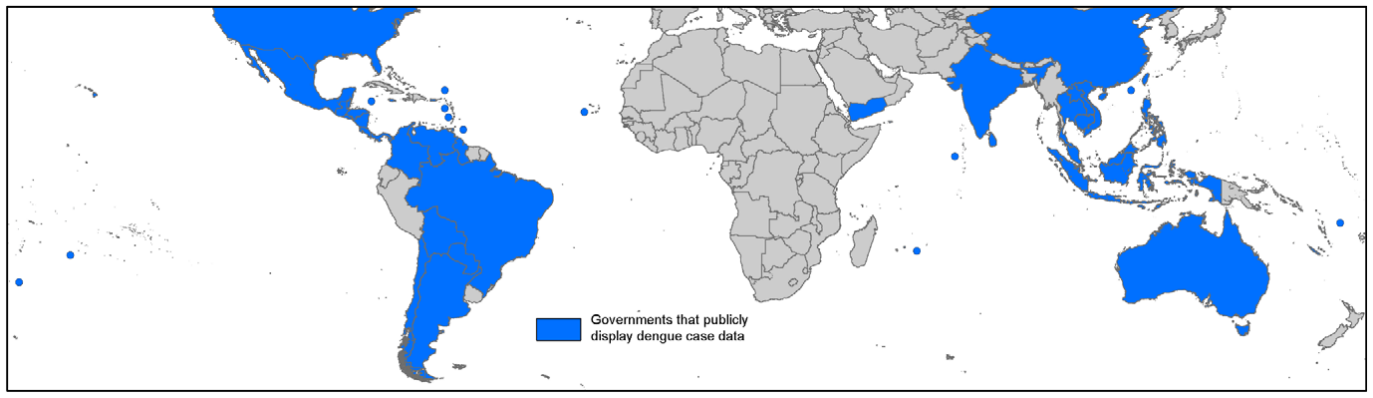
The worldwide variation in governments that publicly display dengue data. The map shows governments that at a minimum display dengue case data at a national level yearly via their official Ministry of Health website.

Public display of dengue data varied by continent (Figure 8). In total, 46 of 128 dengue-present countries displayed annual dengue case numbers. Of these, the highest reporting coverage was observed in Asia and the Americas where 55% and 57% of countries respectively reported dengue publically. This figure was comparably worse in the Pacific (29%) and Africa, Saudi Arabia, Yemen and the western Indian Ocean islands (Africa+) where just 7% of dengue-present countries publicly report dengue and none on mainland continental Africa. There were no regional patterns in the level of dengue case data provided, although the publicising of epidemiological weeks in some Central and South American countries tended to provide higher levels of detail. Deaths due to DHF/DSS/severe dengue were far less commonly reported, although the data are available for some Central American countries. Even allowing for variable internet usage and endogenous public health systems, we highlight the magnitude of disparity in countries' provision of freely available dengue data.

### The Americas

Dengue presence is well documented in the Americas with a continuous set of good- or complete- consensus countries from southern Brazil to the Mexico-U.S.A. border (Figure 3). However, a general regional classification was not producible as in some cases such as Montserrat and Saint Vincent and the Grenadines, where moderate rather than good consensus was found. With only 22% of dengue-present Caribbean countries displaying dengue data publically, dengue status in these small island nations that are characterised by longer inter-epidemic periods proved considerably more heterogeneous. This was mainly due to a lack of confirmed indigenous cases during recent epidemics.

Other regions of uncertainty reflect dynamic dengue status at the limits of the disease distribution. Lower consensus estimates in areas of Florida and Argentina result from reliance on smaller amounts of evidence from recent epidemics. Although the disease extent is better described in Florida (both in terms of resolution and consensus) due to greater data availability, uncertainty is still present due to the unknown persistence of recent events. A similar pattern of uncertainty exists in Texas but for different reasons, being that the occurrence evidence is older and six of seven counties have no record of occurrence since the late 1980s.

### Africa+

A total of 58% of Africa+ countries had a good consensus or better but Africa still showed the highest levels of uncertainty in countries with poor consensus. Concentrations of higher consensus were identified in East and West Africa (Figure 4). Multiple seroprevalence surveys over several years [31], [32], [33], [34], [35] made the most significant contribution in defining East Africa's higher-consensus cluster which ranges from Sudan to Tanzania with only Uganda, Rwanda and Burundi exhibiting poor or worse evidence consensus. In addition to this, evidence of outbreaks in coastal areas of Yemen, Saudi Arabia and some evidence of spill-over into Egypt added certainty to the definition of the East Africa high-consensus cluster. Although not as contiguous a tract of countries, a higher-consensus region also exists in West Africa from Senegal to Gabon. Inclusion of reported dengue cases in travellers and soldiers returning from West Africa was available for 13 countries and proved the most useful information in this region.

Outside of these higher-consensus regions, evidence consensus is low and a series of countries with moderate or worse consensus can be identified from Chad to Mozambique with only the Democratic Republic of Congo exhibiting good evidence consensus. For many of these countries, there are sporadic reports of dengue occurrence combined with poor disease surveillance and a general lack of data. Dated seroprevalence surveys in areas where many other arboviruses are circulating did little to increase certainty. These factors result in a positive evidence consensus that is nevertheless highly uncertain in large portions of Africa. Even where evidence was available from contemporary epidemics, such as in the case of the western Indian Ocean islands, it was often devalued because there was a lack of clinical differentiation between dengue and chikungunya despite epidemics coinciding. The lack of clear clinical distinction between the two diseases [36] makes the scale of dengue here difficult to identify and as a result, some countries (such as Reunion) were identified as having low consensus.

Despite the widespread uncertainty in dengue status in many African countries, we were able to differentiate multiple levels of uncertainty. Angola and Mozambique both show lower consensus due to dated evidence forms, yet they are still distinguishable from countries with no evidence or just sporadic occurrences such as Zambia or Congo.

### Asia

A wide variety of contemporary evidence allowed us to display a near continuous distribution of good or complete evidence consensus countries from Indonesia to as far north as Pakistan and Zhejiang, China (Figure 5). Within this dengue-present area, 58% of countries publicly displayed dengue data (Figure 8) and many reported dengue case data with a high spatial resolution. Minor exceptions to this continuous distribution occur in southern China and North-East India largely due to a lack of contemporary evidence. In Gunagxi and Hainan there is little research interest or case data in recent years despite occurrences in urban centres further along the Chinese coast [37], [38], [39]. In North-East India, lower consensus was observed due to a lack of reported cases in recent years combined with the arrival of chikungunya in the area which complicates any potential dengue reporting [40].

Evidence consensus in Asia is lowest in central Asia where contemporary dengue occurrence records combined with low surveillance capacity results in an unclear boundary to the disease. While evidence for dengue presence in the lowland urban centres of Pakistan is accurate and contemporary, reports from the more remote north-west provinces are contemporary, but not accurate [41], [42], [43]. This makes determining the extent further north into remote and data-deficient areas of Afghanistan and central Asia difficult to assess. We also found serologic evidence consistent with dengue presence in Turkey [44] and Kuwait [45], reducing evidence consensus for absence in these countries despite not belonging to any known cluster of dengue-present countries.

### Europe

Although no countries in Europe were defined as dengue-present, sporadic indigenous transmission events have lowered consensus in some countries (Figure 6). Since the invasion and spread of *Ae. albopictus* along the Mediterranean coast [46], indigenous dengue transmission has been detected in Marseilles, France and Korčula, Croatia (both regions have moderate consensus on dengue absence) and chikungunya has been found in Italy (having good consensus on dengue absence) [47], [48], [49]. These isolated events do not in themselves confer dengue presence, but increased surveillance will be required in light of the *Ae. albopictus* invasion to maintain this status. This, combined with the lower levels of healthcare expenditure, has led to an observed greater uncertainty in some eastern European states.

### Australia and Pacific Islands

In general, consensus on dengue presence and absence was well defined across Australia and the Pacific islands, with 85% of countries showing good or complete evidence consensus (Figure 7). Where low consensus was observed, it was largely due to a lack of contemporary evidence despite Pacific-wide dengue epidemics such as in Niue, Nauru, Tuvalu and Papua New Guinea. The duration between epidemics is typically longer in the Pacific and consensus is subject to continual change; for example, in the Marshall islands evidence consensus was upgraded from moderate to complete in the wake of the December 2011 epidemic, which came two decades after the last reported epidemic [50]. Such fluctuation is not entirely unexpected from remote, isolated communities, however. Even though evidence consensus decreases with time, it still remains positive, allowing for potential re-occurrence.

Lower evidence consensus was observed for Papua New Guinea due to a lack of reported case data since the 1980's, yet multiple literature sources suggest that dengue is still widespread [51], [52], [53]. While dengue occurrence is closely documented in some counties on the Australian coast, the serologic results from Charters Towers has contributed to uncertainty over the inland extent of the disease in Queensland [54]. Only the governments of Australia, New Caledonia and the Solomon Islands report dengue case numbers publicly. Considering the long intervals between epidemics in the Pacific, it is perhaps unsurprising that this is not a priority.

## Discussion

Here we present the distribution of dengue virus transmission as assessed by evidence-based consensus. By emphasising the need for accurate, contemporary evidence through a weighted scoring system, we were able to identify areas where dengue status was more uncertain, particularly in Africa and Central Asia, and identify evidence gaps where surveillance might be better targeted to more accurately assess dengue status. By including a wide variety of evidence we were able to cast doubt on dengue status in countries previously described by health organisations as dengue-absent.

While many studies have focussed on the future threat of dengue as a result of range expansion or climate change, this is the first to assess the entirety of knowledge regarding the extent of current virus transmission. We have found that evidence of dengue virus transmission is temporally dynamic and that a contemporary map must emphasise evidence by weighting it appropriately. By increasing temporal resolution to one inter-epidemic period, we have extended the approach of Van Kleef *et al.* [5] who used evidence from literature searches to produce distribution maps pre- and post- 1975. Focussing on a higher resolution timescale for dengue evidence is necessary if we are to infer changes in the evidence-based distribution of dengue.

The suggestion that dengue is an under-recognised problem in Africa is not a new one [55], [56], [57], but here we present a detailed summary of the specific gaps in evidence that exist in different regions. We show that consensus mapping is flexible to regional differences in evidence availability and as such can produce meaningful outputs in resource-high and low settings. The evidence that dengue is widespread in Africa implies that the continent is underrepresented by occurrence points in the model-based approaches that have been used to investigate the distribution of dengue so far [1], [10], [11]. If we are to estimate the burden of dengue in Africa with any fidelity, available data and their underlying assumptions need to be reassessed.

Evidence consensus maps provide a more informative alternative to existing country-level maps, such as those provided by the WHO [12] and CDC [58]. As presence or absence exists on a continuous scale of certainty, evidence consensus approaches are more adaptable to incorporating diverse forms of dengue evidence ignored by these organisations in producing their estimates. While we show that different evidence weightings in our scoring system do not significantly alter the result, we were unable to formalise a statistical validation of these weightings due to lack of a training dataset. Our results provide the best estimate thus far of where such data are most needed and comparisons with higher-consensus countries in similar settings should form the first step in directing regional surveillance. Development of methodologies to make approaches such as consensus mapping more reliable is needed as dengue status will increasingly rely on harder-to-quantify evidence types, such as internet search engine terms [59] and multi-language internet text-mining systems [60], [61]. The success of automated disease surveillance systems such as HealthMap [16], [17], and Biocaster [60], [63] have already been demonstrated. We believe evidence consensus provides the best platform for integrating these diverse forms of information now available for disease occurrence to create an up-to-date, high-resolution map of dengue evidence, whilst retaining important assessments of certainty. We also intend to extend our own data collection and accessibility with a new website linked to the Global Health Network (http://globalhealthtrials.tghn.org/) that will allow evidence contribution from members and will provide a key platform for display of dengue data and consensus maps. Although the current approach was used to map the distribution of dengue, minor modifications to the scoring system would allow it to be utilised for a variety of diseases for which the quality of presence evidence is spatially variable.

In this work, our aim was to produce a standardised methodology that used the largest variety of evidence to assess country dengue status, whilst still being applicable in diverse healthcare settings and suitable at multiple spatial scales. We considered the stark contrast in evidence available in Africa as compared to the rest of the world. Our results show that the inclusion of supplementary evidence (used in 44% of African countries but only 11% of the rest), healthcare expenditure information (for case data absences) and questionnaires increased evidence consensus in these countries without impacting the methodology applied to the rest of the world. Similarly, we are aware that increasing resolution to Admin1 or Admin2 level may well reduce the evidence available for calculating evidence consensus in each area compared to country-level calculations. As a result, we carefully chose which countries should have increased spatial resolution based on whether sufficient evidence was available in smaller administrative units. We also limited the selection of these countries to those at the limits of the disease's distribution, as data deficiencies in these regions more accurately represent the uncertainty on dengue status given the dynamic nature of global dengue spread. Here we present the most flexible methodology available, to date, for overcoming these problems. We have demonstrated that a systemic approach with relevant optional categories has allowed us to utilise the maximum variety of evidence available for assessing dengue status in the widest variety of situations.

We also openly provide a full list of evidence for each country by category (Table S1). We intend to continue data acquisition by including more endogenous, local evidence through questionnaires and local language search methods, which we expect will allow us to further customise our methodology and assess dengue status in places where we are currently uncertain.

Mapping by evidence consensus is a useful approach to quantifying contemporary disease evidence and can be further integrated with geo-spatial modelling to produce worldwide continuous surfaces of dengue risk [64]. Current mapping approaches use presence/absence expert opinion maps to sample pseudo-presence or pseudo-absence points to increase the number of data points on which to base their prediction [65], [66], [67], [68]. Pseudo-sampling could be improved by using the continuous scale of evidence consensus to either affect sample number or point weight within the geo-spatial model. This will lead to more robust, higher resolution dengue maps which are currently in progress [69]. By combining uncertainty assessment from consensus mapping with high-resolution predictions using geo-spatial modelling, we will be able to make more accurate predictions of disease burden with associated confidence intervals made explicit. This will then provide a series of up-to-date assessments of global dengue distribution, thus providing key information to assess dengue spread and the impact of control measures.

## Supporting Information

Figure S1 **Geographic locations of occurrence data globally.** Country colouring is based on evidence based consensus (see main manuscript) with green representing a complete consensus on dengue absence and red a complete consensus on dengue presence.
(TIF)

Figure S2 **Geographic locations of occurrence data in Africa+.** Country colouring is based on evidence based consensus (see main manuscript) with green representing a complete consensus on dengue absence and red a complete consensus on dengue presence.
(TIF)

Figure S3 **Geographic locations of occurrence data in Asia.** Country colouring is based on evidence based consensus (see main manuscript) with green representing a complete consensus on dengue absence and red a complete consensus on dengue presence.
(TIF)

Figure S4 **Geographic locations of occurrence data in the Americas.** Country colouring is based on evidence based consensus (see main manuscript) with green representing a complete consensus on dengue absence and red a complete consensus on dengue presence.
(TIF)

Figure S5 **Geographic locations of occurrence data in Australia.** Country colouring is based on evidence based consensus (see main manuscript) with green representing a complete consensus on dengue absence and red a complete consensus on dengue presence.
(TIF)

Figure S6 **Number of occurrence samples per year globally (a) and for Africa+ (b), Asia, (c) the Americas and Australia (d).**
(TIF)

Figure S7 **Map of evidence types used for each national and subnational area.** Figure S7 shows the different evidence categories used in assessing evidence consensus for each country and Admin1/2 area. HO = health organisation status, L = literary evidence, CD = case data, SE = supplementary evidence, PO = professional opinion.
(TIF)

Protocol S1 **An outline of the dengue occurrence point database construction and content.** Data sources, searches and exclusion criteria are outlined and the method of geo-positioning explained. The regional bias of available occurrence points is also given in the accompanying figures. Table S1 shows the collection of evidence used to assess evidence consensus for each country and Admin1 and Admin2 areas. Details of the scoring system can be found in the Methods section of the main manuscript. Scores for each category are highlighted in red. Evidence consensus is calculated as the percentage of the maximum possible score (see Fig. 2 in the main manuscript). HE = healthcare expenditure, DENV = dengue virus, DHF = dengue haemorrhagic fever, DSS = dengue shock syndrome, PCR = polymerase chain reaction, DF = dengue fever.
(DOC)

Table S1 **The collection of evidence used to assess evidence consensus for each country and Admin1 and Admin2 areas.** Details of the scoring system can be found in the Methods section of the main manuscript. Scores for each category are highlighted in red. Evidence consensus is calculated as the percentage of the maximum possible score (see Fig. 2 in the main manuscript). HE = healthcare expenditure, DENV = dengue virus, DHF = dengue haemorrhagic fever, PCR = polymerase chain reaction, DF = dengue fever.
(DOC)
