# Supplementary figures and images for: Refining the Global Spatial Limits of Dengue Virus Transmission by Evidence-Based Consensus
Source: PLoS Negl Trop Dis. 2012 Aug 7;6(8):e1760. doi: 10.1371/journal.pntd.0001760 (PMC3413714; doi:10.1371/journal.pntd.0001760)

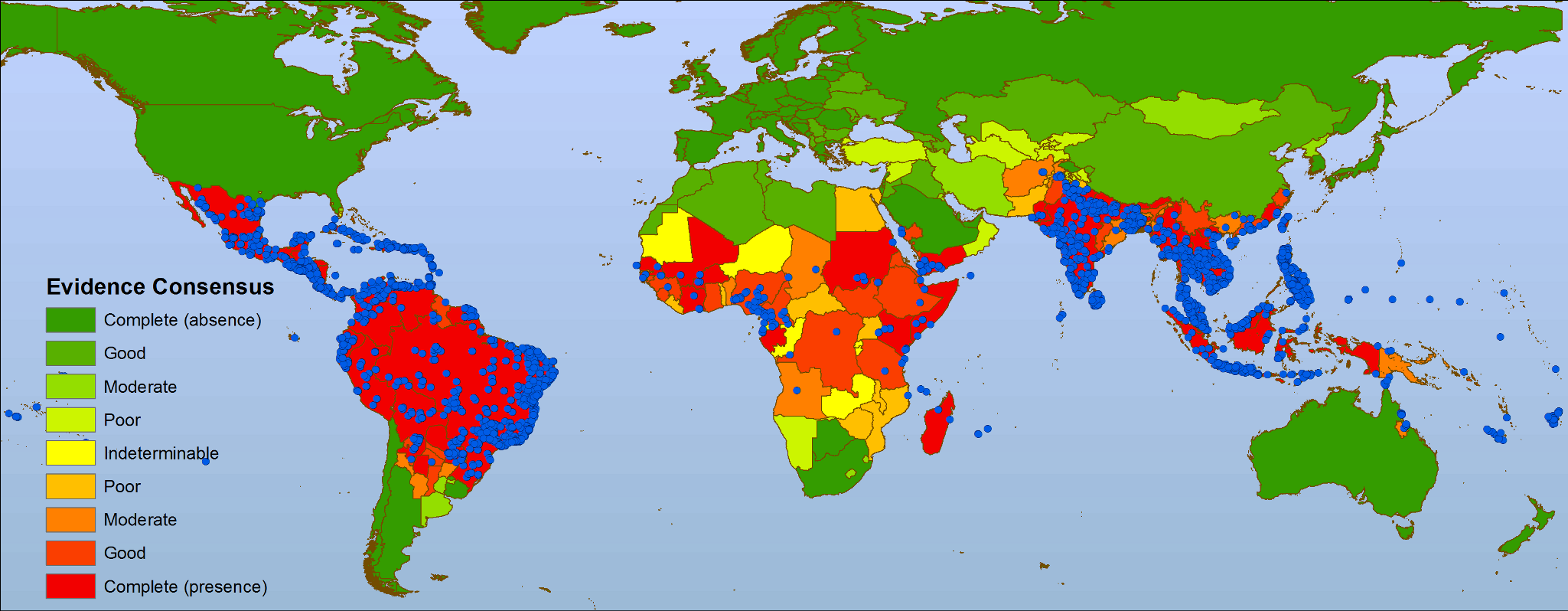

Supplement: Figure S1 — Geographic locations of occurrence data globally. Country colouring is based on evidence based consensus (see main manuscript) with green representing a complete consensus on dengue absence and red a complete consensus on dengue presence. (TIF) [file pntd.0001760.s001.tif]

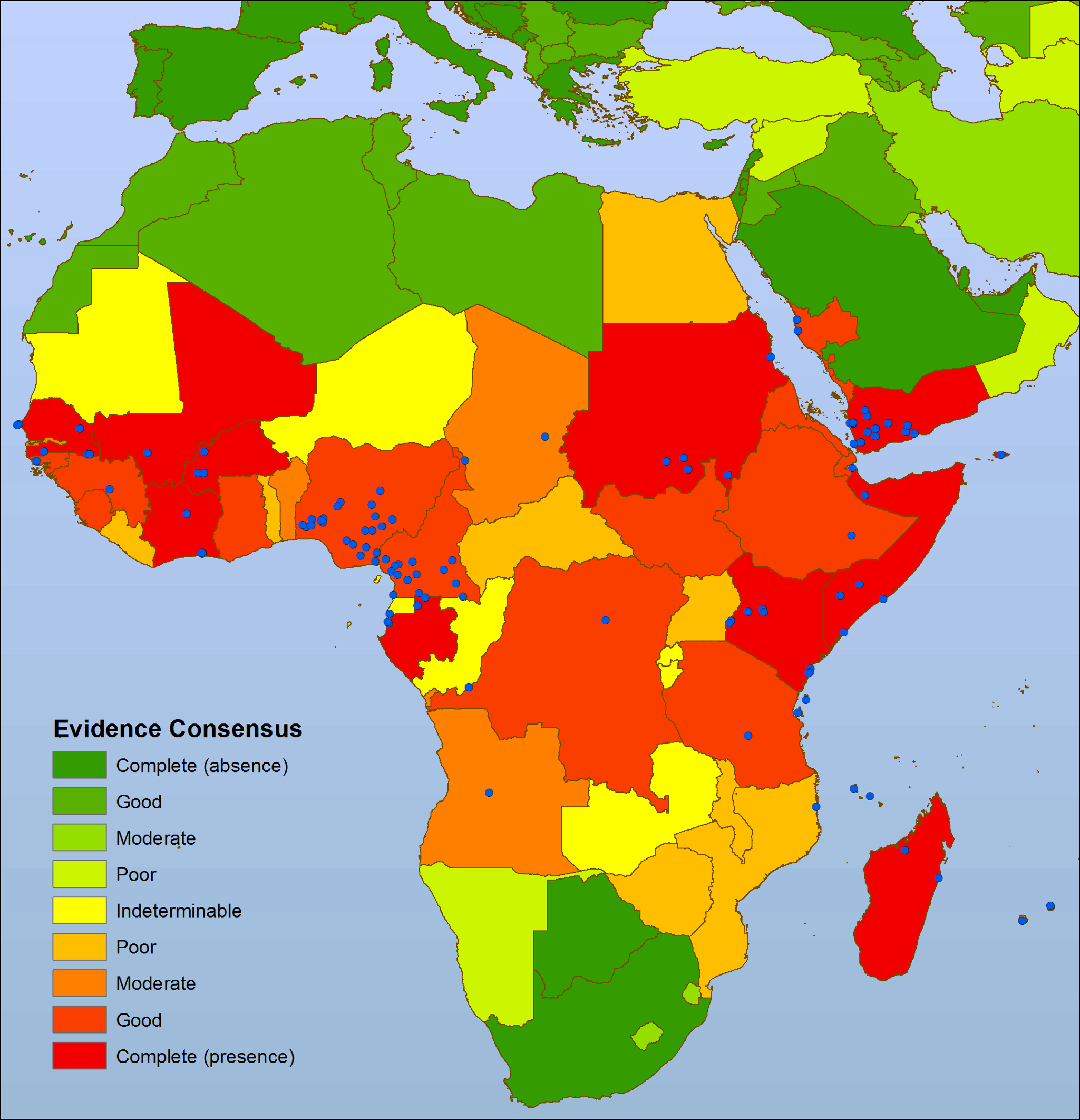

Supplement: Figure S2 — Geographic locations of occurrence data in Africa+. Country colouring is based on evidence based consensus (see main manuscript) with green representing a complete consensus on dengue absence and red a complete consensus on dengue presence. (TIF) [file pntd.0001760.s002.tif]

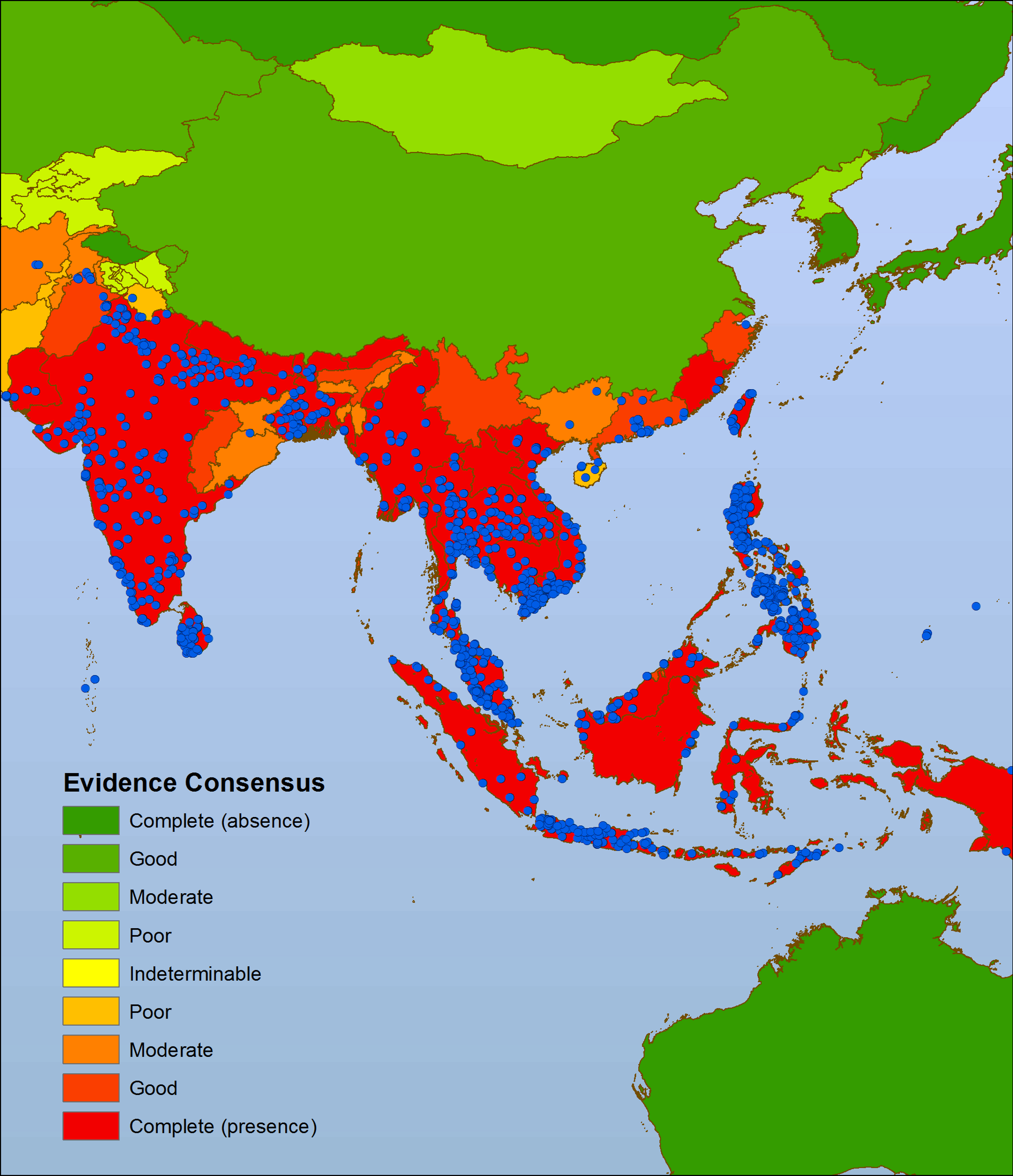

Supplement: Figure S3 — Geographic locations of occurrence data in Asia. Country colouring is based on evidence based consensus (see main manuscript) with green representing a complete consensus on dengue absence and red a complete consensus on dengue presence. (TIF) [file pntd.0001760.s003.tif]

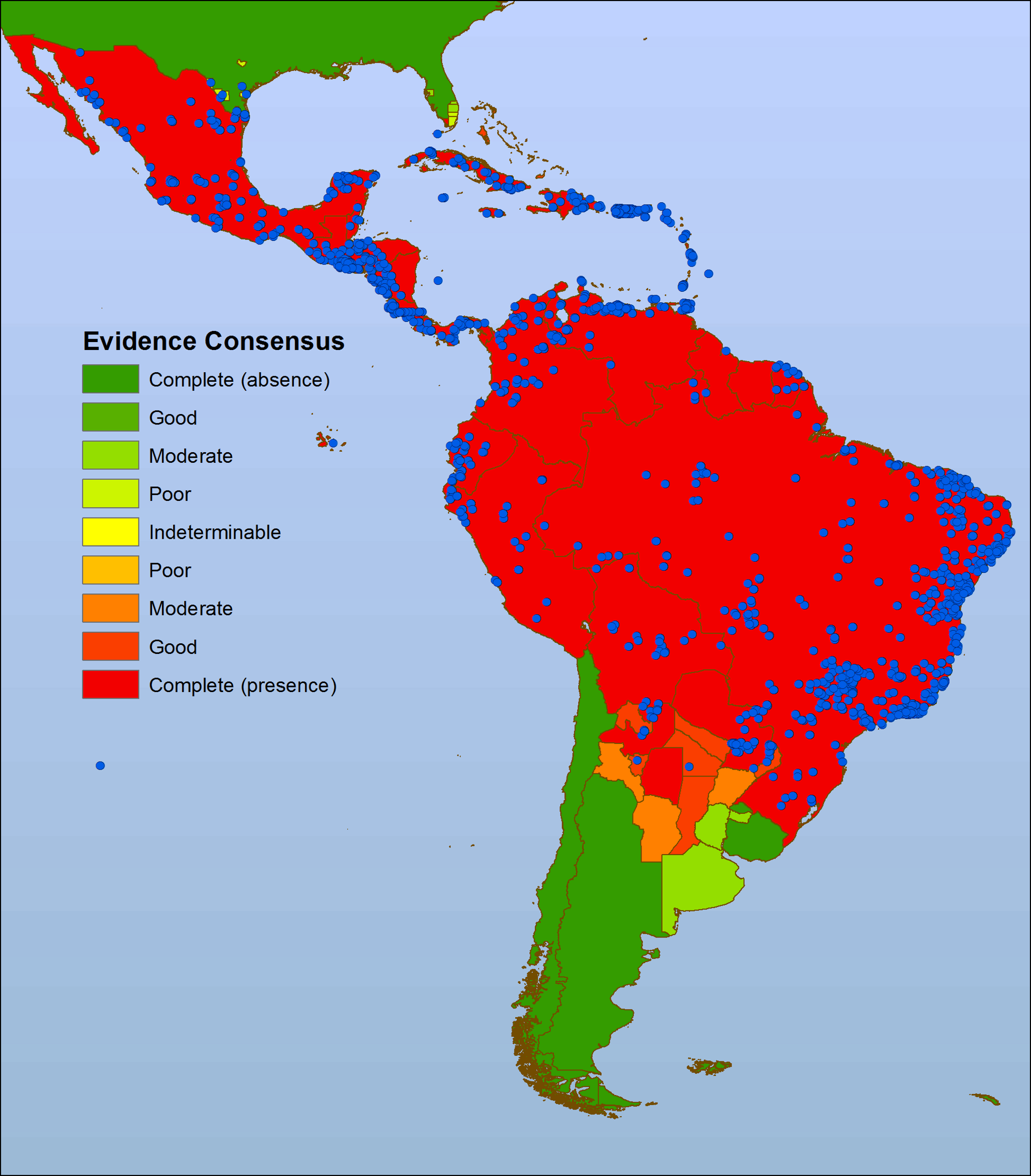

Supplement: Figure S4 — Geographic locations of occurrence data in the Americas. Country colouring is based on evidence based consensus (see main manuscript) with green representing a complete consensus on dengue absence and red a complete consensus on dengue presence. (TIF) [file pntd.0001760.s004.tif]

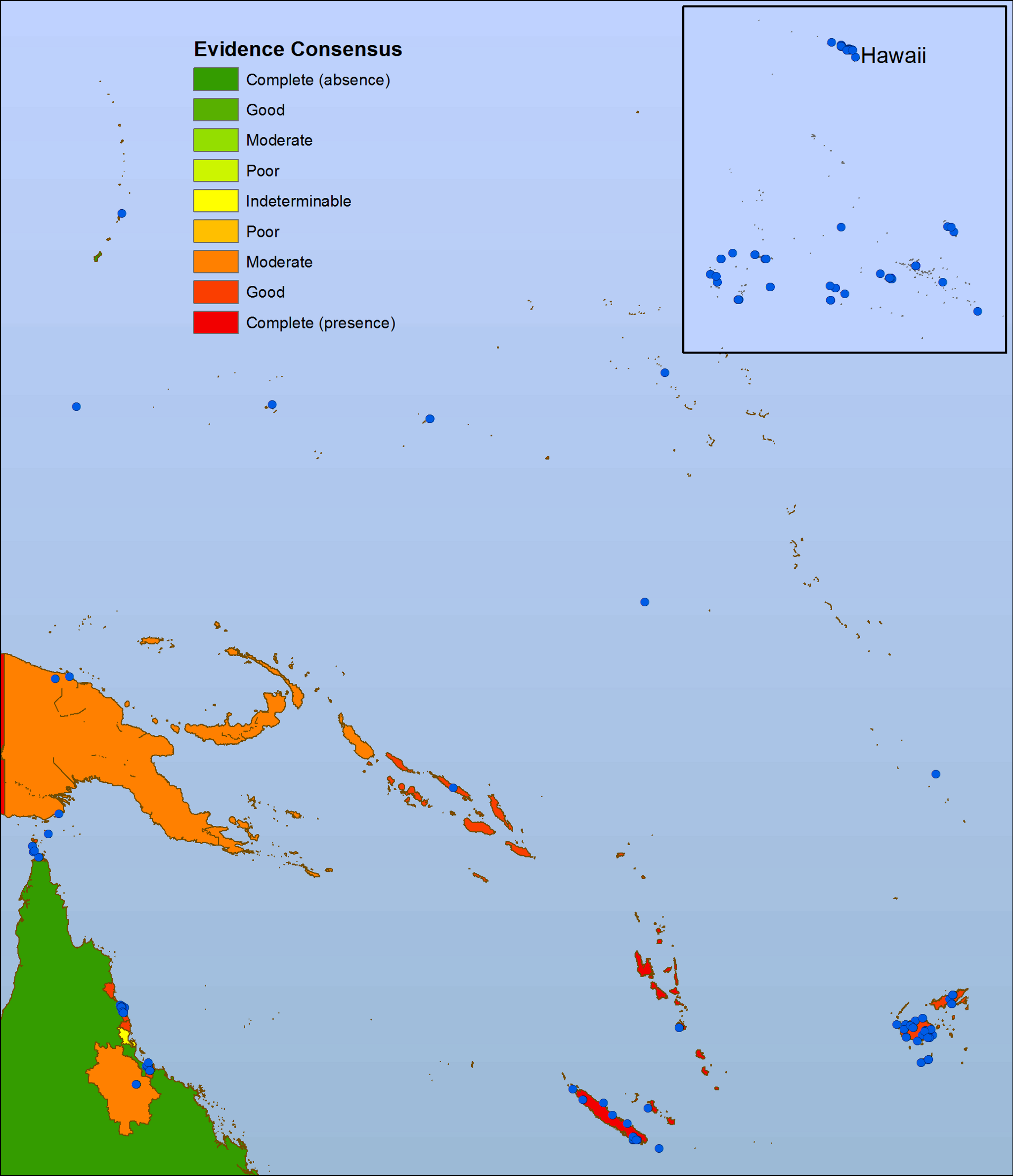

Supplement: Figure S5 — Geographic locations of occurrence data in Australia. Country colouring is based on evidence based consensus (see main manuscript) with green representing a complete consensus on dengue absence and red a complete consensus on dengue presence. (TIF) [file pntd.0001760.s005.tif]

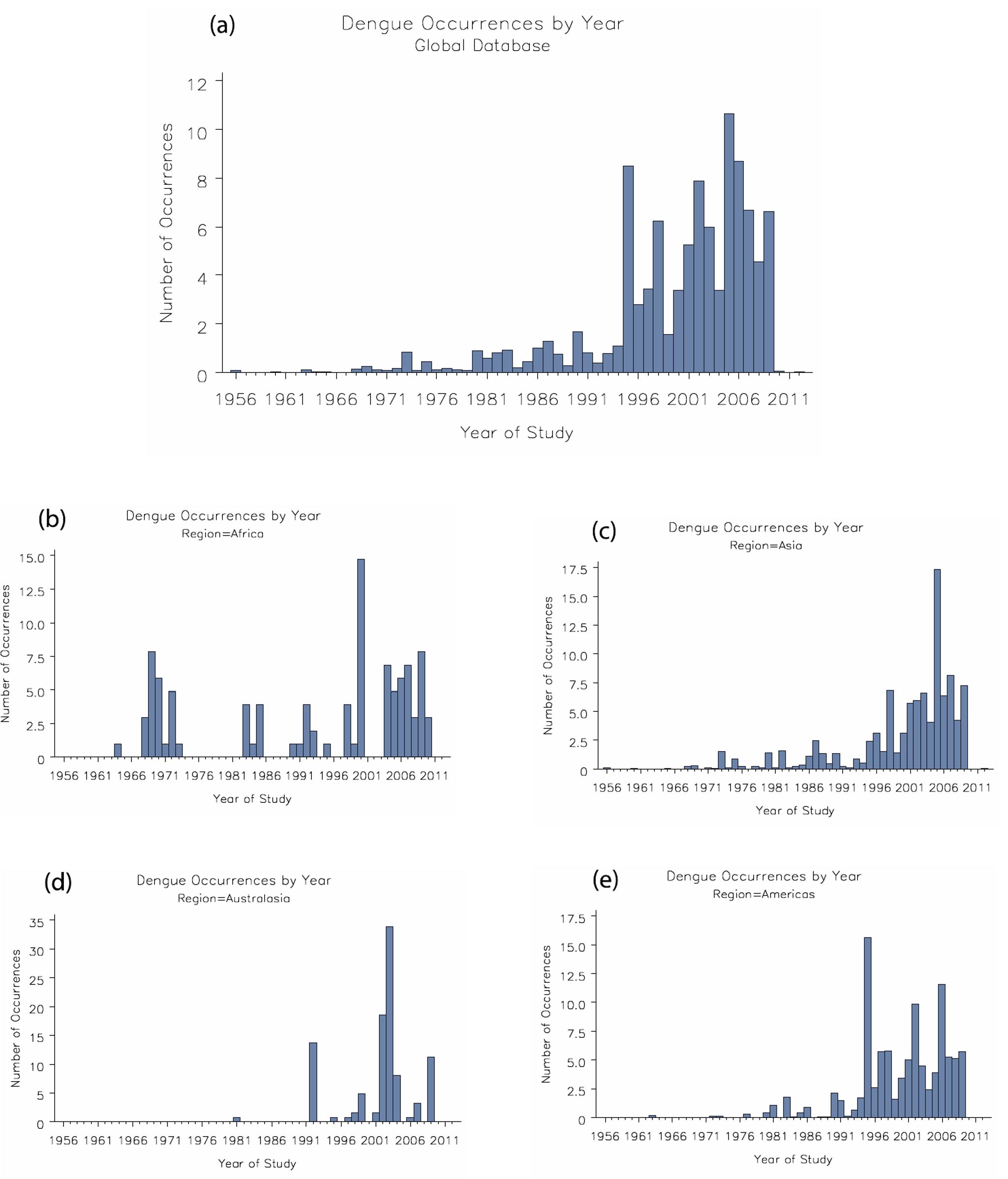

Supplement: Figure S6 — Number of occurrence samples per year globally (a) and for Africa+ (b), Asia, (c) the Americas and Australia (d). (TIF) [file pntd.0001760.s006.tif]

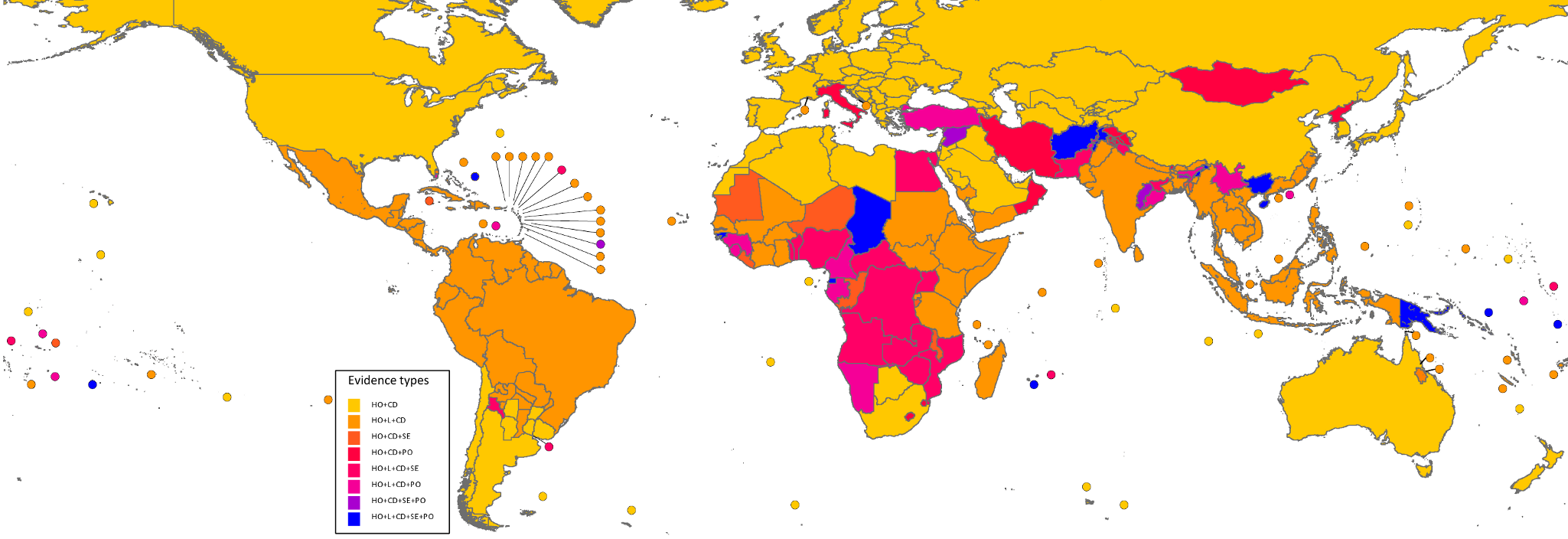

Supplement: Figure S7 — Map of evidence types used for each national and subnational area. Figure S7 shows the different evidence categories used in assessing evidence consensus for each country and Admin1/2 area. HO = health organisation status, L = literary evidence, CD = case data, SE = supplementary evidence, PO = professional opinion. (TIF) [file pntd.0001760.s007.tif]
