# Supplementary material for: Refining the Global Spatial Limits of Dengue Virus Transmission by Evidence-Based Consensus
Source: PLoS Negl Trop Dis. 2012 Aug 7;6(8):e1760. doi: 10.1371/journal.pntd.0001760 (PMC3413714; doi:10.1371/journal.pntd.0001760)
Supplement: Protocol S1 — An outline of the dengue occurrence point database construction and content. Data sources, searches and exclusion criteria are outlined and the method of geo-positioning explained. The regional bias of available occurrence points is also given in the accompanying figures. Table S1 shows the collection of evidence used to assess evidence consensus for each country and Admin1 and Admin2 areas. Details of the scoring system can be found in the Methods section of the main manuscript. Scores for each category are highlighted in red. Evidence consensus is calculated as the percentage of the maximum possible score (see Fig. 2 in the main manuscript). HE = healthcare expenditure, DENV = dengue virus, DHF = dengue haemorrhagic fever, DSS = dengue shock syndrome, PCR = polymerase chain reaction, DF = dengue fever. (DOC) [file pntd.0001760.s008.doc]

**Protocol S1. Assembly of the supplementary dengue database**

**S1.1 Overview**

The dengue database comprises occurrence data linked to point or polygon locations, derived from peer-reviewed literature. Data sources are described in full here. To collate the peer-reviewed database, literature searches were undertaken using the major search engines and the resulting articles were manually reviewed. Geo-spatial information for 7,419 occurrences of dengue was extracted using site descriptions within 2,838 of the articles and recorded as specific coordinates or by a polygon definition when this was the only information available. All data entries from both data sources were manually checked by the authors and then underwent a series of routine quality control procedures to ensure correct geo-positioning.

**S1.2 Peer-reviewed Literature Search**

PubMed (<http://www.ncbi.nlm.nih.gov/entrez/query.fcgi>) 1920 to 2009 was searched using term “dengue”. The MESH term technology used in the PubMed citation archive ensured all pseudonyms were automatically included (<http://www.nlm.nih.gov/mesh/2008/MBrowser.html>) in the searches. The same process was repeated for ISI Web of Science ([http://wok.mimas.ac.uk](http://wok.mimas.ac.uk/)) and ProMED ([http://www.promedmail.org](http://www.promedmail.org/)). The searches were last updated on 8^th^ February 2012. No language restrictions were placed on these searches, however, only those citations with a full title and abstract were downloaded into Endnote version 10 (Thomson ResearchSoft, USA, [http://www.endnote.com](http://www.endnote.com/)). A total of 5,876 references were retrieved and 2,883 unique articles were identified as potentially containing useable location data. The full articles were obtained for 2,838 or 98.4% and the references are listed in S1.6.

In-house language skills allowed processing of all English, French, Portuguese and Spanish articles. Russian articles with data were translated and included in the database. We were unable to extract information from a small number of Turkish, Polish, Hebrew, Italian, German and Chinese articles.

**S1.3 Disease data and imported cases**

Confirmed occurrences of dengue virus transmission, dengue haemorrhagic fever or a specific dengue serotype found within the peer reviewed literature were recorded as a dengue occurrence datapoint. A report of multiple cases (i.e. an outbreak) was reported as a single occurrence datapoint, however, later outbreaks at the same location were recorded as new occurrence datapoints. Reports of autochthonous (locally transmitted) cases or outbreaks were entered as an occurrence within the country. If imported cases were reported with information on the site of contagion, they were recorded as an occurrence from the country of contagion within the database. If imported cases were reported with no information about the site of contagion they were not entered into the database. If an imported case led to an outbreak/local transmission within the recipient country and location information was available for the site of initial contagion and the site of the outbreak, this was recorded as two occurrences; one in the country of contagion and one in the country where the outbreak occurred.

Data from both sources was entered into a bespoke PostgreSQL database that links disease data to spatial data. A link to the PDF library was also included in the database.

**S1.4 Geo-positioning occurrence points**

All available location information in the peer-reviewed literature was extracted from each article. The site name was used together with all contextual information provided about the study site position, for example the approximate distance from the nearest city or the district within which the site was located. The site name and contextual information were used together to locate the study site using Microsoft Encarta (Microsoft Corporation, Redmond, WA) and Google Earth 5.1. Place names are often duplicated within a country so the contextual information was used to ensure the right site was selected. Where the site name was not found, the contextual information was used to scan sites in the approximate area to check for names that had been transliterated in Microsoft Encarta/Google Earth in a different way to the published article (e.g. Imichli and Imishly). If the site was not located using Microsoft Encarta or Google Earth, a range of online georeferencing resources were tried including Getty Thesaurus (<http://www.getty.edu/research/conducting_research/vocabularies/tgn/index.html>), Alexandria (<http://middleware.alexandria.ucsb.edu/client/gaz/adl/index.jsp>) and Falling Rain (<http://www.fallingrain.com/world/>). If the study site could be georeferenced to a specific place, it was recorded as a point location. In total, 3,286 point locations from the peer-reviewed literature were included in the database at this stage. If the study site could only be identified at an administrative area level (e.g. province or district, etc), it was recorded as a polygon and the centroid (mean centre) was derived to give a latitude and longitude for the site. A total of 5,234 polygon locations from the peer-reviewed literature were recorded in the database. All point locations and polygon centroids were converted to decimal degrees before entering the quality control process described below.

**S1.5 Automatic Validation and Quality control**

All datapoints were crosschecked and validated using a standardised validation procedure. First a multi-polygon shapefile defining land/water boundaries was created as a template to validate geopositioned points (see Protocol S2: Environmental Covariates). This land/water shape file was rasterised to spatially overlapping raster pixels (the polygon boundaries must cover the centre of the raster pixel) at a 5 x 5 km resolution and was used to ensure all disease occurrence points were positioned on a valid land pixel.

Any points that met the following criteria were excluded from the database:

1. Points found further than 10km from a land boundary. Points located less than 10km from a land boundary were repositioned to the nearest land raster cell.
2. Points representing the centroid of an administrative division having an area greater than 111km^2^ (1 degree at the equator).

Once the quality control procedure was complete, the final database contained 7,419 occurrence datapoints (including 3,237 point locations and 4,182 small polygon centroids) covering a period from 1956 to 2012. Maps displaying the 7,419 locations are provided in Figures S1-5, and the number of occurrence locations per sampling period are shown in Figure S6.

**S1.6 Bibliography of final endnote library**

1. Aaskov J (1995) DENGUE - COOK ISLANDS. rarotonga (Cook Islands): PROMED.

2. Aaskov J, Buzacott K, Thu HM, Lowry K, Holmes EC (2006) Long-term transmission of defective RNA viruses in humans and Aedes mosquitoes. Science 311: 236-238.

3. Abad DS, Ruiz-Ruiz FJ, Ballarin SM, Alvaro FM (2007) Fiebre y exantema tras viaje a Brasil. Rev Clin Esp 207: 369-370.

4. Abbasi A, Butt N, Sheikh QH, Bhutto AR, Munir SM, et al. (2009) Clinical Features, Diagnostic Techniques and Management of Dual Dengue and Malaria Infection. J Coll Physicians Surg Pak 19: 25-29.

5. Abbott M (2003) DENGUE/DHF UPDATE 2003 (20) -Laos/ Peru. In: ProMED-mail, editor. MASTA Health Report, Fri 16 May 2003 [edited] ed: PROMED.

6. Abell A, Smith B, Fournier M, Betz T, Gaul L, et al. (2007) Dengue hemorrhagic fever - U.S.-Mexico border, 2005 (Reprinted from MMWR, vol 56, pg 785, 2007). JAMA, J Am Med Assoc 298: 2130-2132.

7. Abhyankar AV, Dash PK, Saxena P, Bhargava R, Parida MM, et al. (2006) Comparison of a dipstick dot-ELISA with commercial assays for anti-dengue virus IgM antibodies. Viral Immunol 19: 630-636.

8. AbuBakar S, Wong PF, Chan YF (2002) Emergence of dengue virus type 4 genotype IIA in Malaysia. J Gen Virol 83: 2437-2442.

9. Adams B, Holmes EC, Zhang C, Mammen MP, Nimmannitya S, et al. (2006) Cross-protective immunity can account for the alternating epidemic pattern of dengue virus serotypes circulating in Bangkok. Proc Natl Acad Sci U S A 103: 14234-14239.

10. Adey-Jones M (2001) DENGUE/DHF UPDATES (03): 14 FEB 2001 [Thailand]. The Phuket Gazette Online, Tue 6 Feb 2001 [edited] ed: PROMED.

11. Agarwal JP, Bhattacharyya PC, Das SK, Sharma M, Gupta M (2009) Dengue Encephalitis. Southeast Asian J Trop Med Public Health 40: 54-55.

12. Agarwal R, Kapoor S, Nagar R, Misra A, Tandon R, et al. (1999) A clinical study of the patients with dengue hemorrhagic fever during the epidemic of 1996 at Lucknow, India. Southeast Asian J Trop Med Public Health 30: 735-740.

13. Aggarwal A, Chandra J, Aneja S, Patwari AK, Dutta AK (1998) An epidemic of dengue hemorrhagic fever and dengue shock syndrome in children in Delhi. Indian Pediatr 35: 727-732.

14. Aggarwal P (1996) DENGUE/DENGUE HEMORRHAGIC FEVER - INDIA. In: Sciences AIIoM, editor. Division of Emergency Medicine. New Delhi: PROMED.

15. Ahmad R, Abdul Latiff AK, Abdul Razak S (2007) Myalgia Cruris Epidemica: an unusual presentation of dengue fever. Southeast Asian J Trop Med Public Health 38: 1084-1087.

16. Ahmad R, Ismail A, Saat Z, Lim LH (1997) Detection of dengue virus from field Aedes aegypti and Aedes albopictus adults and larvae. Southeast Asian J Trop Med Public Health 28: 138-142.

17. Ahmed S (2003) Vertical transmission of dengue: first case report from Bangladesh. Southeast Asian J Trop Med Public Health 34: 800-803.

18. Ahmed S, Ali N, Ashraf S, Ilyas M, Tariq WU, et al. (2008) Dengue fever outbreak: a clinical management experience. J Coll Physicians Surg Pak 18: 8-12.

19. Ahmed S, Ali N, Tariq WU (2007) Neurological manifestations as presenting feature in dengue Fever. J Coll Physicians Surg Pak 17: 236-237.

20. Ahmed S, Arif F, Yahya Y, Rehman A, Abbas K, et al. (2008) Dengue fever outbreak in Karachi 2006--a study of profile and outcome of children under 15 years of age. JPMA J Pak Med Assoc 58: 4-8.

21. Ahsan T (2008) Dengue fever: a regular epidemic? JPMA J Pak Med Assoc 58: 1-2.

22. Akram DS, Igarashi A, Takasu T (1998) Dengue virus infection among children with undifferentiated fever in Karachi. Indian J Pediatr 65: 735-740.

23. Alam R, Siddiqui FM, Rahman S, Haque AK, Sarker CB, et al. (2004) Management of dengue by the WHO guided national guidelines. Mymensingh Medical Journal 13: 43-47.

24. Albuquerque LM, Trugilho MRO, Neves-Ferreira AGC, Valente RH, Jurgilas PB, et al. (2006) Comparative proteomic analysis of plasmas from patients with dengue hemorrhagic fever and from healthy donors. Mol Cell Proteomics 5: S331-S331.

25. Albuquerque PL, Silva Junior GB, Diogenes SS, Silva HF (2009) Dengue and aplastic anemia--a rare association. Travel Med Infect Dis 7: 118-120.

26. Alcon-LePoder S, Drouet MT, Roux P, Frenkiel MP, Arborio M, et al. (2005) The secreted form of dengue virus nonstructural protein NS1 is endocytosed by hepatocytes and accumulates in late endosomes: Implications for viral infectivity. J Virol 79: 11403-11411.

27. Alejandria MM (2009) Dengue haemorrhagic fever or dengue shock syndrome in children. Clin Evid.

28. Alexander Diaz-Quijano F, Arali Martinez-Vega R, Elvira Ocazionez R, Angel Villar-Centeno L (2006) Evaluación de la determinación de IgM en suero agudo para el diagnóstico de dengue en un área endémica. Enferm Infecc Microbiol Clin 24: 90-92.

29. Alho MAM, Errea MI, Sguerra VL, D'Accorso NB, Talarico LB, et al. (2005) Synthesis and antiviral evaluation of some carbonucleoside analogues. J Heterocycl Chem 42: 979-983.

30. Ali M, Wagatsuma Y, Emch M, Breiman RF (2003) Use of a geographic information system for defining spatial risk for dengue transmission in Bangladesh: role for Aedes albopictus in an urban outbreak. Am J Trop Med Hyg 69: 634-640.

31. Ali N, Nadeem A, Anwar M, Tariq WU, Chotani RA (2006) Dengue fever in malaria endemic areas. J Coll Physicians Surg Pak 16: 340-342.

32. Ali N, Usman M, Syed N, Khurshid M (2007) Haemorrhagic manifestations and utility of haematological parameters in dengue fever: A tertiary care centre experience at Karachi. Scand J Infect Dis 39: 1025-1028.

33. Allwinn R, Hofknecht N, Doerr HW (2008) Dengue in travellers is still underestimated. Intervirology 51: 96-100.

34. Allwinn R, Schieferstein C, Glauke S, Doerr HW (1999) Rapid diagnosis of primary dengue fever by the immunochromatographic test and by electron microscopy--a case report. Infection 27: 365-367.

35. Almeida MCD, Caiaffa WT, Assuncao RM, Proietti FA (2007) Spatial vulnerability to dengue in a Brazilian urban area during a 7-year surveillance. J Urban Health 84: 334-345.

36. Alvarez DE, Ezcurra ALD, Fucito S, Gamarnik AV (2005) Role of RNA structures present at the 3 ' UTR of dengue virus on translation, RNA synthesis, and viral replication. Virology 339: 200-212.

37. Alvarez DE, Lodeiro MF, Luduena SJ, Pietrasanta LI, Gamarnik AV (2005) Long-range RNA-RNA interactions circularize the dengue virus genorne. J Virol 79: 6631-6643.

38. Alvarez M, Pavon-Oro A, Rodriguez-Roche R, Bernardo L, Morier L, et al. (2008) Neutralizing antibody response variation against dengue 3 strains. J Med Virol 80: 1783-1789.

39. Alvarez M, Rodriguez-Roche R, Bernardo L, Vazquez S, Morier L, et al. (2006) Dengue hemorrhagic fever caused by sequential dengue 1-3 virus infections over a long time interval: Havana epidemic, 2001-2002. Am J Trop Med Hyg 75: 1113-1117.

40. An J, Zhou DS, Zhang JL, Morida H, Wang JL, et al. (2004) Dengue-specific CD8(+) T cells have both protective and pathogenic roles in dengue virus infection. Immunol Lett 95: 167-174.

41. AnandaRao R, Swaminathan S, Fernando S, Jana AM, Khanna N (2005) A custom-designed recombinant multiepitope protein as a dengue diagnostic reagent. Protein Expr Purif 41: 136-147.

42. AnandaRao R, Swaminathan S, Fernando S, Jana AM, Khanna N (2006) Recombinant multiepitope protein for early detection of dengue infections. Clin Vaccine Immunol 13: 59-67.

43. AnandaRao R, Swaminathan S, Khanna N (2005) The identification of immunodominant linear epitopes of dengue type 2 virus capsid and NS4a proteins using pin-bound peptides. Virus Res 112: 60-68.

44. Anantapreecha S, Chanama S, Nuegoonpipat AA, Naemkhunthot S, Sa-Ngasang A, et al. (2005) Serological and virological features of dengue fever and dengue haemorrhagic fever in Thailand from 1999 to 2002. Epidemiol Infect 133: 503-507.

45. Anderson KB, Chunsuttiwat S, Nisalak A, Mammen MP, Libraty DH, et al. (2007) Burden of symptomatic dengue infection in children at primary school in Thailand: a prospective study. Lancet 369: 1452-1459.

46. Anez G, Balza R, Valero N, Larreal Y (2006) Impacto económico del dengue y del dengue hemorrágico en el Estado de Zulia, Venezuela, 1997–2003. Rev Panam Salud Publica 19: 314-320.

47. Ang KT, Ruhaini I, Chua KB (2006) An epidemiological cluster pattern of dengue outbreak amongst close contacts in Selangor, Peninsular Malaysia. Med J Malaysia 61: 292-295.

48. Angel B, Joshi V (2009) Distribution of dengue virus types in Aedes aegypti in dengue endemic districts of Rajasthan, India. Indian J Med Res 129: 665-668.

49. Angibaud G, Luaute J, Laille M, Gaultier C (2001) Brain involvement in Dengue fever. J Clin Neurosci 8: 63-65.

50. Annis B, Nalim S, Hadisuwasono, Widiarti, Boewono DT (1990) Toxorhynchites amboinensis larvae released in domestic containers fail to control dengue vectors in a rural village in central Java. J Am Mosq Control Assoc 6: 75-78.

51. (1991) Current trends. Imported dengue--United States, 1990. MMWR Morb Mortal Wkly Rep 40: 519-520.

52. (1994) Dengue fever among U.S. military personnel--Haiti, September-November, 1994. MMWR Morb Mortal Wkly Rep 43: 845-848.

53. (1994) Imported dengue--United States, 1992. MMWR Morb Mortal Wkly Rep 43: 97-99.

54. (1995) Dengue type 3 infection--Nicaragua and Panama, October-November 1994. MMWR Morb Mortal Wkly Rep 44: 21-24.

55. (1996) Dengue fever at the U.S.-Mexico border, 1995-1996. MMWR Morb Mortal Wkly Rep 45: 841-844.

56. (1998) Dengue outbreak associated with multiple serotypes--Puerto Rico, 1998. MMWR Morb Mortal Wkly Rep 47: 952-956.

57. (1998) Imported dengue--United States, 1996. MMWR Morb Mortal Wkly Rep 47: 544-547.

58. (2000) Imported dengue--United States, 1997 and 1998. MMWR Morb Mortal Wkly Rep 49: 248-253.

59. (2001) Underdiagnosis of dengue--Laredo, Texas, 1999. MMWR Morb Mortal Wkly Rep 50: 57-59.

60. (2002) Imported dengue--United States, 1999 and 2000. MMWR Morb Mortal Wkly Rep 51: 281-283.

61. (1994) DENGUE-3 IN THE AMERICAS. Dengue Surveillance Summary. san Juan, Puerto Rico: PROMED.

62. (1990) Vector transmitted diseases in Central America, Belize and Panama. Epidemiological Bulletin Pan American Health Organization 11: 9-12.

63. (1994) Dengue Fever in Costa Rica and Panama. Epidemiological Bulletin Pan American Health Organization 15: 9-10.

64. (1996) Dengue and Dengue Hemorrhagic Fever, 1996. Epidemiological Bulletin Pan American Health Organization 17: 12-14.

65. (1997) Re-emergence of Dengue in the Americas. Epidemiological Bulletin Pan American Health Organization 18: 1-10.

66. (1999) Floods in Venezuela. Epidemiological Bulletin Pan American Health Organization 20: 1-2.

67. (2000) Dengue in Central America: The epidemics of 2000. Epidemiological Bulletin Pan American Health Organization 21: 4-8.

68. (1995) OUTBREAK OF DENGUE HEMORRHAGIC FEVER IN VALLEDUPAR CESAR - COLOMBIA. (Colombia): PROMED.

69. (1996) DENGUE/DENGUE HEMORRHAGIC FEVER - VENEZUELA: UPDATE. Weekly Epidemiology Record. WER/REH, VOL.71,NO 34,1996 p.257 ed: PROMED.

70. (1996) DENGUE/DHF - INDIA (12). Delhi: PROMED.

71. (1996) DENGUE - SINGAPORE. Singapore: PROMED.

72. (1996) DENGUE/DENGUE HEMORRHAGIC FEVER - MALAYSIA (2). PROMED Communicable disease news - 18 July 1996.

73. (1996) DENGUE - MINAS GERAIS, BRAZIL. In: newspaper EdM, editor. Estado de Minas newspaper, 10 May 1996 ed: PROMED.

74. (1996) DENGUE/DHF - INDIA (7). Disease outbreaks reported: PROMED.

75. (1997) DENGUE - TONGA (UPDATE). In: WHO/CLO, editor. Tonga: PROMED.

76. (1997) DENGUE/DHF - CUBA (02). PROMED.

77. (1998) DENGUE - PUERTO RICO. PROMED.

78. (1998) DENGUE - VIETNAM (06). In: ProMED-mail, editor: PROMED.

79. (1998) DENGUE - AUSTRALIA (QUEENSLAND) (08). CDI (Australia), Vol 22, No 1, January 22, 1998 ed: PROMED.

80. (1998) DENGUE/DHF - MALAYSIA (PERAK). In: GPHIN, editor. New Straits Times (Malaysia) Fri 11 Sep 1998 ed: PROMED.

81. (1999) DENGUE - BRAZIL (SAO PAULO). In: ProMED-mail, editor. Agencia Estado, Brazil, Wed 28 Apr 1999 ed: PROMED.

82. (1999) DENGUE - BRAZIL (AMAZONAS). In: ProMED-mail, editor. Newspaper A Critica (Manaus), Brazil, 4 Jan 1999 <<http://www.agestado.com.br/links/index.frm>> ed: PROMED.

83. (1999) DENGUE/DHF - HONDURAS (03). In: ProMED-mail, editor. EFE News Services (U.S.) Inc., Fri 02 Jul 1999 [edited] ed: PROMED.

84. (1999) DENGUE ADVISORY - CUBA (HAVANA). In: ProMED-mail, editor. El Nuevo Dia, Puerto Rico, 5? May 1999 [in Spanish] ed: PROMED.

85. (1999) DENGUE/DHF - HONDURAS (05): ALERT. PROMED.

86. (1999) DENGUE - INDIA (NORTH). In: ProMED-mail, editor: PROMED.

87. (2000) DENGUE - VENEZUELA: ALERT. In: ProMED-mail, editor. EFE News Services (U.S.) Inc. Tue 18 Jan 2000 ed: PROMED.

88. (2000) DENGUE/DHF - BANGLADESH (03). Kyodo News, 14 Jul 2000 [edited] ed: PROMED.

89. (2000) DENGUE - SRI LANKA (SOUTH): ALERT. In: ProMED-mail, editor. Australian Broadcasting Corp., Thu 2 Nov 2000 (03:44:34 AEST)[edited] ed: PROMED.

90. (2001) DENGUE/DHF - PANAMA. In: ProMED-mail, editor. La Nacion (Costa Rica), ACAN-EFE, Fri 3 Aug 2001 (trans. by Mod.MPP) [edited] ed: PROMED.

91. (2001) DENGUE/DHF UPDATES (01): 8 JAN 2001 [Costa Rica]. In: ProMED-mail, editor. Xinhua 30 Dec 2000 [edited] ed: PROMED.

92. (2001) DENGUE/DHF UPDATES (08): 31 JUL 2001 [San Salvador/Vietnam]. In: ProMED-mail, editor. La Nacion (San Jose, Costa Rica), AP Report, Tue 24 Jul 2001[edited] ed: PROMED.

93. (2001) DENGUE/DHF UPDATES (05): 27 MAR 2001 [Brazil]. In: ProMED-mail, editor. Folha Online, Brazil, 25 Mar 2001 [edited] ed: PROMED.

94. (2001) DENGUE/DHF - VIETNAM. Agencia EFE (via COMTEX), Fri 25 May 2001 8:58 AM EST [edited] ed: PROMED.

95. (2001) DENGUE/DHF UPDATES (06): 24 MAY 2001 [Peru]. El Comerico (Quito) 8 May 2001 (translated by MPP) [edited] ed: PROMED.

96. (2001) DENGUE/DHF - PANAMA (02). Terra Online-Domingo, 4 de novembro de 2001 [edited] ed: PROMED.

97. (2001) DENGUE/DHF UPDATES (18): 14 NOV 2001 [Nicaragua/Panama]. In: ProMED-mail, editor. Agencia EFE (via Cccomtex), Mon 12 Nov 2001 [edited] ed: PROMED.

98. (2001) DENGUE - INDIA (DELHI). In: ProMED-mail, editor. Rediff.com, Sat 13 Oct 2001 [edited] ed: PROMED.

99. (2001) DENGUE - BRAZIL. Agencia EFE (via COMTEX), Wed 17 Oct 2001 [edited] ed: PROMED.

100. (2001) DENGUE/DHF UPDATES (13): 5 APR 2002 [Australia]. In: ProMED-mail, editor. ABC (Australian Broadcasting Corporation), Sun 31 Mar 2002 [edited] ed: PROMED.

101. (2002) DENGUE/DHF UPDATES (14): 12 APR 2002 [Hawaii]. In: ProMED-mail, editor. Associated Press Newswires, Wed 10 April 2002 [edited] ed: PROMED.

102. (2002) DENGUE/DHF (TYPE 4) - EL SALVADOR. In: ProMED-mail, editor. Agencia EFE (via Comtex), Wed 28 Aug 2002 [edited] ed: PROMED.

103. (2002) DENGUE/DHF - GUATEMALA. In: ProMED-mail, editor. Agencia EFE (via COMTEX), Mon 5 Aug 2002 [edited] ed: PROMED.

104. (2002) DENGUE/DHF UPDATES (31): 12 AUG 2002 [Mexico/Bangladesh/Honduras/Venezuela/Taiwan/Laos]. In: ProMED-mail, editor. Agencia EFE (via COMTEX), Tue 6 Aug 2002 [edited] ed: PROMED.

105. (2002) DENGUE/DHF UPDATES (06): 16 FEB 2002 [Brazil]. In: ProMED-mail, editor. O Globo Online 15 Feb 2002 [in Portuguese] ed: PROMED.

106. (2002) DENGUE/DHF UPDATES (27): 15 JUL 2002 [Malaysia/Taiwan]. In: ProMED-mail, editor. New Straits Times, Sat 13 Jul 2002 [edited] ed: PROMED.

107. (2002) DENGUE/DHF UPDATES (28): 22 JUL 2002 [Mexico / Honduras/ China]. In: ProMED-mail, editor. Zwire, EFE (via COMTEX), Tue 16 Jul 2002 [edited] ed: PROMED.

108. (2002) DENGUE/DHF UPDATES (07): 22 FEB 2002 [Peru]. In: ProMED-mail, editor. EFE News, Mon 18 Feb 2002 [edited] ed: PROMED.

109. (2002) DENGUE/DHF UPDATES (11): 22 MAR 2002 [Malaysia / Hawaii/ Honduras]. In: ProMED-mail, editor. The Star Online, Fri 15 Mar 2002 [edited] ed: PROMED.

110. (2002) DENGUE - CHILE (EASTER ISLAND) (02). Agencia EFE (via COMTEX), Tue 19 Mar 2002 [edited] ed: PROMED.

111. (2002) DENGUE/DHF UPDATES (40): 14 OCT 2002 [Malaysia/ India]. In: ProMED-mail, editor. The Star Online, Wed 9 Oct 2002 [edited] ed: PROMED.

112. (2002) DENGUE/DHF UPDATES (37): 23 SEP 2002 [Hong Kong]. In: ProMED-mail, editor. BBC News Online, Sun 22 Sep 2002 [edited] ed: PROMED.

113. (2003) DENGUE/DHF UPDATE 2003 (13) [New Caledonia]. In: ProMED-mail, editor. Australian Broadcasting Company, News, Thu 27 Mar 2003 [edited] ed: PROMED.

114. (2003) DENGUE/DHF UPDATE 2003 (29) [Indonesia/ China]. In: ProMED-mail, editor. Jakarta Post, Fri 18 Jul 2003 [edited] ed: PROMED.

115. (2003) DENGUE/DHF UPDATE 2003 (26) [Vietnam/ Taiwan]. In: ProMED-mail, editor. People's Daily, Tue 24 Jun 2003 [edited] ed: PROMED.

116. (2003) DENGUE/DHF UPDATE 2003 (10) [Indonesia/ Paraguay/ Australia/ Ecuador]. In: ProMED-mail, editor. The Jakarta Post, Tue 5 Mar 2003 [edited] ed: PROMED.

117. (2003) DENGUE/DHF UPDATE 2003 (43) [India]. In: ProMED-mail, editor. Times of India 6 Nov 2003 [edited] ed: PROMED.

118. (2003) DENGUE/DHF UPDATE 2003 (45) [Bangladesh]. In: ProMED-mail, editor. Shanghai Daily news 17 Nov 2003 [edited] ed: PROMED.

119. (2003) DENGUE/DHF UPDATE 2003 (46) [India]. In: ProMED-mail, editor. Newindpress.com 25 Nov 2003 [edited] ed: PROMED.

120. (2003) DENGUE/DHF UPDATE 2003 (40) [Sri Lanka/ India/ Malaysia]. In: ProMED-mail, editor. Daily News 7 Oct 2003 [edited] ed: PROMED.

121. (2003) DENGUE/DHF UPDATE 2003 (41) [El Salvador/ India]. In: ProMED-mail, editor. Associated Press; [edited & translated by Maria Jacobs, ProMED-ESP] ed: PROMED.

122. (2003) DENGUE/DHF UPDATE 2003 (34) [India/ Venezuela]. In: ProMED-mail, editor. The Hindu 18 Aug 2003 [edited] ed: PROMED.

123. (2003) DENGUE/DHF UPDATE 2003 (37) [India/ Indonesia/ Philippines]. In: ProMED-mail, editor. Times of India 22 Sep 2003 [edited] ed: PROMED.

124. (2004) DENGUE/DHF UPDATE 2004 (25) [Taiwan/ Brazil]. In: ProMED-mail, editor. Taipei Times, 7 Aug 2004 [edited] ed: PROMED.

125. (2004) DENGUE/DHF UPDATE 2004 (05) [Indonesia/ Australia/ Honduras]. In: ProMED-mail, editor. Associated Press, 16 Feb 2004 [edited] ed: PROMED.

126. (2004) DENGUE/DHF UPDATE 2004 (04) [Peru/ Honduras/ Bolivia]. In: ProMED-mail, editor. Xinhua News Agency 29 Jan 2004 [edited] ed: PROMED.

127. (2004) DENGUE/DHF UPDATE 2004 (16) [Vietnam/ Indonesia]. In: ProMED-mail, editor. Xinhuanet, China View 29 May 2004 [edited] ed: PROMED.

128. (2004) DENGUE/DHF UPDATE 2004 (18) [Sri Lanka/ Vietnam]. In: ProMED-mail, editor. ColomboPage News Desk, Sri Lanka Sat 19 Jun 2004 [edited] ed: PROMED.

129. (2004) DENGUE FEVER - AUSTRALIA (NORTHERN TERRITORY): MOSQUITO CONTROL. In: ProMED-mail, editor. ABC News Online 10/03/2004 [edited] ed: PROMED.

130. (2004) DENGUE/DHF UPDATE 2004 (09) [Indonesia/ Venezuela/ El Salvador]. In: ProMED-mail, editor. The Jakarta Post, Samarinda, East Kalimantan 24 Mar 2004 [edited] ed: PROMED.

131. (2004) DENGUE/DHF UPDATE 2004 (14) [Sri Lanka]. In: ProMED-mail, editor. Sunday Observer 9 May 2004 [edited] ed: PROMED.

132. (2004) DENGUE/DHF UPDATE 2004 (27) [Palau/ India/ Brazil]. In: ProMED-mail, editor. Pacnews 26 Aug 2004 [edited] ed: PROMED.

133. (2002) DENGUE/DHF UPDATES (24): 25 JUN 2002 [Philippines/ Vietnam/ Brazil]. In: ProMED-mail, editor. The Sun Star, Mon 17 Jun 2002 [edited] ed: PROMED.

134. (2005) DENGUE/DHF ? SUDAN (KORDOFAN): NOT, REQUEST FOR INFORMATION. PROMED.

135. (2004) DENGUE/DHF UPDATE 2004 (32): CHINA. PROMED.

136. (2004) DENGUE/DHF UPDATE 2004 (33): VIET NAM. PROMED.

137. (2004) DENGUE/DHF UPDATE 2004 (34). PROMED.

138. (2004) DENGUE/DHF UPDATE 2004 (36). PROMED.

139. (2005) DENGUE/DHF UPDATE 2005 (01). PROMED.

140. (2005) DENGUE/DHF UPDATE 2005 (03). PROMED.

141. (2005) DENGUE/DHF UPDATE 2005 (04). PROMED.

142. (2005) DENGUE/DHF UPDATE 2005 (05). PROMED.

143. (2005) DENGUE/DHF UPDATE 2005 (07). PROMED.

144. (2005) DENGUE/DHF UPDATE 2005 (08). PROMED.

145. (2005) DENGUE/DHF UPDATE 2005 (11). PROMED.

146. (2005) DENGUE/DHF UPDATE 2005 (13). PROMED.

147. (2005) DENGUE/DHF UPDATE 2005 (20). PROMED.

148. (2005) DENGUE/DHF UPDATE 2005 (22). PROMED.

149. (2005) DENGUE/DHF UPDATE 2005 (24). PROMED.

150. (2005) DENGUE/DHF UPDATE 2005 (25). PROMED.

151. (2005) DENGUE/DHF UPDATE 2005 (26). PROMED.

152. (2005) DENGUE/DHF UPDATE 2005 (27). PROMED.

153. (2005) DENGUE/DHF UPDATE 2005 (32). PROMED.

154. (2005) DENGUE/DHF UPDATE 2005 (36). PROMED.

155. (1981) Dengue type 4 infections in U.S. travelers to the Caribbean. MMWR Morb Mortal Wkly Rep 30: 249-250.

156. (1982) Imported dengue type 4--Florida. MMWR Morb Mortal Wkly Rep 30: 622-623.

157. (1982) Dengue fever in Puerto Rico - 1981. MMWR Morb Mortal Wkly Rep 31: 103-104.

158. (1983) Leads from the MMWR. Imported dengue fever, United States, 1982. the Journal of the American Medical Association JAMA 249: 1990.

159. (1983) Imported dengue fever--United States, 1982. MMWR Morb Mortal Wkly Rep 32: 145-146.

160. (1983) Dengue--Mexico, El Salvador, Honduras. MMWR Morb Mortal Wkly Rep 32: 586-588.

161. (1984) Leads from the MMWR. Dengue--Mexico, 1983. the Journal of the American Medical Association JAMA 251: 2328.

162. (1985) Leads from the MMWR. Imported dengue fever--United States. the Journal of the American Medical Association JAMA 254: 1143.

163. (1985) Dengue fever in U.S. military personnel--Republic of the Philippines. MMWR Morb Mortal Wkly Rep 34: 495-496, 501-502.

164. (1985) Imported dengue fever--United States, 1984. MMWR Morb Mortal Wkly Rep 34: 488-489.

165. (1986) Aedes albopictus introduction--Texas. MMWR Morb Mortal Wkly Rep 35: 141-142.

166. (1986) Dengue hemorrhagic fever--Puerto Rico. MMWR Morb Mortal Wkly Rep 35: 779-782.

167. (1987) Leads from the MMWR. Imported and indigenous dengue fever--United States, 1986. the Journal of the American Medical Association JAMA 258: 1712-1713.

168. (1987) Imported and indigenous dengue fever--United States, 1986. MMWR Morb Mortal Wkly Rep 36: 551-554.

169. (1987) Leads from the MMWR. Dengue hemorrhagic fever--Puerto Rico. the Journal of the American Medical Association JAMA 257: 441, 448.

170. (1989) Imported dengue--United States, 1987. MMWR Morb Mortal Wkly Rep 38: 463-465.

171. (1989) Update: Aedes albopictus infestation--United States, Mexico. MMWR Morb Mortal Wkly Rep 38: 440, 445-446.

172. (1989) Dengue epidemic--Ecuador, 1988. MMWR Morb Mortal Wkly Rep 38: 419-421.

173. (1990) Imported dengue--United States, 1989. MMWR Morb Mortal Wkly Rep 39: 741-742.

174. (1990) Dengue haemorrhagic fever (DHF). Increase in number of cases in the Americas 1980-1987. Wkly Epidemiol Rec 65: 13-15.

175. (1990) Dengue hemorrhagic fever in Venezuela. Epidemiol Bull 11: 7-9.

176. (1991) Imported dengue, 1990. Wkly Epidemiol Rec 66: 282-283.

177. (1991) Imported dengue--United States, 1990. MMWR Morb Mortal Wkly Rep 40: 519-520.

178. (1991) Dengue epidemic--Peru, 1990. Can Dis Wkly Rep 17: 217-218.

179. (1992) From the Centers for Disease Control. Imported dengue--United States, 1991. the Journal of the American Medical Association JAMA 268: 2000.

180. (1992) Imported dengue--United States, 1991. MMWR Morb Mortal Wkly Rep 41: 725, 731-732.

181. (1994) Dengue fever. Outbreak of classic dengue. Wkly Epidemiol Rec 69: 85-86.

182. (1995) From the Centers for Disease Control and Prevention. Imported dengue--United States, 1993-1994. the Journal of the American Medical Association JAMA 274: 113.

183. (1995) Imported dengue--United States, 1993-1994. MMWR Morb Mortal Wkly Rep 44: 353-356.

184. (1995) From the Centers for Disease Control and Prevention. Dengue fever among U.S. military personnel--Haiti, September-November, 1994. the Journal of the American Medical Association JAMA 273: 14-15.

185. (1995) From the Centers for Disease Control and Prevention. Dengue type 3 infection--Nicaragua and Panama, October-November 1994. the Journal of the American Medical Association JAMA 273: 840-841.

186. (1995) Dengue type 3 infection. Nicaragua and Panama, October-November 1994. Wkly Epidemiol Rec 70: 41-43.

187. (1996) Imported dengue--United States, 1995. MMWR Morb Mortal Wkly Rep 45: 988-991.

188. (1996) From the Centers for Disease Control and Prevention. Dengue fever at the US-Mexico border, 1995-1996. the Journal of the American Medical Association JAMA 276: 1464-1465.

189. (1996) Actuels et futurs aspects entomologiques de la dengue et la dengue hémorragique en Guyane française et dans certains pays voisins. Cayenne, Guyane, 23-24 mai 1995. Actes. Bull Soc Pathol Exot Filiales 89: 83-166.

190. (1997) Dengue in Cuba. July 1997. Epidemiol Bull 18: 7-8.

191. (2000) Imported dengue--Florida, 1997-1998. Can Commun Dis Rep 26: 77-79.

192. (2000) From the Centers for Disease Control and Prevention. Imported dengue--United States, 1997 and 1998. the Journal of the American Medical Association JAMA 283: 1953-1954.

193. (2001) From the Centers for Disease Control and Prevention. Underdiagnosis of dengue--Laredo, Texas, 1999. the Journal of the American Medical Association JAMA 285: 877.

194. (2003) Surveillance for acute insecticide-related illness associated with mosquito-control efforts--nine states, 1999-2002. MMWR Morb Mortal Wkly Rep 52: 629-634.

195. (2005) Travel-associated dengue infections--United States, 2001-2004. MMWR Morb Mortal Wkly Rep 54: 556-558.

196. (2006) Dengue fever and dengue haemorrhagic fever. Nurs J India 97: 246-247.

197. (2006) Dengue & dengue hemorrhagic fever. JNMA journal of the Nepal Medical Association 45: I-II.

198. (2006) Travel-associated dengue--United States, 2005. MMWR Morb Mortal Wkly Rep 55: 700-702.

199. (2007) Dengue hemorrhagic fever--U.S.-Mexico border, 2005. MMWR Morb Mortal Wkly Rep 56: 785-789.

200. (2007) Strict criteria for dengue fever may miss cases. Infections in Medicine 24: 195-196.

201. (2008) [A case of falciparum malaria complicated with dengue fever]. Zhongguo Ji Sheng Chong Xue Yu Ji Sheng Chong Bing Za Zhi 26: 408, 411.

202. (2008) Cases of dengue fever on rise in Vietnam. Chin Med J (Engl) 121: 2464.

203. (2009) Wolbachia Parasite Could Limit Dengue Fever. J Exp Biol 212: I-Ii.

204. (2006) Dengue/DHF update 2006 (25) PROMED: Promed.

205. (2006) Dengue, imported, 2005 - USA PROMED: Promed.

206. (2006) Dengue/DHF update 2006 (29) PROMED: Promed.

207. (2006) Dengue/DHF update 2006 (33). PROMED: Promed.

208. (2009) DENGUE/DHF UPDATE 2009. PROMED.

209. (2009) DENGUE/DHF UPDATE 2009 (43). PROMED.

210. (2009) DENGUE/DHF UPDATE 2009 (42). PROMED.

211. (2009) DENGUE/DHF UPDATE 2009 (41). PROMED.

212. (2009) DENGUE/DHF UPDATE 2009 (40). PROMED.

213. (2009) DENGUE/DHF UPDATE 2009 (39). PROMED.

214. (2009) DENGUE/DHF UPDATE 2009 (38). PROMED.

215. (2009) AEDES ALBOPICTUS - FRANCE: FIRST REPORT. PROMED.

216. (2009) DENGUE/DHF UPDATE 2009 (36). PROMED.

217. (2009) DENGUE/DHF UPDATE 2009 (35). PROMED.

218. (2009) DENGUE/DHF UPDATE 2009 (34). PROMED.

219. (2009) DENGUE/DHF UPDATE 2009 (33). PROMED.

220. (2009) DENGUE/DHF UPDATE 2009 (32). PROMED.

221. (2009) DENGUE/DHF UPDATE 2009 (31. PROMED.

222. (2009) DENGUE/DHF UPDATE 2009 (30). PROMED.

223. (2009) DENGUE/DHF UPDATE 2009 (29). PROMED.

224. (2009) DENGUE/DHF UPDATE 2009 (28). PROMED.

225. (2009) DENGUE/DHF UPDATE 2009 (27). PROMED.

226. (2009) DENGUE/DHF UPDATE 2009 (26). PROMED.

227. (2009) DENGUE/DHF UPDATE 2009 (25). PROMED.

228. (2009) DENGUE/DHF UPDATE 2009 (24). PROMED.

229. (2009) DENGUE/DHF UPDATE 2009 (23). PROMED.

230. (2009) DENGUE/DHF UPDATE 2009 (22). PROMED.

231. (2009) DENGUE/DHF UPDATE 2009 (21). PROMED.

232. (2009) DENGUE/DHF UPDATE 2009 (20). PROMED.

233. (2009) DENGUE/DHF UPDATE 2009 (19). PROMED.

234. (2009) DENGUE/DHF UPDATE 2009 (18). PROMED.

235. (2009) DENGUE/DHF UPDATE 2009 (17). PROMED.

236. (2009) DENGUE/DHF UPDATE 2009 (16). PROMED.

237. (2009) DENGUE/DHF UPDATE 2009 (15). PROMED.

238. (2009) DENGUE/DHF UPDATE 2009 (14). PROMED.

239. (2009) DENGUE/DHF UPDATE 2009 (13). PROMED.

240. (2009) DENGUE/DHF UPDATE 2009 (12). PROMED.

241. (2009) DENGUE/DHF UPDATE 2009 (11). PROMED.

242. (2009) DENGUE/DHF UPDATE 2009 (10). PROMED.

243. (2009) DENGUE/DHF UPDATE 2009 (09). PROMED.

244. (2009) DENGUE/DHF UPDATE 2009 (08). PROMED.

245. (2009) DENGUE/DHF UPDATE 2009 (07). PROMED.

246. (2009) DENGUE/DHF UPDATE 2009 (06). PROMED.

247. (2009) DENGUE/DHF UPDATE 2009 (05). PROMED.

248. (2009) DENGUE/DHF UPDATE 2009 (04). PROMED.

249. (2009) DENGUE/DHF UPDATE 2009 (03). PROMED.

250. (2009) DENGUE/DHF UPDATE 2009 (02). PROMED.

251. (2009) DENGUE/DHF UPDATE 2009 (01). PROMED.

252. (2008) DENGUE/DHF UPDATE 2008 (56): SINGAPORE, AUSTRALIA. PROMED.

253. (2008) DENGUE/DHF UPDATE 2008 (55). PROMED.

254. (2008) DENGUE/DHF UPDATE 2008 (54). PROMED.

255. (2008) DENGUE/DHF UPDATE 2008 (53): CAMBODIA, FRANCE ex MALI. PROMED.

256. (2008) DENGUE/DHF UPDATE 2008 (52). PROMED.

257. (2008) DENGUE/DHF UPDATE 2008 (51). PROMED.

258. (2008) DENGUE/DHF UPDATE 2008 (50). PROMED.

259. (2008) DENGUE/DHF UPDATE 2008 (48). PROMED.

260. (2008) DENGUE/DHF UPDATE 2008 (47). PROMED.

261. (2008) DENGUE/DHF UPDATE 2008 (46). PROMED.

262. (2008) DENGUE/DHF UPDATE 2008 (45). PROMED.

263. (2008) DENGUE/DHF UPDATE 2008 (44). PROMED.

264. (2008) DENGUE/DHF UPDATE 2008 (43). PROMED.

265. (2008) DENGUE/DHF UPDATE 2008 (42). PROMED.

266. (2008) DENGUE/DHF UPDATE 2008 (41). PROMED.

267. (2008) DENGUE/DHF UPDATE 2008 (40). PROMED.

268. (2008) DENGUE/DHF UPDATE 2008 (39). PROMED.

269. (2008) DENGUE/DHF UPDATE 2008 (38). PROMED.

270. (2008) DENGUE/DHF UPDATE 2008 (37). PROMED.

271. (2008) DENGUE/DHF UPDATE 2008 (34): PERU, BRAZIL. PROMED.

272. (2008) DENGUE/DHF UPDATE 2008 (33): THAILAND, LAOS, AMERICAN SAMOA. PROMED.

273. (2008) DENGUE/DHF UPDATE 2008 (31). PROMED.

274. (2008) DENGUE/DHF UPDATE 2008 (30): BRAZIL. PROMED.

275. (2008) DENGUE/DHF UPDATE 2008 (29). PROMED.

276. (2008) DENGUE/DHF UPDATE 2008 (28). PROMED.

277. (2008) DENGUE/DHF UPDATE 2008 (27). PROMED.

278. (2008) DENGUE/DHF UPDATE 2008 (26). PROMED.

279. (2008) DENGUE/DHF UPDATE 2008 (25). PROMED.

280. (2008) DENGUE/DHF UPDATE 2008 (24). PROMED.

281. (2008) DENGUE/DHF UPDATE 2008 (23). PROMED.

282. (2008) DENGUE/DHF UPDATE 2008 (22). PROMED.

283. (2008) DENGUE/DHF UPDATE 2008 (21). PROMED.

284. (2008) DENGUE/DHF UPDATE 2008 (20). PROMED.

285. (2008) DENGUE/DHF UPDATE 2008 (19). PROMED.

286. (2008) DENGUE/DHF UPDATE 2008 (18). PROMED.

287. (2008) DENGUE/DHF UPDATE 2008 (17). PROMED.

288. (2008) DENGUE/DHF UPDATE 2008 (16). PROMED.

289. (2008) DENGUE/DHF UPDATE 2008 (15). PROMED.

290. (2008) DENGUE/DHF UPDATE 2008 (14). PROMED.

291. (2008) DENGUE/DHF UPDATE 2008 (13). PROMED.

292. (2008) DENGUE/DHF UPDATE 2008 (12). PROMED.

293. (2008) DENGUE/DHF UPDATE 2008 (11). PROMED.

294. (2008) DENGUE/DHF UPDATE 2008 (10). PROMED.

295. (2008) DENGUE/DHF UPDATE 2008 (09). PROMED.

296. (2008) DENGUE/DHF UPDATE 2008 (08). PROMED.

297. (2008) DENGUE/DHF UPDATE 2008 (07). PROMED.

298. (2008) DENGUE/DHF UPDATE 2008 (06). PROMED.

299. (2008) DENGUE/DHF UPDATE 2008 (05). PROMED.

300. (2008) DENGUE/DHF UPDATE 2008 (04). PROMED.

301. (2008) DENGUE/DHF UPDATE 2008 (03). PROMED.

302. (2008) DENGUE/DHF UPDATE 2008 (02). PROMED.

303. (2008) DENGUE/DHF UPDATE 2008 (01): BRAZIL. PROMED.

304. (2007) DENGUE/DHF UPDATE 2007 (50). PROMED.

305. (2007) DENGUE/DHF UPDATE 2007 (49). PROMED.

306. (2007) DENGUE/DHF UPDATE 2007 (48). PROMED.

307. (2007) DENGUE/DHF UPDATE 2007 (47). PROMED.

308. (2007) DENGUE/DHF UPDATE 2007 (46). PROMED.

309. (2007) DENGUE/DHF UPDATE 2007 (45). PROMED.

310. (2007) DENGUE/DHF UPDATE 2007 (43). PROMED.

311. (2007) DENGUE/DHF UPDATE 2007 (42). PROMED.

312. (2007) DENGUE/DHF UPDATE 2007 (41). PROMED.

313. (2007) DENGUE/DHF UPDATE 2007 (40). PROMED.

314. (2007) DENGUE/DHF UPDATE 2007 (39). PROMED.

315. (2007) DENGUE/DHF UPDATE 2007 (38). PROMED.

316. (2007) DENGUE/DHF UPDATE 2007 (37). PROMED.

317. (2007) DENGUE/DHF UPDATE 2007 (36). PROMED.

318. (2007) DENGUE/DHF UPDATE 2007 (35). PROMED.

319. (2007) DENGUE/DHF UPDATE 2007 (34). PROMED.

320. (2007) DENGUE/DHF UPDATE 2007 (33). PROMED.

321. (2007) DENGUE/DHF UPDATE 2007 (32). PROMED.

322. (2007) DENGUE/DHF UPDATE 2007 (31). PROMED.

323. (2007) DENGUE/DHF UPDATE 2007 (30). PROMED.

324. (2007) DENGUE/DHF UPDATE 2007 (29). PROMED.

325. (2007) DENGUE/DHF UPDATE 2007 (28). PROMED.

326. (2007) DENGUE/DHF UPDATE 2007 (27). PROMED.

327. (2007) DENGUE/DHF UPDATE 2007 (26). PROMED.

328. (2007) DENGUE/DHF UPDATE 2007 (25). PROMED.

329. (2007) DENGUE/DHF UPDATE 2007 (24). PROMED.

330. (2007) DENGUE/DHF UPDATE 2007 (23). PROMED.

331. (2007) DENGUE/DHF UPDATE 2007 (22). PROMED.

332. (2007) DENGUE/DHF UPDATE 2007 (21). PROMED.

333. (2007) DENGUE/DHF UPDATE 2007 (20). PROMED.

334. (2007) DENGUE/DHF UPDATE 2007 (19). PROMED.

335. (2007) DENGUE/DHF UPDATE 2007 (18). PROMED.

336. (2007) DENGUE/DHF UPDATE 2007 (16). PROMED.

337. (2007) DENGUE/DHF UPDATE 2007 (15). PROMED.

338. (2007) DENGUE/DHF UPDATE 2007 (14). PROMED.

339. (2004) Dengue/DHF Update 2004 (32). PROMED.

340. (2004) DENGUE/DHF UPDATE 2004 (34). PROMED. pp. DE_4521.

341. (2005) DENGUE/DHF UPDATE 2005 (22). PROMED. pp. DE_4566.

342. Ansart S, Pajot O, Grivois JP, Zeller V, Klement E, et al. (2004) Pneumonia among travelers returning from abroad. J Travel Med 11: 87-91.

343. Ansart S, Perez L, Vergely O, Danis M, Bricaire F, et al. (2005) Illnesses in travelers returning from the tropics: A prospective study of 622 patients. J Travel Med 12: 312-318.

344. Antinori S, Galimberti L, Gianelli E, Calattini S, Piazza M, et al. (2004) Prospective observational study of fever in hospitalized returning travelers and migrants from tropical areas, 1997-2001. J Travel Med 11: 135-142.

345. A-Nuegoonpipat A, Berlioz-Arthaud A, Chow V, Endy T, Lowry K, et al. (2004) Sustained transmission of dengue virus type 1 in the Pacific due to repeated introductions of different Asian strains. Virology 329: 505-512.

346. Anuradha S, Singh NP, Rizvi SN, Agarwal SK, Gur R, et al. (1998) The 1996 outbreak of dengue hemorrhagic fever in Delhi, India. Southeast Asian J Trop Med Public Health 29: 503-506.

347. Anzai S, Fukuda M, Otsuka Y, Eshita Y (2004) Nucleotide sequence and phylogenetic analyses of dengue type 2 virus isolated in the Dominican Republic. Virus Genes 29: 219-227.

348. Aoki C, Hidari KI, Itonori S, Yamada A, Takahashi N, et al. (2006) Identification and characterization of carbohydrate molecules in Mammalian cells recognized by dengue virus type 2. J Biochem (Tokyo) 139: 607-614.

349. Appanna R, Huat TL, See LLC, Tan PL, Vadivelu J, et al. (2007) Cross-reactive T-Cell responses to the nonstructural regions of dengue viruses among dengue fever and dengue hemorrhagic fever patients in Malaysia. Clin Vaccine Immunol 14: 969-977.

350. Appawu M, Dadzie S, Abdul H, Asmah H, Boakye D, et al. (2006) Surveillance of viral haemorrhagic fevers in ghana: entomological assessment of the risk of transmission in the northern regions. Ghana Med J 40: 137-141.

351. Apt D, Raviprakash K, Brinkman A, Semyonov A, Yang SM, et al. (2006) Tetravalent neutralizing antibody response against four dengue serotypes by a single chimeric dengue envelope antigen. Vaccine 24: 335-344.

352. Aquino JDJD, Tang WF, Ishii R, Ono T, Eshita Y, et al. (2008) Molecular epidemiology of dengue virus serotypes 2 and 3 in Paraguay during 2001-2006: The association of viral clade introductions with shifting serotype dominance. Virus Res 137: 266-270.

353. Araujo FMD, Nogueira RMR, de Araujo JMG, Ramalho ILC, Roriz MLFD, et al. (2006) Concurrent infection with dengue virus type-2 and DENV-3 in a patient from Ceara, Brazil. Mem Inst Oswaldo Cruz 101: 925-928.

354. Arboleda M, Campuzano M, Restrepo BN, Cartagena G (2006) [El comportamiento clínico del dengue en pacientes hospitalizados en el hospital Antonio Roldán Betancur de Apartadó, Antioquia, 2000]. Biomedica 26: 286-294.

355. Arguelles D, Marfil JO, Isip R (1998) DENGUE/DHF - PHILIPPINES (05). Manila Standard Fri 25 Sep 1998 ed: PROMED.

356. Armien B, Suaya JA, Quiroz E, Sah BK, Bayard V, et al. (2008) Clinical characteristics and national economic cost of the 2005 dengue epidemic in Panama. Am J Trop Med Hyg 79: 364-371.

357. Arredondo LB (2005) DENGUE/DHF UPDATE 2005 (07). PROMED.

358. Arunachalam N, Murty US, Kabilan L, Balasubramanian A, Thenmozhi V, et al. (2004) Studies on dengue in rural areas of Kurnool District, Andhra Pradesh, India. J Am Mosq Control Assoc 20: 87-90.

359. Arya SC, Agarwal N (2007) Dengue and dengue hemorrhagic fever. Aust Fam Physician 36: 295-295.

360. Arya SC, Agarwal N (2009) Dengue and concurrent urinary infection in a tertiary care hospital in Delhi. Trans R Soc Trop Med Hyg 103: 642-643.

361. Arya SC, Rajagopal S, Agarwal N, Kaushik M, Maheshwari P, et al. (2004) Private sector hospital response to the 2003 dengue outbreak in the Indian capital metropolis of Delhi. Am J Infect Control 32: 489-492.

362. Asato L (2001) DENGUE - USA (HAWAII) (05). In: ProMED-mail, editor. The Star Bulletin, Sat 13 Oct 2001 [edited] ed: PROMED.

363. Ascher DP, Laws HF, Hayes CG (1989) The use of intravenous gammaglobulin in dengue fever, a case report. Southeast Asian J Trop Med Public Health 20: 549-554.

364. Ashford DA, Savage HM, Hajjeh RA, McReady J, Bartholomew DM, et al. (2003) Outbreak of dengue fever in Palau, Western Pacific: risk factors for infection. Am J Trop Med Hyg 69: 135-140.

365. Atias D, Liebes Y, Chalifa-Caspi V, Bremand L, Lobel L, et al. (2009) Chemiluminescent optical fiber immunosensor for the detection of IgM antibody to dengue virus in humans. Sensors and Actuators B-Chemical 140: 206-215.

366. Attatippaholkun WH, Attatippaholkun MK, Nisalak A, Vaughn DW, Innis BL (1998) Nucleotide sequence and deduced amino acid sequence of the nonstructural proteins of dengue type 3 virus, Bangkok genotype. Southeast Asian J Trop Med Public Health 29: 361-366.

367. Attatippaholkun WH, Attatippaholkun MK, Nisalak A, Vaughn DW, Innis BL (2000) Highly conserved nucleotide sequence and its deduced amino acids of the 5'-noncoding region and the capsid protein of a Bangkok isolate dengue-3 virus. Southeast Asian J Trop Med Public Health 31 Suppl 1: 119-125.

368. Au WY, Ma ES, Kwong YL (2001) Acute myeloid leukemia precipitated by dengue virus infection in a patient with hemoglobin H disease. Haematologica 86: e17.

369. Auyeung TW, Que TL, Lam KS, Ng HL, Szeto ML (2003) The first patient with locally acquired dengue fever in Hong Kong. Hong Kong Med J 9: 127-129.

370. Avila Montes GA, Martinez M, Sherman C, Fernandez Cerna E (2004) El comportamiento clínico del dengue en pacientes hospitalizados en el hospital Antonio Roldán Betancur de Apartadó, Antioquia, 2000. Rev Panam Salud Publica 16: 84-94.

371. Avila-Aguero ML, Avila-Aguero CR, Um SL, Soriano-Fallas A, Canas-Coto A, et al. (2004) Systemic host inflammatory and coagulation response in the Dengue virus primo-infection. Cytokine 27: 173-179.

372. Aviles G, Meissner J, Mantovani R, St Jeor S (2003) Complete coding sequences of dengue-1 viruses from Paraguay and Argentina. Virus Res 98: 75-82.

373. Aviles G, Paz MV, Rangeon G, Ranaivoarisoa MY, Verzeri N, et al. (2003) Laboratory surveillance of dengue in Argentina, 1995-2001. Emerging Infectious Dieseases 9: 738-742.

374. Aviles G, Rangeon G, Baroni P, Paz V, Monteros M, et al. (2000) Brote de virus dengue-2 en Salta, Argentina, 1988. Medicina (Mex) 60: 875-879.

375. Aviles G, Rangeon G, Paz MV, Baroni P, Sabattini MS, et al. (2001) Secundarios respuestas serológicas a la epidemia de dengue en 1998 en Salta, Argentina, donde otros flavivirus co-circulan. Medicina (Mex) 61: 129-136.

376. Aviles G, Rangeon G, Vorndam V, Briones A, Baroni P, et al. (1999) Dengue reemergence in Argentina. Emerg Infect Dis 5: 575-578.

377. Aviles G, Rowe J, Meissner J, Manzur Caffarena JC, Enria D, et al. (2002) Phylogenetic relationships of dengue-1 viruses from Argentina and Paraguay. Arch Virol 147: 2075-2087.

378. Avirutnan P, Punyadee N, Noisakran S, Komoltri C, Thiemmeca S, et al. (2006) Vascular leakage in severe dengue virus infections: A potential role for the nonstructural viral protein NS1 and complement. J Infect Dis 193: 1078-1088.

379. Axelsen PH (1996) DENGUE, SUSPECTED - BRITISH VIRGIN ISLANDS. In: University of Pennsylvania DoP, editor. Philadelphia: PROMED.

380. Ayres CF, Melo-Santos MA, Sole-Cava AM, Furtado AF (2003) Genetic differentiation of Aedes aegypti (Diptera: Culicidae), the major dengue vector in Brazil. J Med Entomol 40: 430-435.

381. Ayyub M, Khazindar AM, Lubbad EH, Barlas S, Alfi AY, et al. (2006) Characteristics of dengue fever in a large public hospital, Jeddah, Saudi Arabia. Journal of Ayub Medical College, Abbottabad 18: 9-13.

382. Azeredo EL, De Oliveira-Pinto LM, Zagne SM, Cerqueira DIS, Nogueira RMR, et al. (2006) NK cells, displaying early activation, cytotoxicity and adhesion molecules, are associated with mild dengue disease. Clin Exp Immunol 143: 345-356.

383. Azeredo EL, Zagne SM, Alvarenga AR, Nogueira RMR, Kubelka CF, et al. (2006) Activated peripheral lymphocytes with increased expression of cell adhesion molecules and cytotoxic markers are associated with dengue fever disease. Mem Inst Oswaldo Cruz 101: 437-449.

384. Azevedo LS, Carvalho DBM, Matuck T, Alvarenga MF, Morgado L, et al. (2007) Dengue in renal transplant patients: A retrospective analysis. Transplantation 84: 792-794.

385. Aziz MM, Hasan KN, Hasanat MA, Siddiqui MA, Salimullah M, et al. (2002) Predominance of the DEN-3 genotype during the recent dengue outbreak in Bangladesh. Southeast Asian J Trop Med Public Health 33: 42-48.

386. Bacsal KE, Chee SP, Cheng CL, Flores JVP (2007) Dengue-associated maculopathy. Arch Ophthalmol 125: 501-510.

387. Badiaga S, Barrau K, Brouqui P, Durant J, Malvy D, et al. (2003) Imported Dengue in French University Hospitals: a 6-year survey. J Travel Med 10: 286-289.

388. Bai ZJ, Liu LC, Tu Z, Yao LS, Liu JW, et al. (2008) Real-time PCR for detecting circulating dengue virus in the Guangdong Province of China in 2006. J Med Microbiol 57: 1547-1552.

389. Bakker RC, Veenstra J, Dingemans-Dumas AM, Wetsteyn J, Kager PA (1996) Imported Dengue in The Netherlands. J Travel Med 3: 204-208.

390. Balasubramanian S, Janakiraman L, Kumar SS, Muralinath S, Shivbalan S (2006) A reappraisal of the criteria to diagnose plasma leakage in dengue hemorrhagic fever. Indian Pediatr 43: 334-339.

391. Balmaseda A, Guzman MG, Hammond S, Robleto G, Flores C, et al. (2003) Diagnosis of dengue virus infection by detection of specific immunoglobulin M (IgM) and IgA antibodies in serum and saliva. Clin Diagn Lab Immunol 10: 317-322.

392. Balmaseda A, Hammond SN, Perez L, Tellez Y, Saborio SI, et al. (2006) Serotype-specific differences in clinical manifestations of dengue. Am J Trop Med Hyg 74: 449-456.

393. Balmaseda A, Hammond SN, Perez MA, Cuadra R, Solano S, et al. (2005) Short report: Assessment of the World Health Organization scheme for classification of dengue severity in Nicaragua. Am J Trop Med Hyg 73: 1059-1062.

394. Balmaseda A, Sandoval E, Perez L, Gutierrez CM, Harris E (1999) Application of molecular typing techniques in the 1998 dengue epidemic in Nicaragua. Am J Trop Med Hyg 61: 893-897.

395. Bancroft WH (1987) Current status of dengue vaccines and prospects for the future. P R Health Sci J 6: 23-26.

396. Bandyopadhyay S, Lum LCS, Kroeger A (2006) Classifying dengue: a review of the difficulties in using the WHO case classification for dengue haemorrhagic fever. Trop Med Int Health 11: 1238-1255.

397. Banks A (2002) DENGUE/DHF UPDATES (49): 23 DEC 2002 [Malaysia]. New Straits Times (Malaysia) online, Tue 17 Dec 2002 [edited] ed: PROMED.

398. Banks A (2002) DENGUE/DHF UPDATES (39): 7 OCT 2002 [Bangladesh/Hong kong]. In: ProMED-mail, editor. The Independent (Bangladesh), Wed 2 Oct 2002 [edited] ed: PROMED.

399. Banks A (2002) DENGUE/DHF UPDATES (34): 1 SEP 2002 [Bangladesh/Nicaragua/Cuba/ Mexico]. In: ProMED-mail, editor. The Daily Star, Tue 27 Aug 2002 [edited] ed: PROMED.

400. Banks A (2002) DENGUE/DHF UPDATES (35): 8 SEP 2002 [Bangladesh/ Mexico]. In: ProMED-mail, editor. The Daily Star (Bangladesh) 2 Sep 2002 [edited] ed: PROMED.

401. Banks A (2003) DENGUE/DHF UPDATE 2003 (33) [Philippines/ China/ Brazil]. In: ProMED-mail, editor. The Sun Star, Wed 13 Aug 2003 [edited] ed: PROMED.

402. Banks A (2003) DENGUE/DHF UPDATE 2003 (08) [India/Thailand/ Mexico]. In: ProMED-mail, editor. Keralanext online, Sat Feb 2003 [edited] ed: PROMED.

403. Banks A (2003) DENGUE/DHF UPDATE 2003 (02) [Malaysia/ Fiji]. In: ProMED-mail, editor. Malay Mail (Malaysia), Tue 7 Jan 2003 [edited] ed: PROMED.

404. Banks A (2003) DENGUE/DHF UPDATE 2003 (03) [India/ Sri Lanka/ Malaysia]. In: ProMED-mail, editor. The Hindu online, Sat 11 Jan 2003 [edited] ed: PROMED.

405. Banks A (2003) DENGUE/DHF UPDATE 2003 (30) [India]. In: ProMED-mail, editor. The Hindu, Tue 22 Jul 2003 [edited] ed: PROMED.

406. Banks A (2003) DENGUE/DHF UPDATE 2003 (22) [Philippines/ Singapore/ India/Brazil]. In: ProMED-mail, editor. Minda News online, Sat 24 May 2003 [edited] ed: PROMED.

407. Banks A (2003) DENGUE/DHF UPDATE 2003 (23) [India]. In: ProMED-mail, editor. The Hindu, Wed 4 Jun 2003 [edited] ed: PROMED.

408. Banks A (2003) DENGUE/DHF UPDATE 2003 (24) [Philippines/ Brazil]. In: ProMED-mail, editor. Sun Star online, Tue 10 Jun 2003 [edited] ed: PROMED.

409. Banks A (2005) DENGUE/DHF UPDATE 2005 (10). PROMED.

410. Banks A (2005) DENGUE/DHF UPDATE 2005 (11). PROMED.

411. Banks A (2005) DENGUE/DHF UPDATE 2005 (12). PROMED.

412. Banks A (2005) DENGUE/DHF UPDATE 2005 (13). PROMED.

413. Banks A (2005) DENGUE/DHF UPDATE 2005 (14). PROMED.

414. Banks A (2005) DENGUE/DHF UPDATE 2005 (15). PROMED.

415. Banks A (2005) DENGUE/DHF UPDATE 2005 (16). PROMED.

416. Banks A (2005) DENGUE/DHF UPDATE 2005 (17). PROMED.

417. Banks A (2005) DENGUE/DHF UPDATE 2005 (18). PROMED.

418. Banks A (2005) DENGUE/DHF UPDATE 2005 (19). PROMED.

419. Banks A (2005) DENGUE/DHF UPDATE 2005 (21). PROMED.

420. Banks A (2005) DENGUE/DHF UPDATE 2005 (23). PROMED.

421. Banks A (2005) DENGUE/DHF UPDATE 2005 (24). PROMED.

422. Banks A (2005) DENGUE/DHF UPDATE 2005 (25). PROMED.

423. Banks A (2005) DENGUE/DHF UPDATE 2005 (26). PROMED.

424. Banks A (2005) DENGUE/DHF UPDATE 2005 (27). PROMED.

425. Banks A (2005) DENGUE/DHF UPDATE 2005 (29). PROMED.

426. Banks A (2005) DENGUE/DHF UPDATE 2005 (30). PROMED.

427. Banks A (2005) DENGUE/DHF UPDATE 2005 (34). PROMED.

428. Banks A (2005) DENGUE/DHF UPDATE 2005 (35). PROMED.

429. Banks A (2005) DENGUE/DHF UPDATE 2005 (37). PROMED.

430. Banks A (2005) DENGUE/DHF UPDATE 2005 (38). PROMED.

431. Banks A (2005) DENGUE/DHF UPDATE 2005 (39). PROMED.

432. Banks A (2006) DENGUE/DHF UPDATE 2006 (01). PROMED.

433. Banks A (2006) DENGUE/DHF UPDATE 2006 (03). PROMED.

434. Banks A (2006) DENGUE/DHF UPDATE 2006 (05). PROMED.

435. Banks A (2006) DENGUE/DHF UPDATE 2006 (07). PROMED.

436. Banks A (2006) DENGUE/DHF UPDATE 2006 (08). PROMED.

437. Banks A (2007) DENGUE/DHF UPDATE 2007 (17). PROMED.

438. Banks A (2007) Dengue/DHF Update 2007 (04). ProMED.

439. Banks A (2007) Dengue/DHF update 2007 (06). PROMED.

440. Banks A (2005) DENGUE/DHF UPDATE 2005 (24). PROMED. pp. DE_4570.

441. Banks A-L (2006) Dengue/DHF update 2006 (24). PROMED: Promed.

442. Banks A-L (2006) Dengue/DHF update 2006 (30) PROMED.

443. Banks A-L (2006) Dengue/DHF update 2006 (39) PROMED: Promed.

444. Banks A-L (2007) Dengue/DHF update 2007 PROMED: Promed.

445. Banks A-L (2007) Dengue/DHF update 2007 (07) PROMED: Promed.

446. Banks A-L (2007) Dengue/DHF update 2007 (09) PROMED: Promed.

447. Banks A-L (2007) Dengue/DHF update 2007 (15) PROMED: Promed.

448. Banks AL (2006) DENGUE/DHF UPDATE 2006 (09). PROMED.

449. Banks AL (2006) DENGUE/DHF UPDATE 2006 (11). PROMED.

450. Banks AL (2006) DENGUE/DHF UPDATE 2006 (12). PROMED.

451. Banks AL (2006) DENGUE/DHF UPDATE 2006 (14). PROMED.

452. Banks A-L, Barrett B, Hopp M (2007) Dengue/DHF update 2007 (08) PROMED: Promed.

453. Banks A-L, Bryant Ka (2007) Dengue/DHF update 2007 (16) PROMED: Promed.

454. Banks A-L, Chiu C-H (2006) Dengue/DHF update 2006 (31) PROMED: Promed.

455. Banks A-L, Dudley J (2007) Dengue/DHF update 2007 (06). PROMED: Promed.

456. Banks A-L, Dudley J, Duffy D (2006) Dengue/DHF update 2006 (32) PROMED: Promed.

457. Banks A-L, Marshall M, Silver D (2007) Dengue/DHF Update 2007 (04) PROMED: Promed.

458. Banks A-L, Rodriguez A (2006) Dengue/DHF update 2006 (21) PROMED.

459. Banks A-L, Roesel T (2006) Dengue/DHF update 2006 (22) PROMED: Promed.

460. Banks A-L, Schmidt T (2007) Dengue/DHF update 2007 (02) PROMED: Promed.

461. Bannatyne S (2005) DENGUE/DHF UPDATE 2005 (16). PROMED.

462. Bannatyne S (2005) DENGUE/DHF UPDATE 2005 (27). PROMED.

463. Bannerjee K (1998) DENGUE - INDIA (MAHARASHTRA). In: ProMED-mail, editor: PROMED.

464. Bannerjee K (1998) DENGUE - INDIA (MAHARASHTRA) (04). PROMED.

465. Barata EA, Costa AI, Chiaravalloti Neto F, Glasser CM, Barata JM, et al. (2001) População de Aedes aegypti (l.) em área endêmica de dengue, Sudeste do Brasil. Rev Saude Publica 35: 237-242.

466. Barbazan P, Yoksan S, Gonzalez JP (2002) Dengue hemorrhagic fever epidemiology in Thailand: description and forecasting of epidemics. Microbes Infect 4: 699-705.

467. Barcellos C, Pustai AK, Weber MA, Brito MRV (2005) Identificação de locais com potencial de transmissão de dengue em Porto Alegre através de técnicas de geoprocessamento. Rev Soc Bras Med Trop 38: 246-250.

468. Barcelos Figueiredo L, Batista Cecilio A, Portela Ferreira G, Paiva Drumond B, Germano de Oliveira J, et al. (2008) Dengue virus 3 genotype 1 associated with dengue fever and dengue hemorrhagic fever, Brazil. Emerg Infect Dis 14: 314-316.

469. Barkham TM, Chung YK, Tang KF, Ooi EE (2006) The performance of RT-PCR compared with a rapid serological assay for acute dengue fever in a diagnostic laboratory. Trans R Soc Trop Med Hyg 100: 142-148.

470. Barrera R, Delgado N, Jimenez M, Villalobos I, Romero I (2000) Estratificación de una ciudad hiperendémica en dengue hemorrágico. Rev Panam Salud Publica 8: 225-233.

471. Barreto FR, Teixeira MG, Costa MDN, Carvalho MS, Barreto ML (2008) Spread pattern of the first dengue epidemic in the city of Salvador, Brazil. BMC Public Health 8: -.

472. Barreto ML, Teixeira MG (2008) Dengue fever: a call for local, national, and international action. Lancet 372: 205-205.

473. Barrett B (2007) Dengue/DHF update 2007 (03) PROMED: Promed.

474. Barrett B (2007) Dengue/DHF update 2007 (03). PROMED. pp. DE_15336.

475. Barrett P (2000) DENGUE/DHF: UPDATES, 15 OCT 2000 [Costa Rica]. In: ProMED-mail, editor. Medical Advisory Service for Travellers Abroad, 2 Oct 2000 15:09:29 +0100 ed: PROMED.

476. Barth OM (1992) Replication of dengue viruses in mosquito cell cultures--a model from ultrastructural observations. Mem Inst Oswaldo Cruz 87: 565-574.

477. Barth OM, Cortes LM, Lampe E, Farias Filho JC (1994) Ultrastructural aspects of virus replication in one fatal case and several other isolates from a dengue type 2 outbreak in Rio de Janeiro. Mem Inst Oswaldo Cruz 89: 21-24.

478. Bartholomew DLT (1996) DENGUE - PALAU ISLANDS (3). PROMED.

479. Bartley LM, Donnelly CA, Garnett GP (2002) The seasonal pattern of dengue in endemic areas: mathematical models of mechanisms. Trans R Soc Trop Med Hyg 96: 387-397.

480. Baruah J, Ananda S, Arun Kumar G (2006) Incidence of dengue in a tertiary care centre--Kasturba Hospital, Manipal. Indian J Pathol Microbiol 49: 462-463.

481. Bashyam HS, Green S, Rothman AL (2006) Dengue virus-reactive CD8+ T cells display quantitative and qualitative differences in their response to variant epitopes of heterologous viral serotypes. J Immunol 176: 2817-2824.

482. Basilio-de-Oliveira CA, Aguiar GR, Baldanza MS, Barth OM, Eyer-Silva WA, et al. (2005) Pathologic study of a fatal case of dengue-3 virus infection in Rio de Janeiro, Brazil. Braz J Infect Dis 9: 341-347.

483. Bassi C, Landry P, Malinverni R (2003) La fièvre au retour d'un voyage: rapport de 3 cas de fièvre dengue. Rev Med Suisse Romande 123: 219-221.

484. Basu M, Dasgupta MK, Kundu TK, Sengupta B, De GK, et al. (2007) Profile of pediatric dengue cases from a tertiary care hospital in Kolkata. Indian J Public Health 51: 234-236.

485. Basurko C, Carles G, Youssef M, Guindi WE (2009) Maternal and foetal consequences of dengue fever during pregnancy. Eur J Obstet Gynecol Reprod Biol.

486. Bayard V (1998) DENGUE - PANAMA. In: ProMED-mail, <http://www.healthnet.org/programs/promed.html>, editors. (Panama): PROMED.

487. Bayard V (1998) DENGUE - PANAMA (02). In: ProMED-mail, editor: PROMED.

488. Bayard V, Quiroz E, Mojica J (1996) Re-emergencia del dengue en Panamá. Rev Med Panama 21: 85-92.

489. Beatty ME, Hunsperger E, Long E, Schurch J, Jain S, et al. (2007) Mosquitoborne infections after Hurricane Jeanne, Haiti, 2004. Emerg Infect Dis 13: 308-310.

490. Becerra A, Warke RV, de Bosch N, Rothman AL, Bosch I (2008) Elevated levels of soluble ST2 protein in dengue virus infected patients. Cytokine 41: 114-120.

491. Becerra A, Warke RV, Xhaja K, Evans B, Evans J, et al. (2009) Increased activity of indoleamine 2,3-dioxygenase in serum from acutely infected dengue patients linked to gamma interferon antiviral function. J Gen Virol 90: 810-817.

492. Beckett CG, Kosasih H, Faisal I, Nurhayati, Tan R, et al. (2005) Early detection of dengue infections using cluster sampling around index cases. Am J Trop Med Hyg 72: 777-782.

493. Bedouelle H, Belkadi L, England P, Guijarro JI, Lisova O, et al. (2006) Diversity and junction residues as hotspots of binding energy in an antibody neutralizing the dengue virus. FEBS J 273: 34-46.

494. Ben RJ, Lo CH, Yu HL, Hsueh CW, Feng NH, et al. (2008) The Features of Clinical Manifestations of Dengue Fever in Kaohsiung City, Taiwan, 2006. Int J Infect Dis 12: E93-E94.

495. Benarroch D, Egloff MP, Mulard L, Guerreiro C, Romette JL, et al. (2004) A structural basis for the inhibition of the NS5 dengue virus mRNA 2 '-O-Methyltransferase domain by ribavirin 5 '-triphosphate. J Biol Chem 279: 35638-35643.

496. Benarroch D, Selisko B, Locatelli GA, Maga G, Romette JL, et al. (2004) The RNA helicase, nucleotide 5 '-triphosphatase, and RNA 5 '-triphosphatase activities of Dengue virus protein NS3 are Mg2+-dependent and require a functional Walker B motif in the helicase catalytic core. Virology 328: 208-218.

497. Bennett KE, Beaty BJ, Black WC (2005) Selection of D2S3, an Aedes aegypti (Diptera : Culicidae) strain with high oral susceptibility to Dengue 2 virus and D2MEB, a strain with a midgut barrier to Dengue 2 escape. J Med Entomol 42: 110-119.

498. Bennett KE, Flick D, Fleming KH, Jochim R, Beaty BJ, et al. (2005) Quantitative trait loci that control dengue-2 virus dissemination in the mosquito Aedes aegypti. Genetics 170: 185-194.

499. Bennett KE, Olson KE, Munoz Mde L, Fernandez-Salas I, Farfan-Ale JA, et al. (2002) Variation in vector competence for dengue 2 virus among 24 collections of Aedes aegypti from Mexico and the United States. Am J Trop Med Hyg 67: 85-92.

500. Bennett SN, Holmes EC, Chirivella M, Rodriguez DM, Beltran M, et al. (2003) Selection-driven evolution of emergent dengue virus. Mol Biol Evol 20: 1650-1658.

501. Beral L, Merle H, David T (2009) Dengue Optic Neuropathy Reply. Ophthalmology 116: 170-170.

502. Berard H, Laille M (1990) 40 cas de dengue (sérotype 3) survenant dans un camp militaire au cours d'une épidémie en Nouvelle-Calédonie (1989). La valeur de la lutte antivectorielle. Med Trop (Mars) 50: 423-428.

503. Berger S (1997) DENGUE - VIETNAM (05). In: Center TAM, editor. Tel Aviv: PROMED.

504. Berger S (1997) DENGUE - AUSTRALIA (QUEENSLAND) (02). In: mail P, editor: PROMED.

505. Berger S (1997) DENGUE-LIKE FEVER - SAMOA AND FIJI (02). PROMED.

506. Berger S (1997) DENGUE - COSTA RICA (02). PROMED.

507. Berger S (1997) DENGUE - TAIWAN: BACKGROUND. PROMED.

508. Berger S (1997) DENGUE - COLOMBIA (05): 1980-1997. PROMED.

509. Berger S (1998) DENGUE/DHF - INDONESIA. PROMED.

510. Berger S (1998) DENGUE/DHF - VIETNAM (02). (Vietnam): PROMED.

511. Berger S (1998) DENGUE - BRAZIL (02). Tel Aviv: PROMED.

512. Berger S (1998) DENGUE - THAILAND (02). Tel Aviv: PROMED.

513. Berger S (1998) DENGUE - INDIA (MAHARASHTRA) (02). GIDEON software ed. Tel Aviv: PROMED.

514. Berger S (1998) DENGUE - GUADELOUPE (02). PROMED.

515. Berger SA (1999) DENGUE/DHF - PHILIPPINES: BACKGROUND. In: ProMED-mail, editor: PROMED.

516. Berger SA (1999) DENGUE/DHF - HONDURAS: BACKGROUND. In: ProMED-mail, editor: PROMED.

517. Berger SA (2000) DENGUE - PARAGUAY: COMMENT (04). Tel Aviv: PROMED.

518. Berger SA (2001) DENGUE, IMPORTED - CHINA EX LAOS (02). The GIDEON Database, Sat 24 Aug 2001 [edited] ed: PROMED.

519. Berlioz A (2003) DENGUE/DHF UPDATE 2003 (11) [New Caledonia/ Indonesia]. In: ProMED-mail, editor: PROMED.

520. Berlioz A (2002) DENGUE/DHF UPDATES (21): 4 JUN 2002 [New Caledonia/ Malaysia/ Venezuela]. Institut Pasteur de Nouvelle Caledonie [edited] ed: PROMED.

521. Bern C, Hightower AW, Chowdhury R, Ali M, Amann J, et al. (2005) Risk Factors for Kala-Azar in Bangladesh. Emerg Infect Dis 11: 655-662.

522. Bernardo L, Izquierdo A, Prado I, Rosario D, Alvarez M, et al. (2008) Primary and secondary infections of Macaca fascicularis monkeys with Asian and American genotypes of dengue virus 2. Clin Vaccine Immunol 15: 439-446.

523. Bernardo L, Yndart A, Vazquez S, Morier L, Guzman MG (2005) Antibody responses to Asian and American genotypes of dengue 2 virus in immunized mice. Clin Diagn Lab Immunol 12: 361-362.

524. Berrington WR, Hitti J, Casper C (2007) A case report of dengue virus infection and acalculous cholecystitis in a pregnant returning traveler. Travel Med Infect Dis 5: 251-253.

525. Bethell DB, Flobbe K, Cao XT, Day NP, Pham TP, et al. (1998) Pathophysiologic and prognostic role of cytokines in dengue hemorrhagic fever. J Infect Dis 177: 778-782.

526. Bharaj P, Chahar HS, Pandey A, Diddi K, Dar L, et al. (2008) Concurrent infections by all four dengue virus serotypes during an outbreak of dengue in 2006 in Delhi, India. Virol J 5: -.

527. Bhatia V, Parida AK, Arora P, Mittal A, Pandey AK, et al. (2007) Electrocardiographic and echocardiographic findings during the recent outbreak of viral fever in National Capital Region. Indian Heart J 59: 360-362.

528. Bhattacharjee N, Mukherjee KK, Chakravarti SK, Mukherjee MK, De PN, et al. (1993) Dengue haemorrhagic fever (DHF) outbreak in Calcutta--1990. J Commun Dis 25: 10-14.

529. Bibi-Triki T, Aras N, Braun T, Lautridou C, Boukari L, et al. (2009) Plasmocytose médullaire périphérique et d'os dans la fièvre de dengue: Rapport d'un cas. Rev Med Interne 30: 274-276.

530. Bider B (2002) DENGUE/DHF UPDATES (44): 11 NOV 2002 [Indonesia]. The Jakarta Post, Sun 10 Nov 2002 [edited] ed: PROMED.

531. Binh PT, Matheus S, Huong VT, Deparis X, Marechal V (2009) Early clinical and biological features of severe clinical manifestations of dengue in Vietnamese adults. J Clin Virol 45: 276-280.

532. Biswas D, Dey S, Dutta RN, Hati AK (1993) Observations on the breeding habitats of Aedes aegypti in Calcutta following an episode of dengue haemorrhagic fever. Indian J Med Res 97: 44-46.

533. Black J (2000) DENGUE/DHF - EAST TIMOR (03). In: Dili WO, editor. Dili (East Timor): PROMED.

534. Black WCt, Gorrochetegui-Escalante N, Randle NP, Donnelly MJ (2008) The Yin and Yang of linkage disequilibrium: mapping of genes and nucleotides conferring insecticide resistance in insect disease vectors. Adv Exp Med Biol 627: 71-83.

535. Blackburn NK, Meenehan G, Aldridge N (1987) The status of dengue fever virus in South Africa--serological studies and diagnosis of a case of dengue fever. Trans R Soc Trop Med Hyg 81: 690-692.

536. Blackburn NK, Rawat R (1987) Dengue fever imported from India. A report of 3 cases. S Afr Med J 71: 386-387.

537. Blacksell SD, Bell D, Kelley J, Mammen MP, Jr., Gibbons RV, et al. (2007) Prospective study to determine accuracy of rapid serological assays for diagnosis of acute dengue virus infection in Laos. Clin Vaccine Immunol 14: 1458-1464.

538. Blacksell SD, Newton PN, Bell D, Kelley J, Mammen MP, et al. (2006) The comparative accuracy of 8 commercial rapid immunochromatographic assays for the diagnosis of acute dengue virus infection. Clin Infect Dis 42: 1127-1134.

539. Blacksell SD, Sharma NP, Phumratanaprapin W, Jenjaroen K, Peacock SJ, et al. (2007) Serological and blood culture investigations of Nepalese fever patients. Trans R Soc Trop Med Hyg 101: 686-690.

540. Blaney JE, Durbin AP, Murphy BR, Whitehead SS (2006) Development of a live attenuated dengue virus vaccine using reverse genetics. Viral Immunol 19: 10-32.

541. Blaney JE, Hanson CT, Firestone CY, Hanley KA, Murphy BR, et al. (2004) Genetically modified, live attenuated dengue virus type 3 vaccine candidates. Am J Trop Med Hyg 71: 811-821.

542. Blaney JE, Hanson CT, Hanley KA, Murphy BR, Whitehead SS (2004) Vaccine candidates derived from a novel infectious cDNA clone of an American genotype dengue virus type 2. BMC Infect Dis 4: -.

543. Blaney JE, Matro JM, Murphy BR, Whitehead SS (2005) Recombinant, live-attenuated tetravalent dengue virus vaccine formulations induce a balanced, broad, and protective neutralizing antibody response against each of the four serotypes in rhesus monkeys. J Virol 79: 5516-5528.

544. Blanton RE, Silva LK, Morato VG, Parrado AR, Dias JP, et al. (2008) Genetic ancestry and income are associated with dengue hemorrhagic fever in a highly admixed population. Eur J Hum Genet 16: 762-765.

545. Blok J, Gibbs AJ, McWilliam SM, Vitarana UT (1991) NS 1 gene sequences from eight dengue-2 viruses and their evolutionary relationships with other dengue-2 viruses. Arch Virol 118: 209-223.

546. Blok J, Kay BH, Hall RA, Gorman BM (1988) Isolation and characterization of dengue viruses serotype 1 from an epidemic in northern Queensland, Australia. Arch Virol 100: 213-220.

547. Blok J, Samuel S, Gibbs AJ, Vitarana UT (1989) Variation of the nucleotide and encoded amino acid sequences of the envelope gene from eight dengue-2 viruses. Arch Virol 105: 39-53.

548. Boisier P, Morvan JM, Laventure S, Charrier N, Martin E, et al. (1994) Dengue 1 épidémie dans l'île Grande Comore (République Fédérale Islamique des Comores). Mars-mai 1993. Ann Soc Belg Med Trop 74: 217-229.

549. Boladuadua A (1998) DENGUE - FIJI (14). In: Ministry of Health F, editor. (Fiji): PROMED.

550. Bongsebandhu-Phubhakdi C, Hemungkom M, Thisyakorn U, Thisyakom C (2008) Risk factors influencing severity in pediatric dengue infection. Asian Biomedicine 2: 409-413.

551. Bordignon J, Probst CM, Mosimann ALP, Pavoni DP, Stella V, et al. (2008) Expression profile of interferon stimulated genes in central nervous system of mice infected with dengue virus Type-1. Virology 377: 319-329.

552. Boromisa RD, Rai KS, Grimstad PR (1987) Variation in the vector competence of geographic strains of Aedes albopictus for dengue 1 virus. J Am Mosq Control Assoc 3: 378-386.

553. Bosio CF, Thomas RE, Grimstad PR, Rai KS (1992) Variation in the efficiency of vertical transmission of dengue-1 virus by strains of Aedes albopictus (Diptera: Culicidae). J Med Entomol 29: 985-989.

554. Botros BA, Watts DM, Soliman AK, Salib AW, Moussa MI, et al. (1989) Serological evidence of dengue fever among refugees, Hargeysa, Somalia. J Med Virol 29: 79-81.

555. Bottieau E, Clerinx J, Schrooten W, Van den Enden E, Wouters R, et al. (2006) Etiology and outcome of fever after a stay in the tropics. Arch Intern Med 166: 1642-1648.

556. Bottieau E, Clerinx J, Van den Enden E, Van Esbroeck M, Colebunders R, et al. (2007) Fever after a stay in the tropics: diagnostic predictors of the leading tropical conditions. Medicine (Baltim) 86: 18-25.

557. Bouldouyre MA, Baumann F, Berlioz-Arthaud A, Chungue E, Lacassin F (2006) Factors of severity at admission during an epidemic of dengue 1 in New Caledonia (South Pacific) in 2003. Scand J Infect Dis 38: 675-681.

558. Bouree P, Lancon A, Anquetil R, Menager C (2001) Dengue en Nouvelle-Calédonie. Etude de 68 cas pédiatriques. Arch Pediatr 8: 1311-1317.

559. Bozza FA, Cruz OG, Zagne SM, Azeredo EL, Nogueira RMR, et al. (2008) Multiplex cytokine profile from dengue patients: MIP-1beta and IFN-gamma as predictive factors for severity. BMC Infect Dis 8: -.

560. Braga EL, Moura P, Pinto LM, Ignacio SR, Oliveira MJ, et al. (2001) Detection of circulant tumor necrosis factor-alpha, soluble tumor necrosis factor p75 and interferon-gamma in Brazilian patients with dengue fever and dengue hemorrhagic fever. Mem Inst Oswaldo Cruz 96: 229-232.

561. Branch SL, Levett PN (1999) Evaluation of four methods for detection of immunoglobulin M antibodies to dengue virus. Clin Diagn Lab Immunol 6: 555-557.

562. Brandler S, Brown N, Ermak TH, Mitchell F, Parsons M, et al. (2005) Replication of chimeric yellow fever virus-Dengue serotype 1-4 virus vaccine strains in dendritic and hepatic cells. Am J Trop Med Hyg 72: 74-81.

563. Bravo JR, Guzman MG, Kouri GP (1987) Why dengue haemorrhagic fever in Cuba? 1. Individual risk factors for dengue haemorrhagic fever/dengue shock syndrome (DHF/DSS). Trans R Soc Trop Med Hyg 81: 816-820.

564. Bretner M, Baier A, Kopanska K, Najda A, Schoof A, et al. (2005) Synthesis and biological activity of 1H-benzotriazole and 1H-benzimidazole analogues--inhibitors of the NTpase/helicase of HCV and of some related Flaviviridae. Antivir Chem Chemother 16: 315-326.

565. Brisola Marcondes C (1998) DENGUE & AEDES - BRAZIL (SANTA CATARINA). In: Paras./CCB DM, editor. Florianopolis: PROMED.

566. Brisola Marcondes C (1999) DENGUE - BRAZIL (SAO PAULO) (02). newspaper Folha de Sao Paulo, Brazil 17 Jun 1999 ed. Florianopolis: PROMED.

567. Brito CA, Albuquerque Mde F, Lucena-Silva N (2007) Evidência de alterações de permeabilidade vascular na dengue: quando a dosagem de albumina sérica define o quadro? Rev Soc Bras Med Trop 40: 220-223.

568. Brown JL, Wilkinson R, Davidson RN, Wall R, Lloyd G, et al. (1996) Rapid diagnosis and determination of duration of viraemia in dengue fever using a reverse transcriptase polymerase chain reaction. Trans R Soc Trop Med Hyg 90: 140-143.

569. Brunkard JM, Robles Lopez JL, Ramirez J, Cifuentes E, Rothenberg SJ, et al. (2007) Dengue fever seroprevalence and risk factors, Texas-Mexico border, 2004. Emerg Infect Dis 13: 1477-1483.

570. Buchy P, Vo VL, Bui KT, Trinh TX, Glaziou P, et al. (2005) Secondary dengue virus type 4 infections in Vietnam. Southeast Asian J Trop Med Public Health 36: 178-185.

571. Buerano CC, Ibrahim IN, Contreras RC, Hasebe F, Matias RR, et al. (2000) IgM-capture ELISA of serum samples collected from Filipino dengue patients. Southeast Asian J Trop Med Public Health 31: 524-529.

572. Buerano CC, Natividad FF, Contreras RC, Ibrahim IN, Mangada MN, et al. (2008) Antigen sandwich ELISA predicts RT-PCR detection of dengue virus genome in infected culture fluids of Aedes albopictus C6/36 cells. Southeast Asian J Trop Med Public Health 39: 817-821.

573. Bulugahapitiya U, Siyambalapitiya S, Seneviratne SL, Fernando DJS (2007) Dengue fever in travellers: A challenge for European physicians. Eur J Intern Med 18: 185-192.

574. Bundo K, Igarashi A (1985) Antibody-capture ELISA for detection of immunoglobulin M antibodies in sera from Japanese encephalitis and dengue hemorrhagic fever patients. J Virol Methods 11: 15-22.

575. Bunyavejchevin S, Tanawattanacharoen S, Taechakraichana N, Thisyakorn U, Tannirandorn Y, et al. (1997) Dengue hemorrhagic fever during pregnancy: antepartum, intrapartum and postpartum management. J Obstet Gynaecol Res 23: 445-448.

576. Burattini MN, Chen M, Chow A, Coutinho FAB, Goh KT, et al. (2008) Modelling the control strategies against dengue in Singapore. Epidemiol Infect 136: 309-319.

577. Burivong P, Pattanakitsakul SN, Thongrungkiat S, Malasit P, Flegel TW (2004) Markedly reduced severity of Dengue virus infection in mosquito cell cultures persistently infected with Aedes albopictus densovirus (AalDNV). Virology 329: 261-269.

578. Burke DS, Kliks S (2006) Antibody-dependent enhancement in dengue virus infections. J Infect Dis 193: 601-603.

579. Burke DS, Nisalak A, Johnson DE, Scott RM (1988) A prospective study of dengue infections in Bangkok. Am J Trop Med Hyg 38: 172-180.

580. Bustos J, Hamdan A, Lorono MA, Montero MT, Gomez B (1990) Serologically proven acute rubella infection in patients with clinical diagnosis of dengue. Epidemiol Infect 104: 297-302.

581. Butrapet S, Kinney RA, Huang CYH (2006) Determining genetic stabilities of chimeric dengue vaccine candidates based on dengue 2 PDK-53 virus by sequencing and quantitative TaqMAMA. J Virol Methods 131: 1-9.

582. Butt N, Abbassi A, Munir SM, Ahmad SM, Sheikh QH (2008) Haematological and biochemical indicators for the early diagnosis of dengue viral infection. Jcpsp-Journal of the College of Physicians and Surgeons Pakistan 18: 282-285.

583. Butthep P, Chunhakan S, Tangnararatchakit K, Yoksan S, Pattanapanyasat K, et al. (2006) Elevated soluble thrombomodulin in the febrile stage related to patients at risk for dengue shock syndrome. Pediatr Infect Dis J 25: 894-897.

584. Cabezas S, Rojas G, Pavon A, Bernardo L, Castellanos Y, et al. (2009) Phage-displayed antibody fragments recognizing dengue 3 and dengue 4 viruses as tools for viral serotyping in sera from infected individuals. Arch Virol 154: 1035-1045.

585. Cabrera-Batista B, Skewes-Ramm R, Fermin CD, Garry RF (2005) Dengue in the Dominican Republic: Epidemiology for 2004. Microsc Res Tech 68: 250-254.

586. Cabrera-Cortina JI, Sanchez-Valdez E, Cedas-DeLezama D, Ramirez-Gonzalez MD (2008) Oral calcium administration attenuates thrombocytopenia in patients with dengue fever. Report of a pilot study. Proc West Pharmacol Soc 51: 38-41.

587. Caceres C, Yung V, Araya P, Tognarelli J, Villagra E, et al. (2008) Complete nucleotide sequence analysis of a Dengue-1 virus isolated on Easter Island, Chile. Arch Virol 153: 1967-1970.

588. Caiaffa WT, Almeida MC, Oliveira CD, Friche AA, Matos SG, et al. (2005) The urban environment from the health perspective: the case of Belo Horizonte, Minas Gerais, Brazil. Cad Saude Publica 21: 958-967.

589. Calisher CH (1997) DENGUE/DENGUE HEMORRHAGIC FEVER - CUBA (06). Fort Collins (Colorado): PROMED.

590. Calisher CH (1998) DENGUE - NEW CALEDONIA & COOK ISLANDS: DATA. In: ProMED-mail, editor: PROMED.

591. Callaway E (2007) Dengue fever climbs the social ladder. Nature 448: 734-735.

592. Calvert AE, Huang CYH, Kinney RM, Roehrig JT (2006) Non-structural proteins of dengue 2 virus offer limited protection to interferon-deficient mice after dengue 2 virus challenge. J Gen Virol 87: 339-346.

593. Cam BV, Fonsmark L, Hue NB, Phuong NT, Poulsen A, et al. (2001) Prospective case-control study of encephalopathy in children with dengue hemorrhagic fever. Am J Trop Med Hyg 65: 848-851.

594. Cam BV, Tuan DT, Fonsmark L, Poulsen A, Tien NM, et al. (2002) Randomized comparison of oxygen mask treatment vs. nasal continuous positive airway pressure in dengue shock syndrome with acute respiratory failure. J Trop Pediatr 48: 335-339.

595. Camacho DE, Alvarez M, Rodriguez-Henriquez F, de Quintana M, Soler M, et al. (2003) Laboratorio de diagnóstico de las infecciones del virus del dengue en el estado Aragua, Venezuela: octubre 1997-diciembre 1998. Invest Clin 44: 91-103.

596. Camacho T, de la Hoz F, Cardenas V, Sanchez C, de Calderon L, et al. (2004) Incomplete surveillance of a dengue-2 epidemic in Ibague, Colombia, 1995-1997. Biomedica 24: 174-182.

597. Camara FP, Gomes AF, dos Santos GT, Camara DCP (2009) Clima e epidemias de dengue no Estado do Rio de Janeiro. Rev Soc Bras Med Trop 42: 137-140.

598. Camara FP, Theophilo RLG, dos Santos GT, Pereira SRFG, Camara DCP, et al. (2007) Estudo retrospectivo (histórico) da dengue no Brasil: características regionais e dinâmicas. Rev Soc Bras Med Trop 40: 192-196.

599. Camion VL (2003) DENGUE/DHF UPDATE 2003 (35) [Laos/ India/ Philippines]. In: ProMED-mail, editor. Organisation Of Asia-Pacific News Agencies 28 Aug 2003 [edited] ed: PROMED.

600. Campagna Dde S, Miagostovich MP, Siqueira MM, Cunha RV (2006) Etiology of exanthema in children in a dengue endemic area. J Pediatr (Rio J) 82: 354-358.

601. Campbell CA, George A, Salas RA, Williams SA, Doon R, et al. (2007) Seroprevalence of dengue in Trinidad using rapid test kits: A cord blood survey. Acta Trop 101: 153-158.

602. Campbell D (1997) DENGUE - VIETNAM. media reports ed: PROMED.

603. Campione-Piccardo J, Ruben M, Vaughan H, Morris-Glasgow V (2003) Dengue viruses in the Caribbean. Twenty years of dengue virus isolates from the Caribbean Epidemiology Centre. West Indian Med J 52: 191-198.

604. Candido Carlos M (2004) DENGUE/DHF UPDATE 2004 (08) [Brazil/ Venezuela]. In: ProMED-mail, editor. EPTV Sul de Minas 2 Mar 2004 18:42 [in Portuguese, summarized by Mod.JW] ed: PROMED.

605. Cao XT, Ngo TN, Kneen R, Bethell D, Le TD, et al. (2004) Evaluation of an algorithm for integrated management of childhood illness in an area of Vietnam with dengue transmission. Trop Med Int Health 9: 573-581.

606. Cao XT, Ngo TN, Wills B, Kneen R, Nguyen TT, et al. (2002) Evaluation of the World Health Organization standard tourniquet test and a modified tourniquet test in the diagnosis of dengue infection in Viet Nam. Trop Med Int Health 7: 125-132.

607. Capeding MR, Paladin FJ, Miranda EG, Navarro XR (1997) Dengue surveillance in Metro Manila. Southeast Asian J Trop Med Public Health 28: 530-534.

608. Caraballo A, Hernandez J (1991) Brote del dengue en San José de Guaribe, Venezuela. Informe preliminar. Rev Inst Med Trop Sao Paulo 33: 413-415.

609. Carbajo AE, Gomez SM, Curto SI, Schweigmann NJ (2004) Variabilidad espacio-temporal en la transmisión del dengue en la ciudad de Buenos Aires. Medicina (Mex) 64: 231-234.

610. Carbajo AE, Schweigmann N, Curto SI, de Garin A, Bejaran R (2001) Dengue transmission risk maps of Argentina. Trop Med Int Health 6: 170-183.

611. Cardenas V (1995) DENGUE/DHF - COLOMBIA. In: FETP C, editor. IQCB-Biweekly Report of Cases and Outbreaks. (Colombia): PROMED.

612. Cardenas V (1997) DENGUE - COLOMBIA: UNDER-/MIS-REPORTING. (Colombia): PROMED.

613. Cardier JE, Balogh V, Perez-Silva C, Romano E, Rivas B, et al. (2006) Relationship of thrombopoietin and interleukin-11 levels to thrombocytopenia associated with dengue disease. Cytokine 34: 155-160.

614. Cardier JE, Marino E, Romano E, Taylor P, Liprandi F, et al. (2005) Proinflammatory factors present in sera from patients with acute dengue infection induce activation and apoptosis of human microvascular endothelial cells: Possible role of TNF-alpha in endothelial cell damage in dengue. Cytokine 30: 359-365.

615. Cardier JE, Rivas B, Romano E, Rothman AL, Perez-Perez C, et al. (2006) Evidence of vascular damage in dengue disease: Demonstration of high levels of soluble cell adhesion molecules and circulating endothelial cells. Endothelium-Journal of Endothelial Cell Research 13: 335-340.

616. Cardosa J, Ooi MH, Tio PH, Perera D, Holmes EC, et al. (2009) Dengue Virus Serotype 2 from a Sylvatic Lineage Isolated from a Patient with Dengue Hemorrhagic Fever. PLoS Negl Trop Dis 3: -.

617. Cardosa MJ (1987) Dengue virus isolation by antibody-dependent enhancement of infectivity in macrophages. Lancet 1: 193-194.

618. Cardosa MJ, Baharudin F, Hamid S, Hooi TP, Nimmanitya S (1995) A nitrocellulose membrane based IgM capture enzyme immunoassay for etiological diagnosis of dengue virus infections. Clin Diagn Virol 3: 343-350.

619. Cardosa MJ, Zuraini I (1991) Comparison of an IgM capture ELISA with a dot enzyme immunoassay for laboratory diagnosis of dengue virus infections. Southeast Asian J Trop Med Public Health 22: 337-340.

620. Cardoso Junior RP, Scandar SA, de Mello NV, Ernandes S, Botti MV, et al. (1996) Detecção de Aedes aegypti e Aedes albopictus, na zona urbana do município de Catanduva, SP, após o controle de uma epidemia de Dengue. Rev Soc Bras Med Trop 30: 37-40.

621. Carles G, Peiffer H, Talarmin A (1999) Effects of dengue fever during pregnancy in French Guiana. Clin Infect Dis 28: 637-640.

622. Carlos CC, Oishi K, Cinco MTDD, Mapua CA, Inoue S, et al. (2005) Comparison of clinical features and hematologic abnormalities between dengue fever and dengue hemorrhagic fever among children in the Philippines. Am J Trop Med Hyg 73: 435-440.

623. Carlos M, Banks A-L (2007) Dengue/DHF update 2007 (13). PROMED: Promed.

624. Carme B, Matheus S, Donutil G, Raulin O, Nacher M, et al. (2009) Concurrent Dengue and Malaria in Cayenne Hospital, French Guiana. Emerg Infect Dis 15: 668-671.

625. Carme B, Sobesky M, Biard MH, Cotellon P, Aznar C, et al. (2003) Non-specific alert system for dengue epidemic outbreaks in areas of endemic malaria. A hospital-based evaluation in Cayenne (French Guiana). Epidemiol Infect 130: 93-100.

626. Carrington CVF, Foster JE, Pybus OG, Bennett SN, Holmes EC (2005) Invasion and maintenance of Dengue virus type 2 and type 4 in the Americas. J Virol 79: 14680-14687.

627. Carroll ID, Toovey S, Van Gompel A (2007) Dengue fever and pregnancy - a review and comment. Travel Med Infect Dis 5: 183-188.

628. Carson RS (1995) DENGUE, CHOLERA, EQUINE ENCEPHALITIS - VENEZUELA. In: Shea J, editor. Caracas: PROMED.

629. Carter K (1995) DENGUE - CARIBBEAN (3). In: PAHO, editor: PROMED.

630. Casali CG, Pereira MR, Santos LM, Passos MN, Fortes Bde P, et al. (2004) A epidemia de dengue/dengue hemorrágico no município do Rio de Janeiro, 2001/2002. Rev Soc Bras Med Trop 37: 296-299.

631. Castle T, Amador M, Rawlins S, Figueroa JP, Reiter P (1999) Absence of impact of aerial malathion treatment on Aedes aegypti during a dengue outbreak in Kingston, Jamaica. Rev Panam Salud Publica 5: 100-105.

632. Castleberry JS, Mahon CR (2003) Dengue fever in the Western Hemisphere. Clin Lab Sci 16: 34-38.

633. Castro MG, Nogueira RM, Schatzmayr HG, Miagostovich MP, Lourenco-de-Oliveira R (2004) Dengue virus detection by using reverse transcription-polymerase chain reaction in saliva and progeny of experimentally infected Aedes albopictus from Brazil. Mem Inst Oswaldo Cruz 99: 809-814.

634. Catteau A, Kalinina O, Wagner MC, Deubel V, Courageot MP, et al. (2003) Dengue virus M protein contains a proapoptotic sequence referred to as ApoptoM. J Gen Virol 84: 2781-2793.

635. Cecilio AB, Campanelli ES, Souza KPR, Figueiredo LB, Resende MC (2009) Natural vertical transmission by Stegomyia albopicta as dengue vector in Brazil. Braz J Biol 69: 123-127.

636. Cerdas M (2006) Epidemiology and control of hypertension and diabetes in Costa Rica. Ren Fail 28: 693-696.

637. Cerdas-Quesada CA, Retana-Salazar AP (2007) Characterization and phylogenetic relationships of strains of Dengue type 1 virus from Costa Rica. Rev Biol Trop 55: 365-372.

638. Cesaire R, Cabie A, Djossou F, Lamaury I, Beaucaire G, et al. (2008) Dengue dans la version française Indes occidentales. Virologie 12: 151-157.

639. Cestero-Rivera R, Ramirez-Rivera J, Jaume-Anselmi F, Guerrero-Dujarric O (2006) Acute renal failure in a man with a small farm. Boletín de la Asociación Médica de Puerto Rico 98: 114-116.

640. Chabasse D, De Bray JM, Le Lay-Rogues G, Nedelec C, Chastel C (1986) Syndrome encéphalitique causée par un flavivirus diagnostiqués après le retour d'un voyage en Inde du Sud: sans doute la dengue avec des manifestations neurologiques. Bull Soc Pathol Exot Filiales 79: 531-538.

641. Chacko B, Subramanian G (2008) Clinical, laboratory and radiological parameters in children with dengue fever and predictive factors for dengue shock syndrome. J Trop Pediatr 54: 137-140.

642. Chadee DD (2003) Surveillance for the dengue vector Aedes aegypti in Tobago, West Indies. J Am Mosq Control Assoc 19: 199-205.

643. Chadee DD (2009) Dengue cases and Aedes aegypti indices in Trinidad, West Indies. Acta Trop 112: 174-180.

644. Chadee DD, Martinez R (2000) Landing periodicity of Aedes aegypti with implications for dengue transmission in Trinidad, West Indies. J Vector Ecol 25: 158-163.

645. Chadee DD, Williams FLR, Kitron UD (2005) Impact of vector control on a dengue fever outbreak in Trinidad, West Indies, in 1998. Trop Med Int Health 10: 748-754.

646. Chadha MS, Arankalle VA, Jadi RS, Joshi MV, Thakare JP, et al. (2005) An outbreak of chandipura virus encephalitis in the eastern districts of Gujarat State, India. Am J Trop Med Hyg 73: 566-570.

647. Chadwick D, Arch B, Wilder-Smith A, Paton N (2006) Distinguishing dengue fever from other infections on the basis of simple clinical and laboratory features: Application of logistic regression analysis. J Clin Virol 35: 147-153.

648. Chai LY, Lim PL, Lee CC, Hsu LY, Teoh YL, et al. (2007) Cluster of Staphylococcus aureus and dengue co-infection in Singapore. Ann Acad Med Singap 36: 847-850.

649. Chaikoolvatana A, Chanruang S, Pothaled P (2008) A comparison of dengue hemorrhagic fever control interventions in northeastern Thailand. Southeast Asian J Trop Med Public Health 39: 617-624.

650. Chairulfatah A, Setiabudi D, Agoes R, van Sprundel M, Colebunders R (2001) Hospital based clinical surveillance for dengue haemorrhagic fever in Bandung, Indonesia 1994-1995. Acta Trop 80: 111-115.

651. Chairulfatah A, Setiabudi D, Ridad A, Colebunders R (1995) Clinical manifestations of dengue haemorrhagic fever in children in Bandung, Indonesia. Ann Soc Belg Med Trop 75: 291-295.

652. Chaiyaratana W, Chuansumrit A, Atamasirikul K, Tangnararatchakit K (2008) Serum ferritin levels in children with dengue infection. Southeast Asian J Trop Med Public Health 39: 832-836.

653. Chaiyaratana W, Chuansumrit A, Pongthanapisith V, Tangnararatchakit K, Lertwongrath S, et al. (2009) Evaluation of dengue nonstructural protein 1 antigen strip for the rapid diagnosis of patients with dengue infection. Diagn Microbiol Infect Dis 64: 83-84.

654. Chakravarti A, Kumaria R (2005) Eco-epidemiological analysis of dengue infection during an outbreak of dengue fever, India. Virol J 2: 32.

655. Chakravarti A, Kumaria R (2006) Circulating levels of tumour necrosis factor-alpha & interferon-gamma; in patients with dengue & dengue haemorrhagic fever during an outbreak. Indian J Med Res 123: 25-30.

656. Chalupa P, Kubek J, Hejlova A (2001) Dengue fever in the Czech Republic. Bratisl Lek Listy 102: 322-325.

657. Chan CP, Choi JW, Cao KY, Wang M, Gao Y, et al. (2006) Detection of serum neopterin for early assessment of dengue virus infection. J Infect.

658. Chan DPL, Teoh SCB, Tan CSH, Nah GKM, Rajagopalan R, et al. (2006) Ophthalmic complications of dengue. Emerg Infect Dis 12: 285-289.

659. Chan KP, Lau GK, Doraisingham S, Chan YC (1995) Adult dengue deaths in Singapore. Clin Diagn Virol 4: 213-222.

660. Chanama S, Anantapreecha S, A-nuegoonpipat A, Sa-gnasang A, Kurane I, et al. (2004) Analysis of specific IgM responses in secondary dengue virus infections: levels and positive rates in comparison with primary infections. J Clin Virol 31: 185-189.

661. Chandralekha, Pratyush G, Anjan T (2008) The north Indian dengue outbreak 2006: a retrospective analysis of intensive care unit admissions in a tertiary care hospital. Trans R Soc Trop Med Hyg 102: 143-147.

662. Chang AY, Parrales ME, Jimenez J, Sobieszczyk ME, Hammer SM, et al. (2009) Combining Google Earth and GIS mapping technologies in a dengue surveillance system for developing countries. Int J Health Geogr 8: 49.

663. Chang CS, Harn MR, Nimmannitya S (1990) [Clinical observation of 15 Thai children with dengue hemorrhagic fever]. Gaoxiong Yi Xue Ke Xue Za Zhi 6: 131-136.

664. Chang PE, Cheng CL, Asok K, Fong KY, Chee SP, et al. (2007) Visual disturbances in dengue fever: an answer at last? Singapore Med J 48: e71-73.

665. Chang SF, Huang JH, Chen LK, Su CL, Liao TL, et al. (2008) Retrospective serological study on sequential dengue virus serotypes 1 to 4 epidemics in Tainan City, Taiwan, 1994 to 2000. J Microbiol Immunol Infect 41: 377-385.

666. Chang TH, Liao CL, Lin YL (2006) Flavivirus induces interferon-beta gene expression through a pathway involving RIG-I-dependent IRF-3 and PI3K-dependent NF-kappa B activation. Microbes Infect 8: 157-171.

667. Chanprapaph S, Saparpakorn P, Sangma C, Niyomrattanakit P, Hannongbua S, et al. (2005) Competitive inhibition of the dengue virus NS3 serine protease by synthetic peptides representing polyprotein cleavage sites. Biochem Biophys Res Commun 330: 1237-1246.

668. Chan-Tack KM (2002) Dengue fever in mid-Missouri. A case report. Mo Med 99: 259-261.

669. Chanthavanich P, Luxemburger C, Sirivichayakul C, Lapphra K, Pengsaa K, et al. (2006) Short report: Immune response and occurrence of dengue infection in thai children three to eight years after vaccination with live attenuated tetravalent dengue vaccine. Am J Trop Med Hyg 75: 26-28.

670. Chanyasanha C, Teetipasatit S, Sujirarat S, Kaewpan W (2008) Factors Associated with Preventive Behavior on Dengue Hemorrhagic Fever Among Family Leaders in One Sub-District of Bangkok, Thailand. Int J Infect Dis 12: E438-E439.

671. Chao DY, King CC, Wang WK, Chen WJ, Wu HL, et al. (2005) Strategically examining the full-genome of dengue virus type 3 in clinical isolates reveals its mutation spectra. Virol J 2: 72.

672. Chao YC, Huang CS, Lee CN, Chang SY, King CC, et al. (2008) Higher infection of dengue virus serotype 2 in human monocytes of patients with G6PD deficiency. PLoS One 3: e1557.

673. Chareonsook O, Foy HM, Teeraratkul A, Silarug N (1999) Changing epidemiology of dengue hemorrhagic fever in Thailand. Epidemiol Infect 122: 161-166.

674. Chareonviriyaphap T, Akratanakul P, Huntamai S, Nettanomsak S, Prabaripai A (2004) Allozyme patterns of Aedes albopictus, a vector of dengue in Thailand. J Med Entomol 41: 657-663.

675. Charnsilpa W, Takhampunya R, Endy TP, Mammen MP, Libraty DH, et al. (2005) Nitric oxide radical suppresses replication of wild-type dengue 2 viruses in vitro. J Med Virol 77: 89-95.

676. Chatterjee S, Chattopadhyay D, Bhattacharya MK, Mukherjee B (2004) Serosurveillance for Japanese encephalitis in children in several districts of West Bengal, India. Acta Paediatr 93: 390-393.

677. Chatterjee TK, Nayak K, Som S, Chatterjee S, Chaudhuris N, et al. (2007) Serological aspects of Dengue fever and its correlation with clinical features in a recent febrile outbreak. Indian J Public Health 51: 130-131.

678. Chaturvedi UC (2006) The curse of dengue. Indian J Med Res 124: 467-470.

679. Chaturvedi UC (2006) Tumour necrosis factor & dengue. Indian J Med Res 123: 11-14.

680. Chaturvedi UC (2009) Shift to Th2 cytokine response in dengue haemorrhagic fever. Indian J Med Res 129: 1-3.

681. Chaturvedi UC, Nagar R, Shrivastava R (2006) Dengue and dengue haemorrhagic fever: implications of host genetics. FEMS Immunol Med Microbiol 47: 155-166.

682. Chaudhary R, Khetan D, Sinha S, Sinha P, Sonker A, et al. (2006) Transfusion support to Dengue patients in a hospital based blood transfusion service in north India. Transfus Apher Sci 35: 239-244.

683. Chavasse D (2001) Know your enemy, some facts about the natural history Of Malawi's Anopheles mosquitoes and implications for malaria control. Malawi Medical Journal: 7-8.

684. Chee HY, AbuBakar S (2004) Identification of a 48 kDa tubulin or tubulin-like C6/36 mosquito cells protein that binds dengue virus 2 using mass spectrometry. Biochem Biophys Res Commun 320: 11-17.

685. Chen HC, Lai SY, Sung JM, Lee SH, Lin YC, et al. (2004) Lymphocyte activation and hepatic cellular infiltration in immunocompetent mice infected by dengue virus. J Med Virol 73: 419-431.

686. Chen HL, Lin SR, Liu HF, King CC, Hsieh SC, et al. (2008) Evolution of dengue virus type 2 during two consecutive outbreaks with an increase in severity in southern Taiwan in 2001-2002. Am J Trop Med Hyg 79: 495-504.

687. Chen LC, Lei HY, Liu CC, Shiesh SC, Chen SH, et al. (2006) Correlation of serum levels of macrophage migration inhibitory factor with disease severity and clinical outcome in dengue patients. Am J Trop Med Hyg 74: 142-147.

688. Chen LH, Wilson ME (2004) Transmission of dengue virus without a mosquito vector: nosocomial mucocutaneous transmission and other routes of transmission. Clin Infect Dis 39: e56-60.

689. Chen M (1997) DENGUE/DENGUE HEMORRHAGIC FEVER - MALAYSIA (03). PROMED.

690. Chen M (2002) DENGUE/DHF UPDATES (50): 30 DEC 2002 [Taiwan]. In: ProMED-mail, editor. Taipei Times, Sat 28 Dec 2002 [edited] ed: PROMED.

691. Chen RF, Liu JW, Yeh WT, Wang L, Chang JC, et al. (2005) Altered T helper 1 reaction but not increase of virus load in patients with dengue hemorrhagic fever. FEMS Immunol Med Microbiol 44: 43-50.

692. Chen RF, Wang L, Cheng JT, Chuang H, Chang JC, et al. (2009) Combination of CTLA-4 and TGF beta 1 gene polymorphisms associated with dengue hemorrhagic fever and virus load in a dengue-2 outbreak. Clin Immunol 131: 404-409.

693. Chen RF, Yang KD, Wang L, Liu JW, Chiu CC, et al. (2007) Different clinical and laboratory manifestations between dengue haemorrhagic fever and dengue fever with bleeding tendency. Trans R Soc Trop Med Hyg 101: 1106-1113.

694. Chen S (1993) [A study on a local epidemic of dengue fever in Shaxi district of Zhongshan city, 1991]. Zhonghua Liu Xing Bing Xue Za Zhi 14: 84-86.

695. Chen TC, Perng DS, Tsai JJ, Lu PL, Chen TP (2004) Dengue hemorrhagic fever complicated with acute pancreatitis and seizure. J Formos Med Assoc 103: 865-868.

696. Chen TY, Lee CT (2007) Guillain-Barre syndrome following dengue fever. Ann Emerg Med 50: 94-95.

697. Chen WH (2006) An unusual transitory increase of lupus anticoagulant in dengue virus infection complicated with cerebral ischaemia. J Infect 52: e87-91.

698. Chen WJ, Chen SL, Chien LJ, Chen CC, King CC, et al. (1996) Silent transmission of the dengue virus in southern Taiwan. Am J Trop Med Hyg 55: 12-16.

699. Chen WJ, Hwang KP, Fang AH (1991) Detection of IgM antibodies from cerebrospinal fluid and sera of dengue fever patients. Southeast Asian J Trop Med Public Health 22: 659-663.

700. Chen WJ, King CC, Chien LY, Chen SL, Fang AH (1997) Changing prevalence of antibody to Dengue virus in paired sera in the two years following an epidemic in Taiwan. Epidemiol Infect 119: 277-279.

701. Chen WJ, Wei HL, Hsu EL, Chen ER (1993) Vector competence of Aedes albopictus and Ae. aegypti (Diptera: Culicidae) to dengue 1 virus on Taiwan: development of the virus in orally and parenterally infected mosquitoes. J Med Entomol 30: 524-530.

702. Chen YC, Huang HN, Lin CT, Chen YF, King CC, et al. (2007) Generation and characterization of monoclonal antibodies against dengue virus type 1 for epitope mapping and serological detection by epitope-based peptide antigens. Clin Vaccine Immunol 14: 404-411.

703. Chen YR, Hwang JS, Guo YJ (1994) Ecology and control of dengue vector mosquitoes in Taiwan. Gaoxiong Yi Xue Ke Xue Za Zhi 10 Suppl: S78-87.

704. Chen Z, Tian Y, Liu L, An J (2008) Production of a monoclonal antibody against non-structural protein 3 of dengue-2 virus by intrasplenic injection. Hybridoma (Larchmt) 27: 467-471.

705. Cheng HJ, Lin CF, Lei HY, Liu HS, Yeh TM, et al. (2009) Proteomic Analysis of Endothelial Cell Autoantigens Recognized by Anti-Dengue Virus Nonstructural Protein 1 Antibodies. Exp Biol Med 234: 63-73.

706. Cheng MH (2009) Winning research: charting the Hong Kong experience with dengue fever. Hong Kong Med J 15: 80.

707. Cheong CY (1999) DENGUE/DHF ADVISORY - MALAYSIA. In: ProMED-mail, editor. Bernama, 28 Apr 1999 [edited] ed: PROMED.

708. Cheong CY (1999) DENGUE - MALAYSIA: CONTROL. In: ProMED-mail, editor. New Straits Times, Tuesday, 2 Feb 1999 [edited] ed: PROMED.

709. Cheong CY (1999) DENGUE - MALAYSIA: 1998 [Borneo]. In: ProMED-mail, editor. BERNAMA, Wednesday, 27 January 1999 [edited] ed: PROMED.

710. Cheong CY (1999) DENGUE - MALAYSIA (SARAWAK). In: ProMED-mail, editor: PROMED.

711. Cheong CY (1999) DENGUE/DHF - MALAYSIA: UPDATE. In: ProMED-mail, editor. Bernama, 25 Jul 1999 [edited] ed: PROMED.

712. Cheong CY (1999) DENGUE - MALAYSIA (SARAWAK) (02). In: ProMED-mail, editor. Sarawak Tribune, 15 Jul 1999 [edited] ed: PROMED.

713. Cheong CY (1999) DENGUE - PHILIPPINES. In: ProMED-mail, editor. Manila Bulletin, 15 May 1999 [edited] ed: PROMED.

714. Cheong CY (1999) DENGUE/DHF - MEXICO & USA. In: ProMED-mail, editor. Nando Times, 22 Oct 1999 ed: PROMED.

715. Cher D (2004) DENGUE/DHF UPDATE 2004 (23) [Singapore]. In: ProMED-mail, editor. Channel News Asia, 27 Jul 2004 [edited] ed: PROMED.

716. Cherian T, Ponnuraj E, Kuruvilla T, Kirubakaran C, John TJ, et al. (1994) An epidemic of dengue haemorrhagic fever & dengue shock syndrome in & around Vellore. Indian J Med Res 100: 51-56.

717. Chhabra A, Malhotra N (2006) Anesthetic management of a pregnant patient with dengue hemorrhagic fever for emergency cesarean section. Int J Obstet Anesth 15: 306-310.

718. Chhina D, Goyal P, Goyal O, Kumar R, Chhina RS (2008) A Study of Clinical Profile of Dengue Fever in Punjab, North India. Int J Infect Dis 12: E98-E98.

719. Chhina DK, Goyal O, Goyal P, Kumar R, Puri S, et al. (2009) Haemorrhagic manifestations of dengue fever & their management in a tertiary care hospital in north India. Indian J Med Res 129: 718-720.

720. Chhina R, Goyal O, Chhina D, Goyal P, Berry A, et al. (2009) Liver Dysfunction in Dengue Fever. J Hepatol 50: S171-S171.

721. Chia A, Luu CD, Mathur R, Cheng B, Chee SP (2006) Electrophysiological findings in patients with dengue-related maculopathy. Arch Ophthalmol 124: 1421-1426.

722. Chien J, Ong A, Low SY (2008) An unusual complication of dengue infection. Singapore Med J 49: E340-E342.

723. Chien LJ, Liao TL, Shu PY, Huang JH, Gubler DJ, et al. (2006) Development of real-time reverse transcriptase PCR assays to detect and serotype dengue viruses. J Clin Microbiol 44: 1295-1304.

724. Chimelli L, Hahn MD, Netto MB, Ramos RG, Dias M, et al. (1990) Dengue: neuropathological findings in 5 fatal cases from Brazil. Clin Neuropathol 9: 157-162.

725. Chin PS, Khoo AP, Asmah Hani AW, Chem YK, Norizah I, et al. (2008) Acute dengue in a neonate secondary to perinatal transmission. Med J Malaysia 63: 265-266.

726. Chingsuwanrote P, Suksanpaisan L, Smith DR (2004) Adaptation of the plaque assay methodology for dengue virus infected HepG2 cells. J Virol Methods 116: 119-121.

727. Chinnawirotpisan P, Mammen MP, Nisalak A, Thaisomboonsuk B, Narupiti S, et al. (2008) Detection of concurrent infection with multiple dengue virus serotypes in Thai children by ELISA and nested RT-PCR assay. Arch Virol 153: 2225-2232.

728. Chiu WW, Kinney RM, Dreher TW (2005) Control of translation by the 5 '- and 3 '-terminal regions of the dengue virus genome. J Virol 79: 8303-8315.

729. Chiu YC, Wu KL, Kuo CH, Hu TH, Chou YP, et al. (2005) Endoscopic findings and management of dengue patients with upper gastrointestinal bleeding. Am J Trop Med Hyg 73: 441-444.

730. Chlebicki MP, Ang B, Barkham T, Laude A (2005) Retinal hemorrhages in 4 patients with dengue fever. Emerg Infect Dis 11: 770-772.

731. Cho Min N (2000) Assessment of dengue hemorrhagic fever in Myanmar. Southeast Asian J Trop Med Public Health 31: 636-641.

732. Chongsrisawat V, Hutagalung Y, Poovorawan Y (2009) Liver Function Test Results and Outcomes in Children with Acute Liver Failure Due to Dengue Infection. Southeast Asian J Trop Med Public Health 40: 47-53.

733. Choochote W, Chaithong U, Kamsuk K, Rattanachanpichai E, Jitpakdi A, et al. (2006) Adulticidal activity against Stegomyia aegypti (Diptera: Culicidae) of three Piper spp. Rev Inst Med Trop Sao Paulo 48: 33-37.

734. Choochote W, Tippawangkosol P, Jitpakdi A, Sukontason KL, Pitasawat B, et al. (2001) Polygamy: the possibly significant behavior of Aedes aegypti and Aedes albopictus in relation to the efficient transmission of dengue virus. Southeast Asian J Trop Med Public Health 32: 745-748.

735. Chotigeat U, Kalayanarooj S, Nisalak A (2003) Vertical transmission of dengue infection in Thai infants: two case reports. J Med Assoc Thai 86 Suppl 3: S628-632.

736. Chotmongkol V, Sawanyawisuth K (2004) Case report: Dengue hemorrhagic fever with encephalopathy in an adult. Southeast Asian J Trop Med Public Health 35: 160-161.

737. Chouhan GS, Rodrigues FM, Shaikh BH, Ilkal MA, Khangaro SS, et al. (1990) Clinical & virological study of dengue fever outbreak in Jalore city, Rajasthan 1985. Indian J Med Res 91: 414-418.

738. Chow VT, Chan YC, Yong R, Lee KM, Lim LK, et al. (1998) Monitoring of dengue viruses in field-caught Aedes aegypti and Aedes albopictus mosquitoes by a type-specific polymerase chain reaction and cycle sequencing. Am J Trop Med Hyg 58: 578-586.

739. Chowdhury M (2000) DENGUE/DHF - BANGLADESH (02). PROMED.

740. Chowdhury MA (2000) DENGUE/DHF - BANGLADESH (04). In: Control SP, editor. News media [edited] ed. Dhaka: PROMED.

741. Chowdhury N, Ghosh A, Chandra G (2008) Mosquito larvicidal activities of Solanum villosum berry extract against the dengue vector Stegomyia aegypti. BMC Complement Altern Med 8: -.

742. Chu JJH, Leong PWH, Ng ML (2005) Characterization of plasma membrane-associated proteins from Aedes albopictus mosquito (C6/36) cells that mediate West Nile virus binding and infection. Virology 339: 249-260.

743. Chu JJH, Rajamanonmani R, Li J, Bhuvanakantham R, Lescar J, et al. (2005) Inhibition of West Nile virus entry by using a recombinant domain III from the envelope glycoprotein. J Gen Virol 86: 405-412.

744. Chua JJE, Bhuvanakantham R, Chow VTK, Ng ML (2005) Recombinant non-structural 1 (NS1) protein of dengue-2 virus interacts with human STAT3 beta protein. Virus Res 112: 85-94.

745. Chua JJE, Ng MML, Chow VTK (2004) The non-structural 3 (NS3) protein of dengue virus type 2 interacts with human nuclear receptor binding protein and is associated with alterations in membrane structure. Virus Res 102: 151-163.

746. Chua SK, Selvanesan S, Sivalingam B, Chem YK, Norizah I, et al. (2006) Isolation of monoclonal antibodies-escape variant of dengue virus serotype 1. Singapore Med J 47: 940-946.

747. Chuang VW, Wong TY, Leung YH, Ma ES, Law YL, et al. (2008) Review of dengue fever cases in Hong Kong during 1998 to 2005. Hong Kong Med J 14: 170-177.

748. Chuansumrit A, Chaiyaratana W, Pongthanapisith V, Tangnararatchakit K, Lertwongrath S, et al. (2008) The use of dengue nonstructural protein 1 antigen for the early diagnosis during the febrile stage in patients with dengue infection. Pediatr Infect Dis J 27: 43-48.

749. Chuansumrit A, Phimolthares V, Tardtong P, Tapaneya-Olarn C, Tapaneya-Olarn W, et al. (2000) Transfusion requirements in patients with dengue hemorrhagic fever. Southeast Asian J Trop Med Public Health 31: 10-14.

750. Chuansumrit A, Tangnararatchakit K, Lektakul Y, Pongthanapisith V, Nimjaroenniyom N, et al. (2004) The use of recombinant activated factor VII for controlling life-threatening bleeding in Dengue Shock Syndrome. Blood Coagul Fibrinolysis 15: 335-342.

751. Chuansumrit A, Wangruangsatid S, Lektrakul Y, Chua MN, Capeding MRZ, et al. (2005) Control of bleeding in children with Dengue hemorrhagic fever using recombinant activated factor VII: a randomized, double-blind, placebo-controlled study. Blood Coagul Fibrinolysis 16: 549-555.

752. Chung YK, Pang FY (2002) Dengue virus infection rate in field populations of female Aedes aegypti and Aedes albopictus in Singapore. Trop Med Int Health 7: 322-330.

753. Chungue E, Boutin JP, Roux J (1991) Dengue surveillance in French Polynesia: an attempt to use the excess number of laboratory requests for confirmation of dengue diagnosis as an indicator of dengue activity. Eur J Epidemiol 7: 616-620.

754. Chungue E, Burucoa C, Boutin JP, Philippon G, Laudon F, et al. (1992) Dengue 1 epidemic in French Polynesia, 1988-1989: surveillance and clinical, epidemiological, virological and serological findings in 1752 documented clinical cases. Trans R Soc Trop Med Hyg 86: 193-197.

755. Chungue E, Deubel V, Cassar O, Laille M, Martin PM (1993) Molecular epidemiology of dengue 3 viruses and genetic relatedness among dengue 3 strains isolated from patients with mild or severe form of dengue fever in French Polynesia. J Gen Virol 74 ( Pt 12): 2765-2770.

756. Chungue E, Marche G, Plichart R, Boutin JP, Roux J (1989) Comparison of immunoglobulin G enzyme-linked immunosorbent assay (IgG-ELISA) and haemagglutination inhibition (HI) test for the detection of dengue antibodies. Prevalence of dengue IgG-ELISA antibodies in Tahiti. Trans R Soc Trop Med Hyg 83: 708-711.

757. Chutinimitkul S, Payungporn S, Theamboonlers A, Poovorawan Y (2005) Dengue typing assay based on real-time PCR using SYBR green I. J Virol Methods 129: 8-15.

758. Cisneros A, Diaz-Badillo A, Cruz-Martinez G, Tovar R, Ramirez-Palacios LR, et al. (2006) Dengue 2 genotypes in the state of Oaxaca, Mexico. Arch Virol 151: 113-125.

759. Clark DV, Mammen MP, Nisalak A, Puthimethee V, Endy TP (2005) Economic impact of dengue fever/dengue hemorrhagic fever in Thailand at the family and population levels. Am J Trop Med Hyg 72: 786-791.

760. Clyde K, Harris E (2006) RNA secondary structure in the coding region of dengue virus type 2 directs translation start codon selection and is required for viral replication. J Virol 80: 2170-2182.

761. Cobelens FG, Groen J, Osterhaus AD, Leentvaar-Kuipers A, Wertheim-van Dillen PM, et al. (2002) Incidence and risk factors of probable dengue virus infection among Dutch travellers to Asia. Trop Med Int Health 7: 331-338.

762. Cobra C, Rigau-Perez JG, Kuno G, Vorndam V (1995) Symptoms of dengue fever in relation to host immunologic response and virus serotype, Puerto Rico, 1990-1991. Am J Epidemiol 142: 1204-1211.

763. Coder D (1997) DENGUE - VENEZUELA (12). El Nacional; Caracas, Venezuela; 29 Nov 1997 ed. Caracas: PROMED.

764. Coder D (1997) DENGUE, HEMORRHAGIC - COSTA RICA. In: I.G H, editor: PROMED.

765. Coder D (1997) DENGUE - VENEZUELA (CARACAS) (04). In: ProMED-mail, editor. El Universal, Caracas, Venezuela, 22 September 1997 ed. Caracas: PROMED.

766. Coder D (1997) DENGUE - VENEZUELA (CARACAS) (05). Newspaper, Caracas 25 September 1997 ed. Caracas: PROMED.

767. Coder D (1997) DENGUE - VENEZUELA (CARACAS). PROMED.

768. Coder D (1997) DENGUE - VENEZUELA (CARACAS) (03). PROMED.

769. Coder D (1997) DENGUE - COLOMBIA. El Tiempo of Bogota, 21 July 1997 ed: PROMED.

770. Coder D (1997) DENGUE/DENGUE HEMORRHAGIC FEVER - VENEZUELA: UPDATE. Excerpted from material by Ernesto Ecarri Hung in El Universal, Caracas; <<http://www.el-universal.com/1997/03/11/11102A.htm>> ed: PROMED.

771. Coder D (1997) DENGUE/DHF - CUBA (11). In: ProMED-mail, editor. Cuba Press,

<http://www.cubafreepress.org/art/cubap971105aa.html> ed: PROMED.

772. Coder D (1997) DENGUE - VENEZUELA (07). El Universal, Caracas, Venezuela, 17 Nov 97 ed. Caracas: PROMED.

773. Coder D (1997) DENGUE - VENEZUELA (09). Source: Nacional, Caracas, Venezuela; 23 Nov 97 ed: PROMED.

774. Coder D (1997) DENGUE - VENEZUELA (10). Source: El Nacional, Caracas, Venezuela, 25 Nov 1997 ed: PROMED.

775. Coelho GE, Burattini MN, Teixeira MD, Coutinho FAB, Massad E (2008) Dynamics of the 2006/2007 dengue outbreak in Brazil. Mem Inst Oswaldo Cruz 103: 535-U537.

776. Coelho GR, Valenca JT, Rocha TDS, Viana CFG, Goncalves BPA, et al. (2007) Classical dengue fever after liver transplantation. Liver Transpl 13: S97-S97.

777. Colbert JA, Gordon A, Roxelin R, Silva S, Silva J, et al. (2007) Ultrasound measurement of gallbladder wall thickening as a diagnostic test and prognostic indicator for severe dengue in pediatric patients. Pediatr Infect Dis J 26: 850-852.

778. Cologna R, Armstrong PM, Rico-Hesse R (2005) Selection for virulent dengue viruses occurs in humans and mosquitoes. J Virol 79: 853-859.

779. Comach G (1998) DENGUE/DHF - VENEZUELA (ARAGUA). In: (BIOMED) CdIB, editor. Carabobo (Venezuela): PROMED.

780. Congpuong K, Chuchan S, Kwangthong S, Kangchaingone Y, Darakapong A (2008) Appropriate laboratory tests for the diagnosis of dengue infection at a general hospital in Southern Thailand. Asian Biomedicine 2: 289-295.

781. Cook S, Diallo M, Sall AA, Cooper A, Holmes EC (2005) Mitochondrial markers for molecular identification of aedes mosquitoes (Diptera : Culicidae) involved in transmission of arboviral disease in west Africa. J Med Entomol 42: 19-28.

782. Cordeiro MT, Schatzmayr HG, Nogueira RMR, de Oliveira VF, de Melo WT, et al. (2007) Dengue and dengue hemorrhagic fever in the State of Pernambuco, 1995-2006. Rev Soc Bras Med Trop 40: 605-611.

783. Cordeiro MT, Silva AM, Brito CAA, Nascimento EJM, Magalhaes MCF, et al. (2007) Characterization of a dengue patient cohort in Recife, Brazil. Am J Trop Med Hyg 77: 1128-1134.

784. Correa PRL, Franca E, Bogutchi TF (2005) Infestação pelo Aedes aegypti e ocorrência da dengue em Belo Horizonte, Minas Gerais. Rev Saude Publica 39: 33-40.

785. Cortes LM, Barth OM, Pantoja JR, Alves CR (2003) Comparative immunological recognition of proteins from New Guinea "C" dengue virus type 2 prototype and from a dengue virus type 2 strain isolated in the State of Rio de Janeiro, Brazil. Mem Inst Oswaldo Cruz 98: 331-334.

786. Corwin AL, Larasati RP, Bangs MJ, Wuryadi S, Arjoso S, et al. (2001) Epidemic dengue transmission in southern Sumatra, Indonesia. Trans R Soc Trop Med Hyg 95: 257-265.

787. Cosgriff M (2000) DENGUE, DECLINING - MALAYSIA. The Star Online, 14 Apr 2000 [edited] ed: PROMED.

788. Cosgriff M (2000) DENGUE/DHF: UPDATES, 6 DEC 2000 [Palau-Micronesia]. In: ProMED-mail, editor. AFP Report, Mon, 4 Dec 12:43 PM SGT [edited] ed: PROMED.

789. Cosgriff M (2000) DENGUE - SINGAPORE: MOSQUITO BREEDING PLACES. In: ProMED-mail, editor: PROMED.

790. Cosgriff M (2000) DENGUE/DHF - INDONESIA (JAKARTA). In: ProMED-mail, editor. Jakarta Post, 3 Mar 2000 ed: PROMED.

791. Cosgriff M (2000) DENGUE/DHF -INDONESIA (JAKARTA) (02). Jakarta Post, 7 Mar 2000 ed: PROMED.

792. Cosgriff M (2000) DENGUE/DHF: UPDATES, 15 NOV 2000 [Malaysia]. In: ProMED-mail, editor. The Star Online (Kuala Lampur), Tue 14 Nov 2000 [edited] ed: PROMED.

793. Cosgriff M, Jelinek J (2000) DENGUE/DHF: UPDATES, 30 SEP 2000 [India/Dominican Republic]. In: ProMED-mail, editor: PROMED.

794. Costa de Leona L, Estevez J, Monsalve de Castillo F, Callejas D, Manuel Echevarria J (2004) Diagnóstico de laboratorio de pacientes con síndromes febriles exantemáticas o que ocurren en el Estado Zulia, Venezuela, durante el año 1998. Rev Med Chil 132: 1078-1084.

795. Costa SM, Freire MS, Alves AM (2005) DNA vaccine against the non-structural 1 protein (NS1) of dengue 2 virus. Vaccine.

796. Costa SM, Paes MV, Barreto DF, Pinhao AT, Barth OM, et al. (2006) Protection against dengue type 2 virus induced in mice immunized with a DNA plasmid encoding the non-structural 1 (NS1) gene fused to the tissue plasminogen activator signal sequence. Vaccine 24: 195-205.

797. Crabtree MB, Kinney RM, Miller BR (2005) Deglycosylation of the NS1 protein of dengue 2 virus, strain 16681: Construction and characterization of mutant viruses. Arch Virol 150: 771-786.

798. Craig S, Thu HM, Lowry K, Wang XF, Holmes EC, et al. (2003) Diverse dengue type 2 virus populations contain recombinant and both parental viruses in a single mosquito host. J Virol 77: 4463-4467.

799. Crowder NB (1997) DENGUE - HONDURAS. In: Traveler NBCELA, editor. Phoenix, AZ: PROMED.

800. Cuddehe M (2009) Mexico fights rise in dengue fever. Lancet 374: 602.

801. Cummings DA, Irizarry RA, Huang NE, Endy TP, Nisalak A, et al. (2004) Travelling waves in the occurrence of dengue haemorrhagic fever in Thailand. Nature 427: 344-347.

802. Cunha BA, Johnson D, McDermott B (2009) Atypical Dengue Fever Mimicking Typhoid Fever in a College Student Traveler. Am J Med 122: E1-E3.

803. Cunha RV, Schatzmayr HG, Miagostovich MP, Barbosa AM, Paiva FG, et al. (1999) Dengue epidemic in the State of Rio Grande do Norte, Brazil, in 1997. Trans R Soc Trop Med Hyg 93: 247-249.

804. da Costa AI, Natal D (1998) [Geographical distribution of dengue and socioeconomic factors in an urban locality in southeastern Brazil]. Rev Saude Publica 32: 232-236.

805. da Cunha RV, Dias M, Nogueira RM, Chagas N, Miagostovich MP, et al. (1995) Secondary dengue infection in schoolchildren in a dengue endemic area in the state of Rio de Janeiro, Brazil. Rev Inst Med Trop Sao Paulo 37: 517-521.

806. da Cunha RV, Maspero RC, Miagostovich MP, de Araujo ES, Luz Dda C, et al. (1997) Dengue infection in Paracambi, State of Rio de Janeiro, 1990-1995. Rev Soc Bras Med Trop 30: 379-383.

807. da Cunha RV, Miagostovich MP, Petrola Z, de Araujo ES, Cortez D, et al. (1998) Retrospective study on dengue in Fortaleza, state of Ceara, Brazil. Mem Inst Oswaldo Cruz 93: 155-159.

808. da Cunha S (2000) DENGUE - INDONESIA (EAST TIMOR): COMMENT. PROMED.

809. da Silva LJ (1998) DENGUE VIRUSES, TRANSMISSION BY _AEDES ALBOPICTUS_(02) [Brazil]. Campinas: PROMED.

810. da Silva LJ (1999) DENGUE/DHF - BRAZIL (SAO PAULO). In: ProMED-mail, editor. ProMED-PORT promed-port@usa.healthnet.org ed: PROMED.

811. da Silva-Nunes M, de Souza VAF, Pannuti CS, Speranca MA, Terzian ACB, et al. (2008) Risk factors for dengue virus infection in rural Amazonia: Population-based cross-sectional surveys. Am J Trop Med Hyg 79: 485-494.

812. Dantes HG, Koopman JS, Addy CL, Zarate ML, Marin MA, et al. (1988) Dengue epidemics on the Pacific Coast of Mexico. Int J Epidemiol 17: 178-186.

813. Dar L, Broor S, Sengupta S, Xess I, Seth P (1999) The first major outbreak of dengue hemorrhagic fever in Delhi, India. Emerg Infect Dis 5: 589-590.

814. Dar L, Gupta E, Narang P, Broor S (2006) Cocirculation of dengue serotypes, Delhi, India, 2003. Emerg Infect Dis 12: 352-353.

815. D'Arcy A, Chaillet M, Schiering N, Villard F, Lim SP, et al. (2006) Purification and crystallization of dengue and West Nile virus NS2B-NS3 complexes. Acta Crystallogr, Sect F: Struct Biol Cryst Commun 62: 157-162.

816. Darcy A, Clothier H, Phillips D, Bakote'e B, Stewart T (2001) Solomon Islands dengue seroprevalence study--previous circulation of dengue confirmed. P N G Med J 44: 43-47.

817. Das S, Pingle MR, Munoz-Jordan J, Rundell MS, Rondini S, et al. (2008) Detection and serotyping of dengue virus in serum samples by multiplex reverse transcriptase PCR-ligase detection reaction assay. J Clin Microbiol 46: 3276-3284.

818. Dash PK, Parida MM, Saxena P, Abhyankar A, Singh CP, et al. (2006) Reemergence of dengue virus type-3 (subtype-III) in India: Implications for increased incidence of DHF & DSS. Virol J 3: -.

819. Dash PK, Parida MM, Saxena P, Kumar M, Rai A, et al. (2004) Emergence and continued circulation of dengue-2 (genotype IV) virus strains in northern India. J Med Virol 74: 314-322.

820. Dash PK, Saxena P, Abhyankar A, Bhargava R, Jana AM (2005) Emergence of dengue virus type-3 in northern India. Southeast Asian J Trop Med Public Health 36: 370-377.

821. Davis JS, Bourke P (2004) Rhabdomyolysis associated with dengue virus infection. Clin Infect Dis 38: E109-E111.

822. De Alwis R (2005) DENGUE/DHF UPDATE 2005 (16). PROMED.

823. de Amorim Garcia CA, Gomes AH, de Oliveira AG (2006) Bilateral stellar neuroretinitis in a patient with dengue fever. Eye 20: 1382-1383.

824. de Araujo JMG, Bello G, Schatzmayr HG, dos Santos FB, Nogueira RMR (2009) Dengue virus type 3 in Brazil: a phylogenetic perspective. Mem Inst Oswaldo Cruz 104: 526-529.

825. de Araujo JMG, Schatzmayr HG, de Filippis AMB, dos Santos FB, Cardoso MA, et al. (2009) A retrospective survey of dengue virus infection in fatal cases from an epidemic in Brazil. J Virol Methods 155: 34-38.

826. de Araujo TP, Rodrigues SG, Costa MI, Vasconcelos PF, da Rosa AP (2002) Diagnóstico sorológico de infecções por dengue e febre amarela em casos suspeitos no Estado do Pará, Brasil, 1999. Rev Soc Bras Med Trop 35: 579-584.

827. de Castro JA, de Andrade HM, do Monte SJ, da Silva AS, Gomes KC, et al. (2003) Dengue viruses activity in Piaui, Brazil. Mem Inst Oswaldo Cruz 98: 1021-1023.

828. De Castro RAC, De Castro JAA, Barez MYC, Frias MV, Dixit J, et al. (2007) Thrombocytopenia associated with dengue hemorrhagic fever responds to intravenous administration of anti-D (Rho-D) immune globulin. Am J Trop Med Hyg 76: 737-742.

829. de Figueiredo RM, Naveca FG, Bastos MD, Melo MD, Viana SD, et al. (2008) Dengue virus type 4, Manaus, Brazil. Emerg Infect Dis 14: 667-669.

830. De Figueiredo RM, Thatcher BD, de Lima ML, Almeida TC, Alecrim WD, et al. (2004) Doenças exantemáticas e primeira epidemia de dengue que ocorrem em Manaus, Amazonas, Brasil, durante 1998-1999. Rev Soc Bras Med Trop 37: 476-479.

831. De Jesus M, Gubler DJ, Sather GE (1982) A clinical survey of reported cases of dengue-like illness during the outbreak of dengue 1981 in Puerto Rico. Boletín de la Asociación Médica de Puerto Rico 74: 76-78.

832. de la CSB, Garcia G, Perez AB, Morier L, Alvarez M, et al. (2006) Ethnicity and difference in dengue virus-specific memory T cell responses in Cuban individuals. Viral Immunol 19: 662-668.

833. de la CSB, Kouri G, Guzman MG (2007) Race: a risk factor for dengue hemorrhagic fever. Arch Virol 152: 533-542.

834. de Lavaissiere M, D'Ortenzio E, Dussart P, Fontanella JM, Djossou F, et al. (2008) Febrile illness at the emergency department of Cayenne Hospital, French Guiana. Trans R Soc Trop Med Hyg 102: 1055-1057.

835. de Mattos Almeida MC, Caiaffa WT, Assuncao RM, Proietti FA (2007) Spatial vulnerability to dengue in a Brazilian urban area during a 7-year surveillance. J Urban Health 84: 334-345.

836. de Mello MT (2008) Dengue e febre amarela: passado, presente e futuro no Rio de Janeiro. Rev Bras Med Vet 30: 202-203.

837. de Melo PRS, Reis EAG, Ciuffo IA, Goes M, Blanton RE, et al. (2007) The dynamics of dengue virus serotype 3 introduction and dispersion in the state of Bahia, Brazil. Mem Inst Oswaldo Cruz 102: 905-912.

838. de Oliveira Poersch C, Pavoni DP, Queiroz MH, de Borba L, Goldenberg S, et al. (2005) Dengue virus infections: comparison of methods for diagnosing the acute disease. J Clin Virol 32: 272-277.

839. de Oliveira RL, Vazeille M, de Filippis AMB, Failloux AB (2003) Large genetic differentiation and low variation in vector competence for dengue and yellow fever viruses of Aedes albopictus from Brazil, the United States, and the Cayman Islands. Am J Trop Med Hyg 69: 105-114.

840. de Oliveira RM (1998) Dengue no Rio de Janeiro: repensando a participação popular em saúde. Cad Saude Publica 14 Suppl 2: 69-78.

841. de Oliveira RM, Valla VV (2001) As condições e as experiências de vida de grupos populares no Rio de Janeiro: repensando a mobilização popular no controle do dengue. Cad Saude Publica 17 Suppl: 77-88.

842. de Oliveira SA, Camacho LAB, Bruno LF, de Gusmao RC, Pereira ACDM, et al. (2009) Acute arthropathy in patients with rash diseases: a comparative study. Clin Rheumatol 28: 1067-1071.

843. de Ory F, Sanz JC, Echevarria JE, Mosquera MD, Guisasola ME, et al. (2004) Comparison of serological procedures used for the diagnosis of viral exanthema in laboratories participating in the measles elimination plan. Enferm Infecc Microbiol Clin 22: 319-322.

844. De Paula SO, De Melo Lima C, Torres MP, Pereira MRG, da Fonseca BAL (2004) One-step RT-PCR protocols improve the rate of dengue diagnosis compared to two-step RT-PCR approaches. J Clin Virol 30: 297-301.

845. De Paula SO, Pires Neto RJ, Correa JA, Assumpcao SR, Costa ML, et al. (2002) The use of reverse transcription-polymerase chain reaction (RT-PCR) for the rapid detection and identification of dengue virus in an endemic region: a validation study. Trans R Soc Trop Med Hyg 96: 266-269.

846. De Rivera IL, Parham L, Murillo W, Moncada W, Vazquez S (2008) Humoral immune response of dengue hemorrhagic fever cases in children from Tegucigalpa, Honduras. Am J Trop Med Hyg 79: 262-266.

847. de Silva AM, Dittus WP, Amerasinghe PH, Amerasinghe FP (1999) Serologic evidence for an epizootic dengue virus infecting toque macaques (Macaca sinica) at Polonnaruwa, Sri Lanka. Am J Trop Med Hyg 60: 300-306.

848. De Simone TS, Nogueira RM, Araujo ES, Guimaraes FR, Santos FB, et al. (2004) Dengue virus surveillance: the co-circulation of DENV-1, DENV-2 and DENV-3 in the State of Rio de Janeiro, Brazil. Trans R Soc Trop Med Hyg 98: 553-562.

849. de Souza LJ, Martins AL, Paravidini PC, Nogueira RM, Gicovate Neto C, et al. (2005) Hemorrhagic encephalopathy in dengue shock syndrome: a case report. Braz J Infect Dis 9: 257-261.

850. de Souza LJ, Nogueira RMR, Soares LC, Soares CEC, Ribas BF, et al. (2007) The impact of dengue on liver function as evaluated by aminotransferase levels. Braz J Infect Dis 11: 407-410.

851. de Souza LJ, Reis AFF, de Almeida FCR, de Souza LA, Abukater M, et al. (2008) Alteration in the Erythrocyte Sedimentation Rate in Dengue Patients: Analysis of 1,398 Cases. Braz J Infect Dis 12: 472-475.

852. de Souza VAUF, Fernandes S, Araujo ES, Tateno AF, Oliveira OMNPF, et al. (2004) Use of an immunoglobulin G avidity test to discriminate between primary and secondary dengue virus infections. J Clin Microbiol 42: 1782-1784.

853. de Souza VAUF, Tateno AF, Oliveira RR, Domingues RB, Araujo ES, et al. (2007) Sensitivity and specificity of three ELISA-based assays for discriminating primary from secondary acute dengue virus infection. J Clin Virol 39: 230-233.

854. de Thoisy B, Dussart P, Kazanji M (2004) Wild terrestrial rainforest mammals as potential reservoirs for flaviviruses (yellow fever, dengue 2 and St Louis encephalitis viruses) in French Guiana. Trans R Soc Trop Med Hyg 98: 409-412.

855. de Thoisy B, Lacoste V, Germain A, Munoz-Jordan J, Colon C, et al. (2009) Dengue Infection in Neotropical Forest Mammals. Vector-Borne and Zoonotic Diseases 9: 157-169.

856. de Wet N, Ye W, Hales S, Warrick R, Woodward A, et al. (2001) Use of a computer model to identify potential hotspots for dengue fever in New Zealand. N Z Med J 114: 420-422.

857. Dechant EJ, Rigau-Perez JG (1999) Hospitalizations for suspected dengue in Puerto Rico, 1991-1995: estimation by capture-recapture methods. The Puerto Rico Association of Epidemiologists. Am J Trop Med Hyg 61: 574-578.

858. Deen JL, Harris E, Wills B, Balmaseda A, Hammond SN, et al. (2006) The WHO dengue classification and case definitions: time for a reassessment. Lancet 368: 170-173.

859. Deepak NA, Patel ND (2006) Differential diagnosis of acute liver failure in India. Ann Hepatol 5: 150-156.

860. Degallier N, da Rosa AP, Vasconcelos PF, Figueiredo LT, da Rosa JF, et al. (1996) Dengue e seus vetores no Brasil. Bull Soc Pathol Exot Filiales 89: 128-135; 136.

861. Degallier N, Favier C, Boulanger JP, Menkes C (2009) Imported and autochthonous cases in the dynamics of dengue epidemics in Brazil. Rev Saude Publica 43: -.

862. Degallier N, Herve JP, Travassos da Rosa AP, Sa GC (1988) [Aedes aegypti (L.): importance of its bioecology in the transmission of dengue and other arboviruses. I]. Bull Soc Pathol Exot Filiales 81: 97-110.

863. Degallier N, Teixeira JM, Soares Sd Sda S, Pereira RD, Pinto SC, et al. (2003) Aedes albopictus may not be vector of dengue virus in human epidemics in Brazil. Rev Saude Publica 37: 386-387.

864. Degallier N, Teixeira JM, Vilarinhos PD, Pinto SC, Pereira RD (2000) First isolation of dengue 1 virus from Aedes aegypti in Federal District, Brazil. Rev Soc Bras Med Trop 33: 95-96.

865. Degallier N, Vilarinhos PT, de Carvalho MS, Knox MB, Caetano J, Jr. (2000) People's knowledge and practice about dengue, its vectors, and control means in Brasilia (DF), Brazil: its relevance with entomological factors. J Am Mosq Control Assoc 16: 114-123.

866. Delgado MJ, Gutierrez JM, Radic LB, Maretic T, Zekan S, et al. (2008) Imported dengue hemorrhagic fever, Europe. Emerg Infect Dis 14: 1329-1330.

867. Delgado S, Erickson BR, Agudo R, Blair PJ, Vallejo E, et al. (2008) Chapare virus, a newly discovered arenavirus isolated from a fatal hemorrhagic fever case in Bolivia. PLoS Pathog 4: e1000047.

868. Deparis X, Chungue E, Pauck S, Roche C, Murgue B, et al. (1998) Surveillance épidémiologique specifique de la dengue: Méthode et intérêt lors de l'épidémie de dengue 2 en Polynésie française en 1996. Trop Med Int Health 3: 566-570.

869. Deparis X, Murgue B, Roche C, Cassar O, Chungue E (1998) Changing clinical and biological manifestations of dengue during the dengue-2 epidemic in French Polynesia in 1996/97--description and analysis in a prospective study. Trop Med Int Health 3: 859-865.

870. Deparis X, Roche C, Murgue B, Chungue E (1998) Possible dengue sequential infection: dengue spread in a neighbourhood during the 1996/97 dengue-2 epidemic in French Polynesia. Trop Med Int Health 3: 866-871.

871. Depradine C, Lovell E (2004) Climatological variables and the incidence of Dengue fever in Barbados. Int J Environ Health Res 14: 429-441.

872. DeRoeck D, Deen J, Clemens JD (2003) Policymakers' views on dengue fever/dengue haemorrhagic fever and the need for dengue vaccines in four southeast Asian countries. Vaccine 22: 121-129.

873. Derouich M, Boutayeb A (2006) Dengue fever: Mathematical modelling and computer simulation. Applied Mathematics and Computation 177: 528-544.

874. Descloux E, Cao-Lormeau VM, Roche C, De Lamballerie X (2009) Dengue 1 diversity and microevolution, French polynesia 2001-2006: connection with epidemiology and clinics. PLoS Negl Trop Dis 3: e493.

875. Dhooria GS, Bhat D, Bains HS (2008) Clinical profile and outcome in children of dengue hemorrhagic fever in North India. Iranian Journal of Pediatrics 18: 222-228.

876. Diallo M, Ba Y, Sall AA, Diop OM, Ndione JA, et al. (2003) Amplification of the sylvatic cycle of dengue virus type 2, Senegal, 1999-2000: entomologic findings and epidemiologic considerations. Emerg Infect Dis 9: 362-367.

877. Diallo M, Sall AA, Moncayo AC, Ba Y, Fernandez Z, et al. (2005) Potential role of sylvatic and domestic African mosquito species in dengue emergence. Am J Trop Med Hyg 73: 445-449.

878. Dias J, Pedral-Sampaio DB, Jones TC (1997) Aedes aegypti Surveillance and Correlation with the Occurrence of Dengue Fever in Bahia, Brazil. Braz J Infect Dis 1: 36-41.

879. Diaz FJ, Black WCt, Farfan-Ale JA, Lorono-Pino MA, Olson KE, et al. (2006) Dengue virus circulation and evolution in Mexico: a phylogenetic perspective. Arch Med Res 37: 760-773.

880. Diaz-Quijano F, Gonzalez-Rangel A, Gomez-Capacho A, Espindola-Gomez R, Martinez-Vega R, et al. (2008) Pluviosidad como Predictor de Consulta por Síndrome Febril Agudo en un Área. Revista de Salud Pública (Bogotá, Colombia) 10: 250-259.

881. Diaz-Quijano FA (2008) Predictors of spontaneous bleeding in dengue patients: a systematic review of the literature. Invest Clin 49: 111-122.

882. Diaz-Quijano FA, Martinez-Vega RA, Villar-Centeno LA (2005) Los primeros indicadores de la gravedad de la infección por el virus del dengue. Enferm Infecc Microbiol Clin 23: 529-532.

883. Diaz-Quijano FA, Martinez-Vega RA, Villar-Centeno LA (2008) Early predictors of haemorrhage in acute febrile syndrome patients from Bucaramanga, Colombia: a dengue endemic area. Singapore Med J 49: 480-482.

884. Diaz-Quijano FA, Villar-Centeno LA, Martinez-Vega RA (2005) Eficacia de la administración de dipirona principios de la severidad de la infección por el virus del dengue en una cohorte prospectiva. Enferm Infecc Microbiol Clin 23: 593-597.

885. Diaz-Quijano FA, Villar-Centeno LA, Martinez-Vega RA (2006) Complicaciones asociadas a la trombocitopenia profunda en pacientes con dengue Rev Med Chil 134: 167-173.

886. Dieng H, Boots M, Tuno N, Tsuda Y, Takagi M (2002) A laboratory and field evaluation of Macrocyclops distinctus, Megacyclops viridis and Mesocyclops pehpeiensis as control agents of the dengue vector Aedes albopictus in a peridomestic area in Nagasaki, Japan. Med Vet Entomol 16: 285-291.

887. Dietz V, Gubler DJ, Ortiz S, Kuno G, Casta-Velez A, et al. (1996) The 1986 dengue and dengue hemorrhagic fever epidemic in Puerto Rico: epidemiologic and clinical observations. P R Health Sci J 15: 201-210.

888. Dietz VJ, Gubler DJ, Rigau-Perez JG, Pinheiro F, Schatzmayr HG, et al. (1990) Epidemic dengue 1 in Brazil, 1986: evaluation of a clinically based dengue surveillance system. Am J Epidemiol 131: 693-701.

889. Dinesh N, Patil VD (2006) Persistent thrombocytopenia after Dengue hemorrhagic fever. Indian Pediatr 43: 1010-1011.

890. Djojodibroto RD, Santoso B, Ngatidjan, Soetrisno U (1978) Serum cholinesterase activity in patients with dengue haemorrhagic fever. Trop Geogr Med 30: 351-353.

891. Dobler GF, M.; Essbauer S.; et al. Antibody prevalence against arboviruses in humans in

Northern Afghanistan; 2007; Vienna. pp. 23-25.

892. Domingo C, de Ory F, Sanz JC, Reyes N, Gascon J, et al. (2009) Molecular and serologic markers of acute dengue infection in naive and flavivirus-vaccinated travelers. Diagn Microbiol Infect Dis 65: 42-48.

893. Domingo C, Palacios G, Jabado O, Reyes N, Niedrig M, et al. (2006) Use of a short fragment of the C-terminal e gene for detection and characterization of two new lineages of dengue virus 1 in India. J Clin Microbiol 44: 1519-1529.

894. Domingues RB, Kuster GW, Onuki de Castro FL, Souza VA, Levi JE, et al. (2006) Headache features in patients with dengue virus infection. Cephalalgia 26: 879-882.

895. Domingues RB, Kuster GW, Onuki-Castro FL, Souza VA, Levi JE, et al. (2008) Involvement of the central nervous system in patients with dengue virus infection. J Neurol Sci 267: 36-40.

896. Donalisio MR, Alves MJ, Visockas A (2001) Inquérito sobre conhecimentos e atitudes da população sobre a transmissão do dengue - região de Campinas São Paulo, Brasil - 1998. Rev Soc Bras Med Trop 34: 197-201.

897. dos Santos CN, Rocha CF, Cordeiro M, Fragoso SP, Rey F, et al. (2002) Genome analysis of dengue type-1 virus isolated between 1990 and 2001 in Brazil reveals a remarkable conservation of the structural proteins but amino acid differences in the non-structural proteins. Virus Res 90: 197-205.

898. dos Santos FB, Miagostovich MP, Nogueira RM, Edgil D, Schatzmayr HG, et al. (2002) Complete nucleotide sequence analysis of a Brazilian dengue virus type 2 strain. Mem Inst Oswaldo Cruz 97: 991-995.

899. Dos Santos FB, Miagostovich MP, Nogueira RMR, Schatzmayr HG, Riley LW, et al. (2004) Analysis of recombinant dengue virus polypeptides for dengue diagnosis and evaluation of the humoral immune response. Am J Trop Med Hyg 71: 144-152.

900. dos Santos HWG, Poloni TRRS, Souza KP, Muller VDM, Tremeschin F, et al. (2008) A simple one-step real-time RT-PCR for diagnosis of dengue virus infection. J Med Virol 80: 1426-1433.

901. dos Santos LU, de Andrade CF (1997) Survey of cyclopids (Crustacea, Copepoda) in Brazil and preliminary screening of their potential as dengue vector predators. Rev Saude Publica 31: 221-226.

902. Dos Santos R, Vendramini V, Mattos N, Kanamura C, de Carvalho LV (2008) Dengue hemorragic fever in brazil: histopathological findings in two fatal cases. Histopathology 53: 3-4.

903. Drinkhall J (1998) DENGUE - FIJI (06). Radio Australia, Melbourne, 0900 GMT 13 Jan 98 ed: PROMED.

904. Duangchanda S, Tanaka M, Morita K, Rojanasuphot S, Igarashi A (1994) Comparative nucleotide and deduced amino acid sequence of the envelope glycoprotein gene among three dengue virus type 2 strains isolated from patients with different disease severities in Maha Sarakham, northeast Thailand. Southeast Asian J Trop Med Public Health 25: 243-251.

905. Duarte HHP, Franca EB (2006) Qualidade dos dados da vigilância epidemiológica da dengue em Belo Horizonte, MG. Rev Saude Publica 40: 134-142.

906. Dudley J (2006) Avian influenza, human (102): Thailand, dengue exposure PROMED: Promed.

907. Dudley J (2006) Dengue/DHF update 2006 (35) PROMED: Promed.

908. Dudley J, Banks A-L (2006) Dengue/DHF update 2006 (38) PROMED: Promed.

909. Dudley J, Banks A-L, Marshall M (2007) Dengue/DHF update 2007 (10) PROMED: Promed.

910. Dudley J, Rodriguez A (2006) Dengue/DHF update 2006 (34) PROMED: Promed.

911. Dudley J, Stevenson E, Silver D, Banks A-L (2007) Dengue/DHF update 2007 (12) PROMED: Promed.

912. Duffy DC (2001) DENGUE - USA (HAWAII) (04). Honolulu Advertiser, Fri 5 Oct 2001 [edited] ed: PROMED.

913. Duffy MR, Chen TH, Hancock WT, Powers AM, Kool JL, et al. (2009) Zika Virus Outbreak on Yap Island, Federated States of Micronesia. N Engl J Med 360: 2536-2543.

914. Dumoulin A, Marti H, Panning M, Hatz C, Hirsch HH (2008) Pan-dengue virus detection by PCR for travelers returning from the tropics. J Clin Microbiol 46: 3104-3106.

915. Duque JE, Navarro-Silva MA, Trejos DY (2009) Gestão de Simulação de Aedes aegypti (Diptera: Culicidae) e seus efeitos em uma epidemia de dengue. Rev Colomb Entomol 35: 66-72.

916. Durand G (2002) DENGUE/DHF UPDATES (26): 8 JUL 2002 [French Polynesia]. PROMED.

917. Durand JP (2001) DENGUE/DHF - WESTERN PACIFIC REGION (02) [Polynesia]. In: Diagnostics A, editor. Unit of Tropical Virology

Tropical Medicine Institute of the Health Military Service (IMTSSA): PROMED.

918. Durand JP (2004) DENGUE/DHF UPDATE 2004 (03) [French Antilles/ Indonesia/ Tonga]. In: ProMED-mail, editor: PROMED.

919. Durbin AP, Vargas MJ, Wanionek K, Hammond SN, Gordon A, et al. (2008) Phenotyping of peripheral blood mononuclear cells during acute dengue illness demonstrates infection and increased activation of monocytes in severe cases compared to classic dengue fever. Virology 376: 429-435.

920. Duseja A, Thumburu KK, Kumar A, Das A, Dhiman RK, et al. (2008) Hepatic dysfunction in dengue fever. J Gastroenterol Hepatol 23: A64-A64.

921. Dussart P, Labeau B, Lagathu G, Louis P, Nunes MRT, et al. (2006) Evaluation of an enzyme immunoassay for detection of dengue virus NS1 antigen in human serum. Clin Vaccine Immunol 13: 1185-1189.

922. Dussart P, Lavergne A, Lagathu G, Lacoste V, Martial J, et al. (2006) Reemergence of dengue virus type 4, French Antilles and French Guiana, 2004-2005. Emerg Infect Dis 12: 1748-1751.

923. Dussart P, Petit L, Labeau B, Bremand L, Leduc A, et al. (2008) Evaluation of Two New Commercial Tests for the Diagnosis of Acute Dengue Virus Infection Using NS1 Antigen Detection in Human Serum. PLoS Negl Trop Dis 2: -.

924. Dutta P, Khan SA, Khan AM, Sharma CK, Mahanta J (2004) Entomological observations on dengue vector mosquitoes following a suspected outbreak of dengue in certain parts of Nagaland with a note on their susceptibility to insecticides. J Environ Biol 25: 209-212.

925. Dutta P, Khan SA, Sharma CK, Doloi P, Hazarika NC, et al. (1998) Distribution of potential dengue vectors in major townships along the national highways and trunk roads of northeast India. Southeast Asian J Trop Med Public Health 29: 173-176.

926. Dwork KG (1964) Dengue in New York City. NY State J Med 64: 543-545.

927. Eamchan P, Nisalak A, Foy HM, Chareonsook OA (1989) Epidemiology and control of dengue virus infections in Thai villages in 1987. Am J Trop Med Hyg 41: 95-101.

928. Earl PR (1996) DENGUE - NUEVA LEON, MEXICO. Nuevo Leon, Mexico: PROMED.

929. Eckels KH, Dubois DR, Putnak R, Vaughn DW, Innis BL, et al. (2003) Modification of dengue virus strains by passage in primary dog kidney cells: Preparation of candidate vaccines and immunization of monkeys. Am J Trop Med Hyg 69: 12-16.

930. Edelman R, Schneider RJ, Chieowanich P, Pornpibul R, Voodhikul P (1975) The effect of dengue virus infection on the clinical sequelae of Japanese encephalitis: a one year follow-up study in Thailand. Southeast Asian J Trop Med Public Health 6: 308-315.

931. Edelman R, Wasserman SS, Bodison SA, Putnak RJ, Eckels KH, et al. (2003) Phase I trial of 16 formulations of a tetravalent live-attenuated dengue vaccine. Am J Trop Med Hyg 69: 48-60.

932. Edgil D, Diamond MS, Holden KL, Paranjape SM, Harris E (2003) Translation efficiency determines differences in cellular infection among dengue virus type 2 strains. Virology 317: 275-290.

933. Effler P, Pang L, Kitsutani P, Vorndam V, Nakata M, et al. (2005) Dengue Fever, Hawaii, 2001–2002. Emerg Infect Dis 11: 742-749.

934. Effler PV, Pang L, Kitsutani P, Vorndam V, Nakata M, et al. (2005) Dengue fever, Hawaii, 2001-2002. Emerg Infect Dis 11: 742-749.

935. Egger JR, Coleman PG (2007) Age and clinical dengue illness. Emerg Infect Dis 13: 924-925.

936. Egger JR, Ooi EE, Kelly DW, Woolhouse ME, Davies CR, et al. (2008) Reconstructing historical changes in the force of infection of dengue fever in Singapore: implications for surveillance and control. Bull World Health Organ 86: 187-196.

937. Einecke D (2007) Dengue-Fieber: dramatischer Anstieg der häufigsten hämorrhagisches Fieber. Fatal Mückenstichen. MMW Fortschr Med 149: 10-11.

938. Eisen L, Lozano-Fuentes S (2009) Use of Mapping and Spatial and Space-Time Modeling Approaches in Operational Control of Aedes aegypti and Dengue. PLoS Negl Trop Dis 3: -.

939. Ellis RD, Fukuda MM, McDaniel P, Welch K, Nisalak A, et al. (2006) Causes of fever in adults on the Thai-Myanmar border. Am J Trop Med Hyg 74: 108-113.

940. Ellis T, Imrie A, Katz AR, Effler PV (2008) Underrecognition of leptospirosis during a dengue fever outbreak in Hawaii, 2001-2002. Vector-Borne and Zoonotic Diseases 8: 541-547.

941. Endy TP, Chunsuttiwat S, Nisalak A, Libraty DH, Green S, et al. (2002) Epidemiology of inapparent and symptomatic acute dengue virus infection: a prospective study of primary school children in Kamphaeng Phet, Thailand. Am J Epidemiol 156: 40-51.

942. Endy TP, Nisalak A, Chunsuttitwat S, Vaughn DW, Green S, et al. (2004) Relationship of preexisting dengue virus (DV) neutralizing antibody levels to viremia and severity of disease in a prospective cohort study of DV infection in Thailand. J Infect Dis 189: 990-1000.

943. Endy TP, Nisalak A, Chunsuttiwat S, Libraty DH, Green S, et al. (2002) Spatial and temporal circulation of dengue virus serotypes: a prospective study of primary school children in Kamphaeng Phet, Thailand. Am J Epidemiol 156: 52-59.

944. Dietz V, Gubler DJ, Ortiz S, Kuno G, Casta-Velez A, et al. (1996) The 1986 dengue and dengue hemorrhagic fever epidemic in Puerto Rico: epidemiologic and clinical observations. P R Health Sci J 15: 201-210.

945. Dietz V, Nieburg, P., Gubler, D.J., Gomez, I. (1992) Diagnosis of measles by clinical case definition in dengue-endemic areas: implications for measles surveillance and control. Bull World Health Organ 70: 745-750.

946. Garcia-Rivera EJ, Rigau-Perez JG (2003) Dengue severity in the elderly in Puerto Rico. Rev Panam Salud Publica 13: 362-368.

947. Gubler DJ, Casta-Valez A (1991) A program for prevention and control of epidemic dengue and dengue hemorrhagic fever in Puerto Rico and the U.S. Virgin Islands. Bull Pan Am Health Organ 25: 237-247.

948. Gubler DJ, Kuno, G., Waterman, S.H. (1985) A case of natural concurrent human infection with two dengue viruses. Am J Trop Med Hyg 34: 170-173.

949. Hafkin B, Kaplan JE, Reed C, Elliott LB, Fontaine R, et al. (1982) Reintroduction of dengue fever into the continental United States. I. Dengue surveillance in Texas, 1980. Am J Trop Med Hyg 31: 1222-1228.

950. Halstead SB, Papaevangelou, G. (1980) Transmission of dengue 1 and 2 viruses in Greece in 1928. Am J Trop Med Hyg 29: 635-637.

951. Hayes EB, Gubler, D.J. (1992) Dengue and dengue hemorrhagic fever. Pediatr Infect Dis J 11: 311-317.

952. Keating J (2001) An investigation into the cyclical incidence of dengue fever. Soc Sci Med 53: 1587-1597.

953. Kuno G, Bailey RE (1994) Cytokine responses to dengue infection among Puerto Rican patients. Mem Inst Oswaldo Cruz 89: 179-182.

954. Lopez-Correa RH, Cline BL, Ramirez-Ronda C, Bermudez R, Sather GE, et al. (1978) Dengue fever with hemorrhagic manifestations: a report of three cases from Puerto Rico. Am J Trop Med Hyg 27: 1216-1224.

955. Malison MD, Waterman SH (1983) Dengue fever in the United States. A report of a cluster of imported cases and review of the clinical, epidemiologic, and public health aspects of the disease. the Journal of the American Medical Association JAMA 249: 496-500.

956. Mogi M, Khamboonruang, C., Choochote, W., Suwanpanti, P. (1988) Ovitrap surveys of dengue vector mosquitoes in Chiang Mai, northern Thailand: seasonal shifts in relative abundance of Aedes albopictus and Ae. aegypti. Med Vet Entomol 2: 319-324.

957. Moore CG, Cline, B.L., Ruiz-Tiben, E., Lee, D., Romney-Joseph, H., Rivera-Correa, E. (1978) Aedes aegypti in Puerto Rico: environmental determinants of larval abundance and relation to dengue virus transmission. Am J Trop Med Hyg 27: 1225-1231.

958. Morens DM, Rigau-Perez JG, Lopez-Correa RH, Moore CG, Ruiz-Tiben EE, et al. (1986) Dengue in Puerto Rico, 1977: public health response to characterize and control an epidemic of multiple serotypes. Am J Trop Med Hyg 35: 197-211.

959. O'Leary DR, Rigau-Perez R, Hayes EB, Vorndam AV, Clark GG, et al. (2002) Assessment of dengue risk in relief workers in Puerto Rico after Hurricane Georges, 1998. Am J Trop Med Hyg 66: 35-39.

960. Ramirez-Ronda CH (1987) Dengue in Puerto Rico: clinical manifestations and management from 1960's to 1987. P R Health Sci J 6: 113-118.

961. Rawlings JA, Hendricks KA, Burgess CR, Campman RM, Clark GG, et al. (1998) Dengue surveillance in Texas, 1995. Am J Trop Med Hyg 59: 95-99.

962. Reiter P, Lathrop S, Bunning M, Biggerstaff B, Singer D, et al. (2003) Texas lifestyle limits transmission of dengue virus. Emerg Infect Dis 9: 86-89.

963. Rigau-Perez JG (1997) Clinical manifestations of dengue hemorrhagic fever in Puerto Rico, 1990-1991. Puerto Rico Association of Epidemiologists. Rev Panam Salud Publica 1: 381-388.

964. Rigau-Perez JG (1999) Surveillance for an emerging disease: dengue hemorrhagic fever in Puerto Rico, 1988-1997. Puerto Rico Association of Epidemiologists. P R Health Sci J 18: 337-345.

965. Rigau-Perez JG, Ayala-Lopez A, Vorndam AV, Clark GG (2001) Dengue activity in Puerto Rico during an interepidemic period (1995-1997). Am J Trop Med Hyg 64: 75-83.

966. Rigau-Perez JG, Ayala-Lopez, A., Garcia-Rivera, E.J., Hudson, S.M., Vorndam, V., Reiter, P., Cano, M.P. Clark, G.G. (2002) The reappearance of dengue-3 and a subsequent dengue-4 and dengue-1 epidemic in Puerto Rico in 1998. Am J Trop Med Hyg 67: 355-362.

967. Rigau-Perez JG, Ayuso-Lamadrid A, Wolff DR, Reiter P, Kuno G (1994) Dengue severity throughout seasonal changes in incidence in Puerto Rico, 1989-1992. The Puerto Rico Association of Epidemiologists. Am J Trop Med Hyg 51: 408-415.

968. Rigau-Perez JG, Bonilla GL (1999) An evaluation of modified case definitions for the detection of dengue hemorrhagic fever. Puerto Rico Association of Epidemiologists. P R Health Sci J 18: 347-352.

969. Rigau-Perez JG, Clark GG (1992) Dengue activity in Puerto Rico, 1990. P R Health Sci J 11: 65-68.

970. Rigau-Perez JG, Gubler DJ, Vorndam AV, Clark GG (1994) Dengue surveillance--United States, 1986-1992. MMWR CDC Surveill Summ 43: 7-19.

971. Rigau-Perez JG, Gubler DJ, Vorndam AV, Clark GG (1997) Dengue: A Literature Review and Case Study of Travelers from the United States, 1986-1994. J Travel Med 4: 65-71.

972. Rigau-Perez JG, Millard PS, Walker DR, Deseda CC, Casta-Velez A (1999) A deviation bar chart for detecting dengue outbreaks in Puerto Rico. Am J Public Health 89: 374-378.

973. Rigau-Perez JG, Vorndam AV, Clark GG (2001) The dengue and dengue hemorrhagic fever epidemic in Puerto Rico, 1994-1995. Am J Trop Med Hyg 64: 67-74.

974. Rodrigues-Figueroa L, Rigau-Perez, J.G., Suarez, E.L., Reiter, P. (1995) Risk factors for dengue infection during an outbreak in Yanes, Puerto Rico in 1991. Am J Trop Med Hyg 52: 496-502.

975. Rodrguez-Tan RS, Weir, M. (1998) Dengue: a review. Tex Med 94: 53-59.

976. Von Allmen SD, Lopez-Correa RH, Woodall JP, Morens DM, Chiriboga J, et al. (1979) Epidemic dengue fever in Puerto Rico, 1977: a cost analysis. Am J Trop Med Hyg 28: 1040-1044.

977. Waterman SH, Kuno, G., Gubler, D.J., Sather, G.E. (1985) Low rates of antigen detection and virus isolation from the peripheral blood leukocytes of dengue fever patients. Am J Trop Med Hyg 34: 380-384.

978. Waterman SH, Novak, R.J., Sather, G.E., Bailey, R.E., Rios, I., Gubler, D.J. (1985) Dengue transmission in two Puerto Rican communities in 1982. Am J Trop Med Hyg 34: 625-632.

979. Epstein DB, Hopp M, Nart P, Doyle P, Banks A-L (2006) Dengue/DHF update 2006 (37) PROMED: Promed.

980. Eram S, Setyabudi Y, Sadono TI, Sutrisno DS, Gubler DJ, et al. (1979) Epidemic dengue hemorrhagic fever in rural Indonesia. II. Clinical studies. Am J Trop Med Hyg 28: 711-716.

981. Evans AS, Wells AV, Ramsay F, Drabkin P, Palmer K (1979) Poliomyelitis, rubella, and dengue antibody survey in Barbados. A follow-up study. Int J Epidemiol 8: 235-241.

982. Evans D (1995) DENGUE - AUSTRALIA. (Australia): PROMED.

983. Evans D (1995) DENGUE - COOK ISLANDS & SOUTH PACIFIC. In: Health CDoHS, editor. Commun Dis Intell. Canberra, Australia: PROMED.

984. Fagbami AH, Mataika JU, Shrestha M, Gubler DJ (1995) Dengue type 1 epidemic with haemorrhagic manifestations in Fiji, 1989-90. Bull World Health Organ 73: 291-297.

985. Fagbami AH, Monath TP, Fabiyi A (1977) Dengue virus infections in Nigeria: a survey for antibodies in monkeys and humans. Trans R Soc Trop Med Hyg 71: 60-65.

986. Fagbo S (2004) DENGUE/DHF UPDATE 2004 (31). PROMED.

987. Fagbo S (2005) DENGUE/DHF UPDATE 2005 (19). PROMED.

988. Failloux AB, Darius H, Pasteur N (1995) Genetic differentiation of Aedes aegypti, the vector of dengue virus in French Polynesia. J Am Mosq Control Assoc 11: 457-462.

989. Failloux AB, Vazeille M, Rodhain F (2002) Geographic genetic variation in populations of the dengue virus vector Aedes aegypti. J Mol Evol 55: 653-663.

990. Fajardo P, Monje CA, Lozano G, Realpe O, Hernandez LE (2001) Nociones populares sobre "dengue" y "rompehuesos", de 2 modelos de la enfermedad en Colombia. Rev Panam Salud Publica 10: 161-168.

991. Fakeeh M, Zaki AM (2001) Virologic and serologic surveillance for dengue fever in Jeddah, Saudi Arabia, 1994-1999. Am J Trop Med Hyg 65: 764-767.

992. Falconar AKI (2007) Antibody responses are generated to immunodominant ELK/KLE-type motifs on the nonstructural-1 glycoprotein during live dengue virus infections in mice and humans: Implications for diagnosis, pathogenesis, and vaccine design. Clin Vaccine Immunol 14: 493-504.

993. Falcon-Lezama JA, Ramos C, Zuniga J, Juarez-Palma L, Rangel-Flores H, et al. (2009) HLA class I and II polymorphisms in Mexican Mestizo patients with dengue fever. Acta Trop 112: 193-197.

994. Falkler WA, Jr., Diwan AR, Halstead SB (1975) A lipid inhibitor of dengue virus in human colostrum and milk; with a note on the absence of anti-dengue secretory antibody. Arch Virol 47: 3-10.

995. Fan WF, Yu SR, Cosgriff TM (1989) The reemergence of dengue in China. Rev Infect Dis 11 Suppl 4: S847-853.

996. Fang R, Lo E, Lim TW (1984) The 1982 dengue epidemic in Malaysia: epidemiological, serological and virological aspects. Southeast Asian J Trop Med Public Health 15: 51-58.

997. Fang R, Sinniah M, Kuen LS (1992) Use of dengue blot in dengue diagnosis: the Malaysian experience. Malays J Pathol 14: 117-120.

998. Faridi MMA, Aggarwal A, Kurnar M, Sarafrazul A (2008) Clinical and biochemical profile of dengue haemorrhagic fever in children in Delhi. Trop Doct 38: 28-30.

999. Farnesi LC, Martins AJ, Valle D, Rezende GL (2009) Embryonic development of Aedes aegypti (Diptera: Culicidae): influence of different constant temperatures. Mem Inst Oswaldo Cruz 104: 124-126.

1000. Fatimil LE, Mollah AH, Ahmed S, Rahman M (2003) Vertical transmission of dengue: first case report from Bangladesh. Southeast Asian J Trop Med Public Health 34: 800-803.

1001. Faulde MK, Scharninghausen JJ, Tisch M (2008) Fire fighting truck-based emergency mosquito biolarviciding to prevent outbreaks of malaria and arboviral disease in Kabul, Afghanistan. J Pest Sci 81: 71-77.

1002. Fauran P, Laille M, Moreau JP (1990) Étude sur la transmission verticale du virus de la dengue dans le Pacifique Sud. Bull Soc Pathol Exot Filiales 83: 311-316.

1003. Feres VC, Martelli CM, Turchi MD, Junior JB, Nogueira RM, et al. (2006) Laboratory surveillance of dengue virus in Central Brazil, 1994-2003. J Clin Virol 37: 179-183.

1004. Ferguson NM, Donnelly CA, Anderson RM (1999) Transmission dynamics and epidemiology of dengue: insights from age-stratified sero-prevalence surveys. Phil Trans R Soc Ser B 354: 757-768.

1005. Fernandez R, Rodriguez T, Borbonet F, Vazquez S, Guzman MG, et al. (1994) Estudio de la relación del dengue-el embarazo en un grupo de cubano-madres. Rev Cuba Med Trop 46: 76-78.

1006. Fernandez Z, Moncayo A, Forattini OP, Weaver SC (2004) Susceptibility of urban and rural populations of Aedes albopictus from Sao Paulo State, Brazil, to infection by dengue-1 and -2 viruses. J Med Entomol 41: 961-964.

1007. Fernandez-Mestre MT, Gendzekhadze K, Rivas-Vetencourt P, Layrisse Z (2004) TNF-alpha-308A allele, a possible severity risk factor of hemorrhagic manifestation in dengue fever patients. Tissue Antigens 64: 469-472.

1008. Ferreira BJ, Souza Mde F, Soares Filho AM, Carvalho AA (2009) Evolução histórica dos programas de prevenção e controle da dengue no Brasil. Ciência & Saúde Coletiva 14: 961-972.

1009. Ferreira MLB, Cavalcanti CG, Coelho CA, Mesquita SD (2005) Manifestações neurológicas de dengue: estudo de 41 casos Arq Neuropsiquiatr 63: 488-493.

1010. Ferrero AJL (1999) DENGUE/DHF - VENEZUELA (ZULIA STATE), ARUBA. Maracaibo: PROMED.

1011. Ferrero AJL (1999) DENGUE/DHF - VENEZUELA (ZULIA). Local press & Weekly Epidemiological Bulletins of the MSAS [Health Ministry] ed. Maracaibo: PROMED.

1012. Figueiredo LB, Cecilio AB, Ferreira GP, Drumond BP, de Oliveira JG, et al. (2008) Dengue virus 3 genotype I associated with dengue fever and dengue hemorrhagic fever, Brazil. Emerg Infect Dis 14: 314-316.

1013. Figueiredo LT (2006) Febres hemorrágicas virais no Brasil. Rev Soc Bras Med Trop 39: 203-210.

1014. Figueiredo LT, Batista WC, Igarashi A (1997) Detection and identification of dengue virus isolates from Brazil by a simplified reverse transcription-polymerase chain reaction (RT-PCR) method. Rev Inst Med Trop Sao Paulo 39: 79-83.

1015. Figueiredo LT, Cavalcante SM, Simoes MC (1990) Dengue serologic survey of schoolchildren in Rio de Janeiro, Brazil, in 1986 and 1987. Bull Pan Am Health Organ 24: 217-225.

1016. Figueiredo LT, Owa MA, Carlucci RH, dal Fabbro AL, de Mello NV, et al. (1995) Dengue serologic survey in Ribeirao Preto, Sao Paulo, Brazil. Bull Pan Am Health Organ 29: 59-69.

1017. Figueiredo LT, Owa MA, Carlucci RH, de Oliveira L (1992) O diagnóstico laboratorial e sintomas do dengue, durante um surto na região de Ribeirão Preto, SP, Brasil. Rev Inst Med Trop Sao Paulo 34: 121-130.

1018. Figueroa M, Pereira R, Gutierrez H, de Mejia C, Padilla N (1982) Dengue epidemic in Honduras, 1978-1980. Bull Pan Am Health Organ 16: 130-137.

1019. Fink J, Gu F, Ling L, Tolfvenstam T, Olfat F, et al. (2007) Host Gene Expression Profiling of Dengue Virus Infection in Cell Lines and Patients. PLoS Negl Trop Dis 1: -.

1020. Finsterer J, Kongchan K (2006) Severe, persisting, steroid-responsive Dengue myositis. J Clin Virol 35: 426-428.

1021. Fior A, Dussart P, Zeddini A, Djossou F (2007) Shock syndrome and adverse outcome of a case of dengue fever caused by den-2 virus inaugural of 2006 epidemic diseases in French Guiana. Virchows Arch 451: 583-583.

1022. Flannery B, Pereira MM, Velloso LdF, Carvalho CdC, De Codes LG, et al. (2001) Referral pattern of leptospirosis cases during a large urban epidemic of dengue. Am J Trop Med Hyg 65: 657-663.

1023. Flauzino RF, Souza-Santos R, Oliveira RM (2009) Dengue, geoprocessamento e indicadores socioeconômicos e ambientais: um estudo de revisão. Rev Panam Salud Publica 25: 456-461.

1024. Focks DA, Brenner RJ, Hayes J, Daniels E (2000) Transmission thresholds for dengue in terms of Aedes aegypti pupae per person with discussion of their utility in source reduction efforts. Am J Trop Med Hyg 62: 11-18.

1025. Focks DA, Daniels E, Haile DG, Keesling JE (1995) A simulation model of the epidemiology of urban dengue fever: literature analysis, model development, preliminary validation, and samples of simulation results. Am J Trop Med Hyg 53: 489-506.

1026. Fointuna Y (2004) DENGUE/DHF UPDATE 2004 (01) [Indonesia]. In: ProMED-mail, editor. The Jakarta Post 5 Jan 2004 [edited] ed: PROMED.

1027. Fong MY, Koh CL, Lam SK (1998) Molecular epidemiology of Malaysian dengue 2 viruses isolated over twenty-five years (1968-1993). Res Virol 149: 457-464.

1028. Fong MY, Yusup R, Yusof R, Lam SK (2004) Neurovirulence of four encephalitogenic dengue 3 virus strains isolated in Malaysia (1992-1994) is not attributed to their envelope protein. Trans R Soc Trop Med Hyg 98: 379-381.

1029. Fontenille D, Toto JC (2001) Aedes (Stegomyia) albopictus (Skuse), a potential new Dengue vector in southern Cameroon. Emerg Infect Dis 7: 1066-1067.

1030. Forde A (2000) DENGUE - INDONESIA (EAST TIMOR) (02). Wellington, New Zealand: PROMED.

1031. Fornells LA (2000) DENGUE/DHF - PARAGUAY (02). In: Diseases CftSoEI, editor. Gazeta do Parana, 16 Apr 2000 ed. Rio de Janeiro: PROMED.

1032. Fornells LA (2000) DENGUE - PARAGUAY, BRAZIL (PARANA). In: ProMED-mail, editor. Gazeta do Parana newspaper, Brazil Mon 27 Mar 2000 [in Portuguese] ed: PROMED.

1033. Fornells LA (2001) DENGUE - USA (HAWAII) (07). In: Diseases CftSoEI, editor. Honolulu Advertiser, Wed 21 Nov 2001 [edited] ed. Rio de Janeiro: PROMED.

1034. Fornells LA (2001) DENGUE/DHF UPDATES (13): 4 OCT 2001[Hong Kong/Nicaragua/Hawaii/Venezuela]. Brasil Online Wed 3 Oct 2001 13:48:48 (Reuters) [translated & edited by JW] ed. Rio de Janeiro: PROMED.

1035. Fornells LA (2002) DENGUE/DHF UPDATES (20): 27 MAY 2002 [Brazil/ Thailand/ Australia]. In: ProMED-mail, editor. Tribuna Digital, O Globo 21 May 2002, (in Portuguese, translated by

JW) [edited] ed: PROMED.

1036. Fornells LA (2004) DENGUE/DHF UPDATE 2004 (31) [Brazil]. In: ProMED-mail, editor. Diario do Nordeste 2 Oct 2004 [in Portuguese, summarized by Mod.JW;

edited] ed: PROMED.

1037. Fornells LA (2004) DENGUE/DHF UPDATE 2004 (31). PROMED.

1038. Fornells LA (2004) DENGUE/DHF UPDATE 2005 (01). PROMED.

1039. Fornells LA (2005) DENGUE/DHF UPDATE 2005 (24). PROMED.

1040. Foster JE, Bennett SN, Carrington CV, Vaughan H, McMillan WO (2004) Phylogeography and molecular evolution of dengue 2 in the Caribbean basin, 1981-2000. Virology 324: 48-59.

1041. Fouque F, Garinci R, Gaborit P (2004) Epidemiological and entomological surveillance of the co-circulation of DEN-I, DEN-2 and DEN-4 viruses in French Guiana. Trop Med Int Health 9: 41-46.

1042. Fouque F, Reynes JM, Moreau JP (1995) Dengue in French Guiana, 1965-1993. Bull Pan Am Health Organ 29: 147-155.

1043. Fouque F, Vazeille M, Mousson L, Gaborit P, Carinci R, et al. (2001) Aedes aegypti in French Guiana: susceptibility to a dengue virus. Trop Med Int Health 6: 76-82.

1044. Fox S (2008) Dengue fever: The next West Nile virus. Infections in Medicine 25: 206-206.

1045. Frank C, Schoneberg I, Krause G, Claus H, Ammon A, et al. (2004) Increase in imported dengue, Germany, 2001-2002. Emerg Infect Dis 10: 903-906.

1046. Franz AWE, Sanchez-Vargas I, Adelman ZN, Blair CD, Beaty BJ, et al. (2006) Engineering RNA interference-based resistance to dengue virus type 2 in genetically modified Aedes aegypti. Proc Natl Acad Sci U S A 103: 4198-4203.

1047. Fraser HS, Wilson WA, Rose E, Thomas EJ, Sissons JG (1978) Dengue fever in Jamaica with shock and hypocomplementaemia, haemorrhagic, visceral and neurological complications. West Indian Med J 27: 106-116.

1048. Fraser HS, Wilson WA, Thomas EJ, Sissons JG (1978) Dengue shock syndrome in Jamaica. Br Med J 1: 893-894.

1049. Fredi AD (2005) Incompleta la vigilancia epidemiológica de la epidemia del dengue-2 en Ibague, Colombia, 1995-1997. - Responder. Biomedica 25: 152; author reply 153.

1050. Freedman DO, Weld LH, Kozarsky PE, Fisk T, Robins R, et al. (2006) Spectrum of disease and relation to place of exposure among ill returned travelers. N Engl J Med 354: 119-130.

1051. Freier JE, Rosen L (1987) Vertical transmission of dengue viruses by mosquitoes of the Aedes scutellaris group. Am J Trop Med Hyg 37: 640-647.

1052. Freire MS, Marchevsky RS, Almeida LFC, Yamamura AMY, Caride EC, et al. (2007) Wild dengue virus types 1, 2 and 3 viremia in rhesus monkeys. Mem Inst Oswaldo Cruz 102: 203-208.

1053. Fruttaldo L, Schettino G, Mongio F, Gatti G, Deambrogio V (2000) A case of dengue from Pune, India. J Travel Med 7: 46-47.

1054. Fuentes O, Lopez R, Marquetti MC, Lugo J (1992) Presence of Aedes (Gymnometopa) mediovittatus in Cuba: a new factor to be considered in the national campaign to eradicate dengue. Bull Pan Am Health Organ 26: 14-17.

1055. Fujita N, Hotta S, Konishi E, Esaki H, Sumarmo, et al. (1997) Dengue hemorrhagic fever in Jakarta, Indonesia in 1988: isolation of dengue virus from patient whole blood using cell cultures. Am J Trop Med Hyg 56: 318-321.

1056. Fukunaga T, Igarashi A, Okuno Y, Ishimine T, Tadano M, et al. (1984) A seroepidemiological study of Japanese encephalitis and dengue virus infections in the Chiang Mai area, Thailand. Biken J 27: 9-17.

1057. Fukunaga T, Okuno Y, Srisupaluck S, Auwanich W, Rojanasuphot S, et al. (1980) Serological and virological studies on patients with dengue hemorrhagic fever (DHF) in Chanthaburi province, Thailand. II. Serological characteristics of viruses isolated from DHF patients using a clone of Singh's Aedes albopictus cells. Biken J 23: 123-133.

1058. Fukunaga T, Okuno Y, Tadano M, Fukai K (1983) A retrospective serological study of Japanese who contracted dengue fever in Thailand. Biken J 26: 67-74.

1059. Fuller DO, Troyo A, Beier JC (2009) El Nino Southern Oscillation and vegetation dynamics as predictors of dengue fever cases in Costa Rica. Environmental Research Letters 4: -.

1060. Gadelha DN, Ventura BV, Ventura LM, Miller MT, Cordeiro MT, et al. (2009) Resposta humoral ao vírus dengue em mães/filhos: relação com a sequência de Möbius. Arq Bras Oftalmol 72: 327-331.

1061. Gagnon SJ, Leporati A, Green S, Kalayanarooj S, Vaughn DW, et al. (2001) T cell receptor Vbeta gene usage in Thai children with dengue virus infection. Am J Trop Med Hyg 64: 41-48.

1062. Galler R, Marchevsky RS, Caride E, Almeida LFC, Yamamura AMY, et al. (2005) Attenuation and immunogenicity of recombinant yellow fever 17D-dengue type 2 virus for rhesus monkeys. Braz J Med Biol Res 38: 1835-1846.

1063. Galli B, Chiaravalloti Neto F (2008) Temporal-spatial risk model to identify areas at high-risk for occurrence of dengue fever. Revista de Salud Pública 42: 656-663.

1064. Gambel JM, Drabick JJ, Swalko MA, Henchal EA, Rossi CA, et al. (1999) Dengue among United Nations mission in Haiti personnel, 1995: implications for preventive medicine. Mil Med 164: 300-302.

1065. Ganesh VK, Muller N, Judge K, Luan CH, Padmanabhan R, et al. (2005) Identification and characterization of nonsubstrate based inhibitors of the essential Dengue and West Nile virus proteases. Bioorg Med Chem 13: 257-264.

1066. Ganeshwaran Y, Seneviratne SM, Jayamaha R, De Silva AP, Balasuriya WK (2001) Dengue fever associated with a haematoma of the rectus abdominis muscle. Ceylon Med J 46: 105-106.

1067. Garcia G, Arango M, Perez AB, Fonte L, Sierra B, et al. (2006) Antibodies from patients with dengue viral infection mediate cellular cytotoxicity. J Clin Virol 37: 53-57.

1068. Garcia G, Perez AB, Sierra B, Aguirre E, Izquierdo A, et al. (2008) Subclass and the FcG Receptor Iia Polymorphism Associate to Dengue Fever, Dengue Hemorrhagic Fever and Asymptomatic Dengue Infection in Cuba. Int J Infect Dis 12: E327-E327.

1069. Garcia JH, Rocha TD, Viana CF, Goncalves BP, Girao ES, et al. (2006) Dengue shock syndrome in a liver transplant recipient. Transplantation 82: 850-851.

1070. Garcia-Cordero J, Ramirez HR, Vazquez-Ochoa M, Gutierrez-Castaneda B, Santos-Argumedo L, et al. (2005) Production and characterization of a monoclonal antibody specific for NS3 protease and the ATPase region of Dengue-2 virus. Hybridoma 24: 160-164.

1071. Garcia-Montalvo BM, Medina F, del Angel RM (2004) La protein binds to NS5 and NS3 and to the 5 ' and 3 ' ends of Dengue 4 virus RNA. Virus Res 102: 141-150.

1072. Garcia-Rivera EJ, Vorndam V, Rigau-Perez JG (2009) Use of an enhanced surveillance system for encephalitis and aseptic meningitis for the detection of neurologic manifestations of dengue in Puerto Rico, 2003. P R Health Sci J 28: 114-120.

1073. Garg P, Nagpal J, Khairnar P, Seneviratne SL (2008) Economic burden of dengue infections in India. Trans R Soc Trop Med Hyg 102: 570-577.

1074. Garg S, Arora R, Kakkar N (2007) Fever and petechiae in a middle aged male. Laboratory Medicine 38: 23-25.

1075. Gasperino J, Yunen J, Guh A, Tanaka KE, Kvetan V, et al. (2007) Fulminant liver failure secondary to haemorrhagic dengue in an international traveller. Liver Int 27: 1148-1151.

1076. George R (1987) Dengue haemorrhagic fever in Malaysia: a review. Southeast Asian J Trop Med Public Health 18: 278-283.

1077. George R, Duraisamy G (1981) Bleeding manifestations of dengue haemorrhagic fever in Malaysia. Acta Trop 38: 71-78.

1078. George R, Lam SK (1997) Dengue virus infection--the Malaysian experience. Ann Acad Med Singapore 26: 815-819.

1079. George R, Liam CK, Chua CT, Lam SK, Pang T, et al. (1988) Unusual clinical manifestations of dengue virus infection. Southeast Asian J Trop Med Public Health 19: 585-590.

1080. Getis A, Morrison AC, Gray K, Scott TW (2003) Characteristics of the spatial pattern of the dengue vector, Aedes aegypti, in Iquitos, Peru. Am J Trop Med Hyg 69: 494-505.

1081. Gianella A (1996) DENGUE SURVEILLANCE, SANTA CRUZ - BOLIVIA. In: CENETROP, editor. (Bolivia): PROMED.

1082. Gianella A, Pirard M, Holzman A, Boelaert M, Fernandez-Ortiz F, et al. (1998) Brote epidémico de dengue, el virus de genotipo 2/Jamaica en Bolivia. Salud Publica Mex 40: 469-473.

1083. Gibbons RV, Kalanarooj S, Jarman RG, Nisalak A, Vaughn DW, et al. (2007) Analysis of repeat hospital admissions for dengue to estimate the frequency of third or fourth dengue infections resulting in admissions and dengue hemorrhagic fever, and serotype sequences. Am J Trop Med Hyg 77: 910-913.

1084. Gil L, Martinez G, Tapanes R, Castro O, Gonzalez D, et al. (2004) Oxidative stress in adult dengue patients. Am J Trop Med Hyg 71: 652-657.

1085. Gill J, Stark LM, Clark GG (2000) Dengue surveillance in Florida, 1997-98. Emerg Infect Dis 6: 30-35.

1086. Gittens-St Hilaire M, Clarke-Greenidge N (2008) An analysis of the subtypes of dengue fever infections in Barbados 2003-2007 by reverse transcriptase polymerase chain reaction. Virol J 5: -.

1087. Glaziou P (1995) DENGUE FEVER IN FRENCH POLYNESIA: UPDATE. In: Malarde I, editor. (Tahiti): PROMED.

1088. Gleeson F, McBride J, Norton R (1999) Culture-amplified detection of dengue virus from serum in an outbreak of dengue fever. J Med Virol 57: 212-215.

1089. Goel A (2006) Dengue fever outbreak in India. Natl Med J India 19: 298-298.

1090. Goh BPK, Tan SG (2006) Case of dengue virus infection presenting with acute acalculous cholecystitis. J Gastroenterol Hepatol 21: 923-924.

1091. Goh KT (1997) Dengue--a re-emerging infectious disease in Singapore. Ann Acad Med Singapore 26: 664-670.

1092. Goh KT, Ng SK, Chan YC, Lim SJ, Chua EC (1987) Epidemiological aspects of an outbreak of dengue fever/dengue haemorrhagic fever in Singapore. Southeast Asian J Trop Med Public Health 18: 295-302.

1093. Goh KT, Yamazaki S (1987) Serological survey on dengue virus infection in Singapore. Trans R Soc Trop Med Hyg 81: 687-689.

1094. Gokhale MD, Jacob PG, Mourya DT (2000) Dengue virus and insecticide susceptibility status of Aedes aegypti mosquitoes from Belagola village, Mandya District, Karnataka state: during and post-epidemic investigations. J Commun Dis 32: 247-253.

1095. Gomber S, Ramachandran VG, Kumar S, Agarwal KN, Gupta P, et al. (2001) Hematological observations as diagnostic markers in dengue hemorrhagic fever--a reappraisal. Indian Pediatr 38: 477-481.

1096. Gomes-Ruiz AC, Nascimento RT, de Paula SO, da Fonseca BAL (2006) SYBR green and TaqMan real-time PCR assays are equivalent for the diagnosis of dengue virus type 3 infections. J Med Virol 78: 760-763.

1097. Gomez-Dantes H (1991) El dengue en las Américas. Un problema de salud regional. Salud Publica Mex 33: 347-355.

1098. Gomez-Machorro C, Bennett KE, Munoz MD, Black WC (2004) Quantitative trait loci affecting dengue midgut infection barriers in an advanced intercross line of Aedes aegypti. Insect Mol Biol 13: 637-648.

1099. Goncalves Neto VS, Rebelo JM (2004) Aspectos epidemiológicos do dengue no Município de São Luís, Maranhão, Brasil, 1997-2002. Cad Saude Publica 20: 1424-1431.

1100. Goncalvez AP, Men R, Wernly C, Purcell RH, Lai CJ (2004) Chimpanzee Fab fragments and a derived humanized immunoglobulin G1 antibody that efficiently cross-neutralize dengue type 1 and type 2 viruses. J Virol 78: 12910-12918.

1101. Goncalvez AP, Purcell RH, Lai CJ (2004) Epitope determinants of a chimpanzee Fab antibody that efficiently cross-neutralizes dengue type 1 and type 2 viruses map to inside and in close proximity to fusion loop of the dengue type 2 virus envelope glycoprotein. J Virol 78: 12919-12928.

1102. Gonzales SO (1998) DENGUE/DHF - PHILIPPINES. In: ProMED-mail, editor. Philippine Daily Inquirer 1Sep 1998 ed: PROMED.

1103. Gonzalez AL, Martinez RA, Villar LA (2008) Evolución clínica de pacientes hospitalizados por dengue en una institución de salud de Bucaramanga, Colombia. Biomedica 28: 531-543.

1104. Gonzalez D, Castro OE, Kouri G, Perez J, Martinez E, et al. (2005) Classical dengue hemorrhagic fever resulting from two dengue infections spaced 20 years or more apart: Havana, Dengue 3 epidemic, 2001-2002. Int J Infect Dis 9: 280-285.

1105. Gorrochotegui-Escalante N, Lozano-Fuentes S, Bennett KE, Molina-Cruz A, Beaty BJ, et al. (2005) Association mapping of segregating sites in the early trypsin gene and susceptibility to dengue-2 virus in the mosquito Aedes aegypti. Insect Biochem Mol Biol 35: 771-788.

1106. Goto A, Roesel T (2006) Dengue/DHF update 2006 (23) PROMED: Promed.

1107. Gould EA, Higgs S, Buckley A, Gritsun TS (2006) Potential arbovirus emergence and implications for the United Kingdom. Emerg Infect Dis 12: 549-555.

1108. Goursaud R (1998) DENGUE - GUADELOUPE: 1997. In: Guadeloupe LdaMIPd, editor: PROMED.

1109. Graham RR, Juffrie M, Tan R, Hayes CG, Laksono I, et al. (1999) A prospective seroepidemiologic study on dengue in children four to nine years of age in Yogyakarta, Indonesia I. studies in 1995-1996. Am J Trop Med Hyg 61: 412-419.

1110. Green D (1997) DENGUE - AUSTRALIA (QUEENSLAND). In: Systems TS, editor. <http://www.q-net.net.au/~legion> ed. Perth: PROMED.

1111. Grobusch MP, Niedrig M, Gobels K, Klipstein-Grobusch K, Teichmann D (2006) Evaluation of the use of RT-PCR for the early diagnosis of dengue fever. Clin Microbiol Infect 12: 395-397.

1112. Guard RW, Stallman ND, Wiemers MA (1984) Dengue in the northern region of Queensland, 1981-1982. Med J Aust 140: 765-769.

1113. Gubler DJ (1987) Dengue and dengue hemorrhagic fever in the Americas. P R Health Sci J 6: 107-111.

1114. Gubler DJ (1989) Surveillance for dengue and dengue hemorrhagic fever. Bull Pan Am Health Organ 23: 397-404.

1115. Gubler DJ (1998) Dengue and dengue hemorrhagic fever. Clin Microbiol Rev 11: 480-496.

1116. Gubler DJ (2004) The changing epidemiology of yellow fever and dengue, 1900 to 2003: full circle? Comp Immunol Microbiol Infect Dis 27: 319-330.

1117. Gubler DJ (1989) La vigilancia activa del dengue y la fiebre hemorrágica del dengue. Bol Oficina Sanit Panam 107: 22-30.

1118. Gubler DJ (1996) DENGUE - SAMOA (2). PROMED.

1119. Gubler DJ (1983) Dengue in the United States, 1981. MMWR Morb Mortal Wkly Rep 32: 23SS-26SS.

1120. Gubler DJ (1984) Dengue in the United States, 1982. MMWR Surveill Summ 33: 9SS-13SS.

1121. Gubler DJ (1985) Dengue in the United States, 1983-1984. MMWR Surveill Summ 34: 5SS-8SS.

1122. Gubler DJ, Clark GG (1994) Community-based integrated control of Aedes aegypti: a brief overview of current programs. Am J Trop Med Hyg 50: 50-60.

1123. Gubler DJ, Kuno G, Sather GE, Velez M, Oliver A (1984) Mosquito cell cultures and specific monoclonal antibodies in surveillance for dengue viruses. Am J Trop Med Hyg 33: 158-165.

1124. Gubler DJ, Novak RJ, Vergne E, Colon NA, Velez M, et al. (1985) Aedes (Gymnometopa) Mediovittatus (Diptera, Culicidae), a Potential Maintenance Vector of Dengue Viruses in Puerto-Rico. J Med Entomol 22: 469-475.

1125. Gubler DJ, Reed D, Rosen L, Hitchcock JR, Jr. (1978) Epidemiologic, clinical, and virologic observations on dengue in the Kingdom of Tonga. Am J Trop Med Hyg 27: 581-589.

1126. Gubler DJ, Sather GE, Kuno G, Cabral JR (1986) Dengue 3 virus transmission in Africa. Am J Trop Med Hyg 35: 1280-1284.

1127. Gubler DJ, Suharyono W, Lubis I, Eram S, Gunarso S (1981) Epidemic dengue 3 in central Java, associated with low viremia in man. Am J Trop Med Hyg 30: 1094-1099.

1128. Gubler DJ, Suharyono W, Lubis I, Eram S, Sulianti Saroso J (1979) Epidemic dengue hemorrhagic fever in rural Indonesia. I. Virological and epidemiological studies. Am J Trop Med Hyg 28: 701-710.

1129. Guilarde AO, Turchi MD, Siqueira JB, Jr., Feres VC, Rocha B, et al. (2008) Dengue and dengue hemorrhagic fever among adults: clinical outcomes related to viremia, serotypes, and antibody response. J Infect Dis 197: 817-824.

1130. Guirakhoo F, Pugachev K, Zhang Z, Myers G, Levenbook I, et al. (2004) Safety and efficacy of chimeric yellow fever-dengue virus tetravalent vaccine formulations in nonhuman primates. J Virol 78: 4761-4775.

1131. Guirakhoo F, Zhang Z, Myers G, Johnson BW, Pugachev K, et al. (2004) A single amino acid substitution in the envelope protein of chimeric yellow fever-dengue 1 vaccine virus reduces neurovirulence for suckling mice and viremia/viscerotropism for monkeys. J Virol 78: 9998-10008.

1132. Gulati S, Maheshwari A (2007) Atypical manifestations of dengue. Trop Med Int Health 12: 1087-1095.

1133. Gunakasem P, Chantrasri C, Chaiyanun S, Simasathien P, Jatanasen S, et al. (1981) Surveillance of dengue hemorrhagic fever cases in Thailand. Southeast Asian J Trop Med Public Health 12: 338-343.

1134. Gunther J, Ramirez-Palacio LR, Perez-Ishiwara DG, Salas-Benito JS (2009) Distribution of dengue cases in the state of Oaxaca, Mexico, during the period 2004-2006. J Clin Virol 45: 218-222.

1135. Gupta A, Srinivasan R, Setia S, Soundravally R, Pandian DG (2009) Uveitis following dengue fever. Eye 23: 873-876.

1136. Gupta E, Dar L, Broor S (2008) Concurrent infection by two dengue virus serotypes among dengue patients. Indian J Med Microbiol 26: 402-403.

1137. Gupta E, Dar L, Kapoor G, Broor S (2006) The changing epidemiology of dengue in Delhi, India. Virol J 3: -.

1138. Gupta E, Dar L, Narang P, Srivastava VK, Broor S (2005) Serodiagnosis of dengue during an outbreak at a tertiary care hospital in Delhi. Indian J Med Res 121: 36-38.

1139. Gupta P, Kumar P, Aggarwal OP (1998) Knowledge, attitude and practices related to dengue in rural and slum areas of Delhi after the dengue epidemic of 1996. J Commun Dis 30: 107-112.

1140. Gurtler RE, Garelli FM, Coto HD (2009) Effects of a Five-Year Citywide Intervention Program To Control Aedes aegypti and Prevent Dengue Outbreaks in Northern Argentina. PLoS Negl Trop Dis 3: -.

1141. Gustave J (1996) La prévention de la dengue en Guadeloupe. Bull Soc Pathol Exot Filiales 89: 143-144.

1142. Guy B, Chanthavanich P, Gimenez S, Sirivichayakul C, Sabchareon A, et al. (2004) Evaluation by flow cytometry of antibody-dependent enhancement (ADE) of dengue infection by sera from Thai children immunized with a live-attenuated tetravalent dengue vaccine. Vaccine 22: 3563-3574.

1143. Guzman MG, Alvarez M, Rodriguez R, Rosario D, Vazquez S, et al. (1999) Fatal dengue hemorrhagic fever in Cuba, 1997. Int J Infect Dis 3: 130-135.

1144. Guzman MG, Alvarez M, Rodriguez-Roche R, Bernardo L, Montes T, et al. (2007) Neutralizing antibodies after infection with dengue 1 virus. Emerg Infect Dis 13: 282-286.

1145. Guzman MG, Deubel V, Pelegrino JL, Rosario D, Marrero M, et al. (1995) Partial nucleotide and amino acid sequences of the envelope and the envelope/nonstructural protein-1 gene junction of four dengue-2 virus strains isolated during the 1981 Cuban epidemic. Am J Trop Med Hyg 52: 241-246.

1146. Guzman MG, Garcia G, Kouri G (2006) El dengue y el dengue hemorrágico: prioridades de investigación. Rev Panam Salud Publica 19: 204-215.

1147. Guzman MG, Kouri G (2002) Dengue: an update. Lancet Infect Dis 2: 33-42.

1148. Guzman MG, Kouri G (2004) Dengue diagnosis, advances and challenges. Int J Infect Dis 8: 69-80.

1149. Guzman MG, Kouri G (2008) Dengue haemorrhagic fever integral hypothesis: confirming observations, 1987-2007. Trans R Soc Trop Med Hyg 102: 522-523.

1150. Guzman MG, Kouri G, Bravo J, Soler M, Martinez E (1991) Sequential infection as risk factor for dengue hemorrhagic fever/dengue shock syndrome (DHF/DSS) during the 1981 dengue hemorrhagic Cuban epidemic. Mem Inst Oswaldo Cruz 86: 367.

1151. Guzman MG, Kouri G, Bravo J, Valdes L, Vazquez S, et al. (2002) Effect of age on outcome of secondary dengue 2 infections. Int J Infect Dis 6: 118-124.

1152. Guzman MG, Kouri G, Halstead SB (2000) Do escape mutants explain rapid increases in dengue case-fatality rates within epidemics? Lancet 355: 1902-1903.

1153. Guzman MG, Kouri G, Martinez E, Bravo J, Riveron R, et al. (1987) Clinical and serologic study of Cuban children with dengue hemorrhagic fever/dengue shock syndrome (DHF/DSS). Bull Pan Am Health Organ 21: 270-279.

1154. Guzman MG, Kouri G, Morier L, Soler M, Fernandez A (1984) A study of fatal hemorrhagic dengue cases in Cuba, 1981. Bull Pan Am Health Organ 18: 213-220.

1155. Guzman MG, Kouri G, Soler M, Bravo J, Rodriguez de La Vega A, et al. (1992) Dengue 2 virus enhancement in asthmatic and non asthmatic individual. Mem Inst Oswaldo Cruz 87: 559-564.

1156. Guzman MG, Kouri G, Valdes L, Bravo J, Alvarez M, et al. (2000) Epidemiologic studies on Dengue in Santiago de Cuba, 1997. Am J Epidemiol 152: 793-799.

1157. Guzman MG, Kouri G, Valdes L, Bravo J, Vazquez S, et al. (2002) Enhanced severity of secondary dengue-2 infections: death rates in 1981 and 1997 Cuban outbreaks. Rev Panam Salud Publica 11: 223-227.

1158. Guzman MG, Kouri GP, Bravo J, Calunga M, Soler M, et al. (1984) Dengue haemorrhagic fever in Cuba. I. Serological confirmation of clinical diagnosis. Trans R Soc Trop Med Hyg 78: 235-238.

1159. Guzman MG, Kouri GP, Bravo J, Soler M, Vazquez S, et al. (1990) Dengue hemorrhagic fever in Cuba, 1981: a retrospective seroepidemiologic study. Am J Trop Med Hyg 42: 179-184.

1160. Guzman MG, Kouri GP, Bravo J, Soler M, Vazquez S, et al. (1984) Dengue haemorrhagic fever in Cuba. II. Clinical investigations. Trans R Soc Trop Med Hyg 78: 239-241.

1161. Guzman MG, Pelaez O, Kouri G, Quintana I, Vazquez S, et al. (2006) Caracterización final y lecciones de la epidemia de dengue 3 en Cuba, 2001–2002. Rev Panam Salud Publica 19: 282-289.

1162. Guzman MG, Rosario D, Mune M, Alvarez M, Rodriguez R, et al. (1996) Relaciones genéticas del virus dengue 3 aislado en la epidemia de FHD en Nicaragua, 1994. Rev Cuba Med Trop 48: 114-117.

1163. Guzman MG, Triana C, Bravo J, Kouri G (1992) Estimación de las afectaciones económicas causadas como consecuencia de la epidemia de dengue hemorrágico ocurrida en Cuba en 1981. Rev Cuba Med Trop 44: 13-17.

1164. Guzman MG, Vazquez S, Martinez E, Alvarez M, Rodriguez R, et al. (1996) Dengue in Nicaragua, 1994: reintroduction of serotype 3 in the Americas. Bol Oficina Sanit Panam 121: 102-110.

1165. Guzman Tirado MG (1980) Dengue. l. Antecedentes históricos; agentes etiologicos; cuadro clínico. Rev Cuba Med Trop 32: 123-130.

1166. Guzman Tirado MG, Kouri Flores G, Bravo Gonzalez J, Silva LC, Vazquez Ramudo S (1984) Encuesta serologica nacional a virus dengue. Cuba 1982. Rev Cuba Med Trop 36: 124-131.

1167. Guzman Tirado MG, Kouri G, Bravo JR, de la Hoz F, Soler M, et al. (1989) Encuesta seroepidemiologica retrospectiva a vrius dengue en los municipios cienfuegos y palmira. Rev Cuba Med Trop 41: 321-332.

1168. Gwinn W, Sun W, Innis BL, Caudill J, King AD (2003) Serotype-specific TH1 responses in recipients of two doses of candidate live-attenuated dengue virus vaccines. Am J Trop Med Hyg 69: 39-47.

1169. Ha DQ (1995) VIROLOGICAL ASPECTS OF DENGUE HAEMORRHAGIC FEVER IN SOUTH VIETNAM DURING 1991 - 199. In: Pasteur Institute Ho Chi Minh city V, editor. Ho Chi Minh city: PROMED.

1170. Ha DQ (1996) DENGUE - VIETNAM (02). In: Institute P, editor. Hanoi: PROMED.

1171. Ha DQ, Tien NT, Huong VT, Loan HT, Thang CM (2000) Dengue epidemic in southern Vietnam, 1998. Emerg Infect Dis 6: 422-425.

1172. Hadad M (2004) DENGUE/DHF UPDATE 2004 (24) [Afghanistan/ Vietnam/ Philippines/ Saudi Arabia]. In: ProMED-mail, editor. Globe and Mail 4 Aug 2004 [edited] ed: PROMED.

1173. Hafner C, Koellner K, Vogt T, Landthaler M, Szeimies RM (2005) [Hemorrhagic dengue fever after trip to Malaysia.]. Hautarzt.

1174. Hafner C, Koellner K, Vogt T, Landthaler M, Szeimies RM (2006) Hämorrhagisches Dengue-Fieber nach Malaysia-Aufenthalt. Hautarzt 57: 705-707.

1175. Hairi F, Ong CH, Suhaimi A, Tsung TW, bin Anis Ahmad MA, et al. (2003) A knowledge, attitude and practices (KAP) study on dengue among selected rural communities in the Kuala Kangsar district. Asia Pac J Public Health 15: 37-43.

1176. Haldar A, Gupta UD, Majumdar KK, Laskar K, Ghosh S, et al. (2008) Community perception of Dengue in slum areas of metropolitan city of West Bengal. J Commun Dis 40: 205-210.

1177. Hales S, de Wet N, Maindonald J, Woodward A (2002) Potential effect of population and climate changes on global distribution of dengue fever: an empirical model. Lancet 360: 830-834.

1178. Halide H, Ridd P (2008) A predictive model for Dengue Hemorrhagic Fever epidemics. Int J Environ Health Res 18: 253-265.

1179. Halstead SB (1984) Selective primary health care: strategies for control of disease in the developing world. XI. Dengue. Rev Infect Dis 6: 251-264.

1180. Halstead SB (1988) Pathogenesis of dengue: challenges to molecular biology. Science 239: 476-481.

1181. Halstead SB (1992) The XXth century dengue pandemic: need for surveillance and research. World Health Statistics Quarterly Rapport Trimestriel de Statistiques Sanitaires Mondiales 45: 292-298.

1182. Halstead SB (1994) Dengue in the health transition. Gaoxiong Yi Xue Ke Xue Za Zhi 10 Suppl: S2-14.

1183. Halstead SB (2002) Dengue. Curr Opin Infect Dis 15: 471-476.

1184. Halstead SB (1974) Etiologies of the experimental dengues of Siler and Simmons. Am J Trop Med Hyg 23: 974-982.

1185. Halstead SB (1998) DENGUE - THAILAND (05). In: Research DoMSaTOoN, editor. Arlington: PROMED.

1186. Halstead SB, Lan NT, Myint TT, Shwe TN, Nisalak A, et al. (2002) Dengue hemorrhagic fever in infants: research opportunities ignored. Emerg Infect Dis 8: 1474-1479.

1187. Halstead SB, Marchette NJ (2003) Biologic properties of dengue viruses following serial passage in primary dog kidney cells: studies at the University of Hawaii. Am J Trop Med Hyg 69: 5-11.

1188. Halstead SB, Streit TG, Lafontant JG, Putvatana R, Russell K, et al. (2001) Haiti: absence of dengue hemorrhagic fever despite hyperendemic dengue virus transmission. Am J Trop Med Hyg 65: 180-183.

1189. Hammon WM, Sather GE, Bond JO, Lewis FY (1966) Effect of previous dengue infection and yellow fever vaccination on St. Louis encephalitis virus serological surveys in Tampa Bay area of Florida. Am J Epidemiol 83: 571-585.

1190. Hammond SN, Balmaseda A, Perez L, Tellez Y, Saborio SI, et al. (2005) Differences in dengue severity in infants, children, and adults in a 3-year hospital-based study in Nicaragua. Am J Trop Med Hyg 73: 1063-1070.

1191. Hanafusa S, Chanyasanha C, Sujirarat D, Khuankhunsathid I, Yaguchi A, et al. (2008) Clinical features and differences between child and adult dengue infections in Rayong Province, southeast Thailand. Southeast Asian J Trop Med Public Health 39: 252-259.

1192. Hang VT, Nguyet NM, Trung DT, Tricou V, Yoksan S, et al. (2009) Diagnostic Accuracy of NS1 ELISA and Lateral Flow Rapid Tests for Dengue Sensitivity, Specificity and Relationship to Viraemia and Antibody Responses. PLoS Negl Trop Dis 3: -.

1193. Hanley KA, Goddard LB, Gilmore LE, Scott TW, Speicher J, et al. (2005) Infectivity of west Nile/Dengue chimeric viruses for West Nile and dengue mosquito vectors. Vector-Borne and Zoonotic Diseases 5: 1-10.

1194. Hanley KA, Manlucu LR, Manipon GG, Hanson CT, Whitehead SS, et al. (2004) Introduction of mutations into the non-structural genes or 3 ' untranslated region of an attenuated dengue virus type 4 vaccine candidate further decreases replication in rhesus monkeys while retaining protective immunity. Vaccine 22: 3440-3448.

1195. Hanley KA, Nelson JT, Schirtzinger EE, Whitehead SS, Hanson CT (2008) Superior infectivity for mosquito vectors contributes to competitive displacement among strains of dengue virus. BMC Ecol 8: 1.

1196. Hanna J (1998) DENGUE - AUSTRALIA (TORRES STRAIT) (03). Cairns: PROMED.

1197. Hanna JN, Ritchie SA, Hills SL, Pyke AT, Montgomery BL, et al. (2003) Dengue in north Queensland, 2002. Commun Dis Intell 27: 384-389.

1198. Hanna JN, Ritchie SA, Merritt AD, van den Hurk AF, Phillips DA, et al. (1998) Two contiguous outbreaks of dengue type 2 in north Queensland. Med J Aust 168: 221-225.

1199. Hanna JN, Ritchie SA, Phillips DA, Serafin IL, Hills SL, et al. (2001) An epidemic of dengue 3 in far north Queensland, 1997-1999. Med J Aust 174: 178-182.

1200. Hansch C, Verma RP (2009) Larvicidal activities of some organotin compounds on mosquito larvae: A QSAR study. Eur J Med Chem 44: 260-273.

1201. Hapugoda MD, Batra G, Abeyewickreme W, Swaminathan S, Khanna N (2007) Single antigen detects both immunoglobulin M (IgM) and IgG antibodies elicited by all four dengue virus serotypes. Clin Vaccine Immunol 14: 1505-1514.

1202. Haritoglou C, Scholz F, Bialasiewicz A, Klauss V (2000) Okuläre Manifestation bei Dengue-Fieber. Ophthalmologe 97: 433-436.

1203. Harn MR (1989) [Clinical study on dengue fever during 1987-1988 epidemic at Kaohsiung City, southern Taiwan]. Gaoxiong Yi Xue Ke Xue Za Zhi 5: 58-65.

1204. Harn MR, Chiang YL, Tian MJ, Chang YH, Ko YC (1993) [The 1991 dengue epidemic in Kaohsiung City]. J Formos Med Assoc 92 Suppl 1: S39-43.

1205. Harris E, Perez L, Phares CR, Perez Mde L, Idiaquez W, et al. (2003) Fluid intake and decreased risk for hospitalization for dengue fever, Nicaragua. Emerg Infect Dis 9: 1003-1006.

1206. Harris E, Roberts TG, Smith L, Selle J, Kramer LD, et al. (1998) Typing of dengue viruses in clinical specimens and mosquitoes by single-tube multiplex reverse transcriptase PCR. J Clin Microbiol 36: 2634-2639.

1207. Harris E, Videa E, Perez L, Sandoval E, Tellez Y, et al. (2000) Clinical, epidemiologic, and virologic features of dengue in the 1998 epidemic in Nicaragua. Am J Trop Med Hyg 63: 5-11.

1208. Harris VK, Danda D, Murali NS, Das PK, Abraham M, et al. (2000) Unusual association of Kikuchi's disease and dengue virus infection evolving into systemic lupus erythematosus. J Indian Med Assoc 98: 391-393.

1209. Harrison B (1998) DENGUE VIRUSES, TRANSMISSION BY AEDES ALBOPICTUS (08) [Thailand]. In: Resources NCDoEN, editor: PROMED.

1210. Harving ML, Ronsholt FF (2007) The economic impact of dengue hemorrhagic fever on family level in Southern Vietnam. Dan Med Bull 54: 170-172.

1211. Hashimoto S, Kawado M, Murakami Y, Izumida M, Ohta A, et al. (2007) Epidemics of vector-borne diseases observed in infectious disease surveillance in Japan, 2000-2005. J Epidemiol 17: S48-S55.

1212. Hasler C, Schnorf H, Enderlin N, Gyr K (1993) Importiertes Dengue-Fieber nach einem Tropenaufenthalt. Schweiz Med Wochenschr 123: 120-124.

1213. Hati AK (2006) Studies on dengue and dengue haemorrhagic fever (DHF) in West Bengal State, India. J Commun Dis 38: 124-129.

1214. Hayes CG, Manaloto CR, Gonzales A, Ranoa CP (1988) Dengue infections in the Philippines: clinical and virological findings in 517 hospitalized patients. Am J Trop Med Hyg 39: 110-116.

1215. Hayes CG, O'Rourke TF, Fogelman V, Leavengood DD, Crow G, et al. (1989) Dengue fever in American military personnel in the Philippines: clinical observations on hospitalized patients during a 1984 epidemic. Southeast Asian J Trop Med Public Health 20: 1-8.

1216. Hayes CG, Phillips IA, Callahan JD, Griebenow WF, Hyams KC, et al. (1996) The epidemiology of dengue virus infection among urban, jungle, and rural populations in the Amazon region of Peru. Am J Trop Med Hyg 55: 459-463.

1217. Hayes JM, Garcia-Rivera E, Flores-Reyna R, Suarez-Rangel G, Rodriguez-Mata T, et al. (2003) Risk factors for infection during a severe dengue outbreak in El Salvador in 2000. Am J Trop Med Hyg 69: 629-633.

1218. Hayes JM, Rigau-Perez JG, Reiter P, Effler PV, Pang L, et al. (2006) Risk factors for infection during a dengue-1 outbreak in Maui, Hawaii, 2001. Trans R Soc Trop Med Hyg 100: 559-566.

1219. Heddini A, Janzon R, Linde A (2009) Increased Number of Dengue Cases in Swedish Travellers to Thailand. Eurosurveillance 14: -.

1220. Helbok R, Dent W, Gattringer K, Innerebner M, Schmutzhard E (2004) Imported Dengue fever presenting with febrile diarrhoea: Report of two cases. Wien Klin Wochenschr 116: 58-60.

1221. Helt AM, Harris E (2005) S-phase-dependent enhancement of dengue virus 2 replication in mosquito cells, but not in human cells. J Virol 79: 13218-13230.

1222. Henchal EA, Repik PM, McCown JM, Brandt WE (1986) Identification of an antigenic and genetic variant of dengue-4 virus from the Caribbean. Am J Trop Med Hyg 35: 393-400.

1223. Hendarto SK, Hadinegoro SR (1992) Dengue encephalopathy. Acta Paediatr Jpn 34: 350-357.

1224. Henika P (1998) DENGUE/DHF - INDONESIA (NORTH SULAWESI). In: ProMED-mail, editor. Kompas daily on Nando net ed: PROMED.

1225. Hermida L, Bernardo L, Martin J, Alvarez M, Prado I, et al. (2006) A recombinant fusion protein containing the domain III of the dengue-2 envelope protein is immunogenic and protective in nonhuman primates. Vaccine 24: 3165-3171.

1226. Hermida L, Rodriguez R, Lazo L, Bernardo L, Silva R, et al. (2004) A fragment of the envelope protein from dengue-I virus, fused in two different sites of the meningococcal P64k protein carrier, induces a functional immune response in mice. Biotechnol Appl Biochem 39: 107-114.

1227. Hermida L, Rodriguez R, Lazo L, Silva R, Zulueta A, et al. (2004) A dengue-2 Envelope fragment inserted within the structure of the P64k meningococcal protein carrier enables a functional immune response against the virus in mice. J Virol Methods 115: 41-49.

1228. Hernandez N, Ramirezronda CH (1983) Dengue in Puerto-Rico 1981-82 Epidemic - a Study of 40 Confirmed Cases. Clin Res 31: A365-A365.

1229. Herrera-Basto E, Prevots DR, Zarate ML, Silva JL, Sepulveda-Amor J (1992) First reported outbreak of classical dengue fever at 1,700 meters above sea level in Guerrero State, Mexico, June 1988. Am J Trop Med Hyg 46: 649-653.

1230. Heukelbach J, de Oliveira FA, Kerr-Pontes LR, Feldmeier H (2001) Risk factors associated with an outbreak of dengue fever in a favela in Fortaleza, north-east Brazil. Trop Med Int Health 6: 635-642.

1231. Hills S, Piispanen J, Foley P, Smith G, Humphreys J, et al. (2000) Public health implications of dengue in personnel returning from East Timor. Commun Dis Intell 24: 365-368.

1232. Hills SL, Piispanen JP, Humphreys JL, Foley PN (2002) A focal, rapidly-controlled outbreak of dengue fever in two suburbs in Townsville, north Queensland, 2001. Commun Dis Intell 26: 596-600.

1233. Hiriyan J, Tyagi BK (2004) Cocoa pod (Theobroma caco)--a potential breeding habit of Aedes albopictus in dengue-sensitive Kerala State, India. J Am Mosq Control Assoc 20: 323-325.

1234. Ho LJ, Hung LF, Weng CY, Wu WL, Chou P, et al. (2005) Dengue virus type 2 antagonizes IFN-alpha but not IFN-gamma antiviral effect via down-regulating Tyk2-STAT signaling in the human dendritic cell. J Immunol 174: 8163-8172.

1235. Ho LJ, Shaio MF, Chang DM, Liao CL, Lai JH (2004) Infection of human dendritic cells by dengue virus activates and primes T cells towards Th0-like phenotype producing both Th1 and Th2 cytokines. Immunol Invest 33: 423-437.

1236. Hoang LP, Thai KTD, Nga TTT, Giao PT, Hung LQ, et al. (2009) Detection of dengue nonstructural 1 (NS1) protein in Vietnamese patients with fever. Diagn Microbiol Infect Dis 63: 372-378.

1237. Hober D, Delannoy AS, Benyoucef S, De Groote D, Wattre P (1996) High levels of sTNFR p75 and TNF alpha in dengue-infected patients. Microbiol Immunol 40: 569-573.

1238. Hober D, Poli L, Roblin B, Gestas P, Chungue E, et al. (1993) Serum levels of tumor necrosis factor-alpha (TNF-alpha), interleukin-6 (IL-6), and interleukin-1 beta (IL-1 beta) in dengue-infected patients. Am J Trop Med Hyg 48: 324-331.

1239. Hochedez P, Canestri A, Guihot A, Brichler S, Bricaire F, et al. (2008) Management of travelers with fever and exanthema, notably dengue and chikungunya infections. Am J Trop Med Hyg 78: 710-713.

1240. Holden KL, Harris E (2004) Enhancement of dengue virus translation: role of the 3 ' untranslated region and the terminal 3 ' stem-loop domain. Virology 329: 119-133.

1241. Holden KL, Stein DA, Pierson TC, Ahmed AA, Clyde K, et al. (2006) Inhibition of dengue virus translation and RNA synthesis by a morpholino oligomer targeted to the top of the terminal 3 ' stem-loop structure. Virology 344: 439-452.

1242. Holmes DA, Purdy DE, Chao DY, Noga AJ, Chang GJJ (2005) Comparative analysis of immunoglobulin M (IgM) capture enzyme-linked immunosorbent assay using virus-like particles or virus-infected mouse brain antigens to detect IgM antibody in sera from patients with evident flaviviral infections. J Clin Microbiol 43: 3227-3236.

1243. Holmes EC (2007) The evolution and epidemiology of dengue virus. Salud Publica Mex 49: E296-E300.

1244. Homchampa P, Sarasombath S, Suvatte V, Vongskul M (1988) Natural killer cells in dengue hemorrhagic fever/dengue shock syndrome. Asian Pac J Allergy Immunol 6: 95-102.

1245. Hommel D, Talarmin A, Deubel V, Reynes JM, Drouet MT, et al. (1998) Dengue encephalitis in French Guiana. Res Virol 149: 235-238.

1246. Honda S, Saito M, Dimaano EM, Morales PA, Alonzo MTG, et al. (2009) Increased Phagocytosis of Platelets from Patients with Secondary Dengue Virus Infection by Human Macrophages. Am J Trop Med Hyg 80: 841-845.

1247. Hongsiriwon S (2002) Dengue hemorrhagic fever in infants. Southeast Asian J Trop Med Public Health 33: 49-55.

1248. Honorio NA, Castro MG, de Barros FS, Magalhaes Mde A, Sabroza PC (2009) The spatial distribution of Aedes aegypti and Aedes albopictus in a transition zone, Rio de Janeiro, Brazil. Cad Saude Publica 25: 1203-1214.

1249. Honorio NA, Silva Wda C, Leite PJ, Goncalves JM, Lounibos LP, et al. (2003) Dispersal of Aedes aegypti and Aedes albopictus (Diptera: Culicidae) in an urban endemic dengue area in the State of Rio de Janeiro, Brazil. Mem Inst Oswaldo Cruz 98: 191-198.

1250. Honsawek S, Kongtawelert P, Pothacharoen P, Khongphatthanayothin A, Chongsrisawat V, et al. (2007) Increased levels of serum hyaluronan in patients with dengue infection. J Infect 54: 225-229.

1251. Hopp M (2004) DENGUE/DHF UPDATE 2004 (10) [Indonesia]. In: ProMED-mail, editor. WHO Dengue fever in Indonesia - update 2 -- 29 March 2004 [edited] ed: PROMED.

1252. Hopp M (2004) DENGUE/DHF UPDATE 2004 (06) [Indonesia/ Peru]. In: ProMED-mail, editor. WHO Outbreak News 26 Feb 2004 [edited] ed: PROMED.

1253. Hopp M (2005) DENGUE/DHF UPDATE 2005 (04). PROMED.

1254. Hopp M (2005) DENGUE/DHF UPDATE 2005 (05). PROMED.

1255. Hopp M (2007) Dengue/DHF update 2007 (05). PROMED.

1256. Hopp M, Marshall M, Dudley J (2007) Dengue/DHF update 2007 (05) PROMED: Promed.

1257. Horvath H (1997) DENGUE - COSTA RICA (03). In: mail P, editor. La Nacion (Costa Rica), 13 August 1997 ed: PROMED.

1258. Horvath H (1997) DENGUE - COSTA RICA. La Nacion (Costa Rica), June 5, 1997 <http://www.nacion.co.cr/ln_ee/1997/junio/05/pagina06.html> ed: PROMED.

1259. Hospedales CJ (1990) An update on Dengue fever in the Caribbean. West Indian Med J 39: 131.

1260. Hospedales CJ (1990) Dengue fever in the Caribbean. West Indian Med J 39: 59-62.

1261. Hospedales J (1997) DENGUE - VENEZUELA (CARACAS) (02). In: CAREC/PAHO/WHO, editor. (Trinidad and Tobago): PROMED.

1262. Hossain MA, Khatun M, Arjumand F, Nisaluk A, Breiman RF (2003) Serologic evidence of dengue infection before onset of epidemic, Bangladesh. Emerg Infect Dis 9: 1411-1414.

1263. Houghton-Trivino N, Montana D, Castellanos J (2008) Dengue-yellow fever sera cross-reactivity; challenges for diagnosis. Revista de Salud Pública 10: 299-307.

1264. How A (2001) DENGUE/DHF UPDATES (16): 19 OCT 2001 [Hawaii]. In: ProMED-mail, editor. The Hawaii Channel, Wed 17 Oct 2001 [edited] ed: PROMED.

1265. Hrobowski YM, Garry RF, Michael SF (2005) Peptide inhibitors of dengue virus and West Nile virus infectivity. Virol J 2: 49.

1266. Hsieh YH, Chen CWS (2009) Turning points, reproduction number, and impact of climatological events for multi-wave dengue outbreaks. Trop Med Int Health 14: 628-638.

1267. Hsieh YH, Ma S (2009) Intervention Measures, Turning Point, and Reproduction Number for Dengue, Singapore, 2005. Am J Trop Med Hyg 80: 66-71.

1268. Hu HP, Hsieh SC, King CC, Wang WK (2007) Characterization of retrovirus-based reporter viruses pseudotyped with the precursor membrane and envelope glycoproteins of four serotypes of dengue viruses. Virology 368: 376-387.

1269. Huang CYH, Silengo SJ, Whiteman MC, Kinney RM (2005) Chimeric dengue 2 PDK-53/West Nile NY99 viruses retain the phenotypic attenuation markers of the candidate PDK-53 vaccine virus and protect mice against lethal challenge with West Nile virus. J Virol 79: 7300-7310.

1270. Huang JH, Liao TL, Chang SF, Su CL, Chien LJ, et al. (2007) Laboratory-based dengue surveillance in Taiwan, 2005: A molecular epidemiologic study. Am J Trop Med Hyg 77: 903-909.

1271. Huang KJ, Yang YC, Lin YS, Huang JH, Liu HS, et al. (2006) The dual-specific binding of dengue virus and target cells for the antibody-dependent enhancement of dengue virus infection. J Immunol 176: 2825-2832.

1272. Huang Q, Fu WL, Chen B, Huang JF, Zhang X, et al. (2004) Inactivation of dengue virus by methylene blue/narrow bandwidth light system. J Photochem Photobiol B Biol 77: 39-43.

1273. Huber K, Le Loan L, Hoang TH, Ravel S, Rodhain F, et al. (2002) Genetic differentiation of the dengue vector, Aedes aegypti (Ho Chi Minh City, Vietnam) using microsatellite markers. Mol Ecol 11: 1629-1635.

1274. Huber K, Luu Le L, Tran Huu H, Tran Khanh T, Rodhain F, et al. (2002) Aedes aegypti, le vecteur des virus de la dengue : structure spatio-temporelle de la variabilité génétique. Bulletin de l Academie Nationale de Medecine 186: 1237-1248; discussion 1248-1250.

1275. Huber K, Mousson L, Rodhain F, Failloux AB (1999) Short report: microsatellite sequences as markers for population genetic studies of the mosquito Aedes aegypti, the vector of dengue viruses. Am J Trop Med Hyg 61: 1001-1003.

1276. Hubert B, Halstead SB (2009) Dengue 1 Virus and Dengue Hemorrhagic Fever, French Polynesia, 2001. Emerg Infect Dis 15: 1265-1270.

1277. Huerre MR, Lan NT, Marianneau P, Hue NB, Khun H, et al. (2001) Liver histopathology and biological correlates in five cases of fatal dengue fever in Vietnamese children. Virchows Arch 438: 107-115.

1278. Huhtamo E, Uzcategui NY, Siikamaki H, Saarinen A, Piiparinen H, et al. (2008) Molecular epidemiology of dengue virus strains from Finnish travelers. Emerg Infect Dis 14: 80-83.

1279. Huhtamo E, Vuorinen S, Uzcategui NY, Vapalahti O, Haapasalo H, et al. (2006) Fatal dengue virus infection in a Finnish traveler. J Clin Virol 37: 323-326.

1280. Hull B, Tikasingh E, de Souza M, Martinez R (1984) Natural transovarial transmission of dengue 4 virus in Aedes aegypti in Trinidad. Am J Trop Med Hyg 33: 1248-1250.

1281. Hung JJ, Hsieh MT, Young MJ, Kao CL, King CC, et al. (2004) An external loop region of domain III of dengue virus type 2 envelope protein is involved in serotype-specific binding to mosquito but not mammalian cells. J Virol 78: 378-388.

1282. Hung NT, Lan NT, Lei HY, Lin YS, Lien LB, et al. (2006) Volume Replacement in Infants with Dengue Hemorrhagic Fever/Dengue Shock Syndrome. Am J Trop Med Hyg 74: 684-691.

1283. Hung NT, Lan NT, Lei HY, Lin YS, Lien LB, et al. (2005) Association between sex, nutritional status, severity of dengue hemorrhagic fever, and immune status in infants with dengue hemorrhagic fever. Am J Trop Med Hyg 72: 370-374.

1284. Hung NT, Lei HY, Lan NT, Lin YS, Huang KJ, et al. (2004) Dengue hemorrhagic fever in infants: A study of clinical and cytokine profiles. J Infect Dis 189: 221-232.

1285. Husbands BS (1999) DENGUE/DHF - PHILIPPINES (LUZON). In: Jr. CAaCM, editor. Philippine Daily Inquirer [edited] ed: PROMED.

1286. Hussin N, Jaafar J, Naing NN, Mat HA, Muhamad AH, et al. (2005) A review of dengue fever incidence in Kota Bharu, Kelantan, Malaysia during the years 1998-2003. Southeast Asian J Trop Med Public Health 36: 1179-1186.

1287. Huy R, Wichmann O, Beatty M, Ngan C, Duong S, et al. (2009) Cost of dengue and other febrile illnesses to households in rural Cambodia: a prospective community-based case-control study. BMC Public Health 9: -.

1288. Hwang KP, Chu PY, Tung YC, Wang HL, Yueh YY, et al. (2003) Molecular epidemiological study of dengue virus type 1 in Taiwan. J Med Virol 70: 404-409.

1289. Hwang KP, Su SC, Chiang CH (1989) [Clinical observations of dengue fever among children]. Gaoxiong Yi Xue Ke Xue Za Zhi 5: 50-57.

1290. Hyams KC, Oldfield EC, Scott RM, Bourgeois AL, Gardiner H, et al. (1986) Evaluation of febrile patients in Port Sudan, Sudan: isolation of dengue virus. Am J Trop Med Hyg 35: 860-865.

1291. Ibrahim F, Ismail NA, Taib MN, Abas WABW (2004) Modeling of hemoglobin in dengue fever and dengue hemorrhagic fever using bioelectrical impedance. Physiol Meas 25: 607-615.

1292. Ibrahim F, Taib MN, Abas WABW, Guan CC, Sulaiman S (2005) A novel approach to classify risk in dengue hemorrhagic fever (DHF) using bioelectrical impedance analysis (BIA). IEEE Trans Instrum 54: 237-244.

1293. Ibrahim F, Taib MN, Abas WAW, Guan CC, Sulaiman S (2005) A novel dengue fever (DF) and dengue haemorrhagic fever (DHF) analysis using artificial neural network (ANN). Comput Methods Programs Biomed 79: 273-281.

1294. Ibrahim NM, Cheong I (1995) Adult dengue haemorrhagic fever at Kuala Lumpur Hospital: retrospective study of 102 cases. Br J Clin Pract 49: 189-191.

1295. Igarashi A (1997) Impact of dengue virus infection and its control. FEMS Immunol Med Microbiol 18: 291-300.

1296. Ilkal MA, Dhanda V, Hassan MM, Mavale M, Mahadev PV, et al. (1991) Entomological investigations during outbreaks of dengue fever in certain villages in Maharashtra state. Indian J Med Res 93: 174-178.

1297. Imbert P, Sordet D, Hovette P, Touze JE (1993) Spleen rupture in a patient with dengue fever. Trop Med Parasitol 44: 327-328.

1298. Imrie A, Meeks J, Gurary A, Sukhbaatar M, Truong TT, et al. (2007) Antibody to dengue 1 detected more than 60 years after infection. Viral Immunol 20: 672-675.

1299. Imrie A, Meeks J, Gurary A, Sukhbataar M, Kitsutani P, et al. (2007) Differential functional avidity of dengue virus-specific T-Cell clones for variant peptides representing heterologous and previously encountered serotypes. J Virol 81: 10081-10091.

1300. Imrie A, Zhao Z, Bennett SN, Kitsutani P, Laille M, et al. (2006) Molecular epidemiology of dengue in the Pacific: introduction of two distinct strains of dengue virus type-1 [corrected] into Hawaii. Ann Trop Med Parasitol 100: 327-336.

1301. Indaratna K, Hutubessy R, Chupraphawan S, Sukapurana C, Tao J, et al. (1998) Application of geographical information systems to co-analysis of disease and economic resources: dengue and malaria in Thailand. Southeast Asian J Trop Med Public Health 29: 669-684.

1302. Isarangkura P, Mahasandana C, Chuansumrit A, Angchaisuksiri P (2004) Acquired bleeding disorders: the impact of health problems in the developing world. Haemophilia 10: 188-195.

1303. Ishikawa H, Okada S, Katayama I, Mazaki H, Nagatake T, et al. (1999) A Japanese case of dengue fever with lymphocytic vasculitis: diagnosis by polymerase chain reaction. J Dermatol 26: 29-32.

1304. Islam MA, Ahmed MU, Begum N, Chowdhury NA, Khan AH, et al. (2006) Molecular characterization and clinical evaluation of dengue outbreak in 2002 in Bangladesh. Jpn J Infect Dis 59: 85-91.

1305. Itha S, Kashyap R, Krishnani N, Saraswat VA, Choudhuri G, et al. (2005) Profile of liver involvement in dengue virus infection. Natl Med J India 18: 127-130.

1306. Ito M, Takasaki T, Yamada KI, Nerome R, Tajima S, et al. (2004) Development and evaluation of fluorogenic TaqMan reverse transcriptase PCR assays for detection of dengue virus types 1 to 4. J Clin Microbiol 42: 5935-5937.

1307. Itoda I, Masuda G, Suganuma A, Imamura A, Ajisawa A, et al. (2006) Clinical features of 62 imported cases of dengue fever in Japan. Am J Trop Med Hyg 75: 470-474.

1308. Itrat A, Khan A, Javaid S, Kamal M, Khan H, et al. (2008) Knowledge, awareness and practices regarding dengue fever among the adult population of dengue hit cosmopolitan. PLoS One 3: e2620.

1309. Iturrino-Monge R, Avila-Aguero ML, Avila-Aguero CR, Moya-Moya T, Canas-Coto A, et al. (2006) Seroprevalence of dengue virus antibodies in asymptomatic Costa Rican children, 2002-2003: a pilot study. Rev Panam Salud Publica 20: 39-43.

1310. Jackson ST, Mullings A, Bennett F, Khan C, Gordon-Strachan G, et al. (2008) Dengue Infection in Patients Presenting with Neurological Manifestations in a Dengue Endemic Population. West Indian Med J 57: 373-376.

1311. Jacobs J, Fernandez EA, Merizalde B, Avila-Montes GA, Crothers D (2007) The use of homeopathic combination remedy for dengue fever symptoms: a pilot RCT in Honduras. Homeopathy 96: 22-26.

1312. Jain D, Singh T (2008) Dengue virus related hemophagocytosis: a rare case report. Hematology 13: 286-288.

1313. Jaiswal S, Khanna N, Swaminathan S (2004) High-level expression and one-step purification of recombinant dengue virus type 2 envelope domain III protein in Escherichia coli. Protein Expr Purif 33: 80-91.

1314. Jaiyen Y, Masrinoul P, Kalayanarooj S, Pulmanausahakul R, Ubol S (2009) Characteristics of dengue virus-infected peripheral blood mononuclear cell death that correlates with the severity of illness. Microbiol Immunol 53: 442-450.

1315. Jamaiah I, Rohela M, Nissapatorn V, Maizatulhikma MM, Norazlinda R, et al. (2005) Prevalence of dengue fever and dengue hemorrhagic fever in Hospital Tengku Ampuan Rahimah, Klang, Selangor, Malaysia. Southeast Asian J Trop Med Public Health 36 Suppl 4: 196-201.

1316. Jampangern W, Vongthoung K, Jittmittraphap A, Worapongpaiboon S, Limkittikul K, et al. (2007) Characterization of atypical lymphocytes and immunophenotypes of lymphocytes in patients with dengue virus infection. Asian Pac J Allergy Immunol 25: 27-36.

1317. Janjindamai W, Pruekprasert P (2003) Perinatal dengue infection: a case report and review of literature. Southeast Asian J Trop Med Public Health 34: 793-796.

1318. Jaufeerally FR, Surrun SK, Chang PE (2007) Acute acalculous cholecystitis in dengue hemorrhagic fever. Indian J Med Sci 61: 613-614.

1319. Jayakeerthi RS, Potula RV, Srinivasan S, Badrinath S (2006) Shell Vial Culture assay for the rapid diagnosis of Japanese encephalitis, West Nile and Dengue-2 viral encephalitis. Virol J 3: 2.

1320. Jayawarna C, McKendrick S, Fernando DJS (2006) Haemorrhagic fever in a returning tourist. Eur J Intern Med 17: 525-525.

1321. Jelinek T (2000) DENGUE - DOMINICAN REPUBLIC: RFI. Munich: PROMED.

1322. Jelinek T (2009) Trends in the Epidemiology of Dengue Fever and Their Relevance for Importation to Europe. Eurosurveillance 14: 4-6.

1323. Jelinek T, Dobler G, Holscher M, Loscher T, Nothdurft HD (1997) Prevalence of infection with dengue virus among international travelers. Arch Intern Med 157: 2367-2370.

1324. Jelinek T, Dobler G, Nothdurft HD (1998) Evidence of Dengue virus infection in a German couple returning from Hawaii. J Travel Med 5: 44-45.

1325. Jelinek T, Nart P (2002) DENGUE/DHF UPDATES (16): 26 APR 2002 [Thailand/Costa Rica]. In: ProMED-mail, editor: PROMED.

1326. Jensenius M, Berild D, Ormaasen V, Maehlen J, Lindegren G, et al. (2007) Fatal subarachnoidal haemorrhage in a Norwegian traveller with dengue virus infection. Scand J Infect Dis 39: 272-274.

1327. Jerzak G, Bernard KA, Kramer LD, Ebel GD (2005) Genetic variation in West Nile virus from naturally infected mosquitoes and birds suggests quasispecies structure and strong purifying selection. J Gen Virol 86: 2175-2183.

1328. Jessie K, Fong MY, Devi S, Lam SK, Wong KT (2004) Localization of dengue virus in naturally infected human tissues, by immunohistochemistry and in situ hybridization. J Infect Dis 189: 1411-1418.

1329. Jiang ZY, Shi YJ, Sun HX (2005) [Study on dengue virus infection of human dendritic cells]. Wei Sheng Wu Xue Bao 45: 598-600.

1330. Jimenez-Lucho VE, Fisher EJ, Saravolatz LD (1984) Dengue with hemorrhagic manifestations: an imported case from the Middle East. Am J Trop Med Hyg 33: 650-653.

1331. Jindadamrongwech S, Smith DR (2004) Virus overlay protein binding assay (VOPBA) reveals serotype specific heterogeneity of dengue virus binding proteins on HepG2 human liver cells. Intervirology 47: 370-373.

1332. Jindadamrongwech S, Thepparit C, Smith DR (2004) Identification of GRP 78 (BiP) as a liver cell expressed receptor element for dengue virus serotype 2. Arch Virol 149: 915-927.

1333. Johnson BW, Chambers TV, Crabtree MB, Guirakhoo F, Monath TP, et al. (2004) Analysis of the replication kinetics of the Chimerivax (TM)-DEN 1, 2, 3, 4 tetravalent virus mixture in Aedes aegypti by real-time reverse transcriptase-polymerase chain reaction. Am J Trop Med Hyg 70: 89-97.

1334. Johnson BW, Russell BJ, Lanciotti RS (2005) Serotype-specific detection of dengue viruses in a fourplex real-time reverse transcriptase PCR assay. J Clin Microbiol 43: 4977-4983.

1335. Joshi PT, Pandya AP, Anjan JK (2000) Epidemiological and entomological investigation in dengue outbreak area of Ahmedabad district. J Commun Dis 32: 22-27.

1336. Joshi R, Colford JM, Reingold AL, Kalantri S (2008) Nonmalarial acute undifferentiated fever in a rural hospital in central India: Diagnostic uncertainty and overtreatment with antimalarial agents. Am J Trop Med Hyg 78: 393-399.

1337. Joshi V, Mathur ML, Dixit AK, Singhi M (1996) Entomological studies in a dengue endemic area, Jalore, Rajasthan. Indian J Med Res 104: 161-165.

1338. Joshi V, Sharma RC, Sharma Y, Adha S, Sharma K, et al. (2006) Importance of socioeconomic status and tree holes in distribution of Aedes mosquitoes (Diptera : Culicidae) in Jodhpur, Rajasthan, India. J Med Entomol 43: 330-336.

1339. Joshipura VP, Soni HN, Patel NR, Haribhakti SP (2007) Dengue fever presenting as acute acalculus cholecystitis. J Indian Med Assoc 105: 338-339.

1340. Jumali, Sunarto, Gubler DJ, Nalim S, Eram S, et al. (1979) Epidemic dengue hemorrhagic fever in rural Indonesia. III. Entomological studies. Am J Trop Med Hyg 28: 717-724.

1341. Jupp PG, Kemp A (1993) The potential for dengue in South Africa: vector competence tests with dengue 1 and 2 viruses and 6 mosquito species. Trans R Soc Trop Med Hyg 87: 639-643.

1342. Jury MR (2008) Climate influence on dengue epidemics in Puerto Rico. Int J Environ Health Res 18: 323-334.

1343. Kabilan L, Balasubramanian S, Keshava SM, Satyanarayana K (2005) The 2001 dengue epidemic in Chennai. Indian J Pediatr 72: 919-923.

1344. Kabilan L, Balasubramanian S, Keshava SM, Thenmozhi V, Sekar G, et al. (2003) Dengue disease spectrum among infants in the 2001 dengue epidemic in Chennai, Tamil Nadu, India. J Clin Microbiol 41: 3919-3921.

1345. Kabilan L, Velayutham T, Sundaram B, Tewari SC, Natarajan A, et al. (2004) Field- and laboratory-based active dengue surveillance in Chennai, Tamil Nadu, India: observations before and during the 2001 dengue epidemic. Am J Infect Control 32: 391-396.

1346. Kabra SK, Jain Y, Pandey RM, Madhulika, Singhal T, et al. (1999) Dengue haemorrhagic fever in children in the 1996 Delhi epidemic. Trans R Soc Trop Med Hyg 93: 294-298.

1347. Kabra SK, Juneja R, Madhulika, Jain Y, Singhal T, et al. (1998) Myocardial dysfunction in children with dengue haemorrhagic fever. Natl Med J India 11: 59-61.

1348. Kabra SK, Verma IC, Arora NK, Jain Y, Kalra V (1992) Dengue haemorrhagic fever in children in Delhi. Bull World Health Organ 70: 105-108.

1349. Kalayanarooj S (2008) Choice of colloidal solutions in dengue hemorrhagic fever patients. J Med Assoc Thai 91 Suppl 3: S97-103.

1350. Kalayanarooj S, Gibbons RV, Vaughn D, Green S, Nisalak A, et al. (2007) Blood group AB is associated with increased risk for severe dengue disease in secondary infections. J Infect Dis 195: 1014-1017.

1351. Kalayanarooj S, Nimmannitya S (2003) Clinical presentations of dengue hemorrhagic fever in infants compared to children. J Med Assoc Thai 86 Suppl 3: S673-680.

1352. Kalayanarooj S, Nimmannitya S (2005) Is dengue severity related to nutritional status? Southeast Asian J Trop Med Public Health 36: 378-384.

1353. Kalayanarooj S, Rimal HS, Andjaparidze A, Vatcharasaevee V, Nisalak A, et al. (2007) Short report: Clinical intervention and molecular characteristics of a dengue hemorrhagic fever outbreak in Timor leste, 2005. Am J Trop Med Hyg 77: 534-537.

1354. Kalayanarooj S, Vaughn DW, Nimmannitya S, Green S, Suntayakorn S, et al. (1997) Early clinical and laboratory indicators of acute dengue illness. J Infect Dis 176: 313-321.

1355. Kalita J, Misra UK, Mahadevan A, Shankar SK (2005) Acute pure motor quadriplegia: is it dengue myositis? Electromyogr Clin Neurophysiol 45: 357-361.

1356. Kamath SR, Ranjit S (2006) Clinical features, complications and atypical manifestations of children with severe forms of dengue hemorrhagic fever in South India. Indian J Pediatr 73: 889-895.

1357. Kamble R, Peruvamba JN, Kovoor J, Ravishankar S, Kolar BS (2007) Bilateral thalamic involvement in dengue infection. Neurol India 55: 418-419.

1358. Kamil S, Mohamad NH, Narazah MY, Khan FA (2006) Dengue haemorrhagic fever with unusual prolonged thrombocytopaenia. Singapore Med J 47: 332-334.

1359. Kanakaratne N, Wahala WM, Messer WB, Tissera HA, Shahani A, et al. (2009) Severe dengue epidemics in Sri Lanka, 2003-2006. Emerg Infect Dis 15: 192-199.

1360. Kanesa-Thasan N, Edelman R, Tacket CO, Wasserman SS, Vaughn DW, et al. (2003) Phase 1 studies of Walter Reed Army Institute of Research candidate attenuated dengue vaccines: Selection of safe and immunogenic monovalent vaccines. Am J Trop Med Hyg 69: 17-23.

1361. Kangwanpong D, Bhamarapravati N, Lucia HL (1995) Diagnosing dengue virus infection in archived autopsy tissues by means of the in situ PCR method: a case report. Clin Diagn Virol 3: 165-172.

1362. Kankirawatana P, Chokephaibulkit K, Puthavathana P, Yoksan S, Apintanapong S, et al. (2000) Dengue infection presenting with central nervous system manifestation. J Child Neurol 15: 544-547.

1363. Kantachuvessiri A (2002) Dengue hemorrhagic fever in Thai society. Southeast Asian J Trop Med Public Health 33: 56-62.

1364. Kanungo S, Shukla D, Kim R (2008) Branch retinal artery occlusion secondary to dengue fever. Indian J Ophthalmol 56: 73-74.

1365. Kao CL, Wu MC, Chiu YH, Lin JL, Wu YC, et al. (2001) Flow cytometry compared with indirect immunofluorescence for rapid detection of dengue virus type 1 after amplification in tissue culture. J Clin Microbiol 39: 3672-3677.

1366. Kaplan JE, Eliason DA, Moore M, Sather GE, Schonberger LB, et al. (1983) Epidemiologic investigations of dengue infection in Mexico, 1980. Am J Epidemiol 117: 335-343.

1367. Kapoor H (2007) Ocular manifestations in dengue fever - Authors reply. Can J Ophthalmol 42: 755-756.

1368. Kapoor HK, Bhai S, John M, Xavier J (2006) Ocular manifestations of dengue fever in an East Indian epidemic. Can J Ophthalmol 41: 741-746.

1369. Karakus A, Banga N, Voorn GP, Meinders AJ (2007) Dengue shock syndrome and rhabdomyolysis. Neth J Med 65: 78-81.

1370. Karande S, Gandhi D, Kulkarni M, Bharadwaj R, Pol S, et al. (2005) Concurrent outbreak of leptospirosis and dengue in Mumbai, India, 2002. J Trop Pediatr 51: 174-181.

1371. Karp BE (1997) Dengue fever: a risk to travelers. Md Med J 46: 299-302.

1372. Karunatilaka DH, De Silva JR, Ranatunga PK, Gunasekara TM, Faizal MA, et al. (2007) Idiopathic purpura fulminans in dengue hemorrhagic fever. Indian J Med Sci 61: 471-473.

1373. Karunatilake H, Vithiya K, Arasalingam A, Malavan R, Kumara DS (2008) Acalculous cholecystitis and dengue fever. Ceylon Med J 53: 30.

1374. Kaul SM, Sharma RS, Sharma SN, Panigrahi N, Phukan PK, et al. (1998) Preventing dengue/dengue haemorrhagic fever outbreaks in the National Capital Territory of Delhi--the role of entomological surveillance. J Commun Dis 30: 187-192.

1375. Kaur H, Prabhakar H, Mathew P, Marshalla R, Arya M (1997) Dengue haemorrhagic fever outbreak in October-November 1996 in Ludhiana, Punjab, India. Indian J Med Res 106: 1-3.

1376. Kaushik RM, Varma A, Kaushik R, Gaur KJ (2007) Concurrent dengue and malaria due to Plasmodium falciparum and P. vivax. Trans R Soc Trop Med Hyg 101: 1048-1050.

1377. Kay BH, Nam VS, Tien TV, Yen NT, Phong TV, et al. (2002) Control of aedes vectors of dengue in three provinces of Vietnam by use of Mesocyclops (Copepoda) and community-based methods validated by entomologic, clinical, and serological surveillance. Am J Trop Med Hyg 66: 40-48.

1378. Kearney M, Porter WP, Williams C, Ritchie S, Hoffmann AA (2009) Integrating biophysical models and evolutionary theory to predict climatic impacts on species' ranges: the dengue mosquito Aedes aegypti in Australia. Funct Ecol 23: 528-538.

1379. Keelapang P, Sriburi R, Supasa S, Panyadee N, Songjaeng A, et al. (2004) Alterations of pr-M cleavage and virus export in pr-M junction chimeric dengue viruses. J Virol 78: 2367-2381.

1380. Kelly N (1997) DENGUE - BARBADOS (06). PROMED.

1381. Kemp A, Jupp PG (1991) Potential for dengue in South Africa: mosquito ecology with particular reference to Aedes aegypti. J Am Mosq Control Assoc 7: 574-583.

1382. Kenyon G (1999) Scientists try new strategy to eradicate dengue fever. Br Med J 318: 555.

1383. Kerdpanich A, Watanaveeradej V, Samakoses R, Chumnanvanakij S, Chulyamitporn T, et al. (2001) Perinatal dengue infection. Southeast Asian J Trop Med Public Health 32: 488-493.

1384. Kerschner JH, Vorndam AV, Monath TP, Trent DW (1986) Genetic and epidemiological studies of dengue type 2 viruses by hybridization using synthetic deoxyoligonucleotides as probes. J Gen Virol 67 ( Pt 12): 2645-2661.

1385. Khan E, Hasan R, Mehraj V, Nasir A, Siddiqui J, et al. (2008) Co-circulations of two genotypes of dengue virus in 2006 out-break of dengue hemorrhagic fever in Karachi, Pakistan. J Clin Virol 43: 176-179.

1386. Khan E, Mehraj V, Nasir A, Khan NA, Billoo B, et al. (2009) Evaluation of two ELISA assay kits against RT-PCR for diagnosis of dengue virus infection in a hospital setting in Karachi, Pakistan. JPMA J Pak Med Assoc 59: 390-394.

1387. Khan E, Siddiqui J, Shakoor S, Mehraj V, Jamil B, et al. (2007) Dengue outbreak in Karachi, Pakistan, 2006: experience at a tertiary care center. Trans R Soc Trop Med Hyg 101: 1114-1119.

1388. Khan NA, Azhar EI, El-Fiky S, Madani HH, Abuljadial MA, et al. (2008) Clinical profile and outcome of hospitalized patients during first outbreak of dengue in Makkah, Saudi Arabia. Acta Trop 105: 39-44.

1389. Khanam S, Etemad B, Khanna N, Swaminathan S (2006) Induction of neutralizing antibodies specific to dengue virus serotypes 2 and 4 by a bivalent antigen composed of linked envelope domains III of these two serotypes. Am J Trop Med Hyg 74: 266-277.

1390. Khawsak P, Phantana S, Chansiri K (2003) Determination of dengue virus serotypes in Thailand using PCR based method. Southeast Asian J Trop Med Public Health 34: 781-785.

1391. Khilnani P, Sarma D, Singh R, Uttam R, Rajdev S, et al. (2004) Demographic profile and outcome analysis of a tertiary level pediatric intensive care unit. Indian J Pediatr 71: 587-591.

1392. Khilnani P, Sarma D, Zimmerman J (2006) Epidemiology and peculiarities of pediatric multiple organ dysfunction syndrome in New Delhi, India. Intensive Care Med 32: 1856-1862.

1393. Kho LK, Sumarmo, Wulur H, Jahja EC, Gubler DJ (1981) Dengue hemorrhagic fever accompanied by encephalopathy in Jakarta. Southeast Asian J Trop Med Public Health 12: 83-86.

1394. Khongphafthanayothin A, Lertsapicharoen P, Supachokchaiwattana P, La-Orkhun V, Khumtonvong A, et al. (2007) Myocardial depression in dengue hemorrhagic fever: Prevalence and clinical description. Pediatr Crit Care Med 8: 524-529.

1395. Khongphatthanayothin A, Lertsapcharoen P, Supachokchaiwattana P, Satupan P, Thongchaiprasit K, et al. (2005) Hepatosplanchnic circulatory dysfunction in acute hepatic infection: The case of dengue hemorrhagic fever. Shock 24: 407-411.

1396. Khongphatthanayothin A, Phumaphuti P, Thongchalprasit K, Poovorawan Y (2006) Serum levels of sICAM-1 and sE-Selectin in patients with dengue virus infection. Jpn J Infect Dis 59: 186-188.

1397. Khor BS, Liu JW, Lee IK, Yang KD (2006) Dengue hemorrhagic fever patients with acute abdomen: Clinical experience of 1.4 cases. Am J Trop Med Hyg 74: 901-904.

1398. Khun S, Manderson L (2007) Community and School-Based Health Education for Dengue Control in Rural Cambodia: A Process Evaluation. PLoS Negl Trop Dis 1: -.

1399. Khun S, Manderson L (2008) Poverty, user fees and ability to pay for health care for children with suspected dengue in rural Cambodia. International Journal for Equity in Health 7: 10.

1400. Kiedrzynski T (1997) DENGUE - PACIFIC AREA (04). In: Body PPHSC, Programme SPC-CH, editors: PROMED.

1401. Kiedrzynski T (1998) DENGUE - NEW CALEDONIA (02). In: Programme SCH, editor. Pacific Public Health Surveillance Coordinating Body: PROMED.

1402. Kiedrzynski T (1998) DENGUE - NEW CALEDONIA (04). In: Programme SCH, editor. Pacific Public Health Surveillance Network ed: PROMED.

1403. Kiedrzynski T (1998) DENGUE - SAIPAN (04). In: Programme SCH, editor: PROMED.

1404. Kiedrzynski T (1998) DENGUE - TONGA (03). In: Network PPHS, editor. WHO CLO Tonga ed: PROMED.

1405. Kiedrzynski T (1998) DENGUE 2 - NEW CALEDONIA (03). In: Network PPHS, editor: PROMED.

1406. Kiedrzynski T (1998) DENGUE - TONGA (02). In: Programme SCH, editor. Pacific Public Health Surveillance Network

PACNET@LISTSERV.SPC.ORG.NC ed: PROMED.

1407. Kiedrzynski T (1998) DENGUE - NEW CALEDONIA (03). In: Programme SCH, editor: PROMED.

1408. Kiedrzynski T (1998) DENGUE - WALLIS AND FUTUNA (02). In: Dr Bezannier Gerard CMO, Department of Hygiene,, Control. PaMED, editors. Pacific Public Health Surveillance Network ed: PROMED.

1409. Kiedrzynski T (1998) DENGUE - NEW CALEDONIA (05). In: Network PPHS, editor: PROMED.

1410. Kiedrzynski T (1998) DENGUE - WALLIS & FUTUNA. In: Programme SCH, editor. Pacific Public Health Surveillance Network ed: PROMED.

1411. Kiedrzynski T (1999) DENGUE/DHF - NEW CALEDONIA (02). In: Network PPHS, editor: PROMED.

1412. Kiedrzynski T (1999) DENGUE/DHF - NEW CALEDONIA. In: Network PPHS, editor: PROMED.

1413. Kielian M (2006) Class II virus membrane fusion proteins. Virology 344: 38-47.

1414. King CC, Chao DY, Chien LJ, Chang GJJ, Lin TH, et al. (2008) Comparative analysis of full genomic sequences among different genotypes of dengue virus type 3. Virol J 5: -.

1415. Kinney RM, Huang CYH, Rose BC, Kroeker AD, Dreher TW, et al. (2005) Inhibition of dengue virus serotypes 1 to 4 in Vero cell cultures with morpholino oligomers. J Virol 79: 5116-5128.

1416. Kitayaporn D, Singhasivanon P, Vasuvat C (1989) Age-adjusted dengue haemorrhagic fever morbidity in Thailand 1983-1987. Southeast Asian J Trop Med Public Health 20: 195-200.

1417. Kitchener S (2000) DENGUE, MILITARY EXPERIENCE - AUSTRALIA EX EAST TIMOR. (Queensland): PROMED.

1418. Kitchener S, Leggat PA, Brennan L, McCall B (2002) Importation of dengue by soldiers returning from East Timor to north Queensland, Australia. J Travel Med 9: 180-183.

1419. Kittayapong P, Chansang U, Chansang C, Bhumiratana A (2006) Community participation and appropriate technologies for dengue vector control at transmission foci in Thailand. J Am Mosq Control Assoc 22: 538-546.

1420. Kittayapong P, Strickman D (1993) Distribution of container-inhabiting Aedes larvae (Diptera: Culicidae) at a dengue focus in Thailand. J Med Entomol 30: 601-606.

1421. Kittigul L, Meethien N, Sujirarat D, Kittigul C, Vasanavat S (1997) Comparison of dengue virus antigens in sera and peripheral blood mononuclear cells from dengue infected patients. Asian Pac J Allergy Immunol 15: 187-191.

1422. Kittigul L, Pitakarnjanakul P, Sujirarat D, Siripanichgon K (2007) The differences of clinical manifestations and laboratory findings in children and adults with dengue virus infection. J Clin Virol 39: 76-81.

1423. Kittigul L, Suankeow K, Sujirarat D, Yoksan S (2003) Dengue hemorrhagic fever: knowledge, attitude and practice in Ang Thong Province, Thailand. Southeast Asian J Trop Med Public Health 34: 385-392.

1424. Kittisupamongkol W (2008) Relative bradycardia in dengue fever. Hong Kong Med J 14: 334.

1425. Klassen P, Biesalski HK, Mazariegos M, Solomons NW, Furst P (2004) Classic dengue fever affects levels of circulating antioxidants. Nutrition 20: 542-547.

1426. Klassen P, Furst P, Schulz C, Mazariegos M, Solomons NW (2001) Plasma free amino acid concentrations in healthy Guatemalan adults and in patients with classic dengue. Am J Clin Nutr 73: 647-652.

1427. Klassen P, Mazariegos M, Solomons NW, Furst P (2000) The pharmacokinetic responses of humans to 20 g of alanyl-glutamine dipeptide differ with the dosing protocol but not with gastric acidity or in patients with acute Dengue fever. J Nutr 130: 177-182.

1428. Klungthong C, Gibbons RV, Thaisomboonsuk B, Nisalak A, Kalayanarooj S, et al. (2007) Dengue virus detection using whole blood for reverse transcriptase PCR and virus isolation. J Clin Microbiol 45: 2480-2485.

1429. Klungthong C, Zhang C, Mammen MP, Jr., Ubol S, Holmes EC (2004) The molecular epidemiology of dengue virus serotype 4 in Bangkok, Thailand. Virology 329: 168-179.

1430. Kmietowicz Z (2007) Dengue fever epidemic in Cambodia affects 17 000. Br Med J 335: 65-65.

1431. Kmietowicz Z (2007) Cambodia faces dengue fever epidemic. BMJ 335: 65.

1432. Knio KN, Baydoun E, Tawk R, Nuwayri-Salti N (2000) Isoenzyme characterization of Leishmania isolates from Lebanon and Syria. Am J Trop Med Hyg 63: 43-47.

1433. Knox TB, Kay BH, Hall RA, Ryan PA (2003) Enhanced vector competence of Aedes aegypti (Diptera: Culicidae) from the Torres Strait compared with mainland Australia for dengue 2 and 4 viruses. J Med Entomol 40: 950-956.

1434. Knudsenb B (2000) DENGUE - BANGLADESH: BACKGROUND. PROMED.

1435. Ko YC (1989) [Epidemiology of dengue fever in Taiwan]. Gaoxiong Yi Xue Ke Xue Za Zhi 5: 1-11.

1436. Ko YC, Chen JW, Chang IC (1989) Attack rate of dengue-like illness among teachers in Kaohsiung City, 1988. Gaoxiong Yi Xue Ke Xue Za Zhi 5: 129-131.

1437. Ko YC, Chen MJ, Yeh SM (1992) The predisposing and protective factors against dengue virus transmission by mosquito vector. Am J Epidemiol 136: 214-220.

1438. Kobayashi N, Thayan R, Sugimoto C, Oda K, Saat Z, et al. (1999) Type-3 dengue viruses responsible for the dengue epidemic in Malaysia during 1993-1994. Am J Trop Med Hyg 60: 904-909.

1439. Kochel T, Aguilar P, Felices V, Comach G, Cruz C, et al. (2008) Molecular epidemiology of dengue virus type 3 in Northern South America: 2000-2005. Infect Genet Evol 8: 682-688.

1440. Kochel TJ, Watts DM, Gozalo AS, Ewing DF, Porter KR, et al. (2005) Cross-serotype neutralization of dengue virus in Aotus nancymae monkeys. J Infect Dis 191: 1000-1004.

1441. Kochel TJ, Watts DM, Halstead SB, Hayes CG, Espinoza A, et al. (2002) Effect of dengue-1 antibodies on American dengue-2 viral infection and dengue haemorrhagic fever. Lancet 360: 310-312.

1442. Kohli U, Saharan S, Lodha R, Kabra SK (2008) Persistent thrombocytopenia following dengue shock syndrome. Indian J Pediatr 75: 82-83.

1443. Kohli U, Sahu J, Lodha R, Agarwal N, Ray R (2007) Invasive nosocomial aspergillosis associated with heart failure and complete heart block following recovery from dengue shock syndrome. Pediatr Crit Care Med 8: 389-391.

1444. Kongsomboon K, Singhasivanon P, Kaewkungwal J, Nimmannitya S, Mammen MP, Jr., et al. (2004) Temporal trends of dengue fever/dengue hemorrhagic fever in Bangkok, Thailand from 1981 to 2000: an age-period-cohort analysis. Southeast Asian J Trop Med Public Health 35: 913-917.

1445. Koopman JS, Prevots DR, Vaca Marin MA, Gomez Dantes H, Zarate Aquino ML, et al. (1991) Determinants and predictors of dengue infection in Mexico. Am J Epidemiol 133: 1168-1178.

1446. Koraka P, Murgue B, Deparis X, van Gorp ECM, Setiati TE, et al. (2004) Elevation of soluble VCAM-1 plasma levels in children with acute dengue virus infection of varying severity. J Med Virol 72: 445-450.

1447. Koraka P, Williams MM, Djamiatun K, Setiati TE, van Batenburg FHD, et al. (2009) RNA secondary structures in the proximal 3 ' UTR of Indonesian Dengue 1 virus strains. Virus Res 142: 213-216.

1448. Kouri G (1998) DENGUE/DHF - CUBA (12). In: Center WPC, editor. Havana: PROMED.

1449. Kouri G (2006) El dengue, un problema creciente de salud en las Américas. Rev Panam Salud Publica 19: 143-145.

1450. Kouri G, Guzman MG, Valdes L, Carbonel I, del Rosario D, et al. (1998) Reemergence of dengue in Cuba: a 1997 epidemic in Santiago de Cuba. Emerg Infect Dis 4: 89-92.

1451. Kouri G, Valdez M, Arguello L, Guzman MG, Valdes L, et al. (1991) Epidemia de dengue en Nicaragua. Rev Inst Med Trop Sao Paulo 33: 365-371.

1452. Kouri GP, Guzman MG, Bravo JR (1987) Why dengue haemorrhagic fever in Cuba? 2. An integral analysis. Trans R Soc Trop Med Hyg 81: 821-823.

1453. Kouri GP, Guzman MG, Bravo JR, Triana C (1989) Dengue haemorrhagic fever/dengue shock syndrome: lessons from the Cuban epidemic, 1981. Bull World Health Organ 67: 375-380.

1454. Kow CY, Koon LL, Yin PF (2001) Detection of dengue viruses in field caught male Aedes aegypti and Aedes albopictus (Diptera: Culicidae) in Singapore by type-specific PCR. J Med Entomol 38: 475-479.

1455. Kowitdamrong E, Thammaborvorn R, Semboonlor L, Mungmee V, Bhattarakosol P (2001) Detection of dengue HI and IgM antibody: is it diagnostically useful? when and how? J Med Assoc Thai 84 Suppl 1: S148-154.

1456. Krippner R, Hanisch G, Kretschmer H (1990) Denguefieber mit hämorrhagischen Manifestationen nach Thailandaufenthalt. Dtsch Med Wochenschr 115: 858-862.

1457. Krishnamurti C, Kalayanarooj S, Cutting MA, Peat RA, Rothwell SW, et al. (2001) Mechanisms of hemorrhage in dengue without circulatory collapse. Am J Trop Med Hyg 65: 840-847.

1458. Kroeger A, Dehlinger U, Burkhardt G, Atehortua W, Anaya H, et al. (1995) Community based dengue control in Columbia: people's knowledge and practice and the potential contribution of the biological larvicide Bti (Bacillus thuringiensis israelensis). Trop Med Parasitol 46: 241-246.

1459. Kuan G, Gordon A, Aviles W, Ortega O, Hammond SN, et al. (2009) The Nicaraguan Pediatric Dengue Cohort Study: Study Design, Methods, Use of Information Technology, and Extension to Other Infectious Diseases. Am J Epidemiol 170: 120-129.

1460. Kuberski T, Rosen L, Reed D, Mataika J (1977) Clinical and laboratory observations on patients with primary and secondary dengue type 1 infections with hemorrhagic manifestations in Fiji. Am J Trop Med Hyg 26: 775-783.

1461. Kukreti H, Chaudhary A, Rautela RS, Anand R, Mittal V, et al. (2008) Emergence of an independent lineage of dengue virus type 1 (DENV-1) and its co-circulation with predominant DENV-3 during the 2006 dengue fever outbreak in Delhi. Int J Infect Dis 12: 542-549.

1462. Kukreti H, Dash PK, Parida M, Chaudhary A, Saxena P, et al. (2009) Phylogenetic studies reveal existence of multiple lineages of a single genotype of DENV-1 (genotype III) in India during 1956-2007. Virol J 6: -.

1463. Kularatne SA, Gawarammana IB, Kumarasiri PR (2005) Epidemiology, clinical features, laboratory investigations and early diagnosis of dengue fever in adults: a descriptive study in Sri Lanka. Southeast Asian J Trop Med Public Health 36: 686-692.

1464. Kularatne SA, Pathirage MM, Kumarasiri PV, Gunasena S, Mahindawanse SI (2007) Cardiac complications of a dengue fever outbreak in Sri Lanka, 2005. Trans R Soc Trop Med Hyg 101: 804-808.

1465. Kularatne SAM, Gihan MC, Weerasinghe SC, Gunasena S (2009) Concurrent outbreaks of Chikungunya and Dengue fever in Kandy, Sri Lanka, 2006-07: a comparative analysis of clinical and laboratory features. Postgrad Med J 85: 342-346.

1466. Kularatne SAM, Pathirage MMK, Gunasena S (2008) A case series of dengue fever with altered consciousness and electroencephalogram changes in Sri Lanka. Trans R Soc Trop Med Hyg 102: 1053-1054.

1467. Kumar A, Sharma SK, Padbidri VS, Thakare JP, Jain DC, et al. (2001) An outbreak of dengue fever in rural areas of northern India. J Commun Dis 33: 274-281.

1468. Kumar J, Kumar A, Gupta S, Jain D (2007) Dengue haemorrhagic fever: an unusual cause of intracranial haemorrhage. Journal of Neurology Neurosurgery and Psychiatry 78: 253-253.

1469. Kumar J, Kumar A, Gupta S, Jain D (2007) Neurological picture. Dengue haemorrhagic fever: an unusual cause of intracranial haemorrhage. J Neurol Neurosurg Psychiatry 78: 253.

1470. Kumar R, Prakash O, Sharma BS (2008) Dengue Hemorrhagic Fever: A Rare Presentation as Atypical Acute Subdural Hematoma. Pediatr Neurosurg 44: 490-492.

1471. Kumar R, Prakash O, Sharma BS (2009) Intracranial hemorrhage in dengue fever: management and outcome A series of 5 cases and review of literature. Surg Neurol.

1472. Kumar R, Tripathi P, Tripathi S, Kanodia A, Pant S, et al. (2008) Prevalence and clinical differentiation of dengue fever in children in northern India. Infection 36: 444-449.

1473. Kumar R, Tripathi P, Tripathi S, Kanodia A, Venkatesh V (2008) Prevalence of dengue infection in north Indian children with acute hepatic failure. Ann Hepatol 7: 59-62.

1474. Kumar R, Tripathi S, Tambe JJ, Arora V, Srivastava A, et al. (2008) Dengue encephalopathy in children in Northern India: Clinical features and comparison with non dengue. J Neurol Sci 269: 41-48.

1475. Kumar V, Ghosh B, Raina UK, Goel N (2009) Bilateral periorbital ecchymosis in a case with dengue fever. Indian J Ophthalmol 57: 242-243.

1476. Kumarasamy V (2006) Dengue fever in Malaysia: time for review? Med J Malaysia 61: 1-3.

1477. Kumarasamy V, Chua SK, Hassan Z, Wahab AH, Chem YK, et al. (2007) Evaluating the sensitivity of a commercial dengue NS1 antigen-capture ELISA for early diagnosis of acute dengue virus infection. Singapore Med J 48: 669-673.

1478. Kumaria R, Chakravarti A (2005) Molecular detection and serotypic characterization of dengue viruses by single-tube multiplex reverse transcriptase-polymerase chain reaction. Diagn Microbiol Infect Dis 52: 311-316.

1479. Kuniholm MH, Wolfe ND, Huang CYH, Mpoudi-Ngole E, Tamoufe U, et al. (2006) Seroprevalence and distribution of Flaviviridae, Togaviridae, and Bunyaviridae arboviral infections in rural Cameroonian adults. Am J Trop Med Hyg 74: 1078-1083.

1480. Kuno G (1993) Computer literature searches on dengue. Bull World Health Organ 71: 165-172.

1481. Kuno G (2007) Research on dengue and dengue-like illness in East Asia and the Western Pacific during the First Half of the 20th century. Rev Med Virol 17: 327-341.

1482. Kuno G, Gomez I, Gubler DJ (1991) An ELISA procedure for the diagnosis of dengue infections. J Virol Methods 33: 101-113.

1483. Kuno G, Gubler DJ, Oliver A (1993) Use of 'original antigenic sin' theory to determine the serotypes of previous dengue infections. Trans R Soc Trop Med Hyg 87: 103-105.

1484. Kuno G, Vorndam AV, Gubler DJ, Gomez I (1990) Study of anti-dengue NS1 antibody by western blot. J Med Virol 32: 102-108.

1485. Kuo MC, Chang JM, Lu PL, Chiu YW, Chen HC, et al. (2007) Case report: Difficulty in diagnosis and treatment of dengue hemorrhagic fever in patients with chronic renal failure: Report of three cases of mortality. Am J Trop Med Hyg 76: 752-756.

1486. Kuo MC, Lu PL, Chang JM, Lin MY, Tsai JJ, et al. (2008) Impact of renal failure on the outcome of dengue viral infection. Clin J Am Soc Nephrol 3: 1350-1356.

1487. Kurukumbi M, Wali JP, Broor S, Aggarwal P, Seth P, et al. (2001) Seroepidemiology and active surveillance of dengue fever/dengue haemorrhagic fever in Delhi. Indian J Med Sci 55: 149-156.

1488. Kwan WH, Helt AM, Maranon C, Barbaroux JB, Hosmalin A, et al. (2005) Dendritic cell precursors are permissive to dengue virus and human immunodeficiency virus infection. J Virol 79: 7291-7299.

1489. Kyaw Zin T, Khin Mar A, Soe T, Than S, Hasebe F, et al. (1995) Genotype determination of three dengue type 2 virus strains from Myanmar by sequencing E/NSI gene junction. Southeast Asian J Trop Med Public Health 26: 664-668.

1490. Laferl H, Szell M, Bischof E, Wenisch C (2006) Imported dengue fever in Austria 1990-2005. Travel Med Infect Dis 4: 319-323.

1491. Lahiri M, Fisher D, Tambyah PA (2008) Dengue mortality: reassessing the risks in transition countries. Trans R Soc Trop Med Hyg 102: 1011-1016.

1492. Lai CJ, Goncalvez AP, Men R, Wernly C, Donau O, et al. (2007) Epitope determinants of a chimpanzee dengue virus type 4 (DENV-4) -neutralizing antibody and protection against DENV-4 challenge in mice and rhesus monkeys by passively transferred humanized antibody. J Virol 81: 12766-12774.

1493. Lai CY, Tsai WY, Lin SR, Kao CL, Hu HP, et al. (2008) Antibodies to envelope glycoprotein of dengue virus during the natural course of infection are predominantly cross-reactive and recognize epitopes containing highly conserved residues at the fusion loop of domain II. J Virol 82: 6631-6643.

1494. Lai I (2006) Dengue/DHF update 2006 (40) - Taiwan. PROMED: Promed.

1495. Lai PC, Lee SS, Kao CH, Chen YS, Huang CK, et al. (2004) Characteristics of a dengue hemorrhagic fever outbreak in 2001 in Kaohsiung. J Microbiol Immunol Infect 37: 266-270.

1496. Lai TYY, Mohamed S, Chan WM, Lai RYK, Lam DSC (2007) Multifocal electroretinography in dengue fever-associated maculopathy. Br J Ophthalmol 91: 1084-1085.

1497. Lai YL, Chung YK, Tan HC, Yap HF, Yap G, et al. (2007) Cost-effective real-time reverse transcriptase PCR (RT-PCR) to screen for dengue virus followed by rapid single-tube multiplex RT-PCR for serotyping of the virus. J Clin Microbiol 45: 935-941.

1498. Laille M, Deubel V, Sainte-Marie FF (1991) Demonstration of concurrent dengue 1 and dengue 3 infection in six patients by the polymerase chain reaction. J Med Virol 34: 51-54.

1499. Laille M, Fauran P, Moreau JP, Flye Sainte-Marie F (1990) Reapparition de la dengue en Nouvelle-Caledonie. Bull Soc Pathol Exot Filiales 83: 591-595.

1500. Laille M, Huerre M, Dubourdieu H, Flye Sainte Marie F (1993) Epidemie de dengue en Nouvelle-Caledonie. Bull Soc Pathol Exot Filiales 86: 442-449.

1501. Laille M, Roche C (2004) Comparison of dengue-1 virus envelope glycoprotein gene sequences from French Polynesia. Am J Trop Med Hyg 71: 478-484.

1502. Laird M (1984) [Integrated vector control methods and the threat of dengue hemorrhagic fever in the tropical zone of the Pacific Ocean]. Parazitologiia 18: 99-105.

1503. Lal M, Aggarwal A, Oberoi A (2007) Dengue fever--an emerging viral fever in Ludhiana, North India. Indian J Public Health 51: 198-199.

1504. Lall R, Dhanda V (1996) Dengue haemorrhagic fever and the dengue shock syndrome in India. Natl Med J India 9: 20-23.

1505. Lam Sai Kit K (2000) DENGUE/DHF: UPDATES, 7 NOV 2000 [Malaysia]. The Sun newspaper, Fri 27 Oct 2000 [edited] ed: PROMED.

1506. Lam SK (1996) DENGUE/DENGUE HEMORRHAGIC FEVER - MALAYSIA. In: DF/DHF WCCf, editor. Kuala Lumpur: PROMED.

1507. Lambeth CR, White LJ, Johnston RE, de Silva AM (2005) Flow cytometry-based assay for titrating dengue virus. J Clin Microbiol 43: 3267-3272.

1508. Lamot P (2002) DENGUE - CUBA: REQUEST FOR INFORMATION. In: ProMED-mail, editor. CubaNet 7 Oct 2002 [in Spanish, summarized by Mod.JW; edited] ed: PROMED.

1509. Lan NTP, Kikuchi M, Huong VTQ, Ha DQ, Thuy TT, et al. (2008) Protective and Enhancing HLA Alleles, HLA-DRB1*0901 and HLA-A*24, for Severe Forms of Dengue Virus Infection, Dengue Hemorrhagic Fever and Dengue Shock Syndrome. PLoS Negl Trop Dis 2: -.

1510. Lanciotti RS, Kosoy OL, Laven JJ, Velez JO, Lambert AJ, et al. (2008) Genetic and serologic properties of zika virus associated with an epidemic, Yap State, Micronesia, 2007. Emerg Infect Dis 14: 1232-1239.

1511. Laoprasopwattana K, Libraty DH, Endy TP, Nisalak A, Chunsuttiwat S, et al. (2007) Antibody-dependent cellular cytotoxicity mediated by plasma obtained before secondary dengue virus infections: Potential involvement in early control of viral replication. J Infect Dis 195: 1108-1116.

1512. Laoprasopwattana K, Libraty DH, Endy TP, Nisalak A, Chunsuttiwat S, et al. (2005) Dengue virus (DV) enhancing antibody activity in preillness plasma does not predict subsequent disease severity or viremia in secondary DV infection. J Infect Dis 192: 510-519.

1513. Lapphra K, Sangcharaswichai A, Chokephaibulkit K, Tiengrim S, Piriyakarnsakul W, et al. (2008) Evaluation of an NS1 antigen detection for diagnosis of acute dengue infection in patients with acute febrile illness. Diagn Microbiol Infect Dis 60: 387-391.

1514. Lardeux F, Riviere F, Sechan Y, Loncke S (2002) Control of the Aedes vectors of the dengue viruses and Wuchereria bancrofti: the French Polynesian experience. Ann Trop Med Parasitol 96 Suppl 2: S105-116.

1515. Larghi OP (2000) DENGUE - ARGENTINA, BRAZIL, PARAGUAY: ALERT. Buenos Aires: PROMED.

1516. LaRocque RC, Breiman RF, Ari MD, Morey RE, Janan FA, et al. (2005) Leptospirosis during Dengue outbreak, Bangladesh. Emerg Infect Dis 11: 766-769.

1517. Larreal Y (2008) Indicación de hemoderivados en dengue. Invest Clin 49: 285-287.

1518. Larreal Y, Valero N, Estevez J, Reyes I, Maldonado M, et al. (2005) Alteraciones hepiiticas en pacientes con dengue. Invest Clin 46: 169-178.

1519. Larru Martinez B, Quiroz E, Bellon JM, Esquivel R, Nieto Guevara J, et al. (2006) Dengue pediátrico en Panamá. An Pediatr 64: 517-522.

1520. Lassalle C, Grizeau P, Isautier H, Bagnis O, Michault A, et al. (1998) Surveillance épidémiologique de la grippe et de la dengue. La Réunion, 1996. Bull Soc Pathol Exot Filiales 91: 61-63.

1521. Lateef A, Fisher DA, Tambyah PA (2007) Dengue and relative bradycardia. Emerg Infect Dis 13: 650-651.

1522. Lau FY, Cheng YM, Chui E (2008) Apheresis platelet for dengue hemorrhagic fever supportive therapy. Vox Sang 95: 82-82.

1523. Laude A, Chlebicki MP, Ang B, Barkham T (2007) Maculopathy and dengue. Emerg Infect Dis 13: 347-348.

1524. Laur F, Murgue B, Deparis X, Roche C, Cassar O, et al. (1998) Plasma levels of tumour necrosis factor alpha and transforming growth factor beta-1 in children with dengue 2 virus infection in French Polynesia. Trans R Soc Trop Med Hyg 92: 654-656.

1525. Laurence B, Harold M, Thierry D (2008) Ocular complications of dengue fever. Ophthalmology 115: 1100-1101.

1526. Lawn SD, Tilley R, Lloyd G, Finlayson C, Tolley H, et al. (2003) Dengue hemorrhagic fever with fulminant hepatic failure in an immigrant returning to Bangladesh. Clin Infect Dis 37: e1-4.

1527. Lawuyan S (1997) DENGUE - INDONESIA (EAST JAVA) (03): REQUEST FOR INFO. In: Control CD, Service SMH, editors. Surabaya: PROMED.

1528. Lay JG, Lin ZH, Yap KH, Wu PC, Su HJ (2006) Temperature variability and spatial hotspots of dengue fever occurrence in Taiwan. Epidemiology 17: S485-S485.

1529. Lazaro-Olan L, Mellado-Sanchez G, Garcia-Cordero J, Escobar-Gutierrez A, Santos-Argumedo L, et al. (2008) Analysis of antibody response in human dengue patients from the Mexican coast using recombinant antigens. Vector-Borne and Zoonotic Diseases 8: 69-79.

1530. Le Gonidec G, Quene JP, Fauran P (1982) Sur Une Epidemie de dengue de type 4 a thio, Nouvelle-Caledonie. Aspects Epidemiologiques et cliniques. Bull Soc Pathol Exot Filiales 75: 141-150.

1531. Lee A (1997) DENGUE - INDONESIA (EAST JAVA). In: ProMED-mail, editor. Media sources ed: PROMED.

1532. Lee A (1997) DENGUE HEMORRHAGIC FEVER - INDONESIA (KALIMANTAN). In: ProMED-mail, editor: PROMED.

1533. Lee CH, Teo C, Low AF (2009) Fulminant dengue myocarditis masquerading as acute myocardial infarction. Int J Cardiol 136: E69-E71.

1534. Lee CY, Seet RC, Huang SH, Long LH, Halliwell B (2008) Different patterns of oxidized lipid products in plasma and urine of dengue fever, stroke and Parkinsons disease patients. Cautions in the use of biomarkers of oxidative stress. Antioxid Redox Signal.

1535. Lee D, Moore CG (1973) Mosquito Studies during an Interepidemic Outbreak of Dengue in Puerto-Rico. Mosq News 33: 506-509.

1536. Lee E, Gubler DJ, Weir RC, Dalgarno L (1993) Genetic and biological differentiation of dengue 3 isolates obtained from clinical cases in Java, Indonesia, 1976-1978. Arch Virol 133: 113-125.

1537. Lee E, Pavy M, Young N, Freeman C, Lobigs M (2006) Antiviral effect of the heparan sulfate mimetic, PI-88, against dengue and encephalitic flaviviruses. Antivir Res 69: 31-38.

1538. Lee IK, Khor BS, Kee KM, Yang KD, Liu JW (2007) Hyperlipasemia/pancreatitis in adults with dengue hemorrhagic fever. Pancreas 35: 381-382.

1539. Lee IK, Liu JW, Yang KD (2005) Clinical characteristics and risk factors for concurrent bacteremia in adults with dengue hemorrhagic fever. Am J Trop Med Hyg 72: 221-226.

1540. Lee IK, Liu JW, Yang KD (2008) Clinical and laboratory characteristics and risk factors for fatality in elderly patients with dengue hemorrhagic fever. Am J Trop Med Hyg 79: 149-153.

1541. Lee IK, Liu JW, Yang KD (2009) Clinical Characteristics, Risk Factors, and Outcomes in Adults Experiencing Dengue Hemorrhagic Fever Complicated with Acute Renal Failure. Am J Trop Med Hyg 80: 651-655.

1542. Lee MS, Hwang KP, Chen TC, Lu PL, Chen TP (2006) Clinical characteristics of dengue and dengue hemorrhagic fever in a medical center of southern Taiwan during the 2002 epidemic. J Microbiol Immunol Infect 39: 121-129.

1543. Lee V, Lye D, Sun Y, Fernandez G, Ong A, et al. (2007) Clinical features of dengue infections, and predictors of dengue haemorrhagic fever in Singapore. Int J Antimicrob Agents 29: S49-S49.

1544. Lee VJ, Lye DC, Sun Y, Leo YS (2009) Decision tree algorithm in deciding hospitalization for adult patients with dengue haemorrhagic fever in Singapore. Trop Med Int Health 14: 1154-1159.

1545. Lee VJ, Lye DCB, Sun Y, Fernandez G, Ong A, et al. (2008) Predictive value of simple clinical and laboratory variables for dengue hemorrhagic fever in adults. J Clin Virol 42: 34-39.

1546. Lee YR, Huang KJ, Lei HY, Chen SH, Lin YS, et al. (2005) Suckling mice were used to detect infectious dengue-2 viruses by intracerebral injection of the full-length RNA transcript. Intervirology 48: 161-166.

1547. Lee YR, Liu MT, Lei HY, Liu CC, Wu JM, et al. (2006) MCP-1, a highly expressed chemokine in dengue haemorrhagic fever/dengue shock syndrome patients, may cause permeability change, possibly through reduced tight junctions of vascular endothelium cells. J Gen Virol 87: 3623-3630.

1548. Lee YY, Safhan MNF, Abd AD, Tee HP (2008) Liver function derangement and bleeding morbidity in dengue hemorrhagic fever (DHF). J Gastroenterol Hepatol 23: A36-A37.

1549. Leelarasamee A, Chupaprawan C, Chenchittikul M, Udompanthurat S (2004) Etiologies of acute undifferentiated febrile illness in Thailand. J Med Assoc Thai 87: 464-472.

1550. Leggat PA (2007) Assessment of febrile illness in the returned traveller. Aust Fam Physician 36: 328-333.

1551. Lenzi Mde F, Coura LC (2004) Prevenção da dengue: a informação em foco. Rev Soc Bras Med Trop 37: 343-350.

1552. Lenzi MF, Camillo-Coura L, Grault CE, Val MB (2000) Estudo do dengue em área urbana favelizada do Rio de Janeiro: considerações iniciais. Cad Saude Publica 16: 851-856.

1553. Leparc-Goffart I, Baragatti M, Temmam S, Tuiskunen A, Moureau G, et al. (2009) Development and validation of real-time one-step reverse transcription-PCR for the detection and typing of dengue viruses. J Clin Virol 45: 61-66.

1554. Lesh W (2005) DENGUE/DHF UPDATE 2005 (06). PROMED.

1555. Levett P (1995) LEPTOSPIROSIS & DENGUE - BARBADOS. In: UWI CH, editor. (Barbados): PROMED.

1556. Levett P (1995) DENGUE - BARBADOS (2). In: Barbados U, editor. (Barbados): PROMED.

1557. Levett P (1995) DENGUE - BARBADOS. PROMED.

1558. Levett P (1997) DENGUE - BARBADOS (08). <http://www.sunbeach.net/comp/lepto/pnllepto.htm> ed. St.Michael (Barbados): PROMED.

1559. Levett PN, Branch SL, Edwards CN (2000) Detection of dengue infection in patients investigated for leptospirosis in Barbados. Am J Trop Med Hyg 62: 112-114.

1560. Levett PN, Branch SL, Edwards CN (2000) Detection of dengue infection in patients investigated for leptospirosis in Barbados. The American Journal of Tropical Medicine and Hygiene 62: 112-114.

1561. Levi JE, Tateno AF, Machado AF, Ramalho DC, de Souza VAUF, et al. (2007) Evaluation of a commercial real-time PCR kit for detection of dengue virus in samples collected during an outbreak in Goiania, central Brazil, in 2005. J Clin Microbiol 45: 1893-1897.

1562. Li CF, Lim TW, Han LL, Fang R (1985) Rainfall, abundance of Aedes aegypti and dengue infection in Selangor, Malaysia. Southeast Asian J Trop Med Public Health 16: 560-568.

1563. Li FS, Yang FR, Song JC, Gao H, Tang JQ, et al. (1986) Etiologic and serologic investigations of the 1980 epidemic of dengue fever on Hainan Island, China. Am J Trop Med Hyg 35: 1051-1054.

1564. Li J, Lim SP, Beer D, Patel V, Wen DY, et al. (2005) Functional profiling of recombinant NS3 proteases from all four serotypes of dengue virus using tetrapeptide and octapeptide substrate libraries. J Biol Chem 280: 28766-28774.

1565. Lian CW, Seng CM, Chai WY (2006) Spatial, environmental and entomological risk factors analysis on a rural dengue outbreak in Lundu District in Sarawak, Malaysia. Tropical Biomedicine 23: 85-96.

1566. Libraty DH, Endy TP, Kalayanarooj S, Chansiriwongs W, Nisalak A, et al. (2002) Assessment of body fluid compartment volumes by multifrequency bioelectrical impedance spectroscopy in children with dengue. Trans R Soc Trop Med Hyg 96: 295-299.

1567. Liew KJL, Chow VTK (2004) Differential display RT-PCR analysis of ECV304 endothelial-like cells infected with dengue virus type 2 reveals messenger RNA expression profiles of multiple human genes involved in known and novel roles. J Med Virol 72: 597-609.

1568. Liew KJL, Chow VTK (2006) Microarray and real-time RT-PCR analyses of a novel set of differentially expressed human genes in ECV304 endothelial-like cells infected with dengue virus type 2. J Virol Methods 131: 47-57.

1569. Ligtenberg JJ, Hospers GA, Sprenger HG, Weits J (1991) Hemorragische koorts door dengue bij twee toeristen. Ned Tijdschr Geneeskd 135: 2394-2397.

1570. Likitnukul S, Prapphal N, Pongpunlert W, Kingwatanakul P, Poovorawan Y (2004) Dual infection: dengue hemorrhagic fever with unusual manifestations and mycoplasma pneumonia in a child. Southeast Asian J Trop Med Public Health 35: 399-402.

1571. Likosky WH, Calisher CH, Michelson AL, Correa-Coronas R, Henderson BE, et al. (1973) An epidermiologic study of dengue type 2 in Puerto Rico, 1969. Am J Epidemiol 97: 264-275.

1572. Lim CS, Chua JJE, Wilkerson J, Chow VTK (2006) Differential dengue cross-reactive and neutralizing antibody responses in BALB/c and Swiss albino mice induced by immunization with flaviviral vaccines and by infection with homotypic dengue-2 virus strains. Viral Immunol 19: 33-41.

1573. Lim M, Goh HK (2005) Rhabdomyolysis following dengue virus infection. Singapore Med J 46: 645-646.

1574. Lim WK, Mathur R, Koh A, Yeoh R, Chee SP (2004) Ocular manifestations of dengue fever. Ophthalmology 111: 2057-2064.

1575. Lima EQ, Gorayeb FS, Zanon JR, Nogueira ML, Ramalho HJ, et al. (2007) Dengue haemorrhagic fever-induced acute kidney injury without hypotension, haemolysis or rhabdomyolysis. Nephrology Dialysis Transplantation 22: 3322-3326.

1576. Lima EQ, Nogueira ML (2008) Viral hemorrhagic fever-induced acute kidney injury. Semin Nephrol 28: 409-415.

1577. Lima RS, Scarpassa VM (2009) Evidence of two lineages of the dengue vector Aedes aegypti in the Brazilian Amazon, based on mitochondrial DNA ND4 gene sequences. Genet Mol Biol 32: 414-422.

1578. Lima VL, Figueiredo LT, Correa FH, Leite OF, Rangel O, et al. (1999) Dengue: inquerito sorologico pos-epidemico em zona urbana do Estado de Sao Paulo (Brasil). Rev Saude Publica 33: 566-574.

1579. Limkittikul K, Yingsakmongkon S, Jittmittraphap A, Chuananon S, Kongphrai Y, et al. (2005) Clinical differences among PCR-proven dengue serotype infections. Southeast Asian J Trop Med Public Health 36: 1432-1438.

1580. Limon-Flores AY, Perez-Tapia M, Estrada-Garcia I, Vaughan G, Escobar-Gutierrez A, et al. (2005) Dengue virus inoculation to human skin explants: an effective approach to assess in situ the early infection and the effects on cutaneous dendritic cells. Int J Exp Pathol 86: 323-334.

1581. Limonta D, Capo V, Torres G, Perez AB, Guzman MG (2007) Apoptosis in tissues from fatal dengue shock syndrome. J Clin Virol 40: 50-54.

1582. Limonta D, Torres G, Capo V, Guzman MG (2008) Apoptosis, vascular leakage and increased risk of severe dengue in a type 2 diabetes mellitus patient. Diab Vasc Dis Res 5: 213-214.

1583. Lin CF, Chiu SC, Hsiao YL, Wan SW, Lei HY, et al. (2005) Expression of cytokine, chemokine, and adhesion molecules during endothelial cell activation induced by antibodies against dengue virus nonstructural protein 1. J Immunol 174: 395-403.

1584. Lin CF, Lei HY, Liu CC, Liu HS, Yeh TM, et al. (2001) Generation of IgM anti-platelet autoantibody in dengue patients. J Med Virol 63: 143-149.

1585. Lin CF, Wan SW, Cheng HJ, Lei HY, Lin YS (2006) Autoimmune pathogenesis in dengue virus infection. Viral Immunol 19: 127-132.

1586. Lin HM, Chen CS, Hsu CC, Chung CL (1986) [Dengue vector density survey in Liuchiu, Pintung, Taiwan]. Zhonghua Min Guo Wei Sheng Wu Ji Mian Yi Xue Za Zhi 19: 218-223.

1587. Lin SR, Hsieh SC, Yueh YY, Lin TH, Chao DY, et al. (2004) Study of sequence variation of dengue type 3 virus in naturally infected mosquitoes and human hosts: Implications for transmission and evolution. J Virol 78: 12717-12721.

1588. Lindegren G, Vene S, Lundkvist A, Falk KI (2005) Optimized diagnosis of acute dengue fever in Swedish travelers by a combination of reverse transcription-PCR and immunoglobulin m detection. J Clin Microbiol 43: 2850-2855.

1589. Ling LM, Wilder-Smith A, Leo YS (2007) Fulminant hepatitis in dengue haemorrhagic fever. J Clin Virol 38: 265-268.

1590. Linnen JM, Vinelli E, Sabino EC, Tobler LH, Hyland C, et al. (2008) Dengue viremia in blood donors from Honduras, Brazil, and Australia. Transfusion (Paris) 48: 1355-1362.

1591. Liou LM, Lan SH, Lai CL (2008) Electroencephalography burst suppression in a patient with dengue encephalopathy: A case report. Clin Neurophysiol 119: 2205-2208.

1592. Liou LM, Lan SH, Lai CL (2008) Dengue fever with ischemic stroke: a case report. Neurologist 14: 40-42.

1593. Liu C, Broom AK, Kurucz N, Whelan PI (2005) Communicable Diseases Network Australia: National Arbovirus and Malaria Advisory Committee annual report 2004-05. Commun Dis Intell 29: 341-357.

1594. Liu CC, Huang KJ, Lin YS, Yeh TM, Liu HS, et al. (2002) Transient CD4/CD8 ratio inversion and aberrant immune activation during dengue virus infection. J Med Virol 68: 241-252.

1595. Liu CC, Wu SC (2004) Mosquito and mammalian cells grown on microcarriers for four-serotype dengue virus production: Variations in virus titer, plaque morphology, and replication rate. Biotechnol Bioeng 85: 482-488.

1596. Liu HW, Ho TL, Hwang CS, Liao YH (1989) Clinical observations of virologically confirmed dengue fever in the 1987 outbreak in southern Taiwan. Gaoxiong Yi Xue Ke Xue Za Zhi 5: 42-49.

1597. Liu P, Woda M, Ennis FA, Libraty DH (2009) Dengue Virus Infection Differentially Regulates Endothelial Barrier Function over Time through Type I Interferon Effects. J Infect Dis 200: 191-201.

1598. Liu TC, Chan YC, Han P (1991) Lymphocyte changes in secondary dengue fever: use of the Technicon H*1 to monitor progress of infection. Southeast Asian J Trop Med Public Health 22: 332-336.

1599. Liu WT, Chen CL, Lee SS, Chan CC, Lo FL, et al. (1991) Isolation of dengue virus with a human promonocyte cell line. Am J Trop Med Hyg 44: 494-499.

1600. Lloyd AT, McKenna DP (2008) Fever, nausea, and vomiting in a student from Thailand. Journal of the American Academy of Physician Assistants 21: 32, 34-35.

1601. Lloyd LS, Winch P, Ortega-Canto J, Kendall C (1992) Results of a community-based Aedes aegypti control program in Merida, Yucatan, Mexico. Am J Trop Med Hyg 46: 635-642.

1602. Loa CC, Adelson ME, Mordechai E, Raphaelli I, Tilton RC (2004) Serological diagnosis of human babesiosis by IgG enzyme-linked immunosorbent assay. Curr Microbiol 49: 385-389.

1603. Loh BK, Bacsal K, Chee SP, Cheng BC, Wong D (2008) Foveolitis associated with dengue Fever: a case series. Ophthalmologica 222: 317-320.

1604. Loke H, Bethell D, Phuong CX, Day N, White N, et al. (2002) Susceptibility to dengue hemorrhagic fever in vietnam: evidence of an association with variation in the vitamin d receptor and Fc gamma receptor IIa genes. Am J Trop Med Hyg 67: 102-106.

1605. Loke H, Bethell DB, Phuong CX, Dung M, Schneider J, et al. (2001) Strong HLA class I--restricted T cell responses in dengue hemorrhagic fever: a double-edged sword? J Infect Dis 184: 1369-1373.

1606. Lolekha R, Chokephaibulkit K, Yoksan S, Vanprapar N, Phongsamart W, et al. (2004) Diagnosis of dengue infection using various diagnostic tests in the early stage of illness. Southeast Asian J Trop Med Public Health 35: 391-395.

1607. Long HT, Hibberd ML, Hien TT, Dung NM, Van Ngoc T, et al. (2009) Patterns of Gene Transcript Abundance in the Blood of Children with Severe or Uncomplicated Dengue Highlight Differences in Disease Evolution and Host Response to Dengue Virus Infection. J Infect Dis 199: 537-546.

1608. Lopez C, Sanchez J, Hermida L, Zulueta A, Marquez G (2004) Cysteine mediated multimerization of a recombinant dengue E fragment fused to the P64k protein following immobilized metal ion affinity chromatography. Protein Expr Purif 34: 176-182.

1609. Lopez-Medrano F, Vergara A, Garcia-Donoso C, Menassa A, Aguado JM (2006) MuJer proveniente de Bolivia con fiebre. exantema y signo del torniquetell positivo. A proposito de un caso de dengue. Rev Clin Esp 206: 212-212.

1610. Lopez-Velez R, Perez-Casas C, Vorndam AV, Rigau J (1996) Dengue in Spanish travelers returning from the tropics. Eur J Clin Microbiol Infect Dis 15: 823-826.

1611. Lopez-Velez R, Tapia-Ruano C, Garcia-Camacho A, Sanchez R (1994) Dengue: enfermedad importada del subcontinente indio. Enferm Infecc Microbiol Clin 12: 182-186.

1612. Lorono Pino MA, Farfan Ale JA, Rosado Paredes EP, Kuno G, Gubler DJ (1993) Epidemic dengue 4 in the Yucatan, Mexico, 1984. Rev Inst Med Trop Sao Paulo 35: 449-455.

1613. Lorono-Pino MA, Farfan-Ale JA, Zapata-Peraza AL, Rosado-Paredes EP, Flores-Flores LF, et al. (2004) Introduction of the American/Asian genotype of dengue 2 virus into the Yucatan State of Mexico. Am J Trop Med Hyg 71: 485-492.

1614. Lourenco de Oliveira R, Vazeille M, de Filippis AM, Failloux AB (2003) Large genetic differentiation and low variation in vector competence for dengue and yellow fever viruses of Aedes albopictus from Brazil, the United States, and the Cayman Islands. Am J Trop Med Hyg 69: 105-114.

1615. Lourenco-de-Oliveira R, Castro MG, Braks MA, Lounibos LP (2004) The invasion of urban forest by dengue vectors in Rio de Janeiro. J Vector Ecol 29: 94-100.

1616. Lourenco-de-Oliveira R, Honorio NA, Castro MG, Schatzmayr HG, Miagostovich MP, et al. (2002) Dengue virus type 3 isolation from Aedes aegypti in the municipality of Nova Iguacu, State of Rio de Janeiro. Mem Inst Oswaldo Cruz 97: 799-800.

1617. Lourenco-de-Oliveira R, Vazeille M, de Filippis AM, Failloux AB (2004) Aedes aegypti in Brazil: genetically differentiated populations with high susceptibility to dengue and yellow fever viruses. Trans R Soc Trop Med Hyg 98: 43-54.

1618. Low JGH, Ooi EE, Tolfvenstam T, Leo YS, Hibberd ML, et al. (2006) Early dengue infection and outcome study (EDEN) - Study design and preliminary findings. Ann Acad Med Singap 35: 783-789.

1619. Lozach PY, Burleigh L, Staropoli I, Navarro-Sanchez E, Harriague J, et al. (2005) Dendritic cell-specific intercellular adhesion molecule 3-grabbing non-integrin (DC-SIGN)-mediated enhancement of dengue virus infection is independent of DC-SIGN internalization signals. J Biol Chem 280: 23698-23708.

1620. Lozano-Fuentes S, Fernandez-Salas I, Munoz MD, Garcia-Rejon J, Olson KE, et al. (2009) The Neovolcanic Axis Is a Barrier to Gene Flow among Aedes aegypti Populations in Mexico That Differ in Vector Competence for Dengue 2 Virus. PLoS Negl Trop Dis 3: -.

1621. Lu PL, Hsiao HH, Tsai JJ, Chen TC, Feng MC, et al. (2005) Dengue virus-associated hemophagocytic syndrome and dyserythropoiesis: a case report. Kaohsiung J Med Sci 21: 34-39.

1622. Lucas GN, Amerasinghe A, Sriranganathan S (2000) Dengue haemorrhagic fever in Sri Lanka. Indian J Pediatr 67: 503-504.

1623. Lum LCS, Suaya JA, Tan LH, Sah BK, Shepard DS (2008) Quality of life of dengue patients. Am J Trop Med Hyg 78: 862-867.

1624. Luo H, He J, Zheng K, Li L, Jiang L (2002) [Analysis on the epidemiologic features of Dengue fever in Guangdong province, 1990-2000]. Zhonghua Liu Xing Bing Xue Za Zhi 23: 427-430.

1625. Luz PM, Codeco CT, Massad E, Struchiner CJ (2003) Uncertainties regarding dengue modeling in Rio de Janeiro, Brazil. Mem Inst Oswaldo Cruz 98: 871-878.

1626. Luz PM, Grinsztejn B, Galvani AP (2009) Disability adjusted life years lost to dengue in Brazil. Trop Med Int Health 14: 237-246.

1627. Lye DC, Chan M, Lee VJ, Leo YS (2008) Do young adults with uncomplicated dengue fever need hospitalisation? A retrospective analysis of clinical and laboratory features. Singapore Med J 49: 476-479.

1628. Lye DC, Lee VJ, Sun Y, Leo YS (2009) Lack of Efficacy of Prophylactic Platelet Transfusion for Severe Thrombocytopenia in Adults with Acute Uncomplicated Dengue Infection. Clin Infect Dis 48: 1262-1265.

1629. Lyerla R, Rigau-Perez JG, Vorndam AV, Reiter P, George AM, et al. (2000) A dengue outbreak among camp participants in a Caribbean island, 1995. J Travel Med 7: 59-63.

1630. Ma LX, Jones CT, Groesch TD, Kuhn RJ, Post CB (2004) Solution structure of dengue virus capsid protein reveals another fold. Proc Natl Acad Sci U S A 101: 3414-3419.

1631. Mabalirajan U, Kadhiravan T, Sharma SK, Banga A, Ghosh B (2005) Short report: T-I-(I)2 immune response in patients with dengue during defervescence: Preliminary evidence. Am J Trop Med Hyg 72: 783-785.

1632. Macay AP (2002) DENGUE - ECUADOR (GALAPAGOS ISLANDS). In: ProMED-mail, editor. El Comercio, Tue 13 Aug 2002 (translated by MPP) [edited] ed: PROMED.

1633. Machado JP, de Oliveira RM, Souza-Santos R (2009) Análise espacial da ocorrência de dengue e condições de vida na cidade de Nova Iguaçu, Estado do Rio de Janeiro, Brasil. Cad Saude Publica 25: 1024-1033.

1634. Madeira NG, Macharelli CA, Pedras JF, Delfino MC (2002) Education in primary school as a strategy to control dengue. Rev Soc Bras Med Trop 35: 221-226.

1635. Madhav N, Banks AL (2006) DENGUE/DHF UPDATE 2006 (09). PROMED.

1636. Madhav N, Banks AL (2006) DENGUE/DHF UPDATE 2006 (11). PROMED.

1637. Magpusao NS, Monteclar A, Deen JL (2003) Slow improvement of clinically-diagnosed dengue haemorrhagic fever case fatality rates. Trop Doct 33: 156-159.

1638. Maguire T (1994) Do Ross River and dengue viruses pose a threat to New Zealand? N Z Med J 107: 448-450.

1639. Mahadev PV, Kollali VV, Rawal ML, Pujara PK, Shaikh BH, et al. (1993) Dengue in Gujarat state, India during 1988 & 1989. Indian J Med Res 97: 135-144.

1640. Mahadev PV, Prasad SR, Ilkal MA, Mavale MS, Bedekar SS, et al. (1997) Activity of dengue-2 virus and prevalence of Aedes aegypti in the Chirimiri colliery area, Madhya Pradesh, India. Southeast Asian J Trop Med Public Health 28: 126-137.

1641. Mahilum MM, Ludwig M, Madon MB, Becker N (2005) Evaluation of the present dengue situation and control strategies against Aedes aegypti in Cebu City, Philippines. J Vector Ecol 30: 277-283.

1642. Mahsinah SA (2004) DENGUE/DHF UPDATE 2004 (20) [Malaysia/ Sri Lanka/ Indonesia]. In: ProMED-mail, editor. New Straits Times, 3 Jul 2004 [edited] ed: PROMED.

1643. Mairuhu A, Setiati T, Koraka P, Hack C, Leyte A, et al. (2005) Increased PAI-1 plasma levels and risk of death from dengue: no association with the 4G/5G promoter polymorphism. Thromb J 3: 17.

1644. Mairuhu ATA, Peri G, Setiati TE, Hack CE, Koraka P, et al. (2005) Elevated plasma levels of the long pentraxin, pentraxin 3, in severe dengue virus infections. J Med Virol 76: 547-552.

1645. Malavige GN, Ranatunga PK, Jayaratne SD, Wijesiriwardana B, Seneviratne SL, et al. (2007) Dengue viral infections as a cause of encephalopathy. Indian J Med Microbiol 25: 143-145.

1646. Malavige GN, Ranatunga PK, Velathanthiri VG, Fernando S, Karunatilaka DH, et al. (2006) Patterns of disease in Sri Lankan Paediatric dengue patients. Arch Dis Child.

1647. Malavige GN, Velathanthiri VG, Wijewickrama ES, Fernando S, Jayaratne SD, et al. (2006) Patterns of disease among adults hospitalized with dengue infections. Q J Med.

1648. Malcolm RL, Hanna JN, Phillips DA (1999) The timeliness of notification of clinically suspected cases of dengue imported into north Queensland. Aust N Z J Public Health 23: 414-417.

1649. Mallet EC, Gestas P, Pilorget H, Bataille H (1993) La dengue hemorragique avec choc chez l'enfant en Polynesie francaise. Bull Soc Pathol Exot Filiales 86: 450-454.

1650. Manaloto CR, Hayes CG (1989) Isolation of dengue viruses from hospitalized patients in the Philippines, 1983-1986. Southeast Asian J Trop Med Public Health 20: 541-547.

1651. Mangada MM, Endy TP, Nisalak A, Chunsuttiwat S, Vaughn DW, et al. (2002) Dengue-specific T cell responses in peripheral blood mononuclear cells obtained prior to secondary dengue virus infections in Thai schoolchildren. J Infect Dis 185: 1697-1703.

1652. Mangada MM, Ennis FA, Rothman AL (2004) Quantitation of dengue virus specific CD4+T cells by intracellular cytokine staining. J Immunol Methods 284: 89-97.

1653. Mangada MN, Igarashi A (1998) Molecular and in vitro analysis of eight dengue type 2 viruses isolated from patients exhibiting different disease severities. Virology 244: 458-466.

1654. Mangada MN, Igarashi A (1997) Sequences of terminal non-coding regions from four dengue-2 viruses isolated from patients exhibiting different disease severities. Virus Genes 14: 5-12.

1655. Mangara SG, Sukmono, Kusumadiharja J, Suroso T, Sutjipto H (2000) The risk of dengue hemorrhagic fever (DHF) outbreak based on vector density in Kurau, Riau province, Indonesia. Southeast Asian J Trop Med Public Health 31 Suppl 1: 134-139.

1656. Manock SR, Jacobsen KH, de Bravo NB, Russell KL, Negrete M, et al. (2009) Etiology of Acute Undifferentiated Febrile Illness in the Amazon Basin of Ecuador. Am J Trop Med Hyg 81: 146-151.

1657. Mansuy JM, Delor R, Mehdaoui H, Elizabeth L (1996) Premier cas de dengue hemorragique avec syndrome de choc observe en Martinique. Bull Soc Pathol Exot Filiales 89: 243-244.

1658. Mantke OD, Lemmer K, Biel SS, Groen J, Schmitz H, et al. (2004) Quality control assessment for the serological diagnosis of dengue virus infections. J Clin Virol 29: 105-112.

1659. Marchette NJ, Sung Chow JS, Halstead SB, Lolekha S, Pongpanich B (1975) Dengue virus replication in cultures of peripheral blood leukocytes during the course of dengue haemorrhagic fever. Southeast Asian J Trop Med Public Health 6: 316-321.

1660. Marchiori E, Ferreira JLN, Bittencourt CN, Neto CAD, Zanetti G, et al. (2009) Pulmonary hemorrhage syndrome associated with dengue fever, High-resolution computed tomography findings: a case report. Orphanet J Rare Dis 4: -.

1661. Markon C (1998) DENGUE/DHF - INDONESIA (JAKARTA). The Straits Times, Malaysia ed: PROMED.

1662. Markon C (1998) DENGUE/DHF - INDONESIA (03). PROMED.

1663. Markon C (1998) DENGUE/DHF - INDONESIA (04). CNN Internet News - 20 April 98 ed: PROMED.

1664. Markon C (1998) DENGUE - PHILIPPINES. Sun.Star Bacolod Daily, Philippines ed. Bacolod, Philippines: PROMED.

1665. Markon C (1998) DENGUE - PHILIPPINES (03). Philippine Daily Inquirer <http://www.healthnet.org/programs/promed.html> ed: PROMED.

1666. Markon C (1998) DENGUE - MALAYSIA (SARAWAK) (02). The Sarawak Tribune Online - 4 August ed: PROMED.

1667. Markon C (1998) DENGUE - PHILIPPINES (04). Philippine Inquirer, 3 August ed: PROMED.

1668. Markon C (1998) DENGUE - MALAYSIA (PETALING JAYA). The Star Online - Malaysia August 11 1998 ed: PROMED.

1669. Markon C (1998) DENGUE/DHF - THAILAND (PHUKET). Phuket Gazet - August 13 Internet Edition. ed: PROMED.

1670. Markon C (1998) DENGUE/DHF - CAMBODIA (02). Xinhua, 21 AUG 1998 ed. Phnom Penh: PROMED.

1671. Markon C (1998) DENGUE/DHF - VIETNAM (07). 21 August CNN Custom News - Edited ed: PROMED.

1672. Markon C (1998) DENGUE - VIETNAM (04). In: ProMED-mail, editor. Vietnam News - Internet Edition 22 July ed: PROMED.

1673. Markon C (1998) DENGUE - PHILIPPINES (02). In: ProMED-mail, editor. Philippine Daily Inquirer Internet Edition, July 15 ed: PROMED.

1674. Markon C (1998) DENGUE - COSTA RICA (GOLFITO) (02). In: ProMED-mail, editor. CNN Custom News - July 15 (Edited) ed: PROMED.

1675. Markon C (1998) DENGUE/DHF - THAILAND. In: ProMED-mail, editor. The Phuket Gazette (Internet Edition) 13 Jul 1998 ed: PROMED.

1676. Markon C (1998) DENGUE/DHF - MALAYSIA (SARAWAK). In: ProMED-mail, editor. Sarawak Tribune Online 9 Jul 1998 ed: PROMED.

1677. Markon C (1998) DENGUE - VIET NAM. In: ProMED-mail, editor. Vietnam News (Internet) - July 8 ed: PROMED.

1678. Markon C (1998) DENGUE - SINGAPORE. In: ProMED-mail, editor. The Straits Times (Singapore): JULY 3 1998 ed: PROMED.

1679. Markon C (1998) DENGUE - MALAYSIA (SARAWAK). Sarawak Tribune Online ed: PROMED.

1680. Markon C (1998) DENGUE/DHF - INDONESIA (07). In: ProMED-mail, editor. CNN Interactive - 4 May (Edited) ed: PROMED.

1681. Markon C (1998) DENGUE/DHF - PHILIPPINES (04). In: ProMED-mail, editor. Xinhua via CNN Internet News, 23 September 1998 ed: PROMED.

1682. Markon C (1998) DENGUE/DHF - PHILIPPINES (03). In: ProMED-mail, editor. Philippine Daily Inquirer 17 September 1998 ed: PROMED.

1683. Markon C (1998) DENGUE/DHF - LAOS. In: ProMED-mail, editor. Vientiane Times September 1 - 3 ed: PROMED.

1684. Markon C (1998) DENGUE/DHF - PHILIPPINES (02). In: ProMED-mail, editor. Media reports (CNN, Reuters) ed: PROMED.

1685. Markon C (1998) DENGUE - PHILIPPINES (05). In: ProMED-mail, editor. Philippine Daily Inquirer 4 September ed: PROMED.

1686. Markon C (1998) DENGUE - INDIA (MAHARASHTRA) (03). In: ProMED-mail, editor. Deccan Chronicle 3 September ed: PROMED.

1687. Markon C (1999) DENGUE/DHF - INDONESIA (JAKARTA). In: ProMED-mail, editor. Xinhua, 23 Feb 1999 ed: PROMED.

1688. Markon C (1999) DENGUE - INDIA (MUMBAI). In: ProMED-mail, editor. Times of India 11 Oct 1999 [edited] ed: PROMED.

1689. Markon C (2000) DENGUE/DHF - PARAGUAY. In: ProMED-mail, editor. Reuters 9 March 2000 ed: PROMED.

1690. Marks EN, Kay BH, Elson-Harris MM (1980) Potential vectors of malaria and dengue at Townsville, Queensland. Med J Aust 2: 676-677.

1691. Maroun SLC, Marliere RCC, Barcellus RC, Barbosa CN, Ramos JRM, et al. (2008) Case report: vertical dengue infection. J Pediatr (Rio J) 84: 556-559.

1692. Marques CA, Forattini OP, Massad E (1994) The basic reproduction number for dengue fever in Sao Paulo state, Brazil: 1990-1991 epidemic. Trans R Soc Trop Med Hyg 88: 58-59.

1693. Marques CC, Marques GR, de Brito M, dos Santos Neto LG, Ishibashi Vde C, et al. (1993) Estudo comparativo de eficacia de larvitrampas e ovitrampas para vigilancia de vetores de dengue e febre amarela. Rev Saude Publica 27: 237-241.

1694. Marquetti Mdel C, Suarez S, Bisset J, Leyva M (2005) Reporte de habitats utilizados por Aedes aegypti en Ciudad de la Habana, Cuba. Rev Cuba Med Trop 57: 159-161.

1695. Marquez C (2004) DENGUE/DHF UPDATE 2004 (22) [Philippines]. In: ProMED-mail, editor. ABS - CBN 21 Jul 2004 [edited] ed: PROMED.

1696. Marshall M (2005) DENGUE/DHF UPDATE 2005 (30). PROMED.

1697. Marshall M, Banks A-L, Barrett B (2007) Dengue/DHF update 2007 (11) PROMED: Promed.

1698. Martin J, Hermida L, Castro J, Lazo L, Martinez R, et al. (2009) Viremia and antibody response in green monkeys (Chlorocebus aethiops sabaeus) infected with dengue virus type 2: A potential model for vaccine testing. Microbiol Immunol 53: 216-223.

1699. Martin NC, Pardo J, Simmons M, Tjaden JA, Widjaja S, et al. (2006) An immunocytometric assay based on dengue infection via DC-SIGN permits rapid measurement of anti-dengue neutralizing antibodies. J Virol Methods.

1700. Martinet V, Reynders M, Theunissen C (2009) Fievre au retour des Tropiques. Rev Med Brux 30: 192-194.

1701. Martinez RA, Diaz FA, Villar LA (2005) Evaluación de la definición clínica de dengue sugerida por la Organización Mundial de la Salud. Biomedica 25: 412-416.

1702. Martinez-Vega RA, Diaz-Quijano FA, Villar-Centeno LA (2006) Dificultad para el diagnóstico clínico temprano del dengue en un área endémica y su impacto sobre el manejo médico inicial. Rev Med Chil 134: 1153-1160.

1703. Marzochi KB (1994) Dengue in Brazil--situation, transmission and control--a proposal for ecological control. Mem Inst Oswaldo Cruz 89: 235-245.

1704. Massad E, Burattini MN, Coutinho FA, Lopez LF (2003) Dengue and the risk of urban yellow fever reintroduction in Sao Paulo State, Brazil. Rev Saude Publica 37: 477-484.

1705. Massad E, Coutinho FA, Burattini MN, Lopez LF (2001) The risk of yellow fever in a dengue-infested area. Trans R Soc Trop Med Hyg 95: 370-374.

1706. Massad E, Coutinho FA, Ma S, Burattini MN (2009) A hypothesis for the 2007 dengue outbreak in Singapore. Epidemiol Infect: 1-7.

1707. Massad E, Wilder-Smith A (2009) Risk Estimates of Dengue in Travelers to Dengue Endemic Areas Using Mathematical Models. J Travel Med 16: 191-193.

1708. Masuh H, De Licastro SA, Lopez PA, Vega C, Zerba E (2003) Field evaluation of a smoke-generating formulation containing beta-cypermethrin against the dengue vector in Argentina. J Am Mosq Control Assoc 19: 53-57.

1709. Mathenge EGM, Parquet MDC, Funakoshi Y, Houhara S, Wong PF, et al. (2004) Fusion PCR generated Japanese encephalitis virus/dengue 4 virus chimera exhibits lack of neuroinvasiveness, attenuated neurovirulence, and a dual-flavi immune response in mice. J Gen Virol 85: 2503-2513.

1710. Matheus S, Deparis X, Labeau B, Lelarge J, Morvan J, et al. (2005) Use of four dengue virus antigens for determination of dengue immune status by enzyme-linked Immunosorbent assay of immunoglobulin G avidity. J Clin Microbiol 43: 5784-5786.

1711. Matheus S, Deparis X, Labeau B, Lelarge J, Morvan J, et al. (2005) Discrimination between primary and secondary Dengue virus infection by an immunoglobulin G avidity test using a single acute-phase serum sample. J Clin Microbiol 43: 2793-2797.

1712. Matheus S, Meynard JB, Lacoste V, Morvan J, Deparis X (2007) Use of capillary blood samples as a new approach for diagnosis of dengue virus infection. J Clin Microbiol 45: 887-890.

1713. Matheus S, Meynard JB, Lavergne A, Girod R, Moua D, et al. (2008) Short Report: Dengue-3 Outbreak in Paraguay: Investigations Using Capillary Blood Samples on Filter Paper. Am J Trop Med Hyg 79: 685-687.

1714. Mathew A, Kurane I, Green S, Stephens HA, Vaughn DW, et al. (1998) Predominance of HLA-restricted cytotoxic T-lymphocyte responses to serotype-cross-reactive epitopes on nonstructural proteins following natural secondary dengue virus infection. J Virol 72: 3999-4004.

1715. Matlani M, Chakravarti A, Rawal A, Kashyap B, Gurtoo A (2009) Dengue encephalitis: an entity now common in dengue-prone regions. Trop Doct 39: 115-116.

1716. Matsuda T, Almasan A, Tomita M, Tamaki K, Saito M, et al. (2005) Dengue virus-induced apoptosis in hepatic cells is partly mediated by Apo2 ligand/tumour necrosis factor-related apoptosis-inducing ligand. J Gen Virol 86: 1055-1065.

1717. Mazariegos M, Klassen P, Solomons NW, Furst P (2000) Bioelectrical impedance spectroscopy in health and disease. Correspondence between whole body and segmental bioelectrical impedance spectroscopy indices in patients with classical dengue fever. Ann N Y Acad Sci 904: 205-209.

1718. McAvin JC, Escamilla EM, Blow JA, Turell MJ, Quintana M, et al. (2005) Rapid identification of dengue virus by reverse transcription-polymerase chain reaction using field-deployable instrumentation. Mil Med 170: 1053-1059.

1719. McBride J (1998) DENGUE - AUSTRALIA (TORRES STRAIT). Cairns (Australia): PROMED.

1720. McBride JH (1999) Dengue fever. An Australian perspective. Aust Fam Physician 28: 319-323.

1721. McBride WJ (2009) Evaluation of dengue NS1 test kits for the diagnosis of dengue fever. Diagn Microbiol Infect Dis 64: 31-36.

1722. McBride WJ, Bielefeldt-Ohmann H (2000) Dengue viral infections; pathogenesis and epidemiology. Microbes Infect 2: 1041-1050.

1723. McBride WJ, Mullner H, LaBrooy JT, Wronski I (1998) The 1993 dengue 2 epidemic in Charters Towers, North Queensland: clinical features and public health impact. Epidemiol Infect 121: 151-156.

1724. McBride WJ, Mullner H, LaBrooy JT, Wronski I (1998) The 1993 dengue 2 epidemic in North Queensland: a serosurvey and comparison of hemagglutination inhibition with an ELISA. Am J Trop Med Hyg 59: 457-461.

1725. McBride WJ, Mullner H, Muller R, Labrooy J, Wronski I (1998) Determinants of dengue 2 infection among residents of Charters Towers, Queensland, Australia. Am J Epidemiol 148: 1111-1116.

1726. McBride WJH (2005) Deaths associated with dengue haemorrhagic fever: the first in Australia in over a century. Med J Aust 183: 35-37.

1727. McCarthy MA, Carpenter D, Goyette M, Nguyen DT (1995) Dengue fever in Canada. Can Commun Dis Rep 21: 185-187.

1728. McConnell KJ, Gubler DJ (2003) Guidelines on the cost-effectiveness of larval control programs to reduce dengue transmission in Puerto Rico. Rev Panam Salud Publica 14: 9-16.

1729. McCredie J (2009) Dengue fever epidemic hits northern Australia. Br Med J 338: -.

1730. Medin CL, Fitzgerald KA, Rothman AL (2005) Dengue virus nonstructural protein NS5 induces interleukin-8 transcription and secretion. J Virol 79: 11053-11061.

1731. Mehendale SM, Risbud AR, Rao JA, Banerjee K (1991) Outbreak of dengue fever in rural areas of Parbhani district of Maharashtra (India). Indian J Med Res 93: 6-11.

1732. Mehta S (2004) DENGUE/DHF UPDATE 2004 (34). PROMED.

1733. Mein J, O'Grady KA, Whelan P, Merianos A (1998) Dengue or Kokobera? A case report from the top end of the Northern Territory. Commun Dis Intell 22: 105-107.

1734. Mekmullica J, Pancharoen C, Deerojanawong J, Lertsapcharoen P, Thisyakorn U, et al. (2005) Concomitant dengue infection and Kawasaki disease in an infant: a case report and literature review. J Med Assoc Thai 88: 436-439.

1735. Mekmullica J, Suwanphatra A, Thienpaitoon H, Chansongsakul T, Cherdkiatkul T, et al. (2005) Serum and urine sodium levels in dengue patients. Southeast Asian J Trop Med Public Health 36: 197-199.

1736. Melissant CF, Kauffmann RH (1992) Infection with Dengue virus. Neth J Med 41: 272-274.

1737. Melo PR, Reis EA, Ciuffo IA, Goes M, Blanton RE, et al. (2007) The dynamics of dengue virus serotype 3 introduction and dispersion in the state of Bahia, Brazil. Mem Inst Oswaldo Cruz 102: 905-912.

1738. Meltzer MI, Rigau-Perez JG, Clark GG, Reiter P, Gubler DJ (1998) Using disability-adjusted life years to assess the economic impact of dengue in Puerto Rico: 1984-1994. Am J Trop Med Hyg 59: 265-271.

1739. Men R, Yamashiro T, Goncalvez AP, Wernly C, Schofield DJ, et al. (2004) Identification of chimpanzee Fab fragments by repertoire cloning and production of a full-length humanized immunoglobulin G1 antibody that is highly efficient for neutralization of dengue type 4 virus. J Virol 78: 4665-4674.

1740. Mendes TS, de Almeida Sobrinho EF, Rosa AA, Dos Anjos LM, da Costa GM, et al. (2009) Dengue maculopathy: visual electrophysiology and optical coherence tomography. Doc Ophthalmol 119: 145-155.

1741. Mendes WDS, Branco MDFC, Medeiros MNL (2006) Clinical case report: Dengue hemorrhagic fever in a patient with acquired immunodeficiency syndrome. Am J Trop Med Hyg 74: 905-907.

1742. Mendes-Ribeiro AC, Moss MB, Siqueira MAS, Moraes TL, Ellory JC, et al. (2008) Dengue fever activates the l-arginine-nitric oxide pathway: An explanation for reduced aggregation of human platelets. Clin Exp Pharmacol Physiol 35: 1143-1146.

1743. Mendez A, Gonzalez G (2003) Dengue hemorragico en ninos: diez anos de experiencia clinica. Biomedica 23: 180-193.

1744. Mendez A, Gonzalez G (2006) Manifestaciones clinicas inusuales del dengue hemorragico en ninos. Biomedica 26: 61-70.

1745. Mendez F, Barreto M, Arias JF, Rengifo G, Munoz J, et al. (2006) Human and mosquito infections by dengue viruses during and after epidemics in a dengue-endemic region of Colombia. Am J Trop Med Hyg 74: 678-683.

1746. Mercado R, Fernandez I, Lozano S (2002) Spatial analysis of dengue cases in Guadalupe, Nuevo Leon, Mexico 1995-96. Southwestern Entomologist 27: 85-90.

1747. Messer WB, Gubler DJ, Harris E, Sivananthan K, de Silva AM (2003) Emergence and global spread of a dengue serotype 3, subtype III virus. Emerg Infect Dis 9: 800-809.

1748. Messer WB, Vitarana UT, Sivananthan K, Elvtigala J, Preethimala LD, et al. (2002) Epidemiology of dengue in Sri Lanka before and after the emergence of epidemic dengue hemorrhagic fever. Am J Trop Med Hyg 66: 765-773.

1749. Meyer C (2001) DENGUE - USA (HAWAII) (02). PROMED.

1750. Meynard JB, Ardillon V, Venturin C, Ravachol F, Basurko C, et al. (2009) First description of a dengue fever outbreak in the interior of French Guiana, February 2006. Eur J Public Health 19: 183-188.

1751. Meynard JB, Chaudet H, Texier G, Ardillon V, Ravachol F, et al. (2008) Value of syndromic surveillance within the Armed Forces for early warning during a dengue fever outbreak in French Guiana in 2006. BMC Med Inform Decis Mak 8: -.

1752. Meynard JB, Summers RH, Faulde M, Texier G, Deparis X, et al. (2006) Epidemie de dengue en Afghanistan: une fausse alerte. Med Trop (Mars) 66: 98-99.

1753. Mi Mi K, khin Aye T (1976) Isolation of dengue type 3 from mosquitoes in Rangoon. Southeast Asian J Trop Med Public Health 7: 507-512.

1754. Miagostovich MP, dos Santos FB, de Araujo ES, Dias J, Schatzmayr HG, et al. (1997) Diagnosis of dengue by using reverse transcriptase-polymerase chain reaction. Mem Inst Oswaldo Cruz 92: 595-599.

1755. Miagostovich MP, dos Santos FB, de Simone TS, Costa EV, Filippis AM, et al. (2002) Genetic characterization of dengue virus type 3 isolates in the State of Rio de Janeiro, 2001. Braz J Med Biol Res 35: 869-872.

1756. Miagostovich MP, dos Santos FB, Fumian TM, Guimaraes FR, da Costa EV, et al. (2006) Complete genetic characterization of a Brazilian dengue virus type 3 strain isolated from a fatal outcome. Mem Inst Oswaldo Cruz 101: 307-313.

1757. Miagostovich MP, Nogueira RM, Cavalcanti SM, Marzochi KB, Schatzmayr HG (1993) Dengue epidemic in the state of Rio de Janeiro, Brazil: virological and epidemiological aspects. Rev Inst Med Trop Sao Paulo 35: 149-154.

1758. Miagostovich MP, Sequeira PC, Dos Santos FB, Maia A, Nogueira RM, et al. (2003) Molecular typing of dengue virus type 2 in Brazil. Rev Inst Med Trop Sao Paulo 45: 17-21.

1759. Miguez-Burbano MJ, Jaramillo CA, Palmer CJ, Shor-Posner G, Velasquez LS, et al. (1999) Total immunoglobulin E levels and dengue infection on San Andres Island, Colombia. Clin Diagn Lab Immunol 6: 624-626.

1760. Milagres M (2003) DENGUE/DHF UPDATE 2003 (31) [Brazil]. In: ProMED-mail, editor. Informativo noolhar, Sat 26 Jul 2003 (translated by Maria Jacobs,

ProMED-ESP) [edited] ed: PROMED.

1761. Milagres M (2003) DENGUE/DHF UPDATE 2003 (49) [Brazil/ Bangladesh/ West Java/ Philippines/ Venezuela]. In: ProMED-mail, editor. Portal de MG 11 Dec 2003 [in Portuguese, summarized by Mod.JW; edited] ed: PROMED.

1762. Milagres M (2003) DENGUE/DHF UPDATE 2003 (04) [Brazil]. In: ProMED-mail, editor: PROMED.

1763. Milagres M (2003) DENGUE/DHF UPDATE 2003 (09) [Paraguay/ Argentina/ Bolivia/ Ecuador/ Saudi Arabia/ Indonesia]. In: ProMED-mail, editor. EFE Agency, Ultimo Segundo, Fri 21 Feb 2003 [in Portuguese,

translated and summarized by Mod.JW] ed: PROMED.

1764. Milagres M (2003) DENGUE/DHF UPDATE 2003 (38) [Colombia/ Panama]. In: ProMED-mail, editor. El Tiempo - Oriente, Colombia, 24 Sep 2003 [in Spanish, summarized

by Maria Jacobs] [edited]

La Prensa 27 Sep 2003 [in Spanish, summarized by Mod.JW] [edited] ed: PROMED.

1765. Milagres M (2004) DENGUE/DHF UPDATE 2004 (02) [Brazil]. In: ProMED-mail, editor. Secretaria de Vigilancia em Saude, Ministry of Health, Brazil Bulletin for week 51 [ending 20 Dec] 2003 [in Portuguese, summarized by Mod.JW; edited] ed: PROMED.

1766. Miller BR, Ballinger ME (1988) Aedes albopictus mosquitoes introduced into Brazil: vector competence for yellow fever and dengue viruses. Trans R Soc Trop Med Hyg 82: 476-477.

1767. Mills GD, Jones PD (1991) Clinical spectrum of dengue fever in travellers. N Z Med J 104: 228-230.

1768. Min M, U T, Aye M, Shwe TN, Swe T (1975) Hydrocortisone in the management of dengue shock syndrome. Southeast Asian J Trop Med Public Health 6: 573-579.

1769. Miranda de Sousa A, Puccioni-Sohler M, Dias Borges A, Fernandes Adorno L, Papais Alvarenga M, et al. (2006) Post-dengue neuromyelitis optica: case report of a Japanese-descendent Brazilian child. J Infect Chemother 12: 396-398.

1770. Miranda LE, Miranda SJ, Rolland M (2003) Case report: spontaneous rupture of the spleen due to dengue fever. Braz J Infect Dis 7: 423-425.

1771. Misra UK, Kalita J, Syam UK, Dhole TN (2006) Neurological manifestations of dengue virus infection. J Neurol Sci.

1772. Mitchell CJ (1998) DENGUE VIRUSES, TRANSMISSION BY _AEDES ALBOPICTUS_ (09) [Taipei]. In: Diseases DoV-BI, Control CfD, editors. Ft. Collins (Colorado): PROMED.

1773. Mitchell CJ, Miller BR (1990) Vertical transmission of dengue viruses by strains of Aedes albopictus recently introduced into Brazil. J Am Mosq Control Assoc 6: 251-253.

1774. Mohammed H, Linnen JM, Munoz-Jordan JL, Tomashek K, Foster G, et al. (2008) Dengue virus in blood donations, Puerto Rico, 2005. Transfusion (Paris) 48: 1348-1354.

1775. Mohan B, Patwari AK, Anand VK (2000) Hepatic dysfunction in childhood dengue infection. J Trop Pediatr 46: 40-43.

1776. Momen H (1998) DENGUE - BRAZIL (03). In: Cruz IO, editor. Rio de Janeiro: PROMED.

1777. Moncayo AC, Fernandez Z, Ortiz D, Diallo F, Sall A, et al. (2004) Dengue emergence and adaptation to peridomestic mosquitoes. Emerg Infect Dis 10: 1790-1796.

1778. Mondini A, Bronzoni RV, Cardeal IL, dos Santos TM, Lazaro E, et al. (2007) Simultaneous infection by DENV-3 and SLEV in Brazil. J Clin Virol 40: 84-86.

1779. Mondini A, Bronzoni RVD, Nunes SHP, Neto FC, Massad E, et al. (2009) Spatio-Temporal Tracking and Phylodynamics of an Urban Dengue 3 Outbreak in Sao Paulo, Brazil. PLoS Negl Trop Dis 3: -.

1780. Mondini A, Neto FC, Sanches MGY, Lopes JCC (2005) Spatial analysis of dengue transmission in a medium-sized city in Brazil. Rev Saude Publica 39: 444-451.

1781. Mongkolsapaya J, Dejnirattisai W, Xu XN, Vasanawathana S, Tangthawornchaikul N, et al. (2003) Original antigenic sin and apoptosis in the pathogenesis of dengue hemorrhagic fever. Nat Med 9: 921-927.

1782. Mongkolsapaya J, Duangchinda T, Dejnirattisai W, Vasanawathana S, Avirutnan P, et al. (2006) T cell responses in dengue hemorrhagic fever: are cross-reactive T cells suboptimal? J Immunol 176: 3821-3829.

1783. Monnin M, M'bou F (2005) An epidemic of dengue fever in a department of paediatrics: report on 58 cases in Lamentin (Martinique). Arch Pediatr 12: 144-150.

1784. Montenegro D, Lacerda HR, Lira TM, de Oliveira DSC, de Lima AAF, et al. (2006) Aspectos clínicos e epidemiológicos da epidemia de dengue no Recife, PE, em 2002. Rev Soc Bras Med Trop 39: 9-13.

1785. Moorthy M, Chandy S, Selvaraj K, Abraham AM (2009) Evaluation of a rapid immunochromatographic device for the detection of IgM IgG antibodies to Dengue viruses (DENV) in a tertiary care hospital in South India. Indian J Med Microbiol 27: 254-256.

1786. Morais SM, Cavalcanti ESB, Bertini LM, Oliveira CLL, Rodrigues JRB, et al. (2006) Larvicidal activity of essential oils from Brazilian Croton species against Aedes aegypti L. J Am Mosq Control Assoc 22: 161-164.

1787. Moreno-Altamirano MMB, Romano M, Legorreta-Herrera M, Sanchez-Garcia FJ (2004) Gene expression in human macrophages infected with dengue virus serotype-2. Scand J Immunol 60: 631-638.

1788. Morens DM (2009) Dengue Fever and Dengue Hemorrhagic Fever. Pediatr Infect Dis J 28: 635-636.

1789. Morens DM, Fauci AS (2008) Dengue and hemorrhagic fever - A potential threat to public health in the United States. the Journal of the American Medical Association JAMA 299: 214-216.

1790. Morens DM, Sather GE, Gubler DJ, Rammohan M, Woodall JP (1987) Dengue shock syndrome in an American traveler with primary dengue 3 infection. Am J Trop Med Hyg 36: 424-426.

1791. Morens DM, Woodall JP, Lopez-Correa RH (1978) Dengue in American children of the Caribbean. J Pediatr 93: 1049-1051.

1792. Morita Y, Kogure H, Sandoh M, Kawashima G, Sato Y, et al. (2008) An imported dengue fever case by dengue virus 3 (DENV-3) infection in Gunma, Japan. Jpn J Infect Dis 61: 90-92.

1793. Moros ZC, Abad MJ, Arsenak M, Martinez D, Cierco MM, et al. (2003) [Molecular and serological diagnosis of a dengue outbreak in Coro, Falcon state, Venezuela]. Invest Clin 44: 219-226.

1794. Morrison AC, Getis A, Santiago M, Rigau-Perez JG, Reiter P (1998) Exploratory space-time analysis of reported dengue cases during an outbreak in Florida, Puerto Rico, 1991-1992. Am J Trop Med Hyg 58: 287-298.

1795. Mosquera JA, Hernandez JP, Valero N, Espina LM, Anez GJ (2005) Ultrastructural studies on dengue virus type 2 infection of cultured human monocytes. Virol J 2: 26.

1796. Moura A (1998) DENGUE - BRAZIL (BELO HORIZONTE). Belo Horizonte: PROMED.

1797. Moura A (1998) DENGUE - BRAZIL (MINAS GERAIS). Belo Horizonte: PROMED.

1798. Moura A (1998) DENGUE - BRAZIL (MINAS GERAIS) (02). Belo Horizonte: PROMED.

1799. Mourao MP, Lacerda MV, Bastos Mde S, Albuquerque BC, Alecrim WD (2004) Dengue hemorrhagic fever and acute hepatitis: a case report. Braz J Infect Dis 8: 461-464.

1800. Mourao MP, Lacerda MV, Macedo VO, Santos JB (2007) Thrombocytopenia in patients with dengue virus infection in the Brazilian Amazon. Platelets 18: 605-612.

1801. Mourya DT, Gokhale MD, Mishra AC (1994) Biochemical basis of DDT-resistance in Aedes aegypti population from a dengue affected area in Shahjahanpur city. Indian J Med Res 99: 212-215.

1802. Mourya DT, Yadav P (2006) Vector biology of dengue & chikungunya viruses. Indian J Med Res 124: 475-480.

1803. Mousson L, Vazeille M, Chawprom S, Prajakwong S, Rodhain F, et al. (2002) Genetic structure of Aedes aegypti populations in Chiang Mai (Thailand) and relation with dengue transmission. Trop Med Int Health 7: 865-872.

1804. Moxon C, Wills B (2008) Management of severe dengue in children. Hot Topics in Infection and Immunity in Children Iv 609: 131-144.

1805. Mudur G (2006) Failure to control mosquitoes has led to two fever epidemics in India. BMJ 333: 773.

1806. Mukherjee KK, Chakravarti SK, Dey PN, Dey S, Chakraborty MS (1987) Outbreak of febrile illness due to dengue virus type 3 in Calcutta during 1983. Trans R Soc Trop Med Hyg 81: 1008-1010.

1807. Munoz J, Puente S, Lopez-Velez R, Domingo C, Ruiz J, et al. (2008) Estudio clinicoepidemiologico del dengue importado en Espana. Med Clin (Barc) 131: 18-21.

1808. Munoz-Jordan JL, Collins CS, Vergne E, Santiago GA, Petersen L, et al. (2009) Highly Sensitive Detection of Dengue Virus Nucleic Acid in Samples from Clinically Ill Patients. J Clin Microbiol 47: 927-931.

1809. Murgue B, Cassar O, Deparis X (2001) Plasma concentrations of sVCAM-1 and severity of dengue infections. J Med Virol 65: 97-104.

1810. Murgue B, Deparis X, Chungue E, Cassar O, Roche C (1999) Dengue: an evaluation of dengue severity in French Polynesia based on an analysis of 403 laboratory-confirmed cases. Trop Med Int Health 4: 765-773.

1811. Murgue B, Roche C, Chungue E, Deparis X (2000) Prospective study of the duration and magnitude of viraemia in children hospitalised during the 1996-1997 dengue-2 outbreak in French Polynesia. J Med Virol 60: 432-438.

1812. Murillo-Llanes J, Soto-Valenzuela H, Flores-Flores P, Peraza-Garay F (2007) Caracterizacion clinica y epidemiologica del dengue. Rev Med Inst Mex Seguro Soc 45: 485-491.

1813. Murray-Smith S, Weinstein P, Skelly C (1996) Field epidemiology of an outbreak of dengue fever in Charters Towers, Queensland: are insect screens protective? Aust N Z J Public Health 20: 545-547.

1814. Muzaffar J, Venkata Krishnan P, Gupta N, Kar P (2006) Dengue encephalitis: why we need to identify this entity in a dengue-prone region. Singapore Med J 47: 975-977.

1815. Myat Thu H, Lowry K, Jiang L, Hlaing T, Holmes EC, et al. (2005) Lineage extinction and replacement in dengue type 1 virus populations are due to stochastic events rather than to natural selection. Virology 336: 163-172.

1816. Myint KS, Endy TP, Mongkolsirichaikul D, Manomuth C, Kalayanarooj S, et al. (2006) Cellular immune activation in children with acute dengue virus infections is modulated by apoptosis. J Infect Dis 194: 600-607.

1817. Myo K, Soe T, Thein Thein M, Than Nu S, Tin Tin S, et al. (1995) Serum cortisol levels in children with dengue haemorrhagic fever. J Trop Pediatr 41: 295-297.

1818. Nagao Y, Koelle K (2008) Decreases in dengue transmission may act to increase the incidence of dengue hemorrhagic fever. Proc Natl Acad Sci U S A 105: 2238-2243.

1819. Nah G, Tan M, Teoh S, Chong CH (2007) Maculopathy associated with dengue fever in a military pilot. Aviat Space Environ Med 78: 1064-1067.

1820. Nainiwal S, Garg P, Prakash G, Nainiwal N (2006) Bilateral vitreous hemorrhage associated with dengue fever. Eye 20: 1404-1405.

1821. Nakhapakorn K, Tripathi NK (2005) An information value based analysis of physical and climatic factors affecting dengue fever and dengue haemorrhagic fever incidence. Int J Health Geogr 4: 13.

1822. Nalim S, Gubler DJ, Basuno E, Suwasono H, Masran M, et al. (1978) Studies on the susceptibility of a large urban population of Aedes aegypti to infection with dengue viruses. Southeast Asian J Trop Med Public Health 9: 494-500.

1823. Nalongsack S, Yoshida Y, Morita S, Sosouphanh K, Sakamoto J (2009) Knowledge, attitude and practice regarding dengue among people in Pakse, Laos. Nagoya J Med Sci 71: 29-37.

1824. Nam VS, Yen NT, Holynska M, Reid JW, Kay BH (2000) National progress in dengue vector control in Vietnam: survey for Mesocyclops (Copepoda), Micronecta (Corixidae), and fish as biological control agents. Am J Trop Med Hyg 62: 5-10.

1825. Narayanan M, Aravind MA, Thilothammal N, Prema R, Sargunam CS, et al. (2002) Dengue fever epidemic in Chennai--a study of clinical profile and outcome. Indian Pediatr 39: 1027-1033.

1826. Naresh G, Kulkarni AV, Sinha N, Jhamb N, Gulati S (2008) Dengue hemorrhagic fever complicated with encephalopathy and myocarditis: a case report. J Commun Dis 40: 223-224.

1827. Nart P (2001) DENGUE/DHF - SINGAPORE (02). Straits Times Interactive, 5 Aug 2001 [edited] ed: PROMED.

1828. Nart P (2001) DENGUE/DHF UPDATES (09): 15 AUG 2001 [Philippines]. PROMED.

1829. Nart P (2001) DENGUE/DHF - THAILAND. Xinhua News Agency, Thu 16 Aug 2001 [edited] ed: PROMED.

1830. Nart P (2001) DENGUE/DHF UPDATES (04): 18 MAR 2001 [El Salvador]. In: ProMED-mail, editor. Glasgow: PROMED.

1831. Nart P (2001) DENGUE/DHF UPDATES (17): 1 NOV 2001 [Martinique]. Orlando Sentinel, Fri 26 Oct 2001 [edited] ed: PROMED.

1832. Nart P (2001) DENGUE/DHF UPDATES (20): 28 NOV 2001 [Panama]. Agencia EFE (via COMTEX), Wed 21 Nov 2001 [edited] ed: PROMED.

1833. Nart P (2001) DENGUE/DHF UPDATES (12): 1 OCT 2001 [Philippines]. In: ProMED-mail, editor. Philippine Daily Inquirer 26 Sep 2001 [edited] ed: PROMED.

1834. Nart P (2001) DENGUE/DHF - CHINA (HONG KONG, MACAU) (04). PROMED.

1835. Nart P (2001) DENGUE/DHF - MEXICO (YUCATAN). Diario de Yucatan, 3 Sep 2001 [edited] ed: PROMED.

1836. Nart P (2001) DENGUE/DHF - MEXICO. Yahoo, Tue 4 Sep 2001 [edited] ed: PROMED.

1837. Nart P (2001) DENGUE/DHF UPDATES (11): 11 SEP 2001 [Philippines/Costa Rica]. In: ProMED-mail, editor. La Nacion Costa Rica, Fri 7 Sep 2001 [trans. by Mod. MPP and edited]; Philippine Daily Inquirer, Wed 5 Sep 2001, and Xinhua News Agency, Wed 5 Sep 2001 [edited] ed: PROMED.

1838. Nart P (2001) DENGUE/DHF - VIETNAM (02). Associated Press Worldstream, 13 Sep 2001 [edited] ed: PROMED.

1839. Nart P (2002) DENGUE/DHF UPDATES (15): 19 APR 2002 [Cook Islands/Thailand]. Pacific Islands News (PACNEWS), Wed 17 April 2002 [edited] ed: PROMED.

1840. Nart P (2002) DENGUE/DHF UPDATES (32): 19 AUG 2002 [Taiwan]. Central News Agency (Taipei), Sun 11 Aug 2002 [edited] ed: PROMED.

1841. Nart P (2002) DENGUE/DHF UPDATES (05): 8 FEB 2002 [Cuba/Malaysia]. EFE news service, Sat 2 Feb 2002 [edited] ed: PROMED.

1842. Nart P (2002) DENGUE/DHF UPDATES (01): 14 JAN 2002 [Indonesia]. In: ProMED-mail, editor. Jakarta Post (via AP Worldstream), Fri 11 Jan 2002 [edited] ed: PROMED.

1843. Nart P (2002) DENGUE/DHF UPDATES (25): 1 JUL 2002 [Sri Lanka/ Malaysia/ El Salvador/ Brazil]. In: ProMED-mail, editor. The Times of India, Wed 26 Jun 2002 [edited] ed: PROMED.

1844. Nart P (2002) DENGUE/DHF UPDATES (29): 29 JUL 2002 [Taiwan/ Malaysia/ India/ Bangladesh]. In: ProMED-mail, editor. Taiwan News, via Newsline, Tue 23 Jul 2002 [edited] ed: PROMED.

1845. Nart P (2002) DENGUE/DHF UPDATES (08): 1 MAR 2002 [Indonesia]. In: ProMED-mail, editor. The Jakarta Post, Sun 24 Feb 2002[edited] ed: PROMED.

1846. Nart P (2002) DENGUE/DHF UPDATES (19): 19 MAY 2002 [Australia/Nicaragua]. In: ProMED-mail, editor. Townsville Bulletin (Queensland, Australia) ed: PROMED.

1847. Nart P (2002) DENGUE/DHF UPDATES (45): 18 NOV 2002 [Philippines/ Mexico]. In: ProMED-mail, editor. Philippine Daily Inquirer via Newsline, Tue 12 Nov 2002 [edited] ed: PROMED.

1848. Nart P (2002) DENGUE/DHF UPDATES (41): 21 OCT 2002 [India/ New Caledonia/ Panama]. In: ProMED-mail, editor. The Times of India, Tue 15 Oct 2002 [edited] ed: PROMED.

1849. Nart P (2002) DENGUE/DHF UPDATES (36): 16 SEP 2002 [Malaysia]. In: ProMED-mail, editor. New Straits Times, Fri 13 Sep 2002 [edited] ed: PROMED.

1850. Nart P (2003) DENGUE/DHF UPDATE 2003 (16) [Australia]. In: ProMED-mail, editor. Ninemsn.com, Tue 15 Apr 2003 [edited] ed: PROMED.

1851. Nart P (2003) DENGUE/DHF UPDATE 2003 (17) [New Caledonia/ Brazil/ Guatemala]. In: ProMED-mail, editor. Australian Broadcasting Corporation, Wed 23 Apr 2003 [edited] ed: PROMED.

1852. Nart P (2003) DENGUE/DHF UPDATE 2003 (31) [Malaysia/ Panama]. In: ProMED-mail, editor. The Star (Malaysia), Wed 30 Jul 20003 [edited] ed: PROMED.

1853. Nart P (2003) DENGUE/DHF UPDATE 2003 (05) [Mexico/ Java]. In: ProMED-mail, editor. Reforma, Sat 1 Feb 2003 (in Spanish, translated by JW) [edited] ed: PROMED.

1854. Nart P (2003) DENGUE/DHF UPDATE 2003 (07) [Bolivia/ Philippines]. In: ProMED-mail, editor. Los Tiempos (Bolivia), Fri 7 Feb 2003 [in Spanish; English summary by Mod.JW] ed: PROMED.

1855. Nart P (2003) DENGUE/DHF UPDATE 2003 (01) [Malaysia/ Bolivia/ Dominican Republic]. In: ProMED-mail, editor. New Straits Times online, Sat 28 Dec 2002 [edited] ed: PROMED.

1856. Nart P (2003) DENGUE/DHF UPDATE 2003 (28) [Costa Rica]. In: ProMED-mail, editor. La Nacion, Costa Rica, Tue 8 Jul 2003 [in Spanish, translated and edited by Mod.JW] ed: PROMED.

1857. Nart P (2003) DENGUE/DHF UPDATE 2003 (25) [India/ Myanmar/ Laos/ Honduras]. In: ProMED-mail, editor. The Times of India, Tue 17 Jun 2003 [edited] ed: PROMED.

1858. Nart P (2003) DENGUE/DHF UPDATE 2003 (18) [Costa Rica]. In: ProMED-mail, editor. La Nacion (San Jose, Costa Rica), Wed 30 Apr 2003 (Translated by Maria Jacobs, ProMED-ESP) [edited] ed: PROMED.

1859. Nart P (2004) DENGUE/DHF UPDATE 2004 (26) [Panama]. In: ProMED-mail, editor. La Prensa, Panama, 25 Aug 2004 [in Spanish, translated by Mod.JW, edited] ed: PROMED.

1860. Nart P (2002) DENGUE/DHF UPDATES (22): 10 JUN 2002 [El Salvador]. In: ProMED-mail, editor. Agence France Press, via Newsline, Tue 4 Jun 2002 [edited] ed: PROMED.

1861. Nart P (2002) DENGUE/DHF UPDATES (23): 17 JUN 2002 [Cambodia]. In: ProMED-mail, editor. Xinhua News Agency, Tue 11 June 2002 [edited] ed: PROMED.

1862. Nart P (2004) DENGUE/DHF UPDATE 2005 (02). PROMED.

1863. Nart P (2005) DENGUE/DHF UPDATE 2005 (30). PROMED.

1864. Nart P (2006) DENGUE/DHF UPDATE 2006 (10). PROMED.

1865. Nascimento EJ, Silva AM, Cordeiro MT, Brito CA, Gil LH, et al. (2009) Alternative complement pathway deregulation is correlated with dengue severity. PLoS One 4: e6782.

1866. Nasim A (2009) Dengue Fever presenting as acute acalculous cholecystitis. J Coll Physicians Surg Pak 19: 531-533.

1867. Nathin MA, Harun SR, Sumarmo (1988) Dengue haemorrhagic fever and Japanese B encephalitis in Indonesia. Southeast Asian J Trop Med Public Health 19: 475-481.

1868. Natiello M, Ritacco V, Morales MA, Deodato B, Picollo M, et al. (2008) Indigenous dengue fever, Buenos Aires, Argentina. Emerg Infect Dis 14: 1498-1499.

1869. Navarrete-Espinosa J, Acevedo-Vales JA, Huerta-Hernandez E, Torres-Barranca J, Gavalon-Rosas DG (2006) Prevalencia de anticuerpos contra dengue y leptospira en la población de Jáltipan, Veracruz. Salud Publica Mex 48: 220-228.

1870. Navarrete-Espinosa J, Cuervo-Hernandez NM, Vazquez-Martinez JL (2008) Dengue hemorrágico sin hemorragias: ¿otra categoría? Gac Med Mex 144: 105-110.

1871. Navarrete-Espinosa J, Gomez-Dantes H (2006) Arbovirus causales de fiebre hemorragica en pacientes del Instituto Mexicano del Seguro Social. Rev Med Inst Mex Seguro Soc 44: 347-353.

1872. Navarrete-Espinosa J, Gomez-Dantes H, Celis-Quintal JG, Vazquez-Martinez JL (2005) Clinical profile of dengue hemorrhagic fever cases in Mexico. Salud Publica Mex 47: 193-200.

1873. Nawa M, Takasaki T, Ito M, Inoue S, Morita K, et al. (2005) Immunoglobulin A antibody responses in dengue patients: A useful marker for serodiagnosis of dengue virus infection. Clin Diagn Lab Immunol 12: 1235-1237.

1874. Neeraja M, Lakshmi V, Teja VD, Umabala P, Subbalakshmi MV (2006) Serodiagnosis of dengue virus infection in patients presenting to a tertiary care hospital. Indian J Med Microbiol 24: 280-282.

1875. Neff JM, Morris L, Gonzalez-Alcover R, Coleman PH, Lyss SB, et al. (1967) Dengue fever in a Puerto Rican community. Am J Epidemiol 86: 162-184.

1876. Neto RJP, Lima DM, de Paula SO, Lima CM, Rocco IM, et al. (2005) Molecular epidemiology of type 1 and 2 dengue viruses in Brazil from 1988 to 2001. Braz J Med Biol Res 38: 843-852.

1877. Nevalainen TJ, Losacker W (1997) Serum phospholipase A2 in dengue. J Infect 35: 251-252.

1878. Neves-Souza PCF, Azeredo EL, Zagne SMO, Valles-de-Souza R, Reis SRNI, et al. (2005) Inducible nitric oxide synthase (iNOS) expression in monocytes during acute Dengue Fever in patients and during in vitro infection. BMC Infect Dis 5: -.

1879. Ng CFS, Lum LCS, Ismail NA, Tan LH, Tan CPL (2007) Clinicians' diagnostic practice of dengue infections. J Clin Virol 40: 202-206.

1880. Nga TTT, Thai KTD, Phuong HL, Giao PT, Hung LQ, et al. (2007) Evaluation of two rapid immunochromatographic assays for diagnosis of dengue among Vietnamese febrile patients. Clin Vaccine Immunol 14: 799-801.

1881. Nguyen TH, Nguyen TL, Lei HY, Lin YS, Le BL, et al. (2005) Association between sex, nutritional status, severity of dengue hemorrhagic fever, and immune status in infants with dengue hemorrhagic fever. Am J Trop Med Hyg 72: 370-374.

1882. Nguyen TH, Nguyen TL, Lei HY, Lin YS, Le BL, et al. (2006) Volume replacement in infants with dengue hemorrhagic fever/dengue shock syndrome. Am J Trop Med Hyg 74: 684-691.

1883. Nicolai C (1998) DENGUE - BRAZIL (RIO DE JANEIRO) (02). Rio de Janeiro: PROMED.

1884. Nicolai C (1998) DENGUE/DHF - BRAZIL (RIO DE JANEIRO) (02. Rio de Janeiro: PROMED.

1885. Nilsson J, Vene S, Mattsson L (2005) Dengue encephalitis in a Swedish traveller returning from Thailand. Scand J Infect Dis 37: 776-778.

1886. Nisalak A, Endy TP, Nimmannitya S, Kalayanarooj S, Thisayakorn U, et al. (2003) Serotype-specific dengue virus circulation and dengue disease in Bangkok, Thailand from 1973 to 1999. Am J Trop Med Hyg 68: 191-202.

1887. Nitatpattana N, Singhasivanon P, Kiyoshi H, Andrianasolo H, Yoksan S, et al. (2007) Potential association of dengue hemorrhagic fever incidence and remote senses land surface temperature, Thailand, 1998. Southeast Asian J Trop Med Public Health 38: 427-433.

1888. Niyomrattanakit P, Winoyanuwattikun P, Chanprapaph S, Angsuthanasombat C, Panyim S, et al. (2004) Identification of residues in the dengue virus type 2 NS2B cofactor that are critical for NS3 protease activation. J Virol 78: 13708-13716.

1889. Niyomrattanakit P, Yahorava S, Mutule I, Mutulis F, Petrovska R, et al. (2006) Probing the substrate specificity of the Dengue virus type 2 NS3 serine protease by using internally quenched fluorescent peptides. Biochem J.

1890. Nogueira MB, Stella V, Bordignon J, Batista WC, de Borba L, et al. (2008) Evidence for the co-circulation of dengue virus type 3 genotypes III and V in the Northern region of Brazil during the 2002-2004 epidemics. Mem Inst Oswaldo Cruz 103: 483-U428.

1891. Nogueira RM, Filippis AM, Coelho JM, Sequeira PC, Schatzmayr HG, et al. (2002) Dengue virus infection of the central nervous system (CNS): a case report from Brazil. Southeast Asian J Trop Med Public Health 33: 68-71.

1892. Nogueira RM, Miagostovich MP, Cavalcanti SM, Marzochi KB, Schatzmayr HG (1992) Levels of IgM antibodies against dengue virus in Rio de Janeiro, Brazil. Res Virol 143: 423-427.

1893. Nogueira RM, Miagostovich MP, de Filippis AM, Pereira MA, Schatzmayr HG (2001) Dengue virus type 3 in Rio de Janeiro, Brazil. Mem Inst Oswaldo Cruz 96: 925-926.

1894. Nogueira RM, Miagostovich MP, Lampe E, Souza RW, Zagne SM, et al. (1993) Dengue epidemic in the stage of Rio de Janeiro, Brazil, 1990-1: co-circulation of dengue 1 and dengue 2 serotypes. Epidemiol Infect 111: 163-170.

1895. Nogueira RM, Miagostovich MP, Schatzmayr HG (2000) Molecular epidemiology of dengue viruses in Brazil. Cad Saude Publica 16: 205-211.

1896. Nogueira RM, Miagostovich MP, Schatzmayr HG, dos Santos FB, de Araujo ES, et al. (1999) Dengue in the State of Rio de Janeiro, Brazil, 1986-1998. Mem Inst Oswaldo Cruz 94: 297-304.

1897. Nogueira RM, Miagostovich MP, Schatzmayr HG, Moraes GC, Cardoso MA, et al. (1995) Dengue type 2 outbreak in the south of the state of Bahia, Brazil: laboratorial and epidemiological studies. Rev Inst Med Trop Sao Paulo 37: 507-510.

1898. Nogueira RM, Schatzmayr HG, Miagostovich MP, Farias MF, Farias Filho JD (1988) Virological study of a dengue type 1 epidemic at Rio de Janeiro. Mem Inst Oswaldo Cruz 83: 219-225.

1899. Nogueira RMR, de Araujo JMG, Schatzmayri HG (2007) Dengue viruses in Brazil, 1986-2006. Rev Panam Salud Publica 22: 358-363.

1900. Nogueira RMR, Schatzmayr HC, de Filippis AMB, dos Santos FB, da Cunha RV, et al. (2005) Dengue virus type 3, Brazil, 2002. Emerg Infect Dis 11: 1376-1381.

1901. Noisakran S, Dechtawewat T, Rinkaewkan P, Puttikhunt C, Kanjanahaluethai A, et al. (2007) Characterization of dengue virus NS1 stably expressed in 293T cell lines. J Virol Methods 142: 67-80.

1902. Noisakran S, Gibbons RV, Songprakhon P, Jairungsri A, Ajariyakhajorn C, et al. (2009) Detection of Dengue Virus in Platelets Isolated from Dengue Patients. Southeast Asian J Trop Med Public Health 40: 253-262.

1903. Norlijah O, Khamisah AN, Kamarul A, Paeds M, Mangalam S (2006) Repeated tourniquet testing as a diagnostic tool in dengue infection. Med J Malaysia 61: 22-27.

1904. Norman G, Theodre A, Joseph A (1991) An insular outbreak of dengue fever in a rural south Indian village. J Commun Dis 23: 185-190.

1905. Norwood CG, Larocco A, Laravia DA, Moore RW, Pittman-Cooley L (1999) Dengue fever risk and prevention for short-term group travelers to tropical regions. J La State Med Soc 151: 313-318.

1906. Nospam PL (2005) DENGUE CUBA (HAVANA): REQUEST FOR INFORMATION. PROMED.

1907. Nucci MC, Leach PGL (2007) Lie integrable cases of the simplified multistrain/two-stream model for tuberculosis and dengue fever. Journal of Mathematical Analysis and Applications 333: 430-449.

1908. Nuegoonpipat AA, Berlioz-Arthaud A, Chow V, Endy T, Lowry K, et al. (2004) Sustained transmission of dengue virus type 1 in the Pacific due to repeated introductions of different Asian strains. Virology 329: 505-512.

1909. Nukui Y, Tajima S, Kotaki A, Ito M, Takasaki T, et al. (2006) Novel dengue virus type 1 from travelers to Yap State, Micronesia. Emerg Infect Dis 12: 343-346.

1910. Nunes-Araujo FR, Ferreira MS, Nishioka SD (2003) Dengue fever in Brazilian adults and children: assessment of clinical findings and their validity for diagnosis. Ann Trop Med Parasitol 97: 415-419.

1911. Nunez M (2001) DENGUE/DHF - VENEZUELA (04). In: Maria Jacobs P-E, editor. El Nacional, 10 Sep 2001 [edited & translated by MJ] ed: PROMED.

1912. O'Brien DP, Leder K, Matchett E, Brown GV, Torresi J (2006) Illness in returned travelers and immigrants/refugees: The 6-year experience of two Australian infectious diseases. J Travel Med 13: 145-152.

1913. Ocampo CB, Wesson DM (2004) Population dynamics of Aedes aegypti from a dengue hyperendemic urban setting in Colombia. Am J Trop Med Hyg 71: 506-513.

1914. Ocazionez RE, Cortes FM, Villar LA, Gomez SY (2006) Temporal distribution of dengue virus serotypes in Colombian endemic area and dengue incidence. Re-introduction of dengue-3 associated to mild febrile illness and primary infection. Mem Inst Oswaldo Cruz 101: 725-731.

1915. Ocazionez RE, Gomez SY, Cortes FM (2007) Serotipo, Patron de Infeccion y Dengue Hemorragico en Area Endemica Colombiana. Revista de Salud Pública (Bogotá, Colombia) 9: 262-274.

1916. Odda J, Kristensen S, Kabasa J, Waako P (2008) Larvicidal activity of Combretum collinum Fresen against Aedes aegypti. J Vector Borne Dis 45: 321-324.

1917. Oishi K, Saito M, Mapua CA, Natividad FF (2007) Dengue illness: clinical features and pathogenesis. J Infect Chemother 13: 125-133.

1918. Okanurak K, Sornmani S, Indaratna K (1997) The cost of dengue hemorrhagic fever in Thailand. Southeast Asian J Trop Med Public Health 28: 711-717.

1919. Okuno Y, Fukunaga T, Srisupaluck S, Kasemsarn P, Dharakul C, et al. (1980) Serological and virological studies on patients with dengue hemorrhagic fever (DHF) in Chanthaburi province, Thailand. I. Serological studies on paired sera from DHF patients by neutralization (N), hemagglutination inhibition (HI) and staining tests. Biken J 23: 113-121.

1920. Okuno Y, Fukunaga T, Tadano M, Fukai K, Ikeda T, et al. (1983) Serological studies on volunteers inoculated experimentally with a dengue virus strain in 1943. Biken J 26: 161-163.

1921. Okuno Y, Harada T, Ogawa M, Okamoto Y, Maeda K (1989) A case of dengue hemorrhagic fever in a Japanese child. Microbiol Immunol 33: 649-655.

1922. Ong A, Sandar M, Chen MI, Sin LY (2007) Fatal dengue hemorrhagic fever in adults during a dengue epidemic in Singapore. Int J Infect Dis 11: 263-267.

1923. Ong SH, Yip JT, Chen YL, Liu W, Harun S, et al. (2008) Periodic re-emergence of endemic strains with strong epidemic potential - A proposed explanation for the 2004 Indonesian dengue epidemic. Infect Genet Evol 8: 191-204.

1924. Ooi EE, Goh KT, Gubler DJ (2006) Denque prevention and 35 years of vector control in Singapore. Emerg Infect Dis 12: 887-893.

1925. Ooi EE, Gubler DJ (2009) Dengue in Southeast Asia: epidemiological characteristics and strategic challenges in disease prevention. Cad Saude Publica 25: S115-S124.

1926. Ooi EE, Hart TJ, Tan HC, Chan SH (2001) Dengue seroepidemiology in Singapore. Lancet 357: 685-686.

1927. Osaka K, Ha DQ, Sakakihara Y, Khiem HB, Umenai T (1999) Control of dengue fever with active surveillance and the use of insecticidal aerosol cans. Southeast Asian J Trop Med Public Health 30: 484-488.

1928. Osman O, Fong MY, Devi S (2008) Complete genome sequence analysis of dengue virus type 2 isolated in Brunei. Virus Res 135: 48-52.

1929. Osman O, Fong MY, Sekaran SD (2009) Genetic characterization of dengue virus type 1 isolated in Brunei in 2005-2006. J Gen Virol 90: 678-686.

1930. Osorio J, Carvajal C, Sussman O, Buitrago R, Franco-Paredesa C (2008) Acute liver failure due to dengue virus infection. Int J Infect Dis 12: 444-445.

1931. Ottolenghi A (1997) DENGUE -ECUADOR. Columbus, OHIO: PROMED.

1932. Ottolenghi A (2000) DENGUE - ECUADOR (02. Hoy, on line, 19 Mar 2000 ed. Columbus, Ohio: PROMED.

1933. Padbidri VS, Adhikari P, Thakare JP, Ilkal MA, Joshi GD, et al. (1995) The 1993 epidemic of dengue fever in Mangalore, Karnataka state, India. Southeast Asian J Trop Med Public Health 26: 699-704.

1934. Paes MV, Pinhao AT, Barreto DF, Costa SM, Oliveira MP, et al. (2005) Liver injury and viremia in mice infected with dengue-2 virus. Virology 338: 236-246.

1935. Pai HH, Lu YL, Hong YJ, Hsu EL (2005) The differences of dengue vectors and human behavior between families with and without members having dengue fever/dengue hemorrhagic fever. Int J Environ Health Res 15: 263-269.

1936. Palacio M (1995) DENGUE-3 SPREADS IN NICARAGUA, PANAMA. In: File CWPD, editor. International Notes: PROMED.

1937. Palanca-Tan R (2008) The demand for a dengue vaccine: A contingent valuation survey in Metro Manila. Vaccine 26: 914-923.

1938. Palangchao H (2001) DENGUE/DHF UPDATES (07): 23 JUL 2001 [Philippines/Vietnam]. In: ProMED-mail, editor. Sun Star Wed 4 Jul 2001 [edited] ed: PROMED.

1939. Palma-da Cunha-Matta A, Soares-Moreno SA, Cardoso-de Almeida A, Aquilera-de Freitas V, Carod-Artal FJ (2004) Complicaciones neurológicas de la infección por e1 virus de1 dengue. Rev Neurol 39: 233-237.

1940. Palmer CJ, King SD, Cuadrado RR, Perez E, Baum M, et al. (1999) Evaluation of the MRL diagnostics dengue fever virus IgM capture ELISA and the PanBio Rapid Immunochromatographic Test for diagnosis of dengue fever in Jamaica. J Clin Microbiol 37: 1600-1601.

1941. Palmer CJ, Validum L, Vorndam VA, Clark GG, Validum C, et al. (1999) Dengue in Guyana. Lancet 354: 304.

1942. Palmer DR, Sun PF, Celluzzi C, Bisbing J, Pang SN, et al. (2005) Differential effects of dengue virus on infected and bystander dendritic cells. J Virol 79: 2432-2439.

1943. Pan HY, Chow JS (1984) A case of hemorrhagic dengue without hypovolemia in an adult. Trop Geogr Med 36: 305-307.

1944. Panagos A, Lacy ER, Gubler DJ, Macpherson CNL (2005) Dengue in Grenada. Rev Panam Salud Publica 17: 225-229.

1945. Pancharoen C, Chansongsakul T, Bhattarakosol P (2000) Causes of fever in children with first febrile seizures: how common are human herpesvirus-6 and dengue virus infections? Southeast Asian J Trop Med Public Health 31: 521-523.

1946. Pancharoen C, Kulwichit W, Tantawichien T, Thisyakorn U, Thisyakorn C (2002) Dengue infection: a global concern. J Med Assoc Thai 85 Suppl 1: S25-33.

1947. Pancharoen C, Mekmullica J, Thisyakorn U (2001) Primary dengue infection: what are the clinical distinctions from secondary infection? Southeast Asian J Trop Med Public Health 32: 476-480.

1948. Pancharoen C, Thisyakorn U (2001) Neurological manifestations in dengue patients. Southeast Asian J Trop Med Public Health 32: 341-345.

1949. Pandey A, Diddi K, Dar L, Bharaj P, Chahar HS, et al. (2007) The evolution of dengue over a decade in Delhi, India. J Clin Virol 40: 87-88.

1950. Pandey BD, Igarashi A (2000) Severity-related molecular differences among nineteen strains of dengue type 2 viruses. Microbiol Immunol 44: 179-188.

1951. Pandey BD, Morita K, Hasebe F, Parquet MC, Igarashi A (2000) Molecular evolution, distribution and genetic relationship among the dengue 2 viruses isolated from different clinical severity. Southeast Asian J Trop Med Public Health 31: 266-272.

1952. Pandey BD, Rai SK, Morita K, Kurane I (2004) First case of Dengue virus infection in Nepal. Nepal Medical College journal 6: 157-159.

1953. Pang T, Lam SK, Kok ML, Kok KY, Tho YC (1989) A practical community-based approach to the diagnosis of dengue virus infections. Singapore Med J 30: 525-527.

1954. Panning M, Wichmann D, Grywna K, Annan A, Wijesinghe S, et al. (2009) No evidence of chikungunya virus and antibodies shortly before the outbreak on Sri Lanka. Med Microbiol Immunol 198: 103-106.

1955. Panpanich R, Sornchai P, Kanjanaratanakorn K (2006) Corticosteroids for treating dengue shock syndrome. Cochrane Database Syst Rev: -.

1956. Papaevangelou G, Halstead SB (1977) Infections with two dengue viruses in Greece in the 20th century. Did dengue hemorrhagic fever occur in the 1928 epidemic? J Trop Med Hyg 80: 46-51.

1957. Paramasivan R, Thenmozhi V, Kabilan L, Tewari SC, Arunachalam N, et al. (2006) Seroepidemiology of a focal outbreak of dengue in Tamil Nadu. Indian J Med Res 124: 718-720.

1958. Parc F, Pichon G, Tetaria C, Louis F, Laigret J (1981) La dengue due au virus de type 4 en Polynesie Francaise. I.--Epidemiologie generale aspects cliniques particuliers. Med Trop (Mars) 41: 93-96.

1959. Parc F, Tetaria C, Pichon G (1981) La dengue due au virus de type 4 en Polynesie Francaise. II.--Observations biologiques liminaires sur quelques points precis d'epidemiologie et de physiopathologie. Med Trop (Mars) 41: 97-102.

1960. Parida M, Horioke K, Ishida H, Dash PK, Saxena P, et al. (2005) Rapid detection and differentiation of dengue virus serotypes by a real-time reverse transcription-loop-mediated isothermal amplification assay. J Clin Microbiol 43: 2895-2903.

1961. Parida MM, Dash PK, Upadhyay C, Saxena P, Jana AM (2002) Serological & virological investigation of an outbreak of dengue fever in Gwalior, India. Indian J Med Res 116: 248-254.

1962. Park SB, Ryu SY, Jin KB, Hwang EA, Han SY, et al. (2008) Acute colitis associated with dengue fever in a renal transplant recipient. Transplant Proc 40: 2431-2432.

1963. Parkash OM, Almas A, Shah SHA, Jafri W, Hamid S, et al. (2008) Severity of acute hepatitis and outcome in patients with dengue fever at a tertiary care center. J Gastroenterol Hepatol 23: A31-A31.

1964. Passos AD, Rodrigues EM, Dal-Fabbro AL (1998) Dengue control in Ribeirao Preto, Sao Paulo, Brazil. Cad Saude Publica 14 Suppl 2: 123-128.

1965. Passos MN, Santos LM, Pereira MR, Casali CG, Fortes Bde P, et al. (2004) Diferencas clinicas observadas em pacientes com dengue causadas por diferentes sorotipos na epidemia de 2001/2002, ocorrida no municipio do Rio de Janeiro. Rev Soc Bras Med Trop 37: 293-295.

1966. Passos SR, Bedoya SJ, Hokerberg YH, Maia SC, Georg I, et al. (2008) Clinical and laboratory signs as dengue markers during an outbreak in Rio de Janeiro. Infection 36: 570-574.

1967. Patey O, Ollivaud L, Breuil J, Lafaix C (1993) Unusual neurologic manifestations occurring during dengue fever infection. Am J Trop Med Hyg 48: 793-802.

1968. Paul RE, Patel AY, Mirza S, Fisher-Hoch SP, Luby SP (1998) Expansion of epidemic dengue viral infections to Pakistan. Int J Infect Dis 2: 197-201.

1969. Paupy C, Chantha N, Vazeille M, Reynes JM, Rodhain F, et al. (2003) Variation over space and time of Aedes aegypti in Phnom Penh (Cambodia): genetic structure and oral susceptibility to a dengue virus. Genet Res 82: 171-182.

1970. Paupy C, Girod R, Salvan M, Rodhain F, Failloux AB (2001) Population structure of Aedes albopictus from La Reunion Island (Indian Ocean) with respect to susceptibility to a dengue virus. Heredity 87: 273-283.

1971. Payne AF, Binduga-Gajewska I, Kauffman EB, Kramer LD (2006) Quantitation of flaviviruses by fluorescent focus assay. J Virol Methods.

1972. Peiris JS, Dittus WP, Ratnayake CB (1993) Seroepidemiology of dengue and other arboviruses in a natural population of toque macaques (Macaca sinica) at Polonnaruwa, Sri Lanka. J Med Primatol 22: 240-245.

1973. Pek DCK, Teoh SCB (2007) Ocular manifestations in dengue fever. Can J Ophthalmol 42: 755-755.

1974. Pelaez O, Guzman MG, Kouri G, Perez R, San Martin JL, et al. (2004) Dengue 3 epidemic, Havana, 2001. Emerg Infect Dis 10: 719-722.

1975. Penafiel A, Devanand A, Tan HK, Eng P (2006) Use of molecular adsorbent recirculating system in acute liver failure attributable to dengue hemorrhagic fever. J Intensive Care Med 21: 369-371.

1976. Peng WM, Man Y, Fan BC, Deng YQ, Tao J, et al. (2005) Simultaneous infection with dengue 2 and 3 viruses in a Chinese patient return from Sri Lanka. J Clin Virol 32: 194-198.

1977. Pengsaa K, Luxemburger C, Sabchareon A, Limkittikul K, Yoksan S, et al. (2006) Dengue virus infections in the first 2 years of life and the kinetics of transplacentally transferred dengue neutralizing antibodies in Thai children. J Infect Dis 194: 1570-1576.

1978. Pengsaa K, Yoksan S, Limkittikul K, Wisetsing P, Sirivichayakul C, et al. (2003) Maternally transferred neutralising dengue antibodies in Thai infants: a pilot study. Ann Trop Paediatr 23: 159-165.

1979. Penna G, Pinto LF, Soranz D, Glatt R (2009) High Incidence of Diseases Endemic to the Amazon Region of Brazil, 2001-2006. Emerg Infect Dis 15: 626-632.

1980. Penna ML (2003) Um desafio para a saude publica brasileira: o controle do dengue. Cad Saude Publica 19: 305-309.

1981. Pennel C (1998) DENGUE/DHF - HONDURAS (04). La Nacion Digital 19 Aug 1998 ed. Tegucigalpa: PROMED.

1982. Pennel C (1998) DENGUE/DHF - EL SALVADOR (SAN SALVADOR). In: ProMED-mail, editor. La Nacion - Costa Rica ed: PROMED.

1983. Pennel C (1999) DENGUE/DHF - HONDURAS (04). La Nacion, San Jose, Costa Rica. Tues 27 Jul 1999

<http://www.nacion.co.cr/> ed: PROMED.

1984. Penning HL (2001) DENGUE/DHF - MALAYSIA. The Times of India, Associated Press Report, Tue 12 Jun 2001 [edited] ed: PROMED.

1985. Penning HL (2002) DENGUE/DHF UPDATES (02): 21 JAN 2002 [Brazil]. In: ProMED-mail, editor. Yahoo Daily News, Reuters report, Fri 18 Jan 2002 [edited] <<http://dailynews.yahoo.com/h/nm/20020118/hl/dengue_3.html>> ed: PROMED.

1986. Penning HL, Pollack MP (2000) DENGUE/DHF - CENTRAL AMERICA. In: ProMED-mail, editor. 1- Reuters <<http://newsnet.reuters.com/cgi-bin/basketview.cgi?b=rcom:health&s=nN29413774>>

2- Agencia EFE (via COMTEX), filed 9 Aug 2000 6:15 PM EST [edited] ed: PROMED.

1987. Pepin KM, Hanley KA (2008) Density-Dependent Competitive Suppression of Sylvatic Dengue Virus by Endemic Dengue Virus in Cultured Mosquito Cells. Vector-Borne and Zoonotic Diseases 8: 821-828.

1988. Peragallo MS, Nicoletti L, Lista F, D'Amelio R (2003) Probable dengue virus infection among Italian troops, East Timor, 1999-2000. Emerg Infect Dis 9: 876-880.

1989. Perera N, Aonuma H, Yoshimura A, Teramoto T, Iseki H, et al. (2009) Rapid identification of virus-carrying mosquitoes using reverse transcription-loop-mediated isothermal amplification. J Virol Methods 156: 32-36.

1990. Perez AB, Garcia G, Sierra B, Alvarez M, Vazquez S, et al. (2004) IL-10 levels in Dengue patients: some findings from the exceptional epidemiological conditions in Cuba. J Med Virol 73: 230-234.

1991. Perez-Arellano JL, Bolanos-Rivero M, Angel-Moreno A (2006) Diagnóstico del paciente con fiebre importada. Med Clin (Barc) 127: 158-158.

1992. Periago MR, Guzman MG (2007) Dengue y dengue hemorragico en las Americas. Pan American Journal of Public Health 21: 187-191.

1993. Perich MJ, Bunner BL, Tidwell MA, Williams DC, Mara CD, et al. (1992) Penetration of ultra-low volume applied insecticide into dwellings for dengue vector control. J Am Mosq Control Assoc 8: 137-142.

1994. Perich MJ, Kardec A, Braga IA, Portal IF, Burge R, et al. (2003) Field evaluation of a lethal ovitrap against dengue vectors in Brazil. Med Vet Entomol 17: 205-210.

1995. Perret C (2002) DENGUE - CHILE (EASTER ISLAND) (04). Santiago, Chile: PROMED.

1996. Perret C, Abarca K, Ovalle J, Ferrer P, Godoy P, et al. (2003) Dengue-1 virus isolation during first dengue fever outbreak on Easter Island, Chile. Emerg Infect Dis 9: 1465-1467.

1997. Perret C, Chanthavanich P, Pengsaa K, Limkittikul K, Hutajaroen P, et al. (2005) Dengue infection during pregnancy and transplacental antibody transfer in Thai mothers. J Infect 51: 287-293.

1998. Pervin M, Tabassum S, Islam MN (2002) Isolation and serotyping of dengue viruses by mosquito inoculation technique from clinically suspected cases of dengue fever. Bangladesh Med Res Counc Bull 28: 104-111.

1999. Petdachai W, Sila'on J, Nimmannitya S, Nisalak A (2004) Neonatal dengue infection: report of dengue fever in a 1-day-old infant. Southeast Asian J Trop Med Public Health 35: 403-407.

2000. Peterson AT, Martinez-Campos C, Nakazawa Y, Martinez-Meyer E (2005) Time-specific ecological niche modeling predicts spatial dynamics of vector insects and human dengue cases. Trans R Soc Trop Med Hyg 99: 647-655.

2001. Pettengill D (2003) DENGUE/DHF UPDATE 2003 (47) [Australia]. In: ProMED-mail, editor. ABC News Online Tue 2 Dec 2003. 1:54pm (AEDT) [edited] ed: PROMED.

2002. Peyerl-Hoffmann G, Schwobel B, Jordan S, Vamisaveth V, Phetsouvanh R, et al. (2004) Serological investigation of the prevalence of anti-dengue IgM and IgG antibodies in Attapeu Province, South Laos. Clin Microbiol Infect 10: 181-184.

2003. Peyrefitte CN, Couissinier-Paris P, Mercier-Perennec V, Bessaud M, Martial J, et al. (2003) Genetic characterization of newly reintroduced dengue virus type 3 in Martinique (French West Indies). J Clin Microbiol 41: 5195-5198.

2004. Peyrefitte CN, Pastorino B, Grau GE, Lou J, Tolou H, et al. (2006) Dengue virus infection of human microvascular endothelial cells from different vascular beds promotes both common and specific functional changes. J Med Virol 78: 229-242.

2005. Peyrefitte CN, Pastorino BAM, Bessaud M, Gravier P, Tock F, et al. (2005) Dengue type 3 virus, Saint Martin, 2003-2004. Emerg Infect Dis 11: 757-761.

2006. Peyrefitte CN, Pastorino BAM, Bessaud M, Gravier P, Tock F, et al. (2004) Dengue type 3 virus, Saint Martin, 2003–2004. Emerging Infectious Dieseases 11: 757-761.

2007. Pham TB, Matheus S, Vu TQH, Deparis X, Marechal V (2009) Early clinical and biological features of severe clinical manifestations of dengue in Vietnamese adults. J Clin Virol 45: 276-280.

2008. Pham TB, Nguyen TH, Vu TQ, Nguyen TL, Malvy D (2007) Facteurs predictifs du syndrome de choc lie a la dengue chez les enfants a l'hopital des enfants malades n0 1, Ho-Chi-Minh ville, Vietnam. Bull Soc Pathol Exot Filiales 100: 43-47.

2009. Phan DT, Ha NT, Thuc LT, Diet NH, Phu LV, et al. (1991) Some changes in immunity and blood in relation to clinical states of dengue hemorrhagic fever patients in Vietnam. Haematologia (Budap) 24: 13-21.

2010. Philip Samuel P, Tyagi BK (2006) Diagnostic methods for detection & isolation of dengue viruses from vector mosquitoes. Indian J Med Res 123: 615-628.

2011. Phillips D (1997) DENGUE - AUSTRALIA (TORRES STRAIT ISLANDS) (02). In: Research WCCfARa, editor. Brisbane: PROMED.

2012. Phillips D (1997) DENGUE - SAMOA (02). In: Research WCCfARa, editor. Queensland: PROMED.

2013. Phillips D (1997) DENGUE - SAMOA (03). Pacific Public Health Surveillance Network

PACNET@LISTSERV.SPC.ORG.NC ed. (Australia): PROMED.

2014. Phillips I, Need J, Escamilla J, Colan E, Sanchez S, et al. (1992) First documented outbreak of dengue in the Peruvian Amazon region. Bull Pan Am Health Organ 26: 201-207.

2015. Phongsamart W, Yoksan S, Vanaprapa N, Chokephaibulkit K (2008) Dengue virus infection in late pregnancy and transmission to the infants. Pediatr Infect Dis J 27: 500-504.

2016. Phoolcharoen W, Smith DR (2004) Internalization of the dengue virus is cell cycle modulated in HepG2, but not vero cells. J Med Virol 74: 434-441.

2017. Phuanukoonnon S, Brough M, Bryan JH (2006) Folk knowledge about dengue mosquitoes and contributions of health belief model in dengue control promotion in Northeast Thailand. Acta Trop 99: 6-14.

2018. Phuong CX, Nhan NT, Kneen R, Thuy PT, van Thien C, et al. (2004) Clinical diagnosis and assessment of severity of confirmed dengue infections in Vietnamese children: is the world health organization classification system helpful? Am J Trop Med Hyg 70: 172-179.

2019. Phuong CXT, Nhan NT, Kneen R, Bethell D, Dep LT, et al. (2004) Evaluation of an algorithm for integrated management of childhood illness in an area of Vietnam with dengue transmission. Trop Med Int Health 9: 573-581.

2020. Phuong HL, De Vries PJ, Boonshuyar C, Binh TQ, Nam NV, et al. (2008) Dengue risk factors and community participation in Binh Thuan Province, Vietnam, a household survey. Southeast Asian J Trop Med Public Health 39: 79-89.

2021. Phuong HL, de Vries PJ, Nagelkerke N, Giao PT, Hung LQ, et al. (2006) Acute undifferentiated fever in Binh Thuan province, Vietnam: imprecise clinical diagnosis and irrational pharmaco-therapy. Trop Med Int Health 11: 869-879.

2022. Phuong HL, de Vries PJ, Nga TTT, T Giao P, Hung LQ, et al. (2006) Dengue as a cause of acute undifferentiated fever in Vietnam. BMC Infect Dis 6: -.

2023. Phuong HL, Thai KT, Nga TT, Giao PT, Hung le Q, et al. (2009) Detection of dengue nonstructural 1 (NS1) protein in Vietnamese patients with fever. Diagn Microbiol Infect Dis 63: 372-378.

2024. Pichainarong N, Mongkalangoon N, Kalayanarooj S, Chaveepojnkamjorn W (2006) Relationship between body size and severity of dengue hemorrhagic fever among children aged 0-14 years. Southeast Asian J Trop Med Public Health 37: 283-288.

2025. Pick N, Potasman I (1995) [Dengue fever]. Harefuah 129: 30-32, 78.

2026. Pickering Lopez JM (2005) DENGUE/DHF UPDATE 2005 (27). PROMED.

2027. Pierre Filho Pde T, Carvalho Filho JP, Pierre ET (2008) Bilateral acute angle closure glaucoma in a patient with dengue fever: case report. Arq Bras Oftalmol 71: 265-268.

2028. Pimentel LHC, Gondim FDA, Oliveira GR, MontAlverne FJ (2008) Dengue fever as a cause of benign acute chilhood viral myositis. Neurology 70: A257-A258.

2029. Pinazo Delgado MJ, Munoz Gutierrez J, Betica Radic L, Maretic T, Zekan S, et al. (2008) Imported dengue hemorrhagic fever, Europe. Emerg Infect Dis 14: 1329-1330.

2030. Pincus LB, Grossman ME, Fox LP (2008) The exanthem of dengue fever: Clinical features of two US tourists traveling abroad. J Am Acad Dermatol 58: 308-316.

2031. Pinheiro F (1995) DENGUE IN VENEZUELA - CORRECTION. In: PAHO, editor. Aragua State: PROMED.

2032. Pinheiro F (1995) DENGUE IN THE AMERICAS, 1995. In: PAHO, editor: PROMED.

2033. Pinheiro FP, Corber SJ (1997) Global situation of dengue and dengue haemorrhagic fever, and its emergence in the Americas. World Health Stat Q 50: 161-169.

2034. Pinheiro VCS, Tadei WP, Barros PMSS, Vasconcelos PFC, Cruz ACR (2005) Detection of dengue virus serotype 3 by reverse transcription-polymerase chain reaction in Aedes aegypti (Diptera, Culicidae) captured in Manaus, Amazonas. Mem Inst Oswaldo Cruz 100: 833-839.

2035. Pinto LM, Oliveira SA, Braga EL, Nogueira RM, Kubelka CF (1999) Increased pro-inflammatory cytokines (TNF-alpha and IL-6) and anti-inflammatory compounds (sTNFRp55 and sTNFRp75) in Brazilian patients during exanthematic dengue fever. Mem Inst Oswaldo Cruz 94: 387-394.

2036. Pires Neto RJ, Lima DM, de Paula SO, Lima CM, Rocco IM, et al. (2005) Molecular epidemiology of type 1 and 2 dengue viruses in Brazil from 1988 to 2001. Braz J Med Biol Res 38: 843-852.

2037. Platt KB, Mangiafico JA, Rocha OJ, Zaldivar ME, Mora J, et al. (2000) Detection of dengue virus neutralizing antibodies in bats from Costa Rica and Ecuador. J Med Entomol 37: 965-967.

2038. Playford EG, Phillips D, Looke DF, Whitby M (1998) Three cases of dengue 1 virus infection from islands in the Gulf of Thailand. Commun Dis Intell 22: 107-109.

2039. Poblap T, Nitatpattana N, Chaimarin A, Barbazan P, Chauvancy G, et al. (2006) Silent transmission of virus during a Dengue epidemic, Nakhon Pathom Province, Thailand 2001. Southeast Asian J Trop Med Public Health 37: 899-903.

2040. Podder G, Breiman RF, Azim T, Thu HM, Velathanthiri N, et al. (2006) Origin of dengue type 3 viruses associated with the dengue outbreak in Dhaka, Bangladesh, in 2000 and 2001. Am J Trop Med Hyg 74: 263-265.

2041. Poersch CD, Pavoni DP, Queiroz MH, de Borba L, Goldenberg S, et al. (2005) Dengue virus infections: comparison of methods for diagnosing the acute disease. J Clin Virol 32: 272-277.

2042. Pohan HT, Lie KC, Santoso WD, Eppy (2009) An open pilot study of the efficacy and safety of polygeline in adult subjects with dengue haemorrhagic Fever. Acta Medica Indonesiana 41: 47-53.

2043. Poli L, Chungue E, Soulignac O, Gestas P, Kuo P, et al. (1991) Dengue materno-foetale. A propos de 5 cas observes pendant l'epidemie de Tahiti (1989). Bull Soc Pathol Exot Filiales 84: 513-521.

2044. Polizel JR, Bueno D, Visentainer JE, Sell AM, Borelli SD, et al. (2004) Association of human leukocyte antigen DQ1 and dengue fever in a white Southern Brazilian population. Mem Inst Oswaldo Cruz 99: 559-562.

2045. Pollack MP (1999) DENGUE/DHF - COSTA RICA (04). La Nacion, San Jose, Costa Rica Thu 19 Aug 1999 [edited] ed: PROMED.

2046. Pollack MP (1999) DENGUE/DHF - SRI LANKA (02). In: ProMED-mail, editor. Sunday Observer (Sri Lanka), 8 Aug 1999 [edited] ed: PROMED.

2047. Pollack MP (1999) DENGUE/DHF - COSTA RICA (02). In: ProMED-mail, editor. La Nacion, San Jose, Costa Rica, 9 Jul 1999 ed: PROMED.

2048. Pollack MP (1999) DENGUE/DHF - HONDURAS: CORRECTION? In: ProMED-mail, editor. AFP 7 Jul 1999 [in Spanish] ed: PROMED.

2049. Pollack MP (1999) DENGUE - MEXICO (NUEVO LEON): ALERT. In: ProMED-mail, editor. EFE, 6 July 1999 [edited] ed: PROMED.

2050. Pollack MP (1999) DENGUE/DHF - COSTA RICA; ALERT. In: Ministry of Health CRDoE, Surveillance, editors. San Jose (Costa Rica): PROMED.

2051. Pollack MP (1999) DENGUE/DHF - HONDURAS. In: ProMED-mail, editor. LATNN.com Central American news desk [edited] ed. Tegucigalpa: PROMED.

2052. Pollack MP (1999) DENGUE/DHF ADVISORY - MALAYSIA (PERAK). The Sun, Malaysia, Fri 21 May 1999 ed: PROMED.

2053. Pollack MP (1999) DENGUE/DHF - HONDURAS (07). In: ProMED-mail, editor. media, 16 Nov 1999 ed: PROMED.

2054. Pollack MP (1999) DENGUE/DHF - MEXICO (NUEVO LEON, TAMAULIPAS). In: ProMED-mail, editor. Media reports ed: PROMED.

2055. Pollack MP (1999) DENGUE/DHF - PHILIPPINES (PANGASINAN). In: ProMED-mail, editor. News media, 22 Sep 1999 ed: PROMED.

2056. Pollack MP (1999) DENGUE/DHF - COSTA RICA (05). In: ProMED-mail, editor. La Nacion, San Jose, Costa Rica, 13 Sep 1999

<http://www.nacion.co.cr/ln_ee/1999/septiembre/13/pais5.html> ed: PROMED.

2057. Pollack MP (2000) DENGUE/DHF - CENTRAL AMERICA (02). In: ProMED-mail, editor. Agencia EFE (vua COMTEX): filed: Fri Aug 18, 2000 5:51 PM EST [edited] ed: PROMED.

2058. Pollack MP (2000) DENGUE/DHF - PHILIPPINES & EL SALVADOR. In: ProMED-mail, editor. Xinhua News Agency, 24 Jun 2000 10:50 AM EST [edited] ed: PROMED.

2059. Pollack MP (2000) DENGUE - BOLIVIA (SANTA CRUZ). In: ProMED-mail, editor. Newspaper Los Tiempos (Bolivia) 31 Mar 2000 [in Spanish] ed: PROMED.

2060. Pollock N (1995) DENGUE - PACIFIC ISLANDS. Palau, Cook Islands: PROMED.

2061. Polo-Sabau J, Molina-Robles M, Puente-Puente S (2006) Falso positivo para dengue tras estancia en Ecuador. Enferm Infecc Microbiol Clin 24: 67-67.

2062. Pongsumpun P, Lopez DG, Favier C, Torres L, Llosa J, et al. (2008) Dynamics of dengue epidemics in urban contexts. Trop Med Int Health 13: 1180-1187.

2063. Pongsumpun P, Patanarapelert K, Sriprom M, Varamit S, Tang IM (2004) Infection risk to travelers going to dengue fever endemic regions. Southeast Asian J Trop Med Public Health 35: 155-159.

2064. Pongsumpun P, Tang IM (2001) A realistic age structured transmission model for dengue hemorrhagic fever in Thailand. Southeast Asian J Trop Med Public Health 32: 336-340.

2065. Pongsumpun P, Yoksan S, Tan IM (2002) A comparison of the age distributions in the dengue hemorrhagic fever epidemics in Santiago de Cuba (1997) and Thailand (1998). Southeast Asian J Trop Med Public Health 33: 255-258.

2066. Pontes RJ, Dal Fabbro AL, Rocha Gde M, Santiago RC, Figueiredo LT, et al. (1991) Epidemia de dengue em Ribeirao Preto, SP, Brasil: nota previa. Rev Saude Publica 25: 315-317.

2067. Pontes RJ, Freeman J, Oliveira-Lima JW, Hodgson JC, Spielman A (2000) Vector densities that potentiate dengue outbreaks in a Brazilian city. Am J Trop Med Hyg 62: 378-383.

2068. Pontes RJ, Ruffino-Netto A (1994) Dengue em localidade urbana da regiao sudeste do Brasil: aspectos epidemiologicos. Rev Saude Publica 28: 218-227.

2069. Pontes RJ, Ruffino-Netto A (1997) Vigilancia e busca ativa de casos suspeitos de dengue hemorragico em Ribeirao Preto, Sao Paulo. Rev Panam Salud Publica 1: 186-192.

2070. Poovaneswari S, Lam SK (1992) Problems in dengue control: a case study. Southeast Asian J Trop Med Public Health 23: 723-725.

2071. Poovorawan Y, Hutagalung Y, Chongsrisawat V, Boudville I, Bock HL (2006) Dengue virus infection: a major cause of acute hepatic failure in Thai children. Ann Trop Paediatr 26: 17-23.

2072. Porter KR, Beckett CG, Kosasih H, Tan RI, Alisjahbana B, et al. (2005) Epidemiology of dengue and dengue hemorrhagic fever in a cohort of adults living in Bandung, west Java, Indonesia. Am J Trop Med Hyg 72: 60-66.

2073. Potasman I, Srugo I, Schwartz E (1999) Dengue seroconversion among Israeli travelers to tropical countries. Emerg Infect Dis 5: 824-827.

2074. Potts JA, Rothman AL (2008) Clinical and laboratory features that distinguish dengue from other febrile illnesses in endemic populations. Trop Med Int Health 13: 1328-1340.

2075. Prado I, Rosario D, Bernardo U, Alvarez M, Rodriguez R, et al. (2005) PCR detection of dengue virus using dried whole blood spotted on filter paper. J Virol Methods 125: 75-81.

2076. Pramuljo HS, Harun SR (1991) Ultrasound findings in dengue haemorrhagic fever. Pediatr Radiol 21: 100-102.

2077. Premaratna R, Bailey MS, Ratnasena BGN, de Silva HJ (2007) Dengue fever mimicking acute appendicitis. Trans R Soc Trop Med Hyg 101: 683-685.

2078. Premaratna R, Pathmeswaran A, Amarasekara NDDM, Motha MBC, Perera KVHKK, et al. (2009) A clinical guide for early detection of dengue fever and timing of investigations to detect patients likely to develop complications. Trans R Soc Trop Med Hyg 103: 127-131.

2079. Premkumar A, Horan CR, Gage PW (2005) Dengue virus M protein C-terminal peptide (DVM-C) forms ion channels. J Membr Biol 204: 33-38.

2080. Preslar D (1995) DENGUE IN GIBBON COLONY - THAILAND (2). In: Project AHEAD, editor. Washington: PROMED.

2081. Preslar D (1996) DENGUE/DHF - MEXICO (TABASCO) [ENGLISH/ESPANOL]. In: estatal SdS, [http://cenids.ssa.gob.mx](http://cenids.ssa.gob.mx/), editors. (Mexico): PROMED.

2082. Preslar D (1996) DENGUE HEMORRAGHIC FEVER - INDIA (4). In: Officer/AHEAD WP, editor. (US): PROMED.

2083. Preslar D (1996) DENGUE - VIETNAM. In: AHEAD P, editor. <http://www.fas.org/promed/> ed. Washington: PROMED.

2084. Preslar D (1997) DENGUE - VIETNAM. PROMED.

2085. Preslar D (1997) DENGUE - INDIA. In: ProMED/AHEAD/ProMED-mail, editor. Hindustan Times, 16 Sep 1997 ed: PROMED.

2086. Preslar D (1997) DENGUE - COOK ISLANDS: END. PROMED.

2087. Preslar D (1997) DENGUE/DHF - CUBA (04). CubaPress Independent Press Agency, Havana [in Spanish]

<http://www.cubapress.com/> ed. Havana: PROMED.

2088. Preslar D (1997) DENGUE - PHILIPPINES. In: Preslar DB, Officer P-AP, editors. Washington DC: PROMED.

2089. Preslar D (1997) DENGUE/DENGUE HEMORRHAGIC FEVER - MALAYSIA (02). In: Preslar DB, Officer P-AP, editors. Washington DC: PROMED.

2090. Preslar D (1998) DENGUE - AUSTRALIA (QUEENSLAND) (09). Australian General News, 2 Apr 1998 ed. (Queensland): PROMED.

2091. Preslar D (1998) DENGUE, INDIGENOUS - TAIWAN. PROMED.

2092. Preslar D (1998) DENGUE - INDONESIA (CENTRAL JAVA). In: ProMED-mail, editor. The Jakarta Post, 4 Dec 1998 ed: PROMED.

2093. Preslar D (1998) DENGUE-3 - PUERTO RICO. PROMED.

2094. Preslar D (1998) DENGUE - VIET NAM (02). In: ProMED-mail, editor. Thanh Nie, Hanoi, 3 July ed: PROMED.

2095. Preslar D (1998) DENGUE - INDONESIA (SUMATRA). In: ProMED-mail, editor: PROMED.

2096. Preslar D (1998) DENGUE/DHF - VIETNAM. In: ProMED-mail, editor: PROMED.

2097. Preslar D (1998) DENGUE/DHF, COMMONWEALTH GAMES - MALAYSIA. In: ProMED-mail, editor. AAP (Australia), Agence France Presse, AP Worldstream, 14 Sep 1998 ed: PROMED.

2098. Preslar D (1999) DENGUE/DHF, EPIDEMIC DECLARED - VENEZUELA. In: ProMED-mail, editor. Agence France Presse ed: PROMED.

2099. Pridgeon JW, Becnel JJ, Clark GC, Linthicum KJ (2009) A High-Throughput Screening Method to Identify Potential Pesticides for Mosquito Control. J Med Entomol 46: 335-341.

2100. Pridgeon JW, Becnel JJ, Clark GG, Linthicum KJ (2009) Permethrin Induces Overexpression of Cytochrome c Oxidase Subunit 3 in Aedes aegypti. J Med Entomol 46: 810-819.

2101. Promphan W, Sopontammarak S, Pruekprasert P, Kajornwattanakul W, Kongpattanayothin A (2004) Dengue myocarditis. Southeast Asian J Trop Med Public Health 35: 611-613.

2102. Pryor MJ, Azzola L, Wright PJ, Davidson AD (2004) Histidine 39 in the dengue virus type 2 M protein has an important role in virus assembly. J Gen Virol 85: 3627-3636.

2103. Pugliese A, Beltramo T, Torre D (2005) Seroprevalence study of Tick-borne encephalitis, Borrelia burgdorferi, Dengue and Toscana virus in Turin Province. Cell Biochem Funct.

2104. Pugliese A, Beltramo T, Torre D (2007) Seroprevalence study of Tick-borne encephalitis, Borrelia burgdorferi, Dengue and Toscana virus in Turin Province. Cell Biochem Funct 25: 185-188.

2105. Pungjitprapai A, Tantawichien T (2008) A fatal case of spontaneous rupture of the spleen due to dengue virus infection: case report and review. Southeast Asian J Trop Med Public Health 39: 383-386.

2106. Purdy DE, Chang GJJ (2005) Secretion of noninfectious dengue virus-like particles and identification of amino acids in the stem region involved in intracellular retention of envelope protein. Virology 333: 239-250.

2107. Pushpa V, Venkatadesikalu M, Mohan S, Cherian T, John TJ, et al. (1998) An epidemic of dengue haemorrhagic fever/dengue shock syndrome in tropical India. Ann Trop Paediatr 18: 289-293.

2108. Putnak JR, Coller BA, Voss G, Vaughn DW, Clements D, et al. (2005) An evaluation of dengue type-2 inactivated, recombinant subunit, and live-attenuated vaccine candidates in the rhesus macaque model. Vaccine 23: 4442-4452.

2109. Putonti C, Chumakov S, Mitra R, Fox GE, Willson RC, et al. (2006) Human-blind probes and primers for dengue virus identification - Exhaustive analysis of subsequences present in the human and 83 dengue genome sequences. FEBS J 273: 398-408.

2110. Putvatana R, Yoksan S, Chayayodhin T, Bhamarapravati N, Halstead SB (1984) Absence of dengue 2 infection enhancement in human sera containing Japanese encephalitis antibodies. Am J Trop Med Hyg 33: 288-294.

2111. Qin CF, Jiang T, Chen SP, Yu M, Qin ED (2005) [Capsid-targeted viral inactivation for dengue virus infection]. Wei Sheng Wu Xue Bao 45: 111-115.

2112. Qin CF, Qin E, Yu M, Chen SP, Jiang T, et al. (2005) Therapeutic effects of dengue 2 virus capsid protein and staphylococcal nuclease fusion protein on dengue-infected cell cultures. Arch Virol 150: 659-669.

2113. Qin CF, Qin ED (2006) Capsid-targeted viral inactivation can destroy dengue 2 virus from within in vitro. Arch Virol 151: 379-385.

2114. Qin CF, Qin ED (2004) Development of cell lines stably expressing staphylococcal nuclease fused to dengue 2 virus capsid protein for CTVI. Acta Biochim Biophys Sin 36: 577-582.

2115. Qiu FX, Chen QQ, Ho QY, Chen WZ, Zhao ZG, et al. (1991) The first epidemic of dengue hemorrhagic fever in the People's Republic of China. Am J Trop Med Hyg 44: 364-370.

2116. Qiu FX, Gubler DJ, Liu JC, Chen QQ (1993) Dengue in China: a clinical review. Bull World Health Organ 71: 349-359.

2117. Qiu LW, Di BA, Wen K, Wang XSA, Liang WH, et al. (2009) Development of an Antigen Capture Immunoassay Based on Monoclonal Antibodies Specific for Dengue Virus Serotype 2 Nonstructural Protein 1 for Early and Rapid Identification of Dengue Virus Serotype 2 Infections. Clin Vaccine Immunol 16: 88-95.

2118. Quina MA, Thein S, Auvanich W, Okuno Y, Igarashi A, et al. (1978) Changes in dengue and Japanese encephalitis (JE) antibody after JE vaccination. Biken J 21: 149-159.

2119. Quiroz E, Ortega M, Guzman MG, Vazquez S, Pelegrino JL, et al. (1997) Dengue en Panama, 1993. Rev Cuba Med Trop 49: 86-93.

2120. Quiroz RE (1998) DENGUE - PANAMA (03). In: Lambiz LM, editor. ICGES/MINSA ed. (Panama): PROMED.

2121. Quiroz-Moreno R, Mendez GF, Ovando-Rivera KM (2006) Utilidad clinica del ultrasonido en la identificacion de dengue hromorragico. Rev Med Inst Mex Seguro Soc 44: 243-248.

2122. Qureshi JA, Notta NJ, Salahuddin N, Zaman V, Khan JA (1997) An epidemic of Dengue fever in Karachi--associated clinical manifestations. JPMA J Pak Med Assoc 47: 178-181.

2123. Race MW, Williams MC, Agostini CF (1979) Dengue in the Caribbean: virus isolation in a mosquito (Aedes pseudoscutellaris) cell line. Trans R Soc Trop Med Hyg 73: 18-22.

2124. Radakovic-Fijan S, Graninger W, Muller C, Honigsmann H, Tanew A (2002) Dengue hemorrhagic fever in a British travel guide. J Am Acad Dermatol 46: 430-433.

2125. Raekiansyah M, Pramesyanti A, Bela B, Kosasih H, Ma'roef CN, et al. (2005) Genetic variations and relationship among dengue virus type 3 strains isolated from patients with mild or severe form of dengue disease in Indonesia and Thailand. Southeast Asian J Trop Med Public Health 36: 1187-1197.

2126. Raengsakulrach B, Nisalak A, Maneekarn N, Yenchitsomanus PT, Limsomwong C, et al. (2002) Comparison of four reverse transcription-polymerase chain reaction procedures for the detection of dengue virus in clinical specimens. J Virol Methods 105: 219-232.

2127. Raghupathy R, Chaturvedi UC, Al-Sayer H, Elbishbishi EA, Agarwal R, et al. (1998) Elevated levels of IL-8 in dengue hemorrhagic fever. J Med Virol 56: 280-285.

2128. Rahman M, Rahman K, Siddque AK, Shoma S, Kamal AH, et al. (2002) First outbreak of dengue hemorrhagic fever, Bangladesh. Emerg Infect Dis 8: 738-740.

2129. Rahman M, Siddique AK, Tam FC, Sharmin S, Rashid H, et al. (2007) Rapid detection of early typhoid fever in endemic community children by the TUBEX O9-antibody test. Diagn Microbiol Infect Dis 58: 275-281.

2130. Rahman MT, Tahmin HA, Mannan T, Sultana R (2007) Seropositivity and pattern of dengue infection in Dhaka city. Mymensingh Medical Journal 16: 204-208.

2131. Rajajee S, Ezhilarasi S, Rajarajan K (2005) Benign acute childhood myositis. Indian J Pediatr 72: 399-400.

2132. Rajapakse S (2009) Corticosteroids in the treatment of dengue illness. Trans R Soc Trop Med Hyg 103: 122-126.

2133. Rajendiran S, Lakshamanappa HS, Zachariah B, Nambiar S (2008) Desialylation of plasma proteins in severe dengue infection: Possible role of oxidative stress. Am J Trop Med Hyg 79: 372-377.

2134. Ram S, Khurana S, Kaushal V, Gupta R, Khurana SB (1998) Incidence of dengue fever in relation to climatic factors in Ludhiana, Punjab. Indian J Med Res 108: 128-133.

2135. Rama Krishna AK, Patil S, Srinivas Rao G, Kumar A (2006) Dengue fever presenting with acute colitis. Indian J Gastroenterol 25: 97-98.

2136. Ramchurn SK, Moheeput K, Goorah SS (2009) An analysis of a short-lived outbreak of dengue fever in Mauritius. Eurosurveillance 14.

2137. Ramirez-Ronda CH (1987) Dengue in Puerto Rico: clinical manifestations and management from 1960's to 1987. P R Health Sci J 6: 113-118.

2138. Ramirez-Ronda CH, Garcia CD (1994) Dengue in the Western Hemisphere. Infect Dis Clin North Am 8: 107-128.

2139. Ramirez-Ronda CH, Maldonado NM, Rabell V, Sather G, Cline BL (1979) Dengue hemorrhagic shock in the western hemisphere. Trop Geogr Med 31: 127-131.

2140. Ramirez-Zepeda MG, Velasco-Mondragon HE, Ramos C, Penuelas JE, Maradiaga-Cecena MA, et al. (2009) Caracterización clínica y epidemiológica de los casos de dengue: experiencia del Hospital General de Culiacán, Sinaloa, México. Rev Panam Salud Publica 25: 16-23.

2141. Ramos C, Sanchez G, Pando RH, Baquera J, Hernandez D, et al. (1998) Dengue virus in the brain of a fatal case of hemorrhagic dengue fever. J Neurovirol 4: 465-468.

2142. Ramos MM, Arguello DF, Luxemburger C, Quinones L, Munoz JL, et al. (2008) Epidemiological and clinical observations on patients with dengue in Puerto Rico: results from the first year of enhanced surveillance--June 2005-May 2006. Am J Trop Med Hyg 79: 123-127.

2143. Ramos MM, Mohammed H, Zielinski-Gutierrez E, Hayden MH, Lopez JLR, et al. (2008) Epidemic dengue and dengue hemorrhagic fever at the Texas-Mexico border: Results of a household-based seroepiderniologic survey, December 2005. Am J Trop Med Hyg 78: 364-369.

2144. Ramos-Castaneda J, Gonzalez C, Jimenez MA, Duran J, Hernandez-Martinez S, et al. (2008) Effect of Nitric Oxide on Dengue Virus Replication in Aedes aegypti and Anopheles albimanus. Intervirology 51: 335-341.

2145. Ranjit S, Kissoon N, Gandhi D, Dayal A, Rajeshwari N, et al. (2007) Early differentiation between dengue and septic shock by comparison of admission hemodynamic, clinical, and laboratory variables - A pilot study. Pediatr Emerg Care 23: 368-375.

2146. Ranjit S, Kissoon N, Jayakumar I (2005) Aggressive management of dengue shock syndrome may decrease mortality rate: a suggested protocol. Pediatr Crit Care Med 6: 412-419.

2147. Rao IS, Loya AC, Ratnakar K, Srinivasan V (2005) Lymph node infarction - a rare complication associated with disseminated intra vascular coagulation in a case of dengue fever. BMC Clin Pathol 5: 11.

2148. Ratageri VH, Shepur TA, Wari PK, Chavan SC, Mujahid IB, et al. (2005) Clinical profile and outcome of Dengue fever cases. Indian J Pediatr 72: 705-706.

2149. Rathavuth H, Vaughn DW, Minn K, Nimmannitya S, Nisalak A, et al. (1997) Hemorrhagic fever in Cambodia is caused by dengue viruses: evidence for transmission of all four serotypes. Southeast Asian J Trop Med Public Health 28: 120-125.

2150. Ratho RK, Mishra B, Kaur J, Kakkar N, Sharma K (2005) An outbreak of dengue fever in periurban slums of Chandigarh, India, with special reference to entomological and climatic factors. Indian J Med Sci 59: 518-526.

2151. Rathor HR (1996) The role of vectors in emerging and re-emerging diseases in the Eastern Mediterranean Region. East Mediterr Health J 2: 61-67.

2152. Rattan A (1997) DENGUE - INDIA (02). In: AIIMS, editor. New Delhi: PROMED.

2153. Rawlings J (1996) DENGUE - U.S./MEXICO BORDER, 1995-1996. In: MMWR C, editor. (US): PROMED.

2154. Rawlings J (1999) DENGUE - USA (TEXAS), MEXICO (COAHUILA). In: (IDEAS) IDEaS, editor. (Texas): PROMED.

2155. Rawlins SC, Martinez R, Wiltshire S, Clarke D, Prabhakar P, et al. (1997) Evaluation of Caribbean strains of Macrocyclops and Mesocyclops (Cyclopoida:Cyclopidae) as biological control tools for the dengue vector Aedes aegypti. J Am Mosq Control Assoc 13: 18-23.

2156. Rawlins SC, Martinez R, Wiltshire S, Legall G (1998) A comparison of surveillance systems for the dengue vector Aedes aegypti in Port of Spain, Trinidad. J Am Mosq Control Assoc 14: 131-136.

2157. Ray G, Kumar V, Kapoor AK, Dutta AK, Batra S (1999) Status of antioxidants and other biochemical abnormalities in children with dengue fever. J Trop Pediatr 45: 4-7.

2158. Razak MDA (1995) DENGUE - MALAYSIA. Kuala Lumpur: PROMED.

2159. Razak MDA (1995) DENGUE - MALAYSIA (PENANG). Penang, Malaysia: PROMED.

2160. Rebelo JM, Costa JM, Silva FS, Pereira YN, da Silva JM (1999) Distribuicao do Aedes aegypti e do dengue no Estado do Maranhao - Brasil. Cad Saude Publica 15: 477-486.

2161. Recker M, Blyuss KB, Simmons CP, Hien TT, Wills B, et al. (2009) Immunological serotype interactions and their effect on the epidemiological pattern of dengue. Proceedings of the Royal Society B-Biological Sciences 276: 2541-2548.

2162. Reed D, Maguire T, Mataika J (1977) Type 1 dengue with hemorrhagic disease in Fiji: epidemiologic findings. Am J Trop Med Hyg 26: 784-791.

2163. Regato M, Recarey R, Moratorio G, de Mora D, Garcia-Aguirre L, et al. (2008) Phylogenetic analysis of the NS5 gene of dengue viruses isolated in Ecuador. Virus Res 132: 197-200.

2164. Reiskind MH, Baisley KJ, Calampa C, Sharp TW, Watts DM, et al. (2001) Epidemiological and ecological characteristics of past dengue virus infection in Santa Clara, Peru. Trop Med Int Health 6: 212-218.

2165. Reiter P (1996) La dengue dans les Ameriques. Bull Soc Pathol Exot Filiales 89: 95-96; discussion 97.

2166. Ren RW, Fang MY, Hong WY, Huang BM, Jiang LH, et al. (2003) [Isolation, identification and sequence analyses of dengue virus type 2 strain GD19/2001]. Zhonghua Liu Xing Bing Xue Za Zhi 24: 288-290.

2167. Ren RW, Fang MY, Liu JW, Wang JJ, Hao L, et al. (2005) [Development of multiplex reverse translation-polymerase chain reaction methods for detection of dengue virus type 1-4 and its application in clinical use]. Zhonghua Liu Xing Bing Xue Za Zhi 26: 29-32.

2168. Renaud CJ, Manjit K, Pary S (2007) Dengue has a benign presentation in renal transplant patients: A case series. Nephrology 12: 305-307.

2169. Restrepo BN, Isaza DM, Salazar CL, Ramirez R, Ospina M, et al. (2008) Serum levels of interleukin-6, tumor necrosis factor-alpha and interferon-gamma in infants with and without dengue. Rev Soc Bras Med Trop 41: 6-10.

2170. Restrepo BN, Ramirez RE, Arboleda M, Alvarez G, Ospina M, et al. (2008) Serum Levels of Cytokines in Two Ethnic Groups with Dengue Virus Infection. Am J Trop Med Hyg 79: 673-677.

2171. Reyes E (2004) DENGUE/DHF UPDATE 2004 (21) [Ecuador/ Bangladesh/ Buthan]. In: ProMED-mail, editor: PROMED.

2172. Reyes P (2005) DENGUE/DHF UPDATE 2005 (12). PROMED.

2173. Reyes-dal Valle J, del Angel RM (2004) Isolation of putative dengue virus receptor molecules by affinity chromatography using a recombinant E protein ligand. J Virol Methods 116: 95-102.

2174. Reynes JM, Laurent A, Deubel V, Telliam E, Moreau JP (1994) The first epidemic of dengue hemorrhagic fever in French Guiana. Am J Trop Med Hyg 51: 545-553.

2175. Riaz MM, Mumtaz K, Khan MS, Patel J, Tariq M, et al. (2009) Outbreak of dengue fever in Karachi 2006: a clinical perspective. JPMA J Pak Med Assoc 59: 339-344.

2176. Ricardo GA (1997) DENGUE/DENGUE HEMORRHAGIC FEVER - CUBA (08). CubaPress, 11 July 1997 ed. Santiago de Cuba: PROMED.

2177. Richards AL, Bagus R, Baso SM, Follows GA, Tan R, et al. (1997) The first reported outbreak of dengue hemorrhagic fever in Irian Jaya, Indonesia. Am J Trop Med Hyg 57: 49-55.

2178. Richardson J, Molina-Cruz A, Salazar MI, Black W (2006) Quantitative analysis of dengue-2 virus RNA during the extrinsic incubation period in individual Aedes aegypti. Am J Trop Med Hyg 74: 132-141.

2179. Rico-Hesse R, Harrison LM, Nisalak A, Vaughn DW, Kalayanarooj S, et al. (1998) Molecular evolution of dengue type 2 virus in Thailand. Am J Trop Med Hyg 58: 96-101.

2180. Rico-Hesse R, Harrison LM, Salas RA, Tovar D, Nisalak A, et al. (1997) Origins of dengue type 2 viruses associated with increased pathogenicity in the Americas. Virology 230: 244-251.

2181. Rigau-Perez JG (1998) The early use of break-bone fever (Quebranta huesos, 1771) and dengue (1801) in Spanish. Am J Trop Med Hyg 59: 272-274.

2182. Rigau-Perez JG (1999) Surveillance for an emerging disease: dengue hemorrhagic fever in Puerto Rico, 1988-1997. Puerto Rico Association of Epidemiologists. P R Health Sci J 18: 337-345.

2183. Rigau-Perez JG (1999) Determining the incidence of dengue hemorrhagic fever in Puerto Rico. P R Health Sci J 18: 335.

2184. Rigau-Perez JG (2006) Severe dengue: the need for new case definitions. Lancet Infect Dis 6: 297-302.

2185. Rigau-Perez JG, Bonilla GL (1999) An evaluation of modified case definitions for the detection of dengue hemorrhagic fever. Puerto Rico Association of Epidemiologists. P R Health Sci J 18: 347-352.

2186. Rigau-Perez JG, Clark GG (1992) Dengue activity in Puerto Rico, 1990. P R Health Sci J 11: 65-68.

2187. Rigau-Perez JG, Clark GG, Gubler DJ, Reiter P, Sanders EJ, et al. (1998) Dengue and dengue haemorrhagic fever. Lancet 352: 971-977.

2188. Rigau-Perez JG, Gubler DJ, Vorndam AV, Clark GG (1994) Dengue surveillance--United States, 1986-1992. MMWR Surveill Summ 43: 7-19.

2189. Rigau-Perez JG, Laufer MK (2006) Dengue-related deaths in Puerto Rico, 1992-1996: diagnosis and clinical alarm signals. Clin Infect Dis 42: 1241-1246.

2190. Rigau-Perez JG, Millard PS, Walker DR, Deseda CC, Casta-Velez A (1999) A deviation bar chart for detecting dengue outbreaks in Puerto Rico. Am J Public Health 89: 374-378.

2191. Ritchie S (1997) DENGUE - AUSTRALIA (QUEENSLAND) (03). In: Programme SCH, Unit TPH, editors: PROMED.

2192. Ritchie SA, Long S, Smith G, Pyke A, Knox TB (2004) Entomological investigations in a focus of dengue transmission in Cairns, Queensland, Australia, by using the sticky ovitraps. J Med Entomol 41: 1-4.

2193. Rivetz B, Siman-Tov D, Ambal E, Jaramillo AC, Ben-Zvi A, et al. (2009) New dengue antibody assay with unique differential detection of IgG and IgM antibodies. Clin Biochem 42: 180-184.

2194. Robert V, Lhuillier M, Meunier D, Sarthou JL, Monteny N, et al. (1993) Virus amaril, dengue 2 et autres arbovirus isoles de moustiques, au Burkina Faso, de 1983 a 1986. Considerations entomologiques et epidemiologiques. Bulletin de la Société de pathologie exotique 86: 90-100.

2195. Roberts DR (1998) DENGUE - BRAZIL (MANAUS). In: Medicine/Biometrics DoP, editor. Division of Tropical Public Health. Bethesda: PROMED.

2196. Roca Y, Baronti C, Revollo RJ, Cook S, Loayza R, et al. (2009) Molecular Epidemiological Analysis of Dengue Fever in Bolivia from 1998 to 2008. Vector-Borne and Zoonotic Diseases 9: 337-344.

2197. Rocco IM, Barbosa ML, Kanomata EH (1998) Simultaneous infection with dengue 1 and 2 in a Brazilian patient. Rev Inst Med Trop Sao Paulo 40: 151-154.

2198. Rocco IM, Kavakama BB, Santos CL (2001) First isolation of dengue 3 in Brazil from an imported case. Rev Inst Med Trop Sao Paulo 43: 55-57.

2199. Rocha C, Morrison AC, Forshey BM, Blair PJ, Olson JG, et al. (2009) Comparison of Two Active Surveillance Programs for the Detection of Clinical Dengue Cases in Iquitos, Peru. Am J Trop Med Hyg 80: 656-660.

2200. Rocha C, Silva S, Gordon A, Hammond SN, Elizondo D, et al. (2009) Improvement in Hospital Indicators after Changes in Dengue Case Management in Nicaragua. Am J Trop Med Hyg 81: 287-292.

2201. Rocha LA, Tauil PL (2009) Dengue em crianca: aspectos clinicos e epidemiologicos, Manaus, Estado do Amazonas, no periodo de 2006 e 2007. Rev Soc Bras Med Trop 42: 18-22.

2202. Roche C, Cassar O, Laille M, Murgue B (2007) Dengue-3 virus genomic differences that correlate with in vitro phenotype on a human cell line but not with disease severity. Microbes Infect 9: 63-69.

2203. Rodhain F (1976) Resultats d'une enquete sur les vecteurs potentiels de la dengue dans l'archipel Neo-Caledonien. Bull Soc Pathol Exot Filiales 69: 21-27.

2204. Rodier GR, Gubler DJ, Cope SE, Cropp CB, Soliman AK, et al. (1996) Epidemic dengue 2 in the city of Djibouti 1991-1992. Trans R Soc Trop Med Hyg 90: 237-240.

2205. Rodrigo WWIS, Alcena DC, Kou Z, Kochel TJ, Porter KR, et al. (2009) Difference between the Abilities of Human Fc gamma Receptor-Expressing CV-1 Cells To Neutralize American and Asian Genotypes of Dengue Virus 2. Clin Vaccine Immunol 16: 285-287.

2206. Rodrigo WWSI, Alcena DC, Rose RC, Jin X, Schlesinger JJ (2009) Short Report: An Automated Dengue Virus Microneutralization Plaque Assay Performed in Human Fc gamma Reccptor-expressing CV-1 Cells. Am J Trop Med Hyg 80: 61-65.

2207. Rodrigues EM, Dal-Fabbro AL, Salomao R, Ferreira IB, Rocco IM, et al. (2002) Epidemiologia da infeccao pela dengue em Ribeirao Preto, SP, Brasil. Rev Saude Publica 36: 160-165.

2208. Rodrigues MB, Freire HB, Correa PR, Mendonca ML, Silva MR, et al. (2005) Is it possible to identify dengue in children on the basis of Ministry of Health criteria for suspected dengue cases? J Pediatr (Rio J) 81: 209-215.

2209. Rodriguez A (2004) DENGUE/DHF UPDATE 2004 (12) [Venezuela]. In: ProMED-mail, editor. El Salvador Ministry of Health, Epidemiological information - Dengue Reports, El Salvador - Up to Week 14, 10 Apr 2004 [in Spanish, translated by AR, edited] ed: PROMED.

2210. Rodriguez A (2004) DENGUE/DHF UPDATE 2004 (13) [Australia]. In: ProMED-mail, editor. ABC news, Australia 28 Apr 2004 [edited] ed: PROMED.

2211. Rodriguez A (2004) DENGUE/DHF UPDATE 2004 (15) [Venezuela/ Indonesia]. In: ProMED-mail, editor. El Universal, Venezuela 25 May 2004 [in Spanish, summarized by Mod.JW; edited] ed: PROMED.

2212. Rodriguez A (2004) DENGUE/DHF UPDATE 2004 (29) [China]. In: ProMED-mail, editor. Health & Community News, Hong Kong [edited] ed: PROMED.

2213. Rodriguez A (2005) DENGUE/DHF UPDATE 2005 (31). PROMED.

2214. Rodriguez A (2005) DENGUE/DHF UPDATE 2005 (36). PROMED.

2215. Rodriguez A (2005) DENGUE/DHF UPDATE 2005 (37). PROMED.

2216. Rodriguez A (2005) DENGUE/DHF UPDATE 2005 (38). PROMED.

2217. Rodriguez A (2005) DENGUE/DHF UPDATE 2005 (39). PROMED.

2218. Rodriguez A (2006) DENGUE/DHF UPDATE 2006 (01). PROMED.

2219. Rodriguez A (2006) DENGUE/DHF UPDATE 2006 (02). PROMED.

2220. Rodriguez A (2006) DENGUE/DHF UPDATE 2006 (03). PROMED.

2221. Rodriguez A (2006) DENGUE/DHF UPDATE 2006 (05). PROMED.

2222. Rodriguez A (2006) DENGUE/DHF UPDATE 2006 (06). PROMED.

2223. Rodriguez AJ (2004) DENGUE/DHF UPDATE 2005 (02). PROMED.

2224. Rodriguez AJ (2005) DENGUE/DHF UPDATE 2005 (03). PROMED.

2225. Rodriguez AJ (2005) DENGUE/DHF UPDATE 2005 (05). PROMED.

2226. Rodriguez AJ (2005) DENGUE/DHF UPDATE 2005 (06). PROMED.

2227. Rodriguez AJ (2005) DENGUE/DHF UPDATE 2005 (09). PROMED.

2228. Rodriguez AJ (2005) DENGUE/DHF UPDATE 2005 (12). PROMED.

2229. Rodriguez AJ (2005) DENGUE/DHF UPDATE 2005 (13). PROMED.

2230. Rodriguez AJ (2005) DENGUE/DHF UPDATE 2005 (14). PROMED.

2231. Rodriguez AJ (2005) DENGUE/DHF UPDATE 2005 (18). PROMED.

2232. Rodriguez AJ (2005) DENGUE/DHF UPDATE 2005 (19). PROMED.

2233. Rodriguez AJ (2006) DENGUE/DHF UPDATE 2006 (07). PROMED.

2234. Rodriguez AJ (2006) DENGUE/DHF UPDATE 2006 (08). PROMED.

2235. Rodriguez AJ (2006) DENGUE/DHF UPDATE 2006 (10). PROMED.

2236. Rodriguez AJ (2006) DENGUE/DHF UPDATE 2006 (11). PROMED.

2237. Rodriguez AJ (2006) DENGUE/DHF UPDATE 2006 (12). PROMED.

2238. Rodriguez AJ (2006) DENGUE/DHF UPDATE 2006 (13). PROMED.

2239. Rodriguez AJ (2006) DENGUE/DHF UPDATE 2006 (14). PROMED.

2240. Rodriguez AJ (2006) DENGUE/DHF UPDATE 2006 (17). PROMED.

2241. Rodriguez AJ, Banks A-L (2006) Dengue/DHF update 2006 (18). PROMED: Promed.

2242. Rodriguez AJ, Banks A-L (2006) Dengue/DHF update 2006 (26) PROMED: Promed.

2243. Rodriguez AJ, da Silva LJ, Yang RCA (2006) Dengue/DHF update 2006 (27) PROMED: Promed.

2244. Rodriguez Arias O, Perez Perez A, Despaigne Bicet A, Irarragirri Dorado CA (2001) Caracterizacion de pacientes con diagnostico presuntivo de dengue en el brote epidemico del ano 1997. Rev Cuba Med Trop 53: 24-27.

2245. Rodriguez DR, Rodriguez MG, Chavarria AM, Ramos-Jimenez J, Rivera MA, et al. (2009) Dengue virus antibodies in blood donors from an endemic area. Transfus Med 19: 125-131.

2246. Rodriguez H, de la Hoz F (2005) Dengue and dengue and vector behaviour in Caqueza, Colombia, 2004. Revista de Salud Pública 7: 1-15.

2247. Rodriguez-Roche R, Alvarez M, Gritsun T, Halstead S, Kouri G, et al. (2005) Virus evolution during a severe dengue epidemic in Cuba, 1997. Virology 334: 154-159.

2248. Rodriguez-Roche R, Alvarez M, Gritsun T, Rosario D, Halstead S, et al. (2005) Dengue virus type 2 in Cuba, 1997: conservation of E gene sequence in isolates obtained at different times during the epidemic. Arch Virol 150: 415-425.

2249. Rodriguez-Roche R AM, Holmes EC, Bernardo L, Kouri G, Gould EA, et al. (2005) Dengue virus type 3, Cuba, 2000–2002. Emerg Infect Dis 11: 773-774.

2250. Roehrig JT, Bolin RA, Kelly RG (1998) Monoclonal antibody mapping of the envelope glycoprotein of the dengue 2 virus, Jamaica. Virology 246: 317-328.

2251. Roehrig JT, Volpe KE, Squires J, Hunt AR, Davis BS, et al. (2004) Contribution of disulfide bridging to epitope expression of the dengue type 2 virus envelope glycoprotein. J Virol 78: 2648-2652.

2252. Roesel T (2005) DENGUE/DHF UPDATE 2005 (25). PROMED.

2253. Roesel T (2006) DENGUE/DHF UPDATE 2006 (20). PROMED: Promed.

2254. Roesel T, Milagres M, Moraes LM, A. R (2006) Dengue/DHF update 2006 (19) PROMED: Promed.

2255. Roesel T, Rodriguez A, Dudley J (2006) Dengue/DHF update 2006 (28) PROMED: Promed.

2256. Rojas EM, Diaz-Quijano FA, Coronel-Ruiz C, Martinez-Vega RA, Rueda E, et al. (2007) Correlación entre los niveles de glutatión peroxidasa, un marcador de estrés oxidativo, y la presentación clínica del dengue. Rev Med Chil 135: 743-750.

2257. Romero-Vivas CM, Leake CJ, Falconar AK (1998) Determination of dengue virus serotypes in individual Aedes aegypti mosquitoes in Colombia. Med Vet Entomol 12: 284-288.

2258. Rosado Leon R, Munoz Rodriguez MR, Soler Huerta E, Parissi Crivelli A, Mendez Machado GF (2007) Dengue durante el embarazo. Comunicacion de casos. Ginecol Obstet Mex 75: 687-690.

2259. Rosario D, Alvarez M, Diaz J, Contreras R, Rodriguez R, et al. (1998) Reaccion en cadena de la polimerasa para la deteccion rapida y determinacion del serotipo de virus del dengue en muestras clinicas. Rev Panam Salud Publica 4: 1-5.

2260. Rosen L (1986) Dengue in Greece in 1927 and 1928 and the pathogenesis of dengue hemorrhagic fever: new data and a different conclusion. Am J Trop Med Hyg 35: 642-653.

2261. Rosenbaum J, Nathan MB, Ragoonanansingh R, Rawlins S, Gayle C, et al. (1995) Community participation in dengue prevention and control: a survey of knowledge, attitudes, and practice in Trinidad and Tobago. Am J Trop Med Hyg 53: 111-117.

2262. Rossi CA, Drabick JJ, Gambel JM, Sun W, Lewis TE, et al. (1998) Laboratory diagnosis of acute dengue fever during the United Nations Mission in Haiti, 1995-1996. Am J Trop Med Hyg 59: 275-278.

2263. Row D, Weinstein P, Murray-Smith S (1996) Dengue fever with encephalopathy in Australia. Am J Trop Med Hyg 54: 253-255.

2264. Roy A, R Y, B B, Kc G (2007) Rebound thrombocytosis causing MI following dengue fever? Indian Heart J 59: 94.

2265. Ruangturakit S, Rojanasuphot S, Srijuggravanvong A, Duangchanda S, Nuangplee S, et al. (1994) Storage stability of dengue IgM and IgG antibodies in whole blood and serum dried on filter paper strips detected by ELISA. Southeast Asian J Trop Med Public Health 25: 560-564.

2266. Runge-Ranzinger S, Horstick O, Marx M, Kroeger A (2008) What does dengue disease surveillance contribute to predicting and detecting outbreaks and describing trends? Trop Med Int Health 13: 1022-1041.

2267. Rurayantsev AA, Chanock RM, Murphy BR, Pletnev AG (2006) Comparison of live and inactivated tick-borne encephalitis virus vaccines for safety, immunogenicity and efficacy in rhesus monkeys. Vaccine 24: 133-143.

2268. Russell BM, McBride WJ, Mullner H, Kay BH (2002) Epidemiological significance of subterranean Aedes aegypti (Diptera: Culicidae) breeding sites to dengue virus infection in Charters Towers, 1993. J Med Entomol 39: 143-145.

2269. Russell PK, Buescher EL, McCown JM, Ordonez J (1966) Recovery of dengue viruses from patients during epidemics in Puerto Rico and East Pakistan. Am J Trop Med Hyg 15: 573-579.

2270. Russell RC (2009) Mosquito-borne disease and climate change in Australia: time for a reality check. Aust J Entomol 48: 1-7.

2271. Russell RC, Webb CE, Davies N (2005) Aedes aegypti (L.) and Aedes polynesiensis marks (Diptera : culicidae) in Moorea, French Polynesia: A study of adult population structures and pathogen (Wuchereria bancrofti and Dirofilaria immitis) infection rates to indicate regional and seasonal epidemiological risk for dengue and filariasis. J Med Entomol 42: 1045-1056.

2272. Rymzo WT, Jr., Cline BL, Kemp GE, Sather GE, Craven PC (1976) Dengue outbreaks in Guanica-Ensenada and Villalba, Puerto Rico, 1972-1973. Am J Trop Med Hyg 25: 136-145.

2273. Saadiah S, Sharifah BI, Robson A, Greaves MW (2008) Skin histopathology and immunopathology are not of prognostic value in dengue haemorrhagic fever. Br J Dermatol 158: 836-837.

2274. Saavedra QJ (2003) DENGUE/DHF - BOLIVIA (SANTA CRUZ):SUSPECTED. In: PAHO, editor. dengue reports to the Pan American Health Organization (PAHO) during the period 1995 through 2003 ed: PROMED.

2275. Sabchareon A, Lang J, Chanthavanich P, Yoksan S, Forrat R, et al. (2004) Safety and immunogenicity of a three dose regimen of two tetravalent live-attenuated dengue vaccines in five- to twelve-year-old Thai children. Pediatr Infect Dis J 23: 99-109.

2276. Sabchareon A, Lang J, Chanthavanich P, Yoksan S, Forrat R, et al. (2002) Safety and immunogenicity of tetravalent live-attenuated dengue vaccines in Thai adult volunteers: role of serotype concentration, ratio, and multiple doses. Am J Trop Med Hyg 66: 264-272.

2277. Sadon N, Delers A, Jarman RG, Klungthong C, Nisalak A, et al. (2008) A new quantitative RT-PCR method for sensitive detection of dengue virus in serum samples. J Virol Methods 153: 1-6.

2278. Saenz Abad D, Ruiz-Ruiz FJ, Monzon Ballarin S, Mora Alvaro F (2007) Fiebre y exantema tras viaje a Brasil. Rev Clin Esp 207: 369-370.

2279. Sagsveen M (2009) Okning av denguefeber i India. Tidsskrift for den Norske lægeforening 129: 1130.

2280. Sai PMV, Dev B, Krishnan R (2005) Role of ultrasound in dengue fever. Br J Radiol 78: 416-418.

2281. Saito M, Oishi K, Inoue S, Dimaano EM, Alera MTP, et al. (2004) Association of increased platelet-associated immunoglobulins with thrombocytopenia and the severity of disease in secondary dengue virus infections. Clin Exp Immunol 138: 299-303.

2282. Sakoonwatanyoo P, Boonsanay V, Smith DR (2006) Growth and production of the dengue virus in C6/36 cells and identification of a laminin-binding protein as a candidate serotype 3 and 4 receptor protein. Intervirology 49: 161-172.

2283. Sakuntabhai A, Turbpaiboon C, Casademont I, Chuansumrit A, Lowhnoo T, et al. (2005) A variant in the CD209 promoter is associated with severity of dengue disease. Nat Genet 37: 507-513.

2284. Salas RA, Tovar D, Barreto A, de Miller E, Leitmeyer K, et al. (1998) Serotipos y genotipos de virus dengue circulantes en Venezuela, 1990-1997. Acta Cient Venez 49 Suppl 1: 33-37.

2285. Salazar-Botero R (1997) DENGUE - COLOMBIA (03). In: Malaria] SSA, editor. Source: El Tiempo, Oct. 25, 1997 ed. Cali: PROMED.

2286. Salazar-Botero R (1998) DENGUE/DHF - COLOMBIA (CALI). In: Malaria] SSA, editor. EL TIEMPO, WEDNESDAY JAN 21, 1998 ed. Cali: PROMED.

2287. Salda LTD, Parquet MDC, Matias RR, Natividad FF, Kobayashi N, et al. (2005) Molecular epidemiology of Dengue 2 viruses in the Philippines: Genotype shift and local evolution. Am J Trop Med Hyg 73: 796-802.

2288. Saleem K, Shaikh I (2008) Skin Lesions in Hospitalized Cases of Dengue Fever. Jcpsp-Journal of the College of Physicians and Surgeons Pakistan 18: 608-611.

2289. Sales FM (2008) Acoes de educacao em saude para prevencao e controle da dengue: um estudo em Icarai, Caucaia, Ceara. Ciência & Saúde Coletiva 13: 175-184.

2290. Salgado DM, Rodriguez JA, Garzon M, Cifuentes G, Ibarra M, et al. (2007) Caracterizacion Clinica y Epidemiologica de Dengue Hemorragico en Neiva, Colombia, 2004. Revista de Salud Pública 9: 53-63.

2291. Saluzzo JF, Cornet M, Castagnet P, Rey C, Digoutte JP (1986) Isolation of dengue 2 and dengue 4 viruses from patients in Senegal. Trans R Soc Trop Med Hyg 80: 5.

2292. Sampaio AM, Kligerman DC, Junior SF (2009) Dengue, related to rubble and building construction in Brazil. Waste Manag.

2293. Samsi TK, Wulur H, Sugianto D, Bartz CR, Tan R, et al. (1990) Some clinical and epidemiological observations on virologically confirmed dengue hemorrhagic fever. Paediatr Indones 30: 293-303.

2294. Samuel PP, Thenmozhi V, Tyagi BK (2007) A focal outbreak of dengue fever in a rural area of Tamil Nadu. Indian J Med Res 125: 179-181.

2295. Sanchez V, Hessler C, DeMonfort A, Lang J, Guy B (2006) Comparison by flow cytometry of immune changes induced in human monocyte-derived dendritic cells upon infection with dengue 2 live-attenuated vaccine or 16681 parental strain. FEMS Immunol Med Microbiol 46: 113-123.

2296. Sanchez-Burgos G, Hernandez-Pando R, Campbell IL, Ramos-Castaneda J, Ramos C (2004) Cytokine production in brain of mice experimentally infected with dengue virus. Neuroreport 15: 37-42.

2297. Sanchez-Seco MP, Rosario D, Hernandez L, Domingo C, Valdes K, et al. (2006) Detection and subtyping of dengue 1-4 and yellow fever viruses by means of a multiplex RT-nested-PCR using degenerated primers. Trop Med Int Health 11: 1432-1441.

2298. Sanchez-Vargas I, Travanty EA, Keene KM, Franz AWE, Beaty BJ, et al. (2004) RNA interference, arthropod-borne viruses, and mosquitoes. Virus Res 102: 65-74.

2299. Sanders EJ, Rigau-Perez JG, Smits HL, Deseda CC, Vorndam VA, et al. (1999) Increase of leptospirosis in dengue-negative patients after a hurricane in Puerto Rico in 1996 [correction of 1966]. Am J Trop Med Hyg 61: 399-404.

2300. Sa-Ngasang A, Anantapreecha S, A AN, Chanama S, Wibulwattanakij S, et al. (2005) Specific IgM and IgG responses in primary and secondary dengue virus infections determined by enzyme-linked immunosorbent assay. Epidemiol Infect: 1-6.

2301. Sa-Ngasang A, Anantapreecha S, A-Nuegoonpipat A, Chanama S, Wibulwattanakij S, et al. (2006) Specific IgM and IgG responses in primary and secondary dengue virus infections determined by enzyme-linked immunosorbent assay. Epidemiol Infect 134: 820-825.

2302. Sa-ngasang A, Wibulwattanakij S, Chanama S, O-rapinpatipat A, A-nuegoonpipat A, et al. (2003) Evaluation of RT-PCR as a tool for diagnosis of secondary dengue virus infection. Jpn J Infect Dis 56: 205-209.

2303. Sangkawibha N, Rojanasuphot S, Ahandrik S, Viriyapongse S, Jatanasen S, et al. (1984) Risk factors in dengue shock syndrome: a prospective epidemiologic study in Rayong, Thailand. I. The 1980 outbreak. Am J Epidemiol 120: 653-669.

2304. Sanjay S, Au Eong KG (2007) Bilateral vitreous haemorrhage associated with dengue fever. Eye 21: 144-145.

2305. Sanjay S, Wagle AM, Au Eong KG (2008) Optic neuropathy associated with dengue fever. Eye 22: 722-724.

2306. Santiago A, Fernandez-Sein A (2001) Dengue in children: critical points in management. P R Health Sci J 20: 343-346.

2307. Santos CL, Sallum MA, Foster PG, Rocco IM (2004) Molecular analysis of the dengue virus type 1 and 2 in Brazil based on sequences of the genomic envelope-nonstructural protein 1 junction region. Rev Inst Med Trop Sao Paulo 46: 145-152.

2308. Santos NQ, Azoubel AC, Lopes AA, Costa G, Bacellar A (2004) Guillain-Barre syndrome in the course of dengue: case report. Arq Neuropsiquiatr 62: 144-146.

2309. Sariol CA, Pelegrino JL, Martinez A, Arteaga E, Kouri G, et al. (1999) Detection and genetic relationship of dengue virus sequences in seventeen-year-old paraffin-embedded samples from Cuba. Am J Trop Med Hyg 61: 994-1000.

2310. Sasaki DM (1998) DENGUE (POSSIBLE) - HAWAII (04). In: Health HDo, editor. (Hawaii): PROMED.

2311. Sathish N, Manayani DJ, Shankar V, Abraham M, Nithyanandam G, et al. (2002) Comparison of IgM capture ELISA with a commercial rapid immunochromatographic card test & IgM microwell ELISA for the detection of antibodies to dengue viruses. Indian J Med Res 115: 31-36.

2312. Sathupan P, Khongphattanayothin A, Srisai J, Srikaew K, Poovorawan Y (2007) The role of vascular endothelial growth factor leading to vascular leakage in children with dengue virus infection. Ann Trop Paediatr 27: 179-184.

2313. Saunders D (1997) DENGUE - FIJI. In: Fiji M, editor. (Fiji): PROMED.

2314. Saunders D (1998) DENGUE - FIJI (18). (Fiji): PROMED.

2315. Saunders D (1998) DENGUE - FIJI (19). Suva, Fiji: PROMED.

2316. Saunders D (1998) DENGUE - FIJI (11). In: Network PPHS, editor. Fiji: PROMED.

2317. Saunders D (1998) DENGUE - FIJI (15). In: Ministry of Health F, editor. (Fiji): PROMED.

2318. Saunders D (1998) DENGUE - FIJI (10). In: Ministry of Health F, editor. (Fiji): PROMED.

2319. Saunders D (1998) DENGUE - FIJI (05). In: Ministry of Health F, editor. (Fiji): PROMED.

2320. Saunders D (1998) DENGUE - FIJI (07). In: Ministry of Health F, editor. (Fiji): PROMED.

2321. Saunders D (1998) DENGUE - FIJI (09). In: Ministry of Health F, editor. (Fiji): PROMED.

2322. Saunders D (1998) DENGUE - FIJI (17). In: Ministry of Health F, editor. (Fiji): PROMED.

2323. Saunders D (1998) DENGUE - FIJI (16). In: Ministry of Health F, editor. (Fiji): PROMED.

2324. Saunders D (1998) DENGUE - FIJI (20). In: PACNET@LISTSERV.SPC.ORG.NC, editor: PROMED.

2325. Savage HM, Fritz CL, Rutstein D, Yolwa A, Vorndam V, et al. (1998) Epidemic of dengue-4 virus in Yap State, Federated States of Micronesia, and implication of Aedes hensilli as an epidemic vector. Am J Trop Med Hyg 58: 519-524.

2326. Saxena M (1996) DENGUE/DHF - INDIA (5). New Delhi: PROMED.

2327. Saxena P, Dash PK, Santhosh SR, Shrivastava A, Parida M, et al. (2008) Development and evaluation of one step single tube multiplex RT-PCR for rapid detection and typing of dengue viruses. Virol J 5: 20.

2328. Scat Y, Moreau O, Fougere V (1989) Interet d'une methode d'immunocapture pour la recherche des IgM 6eriques dans la surveillance de la dengue a la Martinique. Bull Soc Pathol Exot Filiales 82: 173-184.

2329. Schatzmayr HG (2000) Dengue situation in Brazil by year 2000. Mem Inst Oswaldo Cruz 95 Suppl 1: 179-181.

2330. Schatzmayr HG, Nogueira RM, Travassos da Rosa AP (1986) An outbreak of dengue virus at Rio de Janeiro--1986. Mem Inst Oswaldo Cruz 81: 245-246.

2331. Schilling S, Ludolfs D, Van An L, Schmitz H (2004) Laboratory diagnosis of primary and secondary dengue infection. J Clin Virol 31: 179-184.

2332. Schioler KL, Macpherson CN (2009) Dengue Transmission in the Small-Island Setting: Investigations from the Caribbean Island of Grenada. Am J Trop Med Hyg 81: 280-286.

2333. Schleupner CJ (1977) Dengue fever in a Western United States City. West J Med 127: 145-149.

2334. Schreiber KV (2001) An investigation of relationships between climate and dengue using a water budgeting technique. Int J Biometeorol 45: 81-89.

2335. Schreiber MJ, Holmes EC, Ong SH, Soh HSH, Liu W, et al. (2009) Genomic Epidemiology of a Dengue Virus Epidemic in Urban Singapore. J Virol 83: 4163-4173.

2336. Schultz GW (1989) Cemetery vase breeding of dengue vectors in Manila, Republic of the Philippines. J Am Mosq Control Assoc 5: 508-513.

2337. Schultz GW (1993) Seasonal abundance of dengue vectors in Manila, Republic of the Philippines. Southeast Asian J Trop Med Public Health 24: 369-375.

2338. Schultze D, Berendonk C, Ammann T, Niedrig M (2002) Isolation of dengue virus serotype 1 from the blood of a Swiss traveler prior to seroconversion. Infection 30: 237-239.

2339. Schwartz E, Mendelson E, Sidi Y (1996) Dengue fever among travelers. Am J Med 101: 516-520.

2340. Schwartz E, Moskovitz A, Potasman I, Peri G, Grossman Z, et al. (2000) Changing epidemiology of dengue fever in travelers to Thailand. Eur J Clin Microbiol Infect Dis 19: 784-786.

2341. Schwartz E, Weld LH, Wilder-Smith A, von Sonnenburg F, Keystone JS, et al. (2008) Seasonality, annual trends, and characteristics of dengue among ill returned travelers, 1997-2006. Emerg Infect Dis 14: 1081-1088.

2342. Schwarz TF, Jager G, Gilch S (1995) Imported dengue virus infections in German tourists. Zentralblatt fur Bakteriologie 282: 533-536.

2343. Scott B (2007) Dengue. Lancet 370: 1644-1652.

2344. Scott R (1998) DENGUE - AUSTRALIA (QUEENSLAND) (10). In: ProMED-mail, editor. Cairns: PROMED.

2345. Seed CR, Kiely P, Hyland CA, Keller AJ (2009) The risk of dengue transmission by blood during a 2004 outbreak in Cairns, Australia. Transfusion (Paris) 49: 1482-1487.

2346. Seeney H (2003) DENGUE/DHF UPDATE 2003 (48) [Australia/ India/ Sri Lanka]. In: ProMED-mail, editor. Townsville Bulletin, Australia 10 Dec 2003 [edited] ed: PROMED.

2347. Seet RC, Chow AW, Quek AM, Chan YH, Lim EC (2009) Relationship between circulating vascular endothelial growth factor and its soluble receptors in adults with dengue virus infection: a case-control study. Int J Infect Dis 13: e248-253.

2348. Seet RCS, Lee CYJ, Lim ECH, Quek AML, Yeo LLL, et al. (2009) Oxidative damage in dengue fever. Free Radic Biol Med 47: 375-380.

2349. Seet RCS, Lim ECH, Wilder-Smith EPV (2006) Acute transverse myelitis following Dengue virus infection. J Clin Virol 35: 310-312.

2350. Seet RCS, Ooi EE, Wong HB, Paton NI (2005) An outbreak of primary dengue infection among migrant Chinese workers in Singapore characterized by prominent gastrointestinal symptoms and a high proportion of symptomatic cases. J Clin Virol 33: 336-340.

2351. Seet RCS, Quek AML, Lim ECH (2007) Symptoms and risk factors of ocular complications following dengue infection. J Clin Virol 38: 101-105.

2352. Seet RCS, Quek AML, Lim ECH (2007) Post-infectious fatigue syndrome in dengue infection. J Clin Virol 38: 1-6.

2353. Seijo A, Cernigoi B, Deodato B (2001) Dengue importado del Paraguay a Buenos Aires. Estudio clinico y epidemiologico de 38 casos. Medicina (Mex) 61: 137-141.

2354. Sekaran SD, Lan EC, Subramaniam G (2008) Comparison of five serological diagnostic assays for the detection of IgM and IgG antibodies to dengue virus. African Journal of Microbiology Research 2: 141-147.

2355. Sellahewa KH (2008) Dengue fever--predictors of disease severity and their influence on management. Ceylon Med J 53: 75-78.

2356. Sellahewa KH, Samaraweera N, Thusita KP, Fernando JL (2008) Is fresh frozen plasma effective for thrombocytopenia in adults with dengue fever? A prospective randomised double blind controlled study. Ceylon Med J 53: 36-40.

2357. Sellors M, Schmidt T (2006) Dengue/DHF update 2006 (36) PROMED: Promed.

2358. Seneviratne SL, Malavige GN, de Silva HJ (2006) Pathogenesis of liver involvement during dengue viral infections. Trans R Soc Trop Med Hyg.

2359. Seravali MR, Santos AH, Costa CE, Rangel DT, Valentim LF, et al. (2008) Spontaneous splenic rupture due to dengue fever: report of two cases. Braz J Infect Dis 12: 538-540.

2360. Serufo JC, Souza AM, Tavares VA, Jammal MC, Silva JG (1993) Dengue in the south-eastern region of Brazil: historical analysis and epidemiology. Rev Saude Publica 27: 157-167.

2361. Setbon M, Raude J (2008) Perceptions et comportements de prevention face a la dengue en Martinique. Medecine Et Maladies Infectieuses 38 Suppl 2: S78-81.

2362. Setiati TE, Mairuhu ATA, Koraka P, Supriatna M, Mac Gillavry MR, et al. (2007) Dengue disease severity in Indonesian children: an evaluation of the World Health Organization classification system. BMC Infect Dis 7: -.

2363. Setlik RF, Ouellette D, Morgan J, McAllister CK, Dorsey D, et al. (2004) Pulmonary hemorrhage syndrome associated with an autochthonous case of dengue hemorrhagic fever. South Med J 97: 688-691.

2364. Setrkraising K, Bongsebandhu-phubhakdi C, Voraphani N, Pancharoen C, Thisyakorn U, et al. (2007) D-dimer as an indicator of dengue severity. Asian Biomedicine 1: 53-57.

2365. Shah GS, Islam S, Das BK (2006) Clinical and laboratory profile of dengue infection in children. Kathmandu Univ Med J 4: 40-43.

2366. Shah I (2008) Dengue and liver disease. Scand J Infect Dis 40: 993-994.

2367. Shah I, Deshpande GC, Tardeja PN (2004) Outbreak of dengue in Mumbai and predictive markers for dengue shock syndrome. J Trop Pediatr 50: 301-305.

2368. Sharma A, Mahajan S, Gupta ML, Kanga A, Sharma V (2005) Investigation of an outbreak of scrub typhus in the Himalayan region of India. Jpn J Infect Dis 58: 208-210.

2369. Sharma JB, Gulati N (1992) Potential relationship between dengue fever and neural tube defects in a northern district of India. Int J Gynaecol Obstet 39: 291-295.

2370. Sharma N, Mahi S, Bhalla A, Singh V, Varma S, et al. (2006) Dengue fever related acalculous cholecystitis in a North Indian tertiary care hospital. J Gastroenterol Hepatol 21: 664-667.

2371. Sharma SK, Gupta BS, Devpura G, Agarwal A, Anand S (2007) Pulmonary haemorrhage syndrome associated with dengue haemorrhagic fever. J Assoc Physicians India 55: 729-730.

2372. Sharma SN, Raina VK, Kumar A (2000) Dengue/DHF: an emerging disease in India. J Commun Dis 32: 175-179.

2373. Sharp TW, Wallace MR, Hayes CG, Sanchez JL, DeFraites RF, et al. (1995) Dengue fever in U.S. troops during Operation Restore Hope, Somalia, 1992-1993. Am J Trop Med Hyg 53: 89-94.

2374. Shaw MTM, Leggat PA, Weld LH, Williams ML, Cetron MS (2003) Illness in returned travellers presenting at GeoSentinel sites in New Zealand. Aust N Z J Public Health 27: 82-86.

2375. Shekhar KC, Huat OL (1992) Epidemiology of dengue/dengue hemorrhagic fever in Malaysia--a retrospective epidemiological study 1973-1987. Part I: Dengue hemorrhagic fever (DHF). Asia Pac J Public Health 6: 15-25.

2376. Shekhar KC, Huat OL (1992) Epidemiology of dengue/dengue hemorrhagic fever in Malaysia--a retrospective epidemiological study. 1973-1987. Part II: Dengue fever (DF). Asia Pac J Public Health 6: 126-133.

2377. Shekhar KC, Senan P (1992) Epidemiology of dengue and dengue haemorrhagic fever in Malaysia. III. A comparative study of clinical features seen in virologically confirmed cases for periods between 1963-1987--a review. J Singapore Paediatr Soc 34: 67-82.

2378. Shirtcliffe P, Cameron E, Nicholson KG, Wiselka MJ (1998) Don't forget dengue! Clinical features of dengue fever in returning travellers. J R Coll Physicians Lond 32: 235-237.

2379. Shivbalan S, Anandnathan K, Balasubramanian S, Datta M, Amalraj E (2004) Predictors of spontaneous bleeding in Dengue. Indian J Pediatr 71: 33-36.

2380. Shresta S, Kyle JL, Snider HM, Basavapatna M, Beatty PR, et al. (2004) Interferon-dependent immunity is essential for resistance to primary dengue virus infection in mice, Whereas T- and B-cell-dependent immunity are less critical. J Virol 78: 2701-2710.

2381. Shresta S, Sharar KL, Prigozhin DM, Beatty PR, Harris E (2006) Murine model for dengue virus-induced lethal disease with increased vascular permeability. J Virol 80: 10208-10217.

2382. Shresta S, Sharar KL, Prigozhin DM, Snider HM, Beatty PR, et al. (2005) Critical roles for both STAT1-dependent and STAT1-independent pathways in the control of primary dengue virus infection in mice. J Immunol 175: 3946-3954.

2383. Shrivastava R, Upreti RK, Chaturvedi UC (2005) Effects of dengue virus infection on the spleen of male mice given hexavalent chromium with drinking water. Toxicol Mech Methods 15: 323-329.

2384. Shu LP, Zuo L, Zhao X, Chen AY, Wei LH (2004) [Susceptibility of 15 collections of Aedes albopictus from Guizhou to dengue virus oral infection]. Zhonghua Shi Yan He Lin Chuang Bing Du Xue Za Zhi 18: 234-237.

2385. Shu PY, Chen LK, Chang SF, Su CL, Chien LJ, et al. (2004) Dengue virus serotyping based on envelope and membrane and nonstructural protein NS1 serotype-specific capture immunoglobulin M enzyme-linked immunosorbent assays. J Clin Microbiol 42: 2489-2494.

2386. Shu PY, Chen LK, Chang SF, Yueh YY, Chow L, et al. (2002) Potential application of nonstructural protein NS1 serotype-specific immunoglobulin G enzyme-linked immunosorbent assay in the seroepidemiologic study of dengue virus infection: correlation of results with those of the plaque reduction neutralization test. J Clin Microbiol 40: 1840-1844.

2387. Shu PY, Chien LJ, Chang SF, Su CL, Kuo YC, et al. (2005) Fever screening at airports and imported dengue. Emerg Infect Dis 11: 460-462.

2388. Shu PY, Su CL, Liao TL, Yang CF, Chang SF, et al. (2009) Molecular Characterization of Dengue Viruses Imported Into Taiwan during 2003-2007: Geographic Distribution and Genotype Shift. Am J Trop Med Hyg 80: 1039-1046.

2389. Shu PY, Yang CF, Kao JF, Su CL, Chang SF, et al. (2009) Application of the Dengue Virus NS1 Antigen Rapid Test for On-Site Detection of Imported Dengue Cases at Airports. Clin Vaccine Immunol 16: 589-591.

2390. Sideridis K, Canario D, Cunha BA (2003) Dengue fever: diagnostic importance of a camelback fever pattern. Heart Lung 32: 414-418.

2391. Sierra B, Alegre R, Perez AB, Garcia G, Sturn-Ramirez K, et al. (2007) HLA-A, -B, -C, and -DRB1 allele frequencies in Cuban individuals with antecedents of dengue 2 disease: Advantages of the Cuban population for HLA studies of dengue virus infection. Hum Immunol 68: 531-540.

2392. Sierra B, Garcia G, Perez AB, Morier L, Rodriguez R, et al. (2002) Long-term memory cellular immune response to dengue virus after a natural primary infection. Int J Infect Dis 6: 125-128.

2393. Sierra BD, Kouri G, Guzman MG (2008) Race: A Risk Factor for Dengue Hemorrhagic Fever. Arch Virol 10: 533-542.

2394. Sierra BDC, Garcia G, Perez AB, Morier L, Alvarez M, et al. (2006) Ethnicity and difference in dengue virus-specific memory T cell responses in Cuban individuals. Viral Immunol 19: 662-668.

2395. Sierra BDL, Kouri G, Guzman MG (2007) Race: a risk factor for dengue hemorrhagic fever. Arch Virol 152: 533-542.

2396. Silarug N, Foy HM, Kupradinon S, Rojanasuphot S, Nisalak A, et al. (1990) Epidemic of fever of unknown origin in rural Thailand, caused by influenza A (H1N1) and dengue fever. Southeast Asian J Trop Med Public Health 21: 61-67.

2397. Silva RL, de Silva AM, Harris E, MacDonald GH (2008) Genetic analysis of Dengue 3 virus subtype III 5' and 3' non-coding regions. Virus Res 135: 320-325.

2398. Simasathien S, Thomas SJ, Watanaveeradej V, Nisalak A, Barberousse C, et al. (2008) Safety and immunogenicity of a tetravalent live-attenuated dengue vaccine in flavivirus naive children. Am J Trop Med Hyg 78: 426-433.

2399. Simmons CP, Chau TN, Thuy TT, Tuan NM, Hoang DM, et al. (2007) Maternal antibody and viral factors in the pathogenesis of dengue virus in infants. J Infect Dis 196: 416-424.

2400. Simmons CP, Dong T, Chau NV, Dung NTP, Chau TNB, et al. (2005) Early T-cell responses to dengue virus epitopes in Vietnamese adults with secondary dengue virus infections. J Virol 79: 5665-5675.

2401. Simmons CP, Popper S, Dolocek C, Chau TN, Griffiths M, et al. (2007) Patterns of host genome-wide gene transcript abundance in the peripheral blood of patients with acute dengue hemorrhagic fever. J Infect Dis 195: 1097-1107.

2402. Singfield P (1997) DENGUE - BELIZE (02). the "Reporter" newspaper, Belize ed. Belize: PROMED.

2403. Singh J, Balakrishnan N, Bhardwaj M, Amuthadevi P, George EG, et al. (2000) Silent spread of dengue and dengue haemorrhagic fever to Coimbatore and Erode districts in Tamil Nadu, India, 1998: need for effective surveillance to monitor and control the disease. Epidemiol Infect 125: 195-200.

2404. Singh K, Lale A, Eong Ooi E, Chiu LL, Chow VT, et al. (2006) A prospective clinical study on the use of reverse transcription-polymerase chain reaction for the early diagnosis of Dengue fever. J Mol Diagn 8: 613-616.

2405. Singh MM, Rahi M, Ahuja C, Garg S, Gupta VK, et al. (2007) Knowledge about Dengue fever and mosquito control practices in an urban slum of Delhi. J Commun Dis 39: 185-187.

2406. Singh N, Sharma KA, Dadhwal V, Mittal S, Selvi AS (2008) A successful management of dengue fever in pregnancy: Report of two cases. Indian J Med Microbiol 26: 377-U377.

2407. Singh NP, Jhamb R, Agarwal SK, Gaiha M, Dewan R, et al. (2005) The 2003 outbreak of Dengue fever in Delhi, India. Southeast Asian J Trop Med Public Health 36: 1174-1178.

2408. Singh S, Jat KR, Suri D, Ratho RK (2009) Dengue fever and Kawasaki disease: a clinical dilemma. Rheumatol Int 29: 717-719.

2409. Singh UB, Maitra A, Broor S, Rai A, Pasha ST, et al. (1999) Partial nucleotide sequencing and molecular evolution of epidemic causing Dengue 2 strains. J Infect Dis 180: 959-965.

2410. Singh UB, Seth P (2001) Use of nucleotide sequencing of the genomic cDNA fragments of the capsid/premembrane junction region for molecular epidemiology of dengue type 2 viruses. Southeast Asian J Trop Med Public Health 32: 326-335.

2411. Singhi S, Jayashree M (2007) Dengue shock syndrome: At the heart of the issue. Pediatr Crit Care Med 8: 583-584.

2412. Sinha N, Gupta N, Jhamb R, Gulati S, Kulkarni Ajit V (2008) The 2006 dengue outbreak in Delhi, India. J Commun Dis 40: 243-248.

2413. Siong WC, Ching TH, Jong GC, Pang CS, Vernon LJ, et al. (2008) Dengue infections in HIV patients. Southeast Asian J Trop Med Public Health 39: 260-265.

2414. Siqueira JB, Martelli CM, Maciel IJ, Oliveira RM, Ribeiro MG, et al. (2004) Household survey of dengue infection in central Brazil: spatial point pattern analysis and risk factors assessment. Am J Trop Med Hyg 71: 646-651.

2415. Siqueira JB, Martelli CMT, Coelho GE, Simplicio ACD, Hatch DL (2005) Dengue and dengue hemorrhagic fever, Brazil, 1981-2002. Emerg Infect Dis 11: 48-53.

2416. Siqueira RC, Vitral NP, Campos WR, Orefice F, Figueiredo LTM (2004) Ocular manifestations in Dengue fever. Ocul Immunol Inflamm 12: 323-327.

2417. Sirinavin S, Nuntnarumit P, Supapannachart S, Boonkasidecha S, Techasaensiri C, et al. (2004) Vertical dengue infection: case reports and review. Pediatr Infect Dis J 23: 1042-1047.

2418. Sistayanarain A, Maneekarn N, Polprasert B, Sirisanthana V, Makino Y, et al. (1996) Primary sequence of the envelope glycoprotein of a dengue type 2 virus isolated from patient with dengue hemorrhagic fever and encephalopathy. Southeast Asian J Trop Med Public Health 27: 221-227.

2419. Sithiprasasna R, Patpoparn S, Attatippaholkun W, Suvannadabba S, Srisuphanunt M (2004) The geographic information system as an epidemiological tool in the surveillance of dengue virus-infected Aedes mosquitos. Southeast Asian J Trop Med Public Health 35: 918-926.

2420. Sittisombut N, Sistayanarain A, Cardosa MJ, Salminen M, Damrongdachakul S, et al. (1997) Possible occurrence of a genetic bottleneck in dengue serotype 2 viruses between the 1980 and 1987 epidemic seasons in Bangkok, Thailand. Am J Trop Med Hyg 57: 100-108.

2421. Smith CE, Tom T, Sasaki J, Ayers T, Effler PV (2005) Dengue risk among visitors to Hawaii during an outbreak. Emerg Infect Dis 11: 750-756.

2422. Soares CN, Cabral-Castro M, Oliveira C, Faria LC, Peralta JM, et al. (2008) Oligosymptomatic dengue infection: a potential cause of Guillain Barre syndrome. Arq Neuropsiquiatr 66: 234-237.

2423. Soares CN, Cabral-Castro MJ, Peralta JM, Freitas MR, Puccioni-Sohler M (2009) Meningitis determined by oligosymptomatic dengue virus type 3 infection: Report of a case. Int J Infect Dis.

2424. Sohan L, Shyamal B, Kumar TS, Malini M, Ravi K, et al. (2008) Studies on leptospirosis outbreaks in Peddamandem Mandal of Chittoor district, Andhra Pradesh. J Commun Dis 40: 127-132.

2425. Solomon T, Dung NM, Vaughn DW, Kneen R, Thao LT, et al. (2000) Neurological manifestations of dengue infection. Lancet 355: 1053-1059.

2426. Solomon T, Mallewa M (2001) Dengue and other emerging flaviviruses. J Infect 42: 104-115.

2427. Songco RS, Hayes CG, Leus CD, Manaloto CO (1987) Dengue fever/dengue haemorrhagic fever in Filipino children: clinical experience during the 1983-1984 epidemic. Southeast Asian J Trop Med Public Health 18: 284-290.

2428. Soni A, Chugh K, Sachdev A, Gupta D (2001) Management of dengue fever in ICU. Indian J Pediatr 68: 1051-1055.

2429. Sonnenberg K, Niedrig M, Steinhagen K, Rohwader E, Meyer W, et al. (2004) State-of-the-art serological techniques for detection of antibodies against tick-borne encephalitis virus. Int J Med Microbiol 293: 148-151.

2430. Sorensen E (1992) Dengue hemoragisk feber. Erfaringer fra Thailand. Tidsskr Nor Laegeforen 112: 2194-2195.

2431. Sosothikul D, Seksarn P, Pongsewalak S, Thisyakorn U, Lusher J (2007) Activation of endothelial cells, coagulation and fibrinolysis in children with Dengue virus infection. Thromb Haemost 97: 627-634.

2432. Souares Y (1997) DENGUE - SAMOA. In: Body PC, editor. <http://www.spc.org.nc/> ed: PROMED.

2433. Souares Y (1997) DENGUE - FRENCH POLYNESIA. In: Body PPHSC, editor: PROMED.

2434. Souares Y (1997) DENGUE - NEW CALEDONIA. In: Body PPHSC, editor: PROMED.

2435. Souares Y (1997) DENGUE - COOK ISLANDS. In: Body PPHSC, editor: PROMED.

2436. Souares Y (1997) DENGUE - FRENCH POLYNESIA (02). In: Body PPHSC, editor: PROMED.

2437. Souares Y (1998) DENGUE ALERT - FED. STATES OF MICRONESIA (KOSRAE). In: Network PPHS, editor: PROMED.

2438. Souares Y (1998) DENGUE ALERT - WALLIS & FUTUNA. In: Network PPHS, editor: PROMED.

2439. Souares Y, Kiedrzynski T (1997) DENGUE - PACIFIC AREA (03) [AUSTRALIA]. In: Programme SCH, editor. Pacific Public Health Surveillance Coordinating Body. (Australia): PROMED.

2440. Soundravally R, Hoti SL (2007) Immunopathogenesis of dengue hemorrhagic fever and shock syndrome: Role of TAP and HPA gene polymorphism. Hum Immunol 68: 973-979.

2441. Soundravally R, Hoti SL (2008) Significance of transporter associated with antigen processing 2 (TAP2) gene polymorphisms in susceptibility to dengue viral infection. J Clin Immunol 28: 256-262.

2442. Soundravally R, Hoti SL (2008) Polymorphisms of the TAP 1 and 2 gene may influence clinical outcome of primary dengue viral infection. Scand J Immunol 67: 618-625.

2443. Soundravally R, Sankar P, Bobby Z, Hoti SL (2008) Oxidative stress in severe dengue viral infection: Association of thrombocytopenia with lipid peroxidation. Platelets 19: 447-454.

2444. Soundravally R, Sankar P, Hoti SL, Selvaraj N, Bobby Z, et al. (2008) Oxidative stress induced changes in plasma protein can be a predictor of imminent severe dengue infection. Acta Trop 106: 156-161.

2445. Souza LJ, Alves JG, Nogueira RM, Gicovate Neto C, Bastos DA, et al. (2004) Aminotransferase changes and acute hepatitis in patients with dengue fever: analysis of 1,585 cases. Braz J Infect Dis 8: 156-163.

2446. Speare R (1999) DENGUE - AUSTRALIA (QUEENSLAND). Townsville, Australia: PROMED.

2447. Spence L (1997) DENGUE - TRINIDAD AND TOBAGO. newspaper report ed: PROMED.

2448. Spence L, Jonkers AH, Casals J (1969) Dengue type 3 virus isolated from an antiguan patient during the 1963-64 Caribbean epidemic. Am J Trop Med Hyg 18: 584-587.

2449. Spigelblatt L, Rosenfeld R, Bonny Y, Laverdiere M (1980) Dengue Hemorrhagic-Fever in North-America - a Case-Report. Pediatrics 66: 631-633.

2450. Srichaikul T, Punyagupta S, Kanchanapoom T, Chanokovat C, Likittanasombat K, et al. (2008) Hemophagocytic syndrome in Dengue hemorrhagic fever with severe multiorgan complications. J Med Assoc Thai 91: 104-109.

2451. Srihongse S, Deibel R, Sather GE, Woodall JP (1982) Imported dengue fever in New York State. N Y State J Med 82: 1057-1059.

2452. Srikiatkhachorn A, Ajariyakhajorn C, Endy TP, Kalayanarooj S, Libraty DH, et al. (2007) Virus-induced decline in soluble vascular endothelial growth receptor 2 is associated with plasma leakage in dengue hemorrhagic fever. J Virol 81: 1592-1600.
[truncated: 70,533 more chars]
