# Supplementary material for: Refining the Global Spatial Limits of Dengue Virus Transmission by Evidence-Based Consensus
Source: PLoS Negl Trop Dis. 2012 Aug 7;6(8):e1760. doi: 10.1371/journal.pntd.0001760 (PMC3413714; doi:10.1371/journal.pntd.0001760)
Supplement: Table S1 — The collection of evidence used to assess evidence consensus for each country and Admin1 and Admin2 areas. Details of the scoring system can be found in the Methods section of the main manuscript. Scores for each category are highlighted in red. Evidence consensus is calculated as the percentage of the maximum possible score (see Fig. 2 in the main manuscript). HE = healthcare expenditure, DENV = dengue virus, DHF = dengue haemorrhagic fever, PCR = polymerase chain reaction, DF = dengue fever. (DOC) [file pntd.0001760.s009.doc]

**Table S1 Evidence collection by country, Admin1 and Admin2 regions.**

| **Country** | **Health organization status** | | | **Peer-reviewed evidence** | **Case data** | **Supplementary evidence** | **Ad-hoc adjustment** | **Evidence consensus** |
| --- | --- | --- | --- | --- | --- | --- | --- | --- |
|  | **WHO** | **CDC** | **GID-**  **EON** |  |  |  |  |  |
| Afghanistan | No | No | No  **-6** | 3.9% sero-positive for Dengue virus . Canadian soldier diagnosed with Dengue fever in 2004 .  **6** | HE low + sporadic cases  **6** | Other arboviruses present. Ae. aegypti nationwide . News reports from the Afghan-Pakistan border reporting to Quetta hospital (Pakistan).  **4** | **+2** for dengue advisory around Kandahar and lack of health organisation recognition | 40% |
| Albania | No | No | No  **-6** | - | HE medium.  **-3** | **-** | - | -60% |
| Algeria | No | No | No  **-6** | - | HE medium.  **-3** | - | - | -60% |
| American Samoa | Yes | No | Yes  **3** | - | Out-breaks reported: major 2001-2, minor 2008-2009. 53 confirmed cases in 2007 .  **9** | Numerous news reports of dengue cases on the rise. Travel advisory. *Ae. aegypti* present .  **4** | - | 76% |
| Andorra | No | No | No  **-6** | - | HE high.  **-9** | - | - | -100% |
| Angola | Yes | Yes | Yes  **6** | Dengue isolated from a Brazilian traveller in 1988 . Dengue isolated from a European traveller 1999-2002 . DENV-1 isolated in 1988 .  **4.66** | HE medium.  **-3** | Multiple circulating arboviruses . Travel advisory. *Ae. Aegypti* present .  **4** | - | 39% |
| Anguilla | Yes | Yes | Yes  **6** | DENV-1 isolated from a patient in 2008 .  **6** | 4 cases in 1995 and 16 cases reported in 2002 .  **6** | - | - | 75% |
| Antigua and Barbuda | Yes | Yes | Yes  **6** | 7 cases serologically confirmed in 1995 .  **3** | Switch from type 2 to type 4 Sept. 2011, but last large case numbers happened in 2001 (20 con-firmed cases) .  **6** | - | - | 63% |
| Netherlands Antilles | No | Yes | Yes  **3** | Sero-prevalence survey of dengue . DENV-2 serologically isolated from 1973 epidemic. .  **4** | 53 cases confirmed in 2007 Over 100 confirmed cases reported in October 2008 .  **9** | - | **+2** for support of recent case data. | 75% |
| Argentina- Buenos Aires df | Yes | No | Yes  **3** | DENV-3 serologically detected in 2007 .  **5** | Cases of dengue have previously been diagnosed and indigenous transmission ruled out + HE high.  **-9** | *Ae. aegypti* present . News reports.  **2** | - | 3% |
| Argentina- Buenos Aires | Yes | No | No  **-3** | - | 14 cases reported in 2009 , but below threshold + HE high.  **-9** | *Ae. aegypti* present . News reports.  **2** | **+2** for 2009 cases that highlight the uncertainty in this region. | -38% |
| Argentina- Catamarca | No | Yes | No  **-3** | Case description from 2009 . DENV-1 serologically found in 2009 .  **4.5** | Major dengue epidemic in the northern provinces in 2009 .  **9** | News reports. *Ae. aegypti* present .  **2** | - | 42% |
| Argentina- Chaco | Yes | Yes | No  **3** | Case description from 2009 . 2 dengue deaths and 3 DHF cases in 2009 . DENV-1 serologically found in 2009 .  **5** | Major dengue epidemic in the northern provinces in 2009 .  **9** | - | - | 71% |
| Argentina- Cordoba | Yes | No | No  **-3** | - | 97 confirmed cases in 2009 .  **9** | - | - | 40% |
| Argentina- Corrientes | Yes | No | No  **-3** | 2.3% Seropositive for dengue . 2 cases serologically diagnosed in 1990 .  **4** | 90 confirmed cases in 2009 .  **9** | Ae. aegypti present . News reports. Multiple other arboviruses present .  **4** | - | 47% |
| Argentina- Entre Rios | Yes | No | No  **-3** | - | 13 cases in 2009 but below threshold, HE high.  **-9** | *Ae. aegypti* present . News reports.  **2** | - | -48% |
| Argentina- Formosa | Yes | Yes | No  **3** | DENV-1 and DENV-3 in Formosa in 2009 . Case description in 2007 . DENV-1 isolated in 2000 .  **6** | Major dengue epidemic in the northern provinces in 2009 .  **9** | - | - | 75% |
| Argentina- Jujuy | Yes | Yes | No  **3** | DENV-1 serologically found in 2009 . DENV-1 isolated in 2000 .    **4.5** | Major dengue epidemic in the northern provinces in 2009 .  **9** | - | - | 69% |
| Argentina- Misiones | Yes | Yes | No  **3** | Case description from 2007 . DENV-1 isolated in 2000 .  **4.5** | Major dengue epidemic in the northern provinces in 2009 .  **9** | - | - | 69% |
| Argentina- | Yes | Yes | Yes  **6** | Case description from 2007 . 1 dengue death and 8 DHF cases in 2009 . DENV-1 serologically found in 2009 . DENV-2 isolated in 1998 .  **4.75** | Major dengue epidemic in the northern provinces in 2009 .  **9** | - | - | 82% |
| Argentina- Santa Fe | Yes | Yes | No  **3** | 4 cases in 2007 .  **4** | Outbreak in northern Santa Fe with 90 confirmed cases  **9** | **-** | - | 67% |
| Argentina- Santiago del Estero | Yes | Yes | No  **3** | - | 413 confirmed cases in 2009 .  **9** | - | - | 80% |
| Argentina- Tucuman | Yes | Yes | No  **3** | Cases description from 2009 .  **4** | Major dengue epidemic in the northern provinces in 2009 .  **9** | - | - | 67% |
| Argentina- Other states | No | No | No  **-6** | - | HE high.  **-9** | - | - | -100% |
| Armenia | No | No | No  **-6** | - | HE medium.  **-3** | - | - | -60% |
| Aruba | No | Yes | Yes  **3** | Molecular characterisation of 1985 outbreak strain .  **4** | A rise in sporadic cases and outbreaks (1985 and 2005), but little or no lab conformation .  **9** | - | - | 67% |
| Australia- Douglas | Yes | No | Yes  **3** | PCR isolation of dengue virus from patients in Port Douglas and Mossman 1997-1999 .  **5** | 76 cases detected over two transmission seasons .  **9** | - | - | 71% |
| Australia- Cardwell | Yes | No | Yes  **3** | Indigenous cases reported from Tully (3) and Wongaling Beach (1) in 2010 .  **5** | HE high  **-9** | - | - | -4% |
| Australia- Cairns | Yes | No | Yes  **3** | DENV-4 isolated by PCR from 1 patient in 2002 . PCR isolation of DENV-2 in 4 people in Kuranda.  **6.5** | 2003/4 Outbreak .  **9** | - | - | 77% |
| Australia- Dalrymple | Yes | No | Yes  **3** | 61.9% seropositive for dengue or closely related flaviviruses in a sample of 1000 people after the 1993 outbreak .  **3** | 1993 Outbreak .  **3** | *Ae. aegypti* present . No other supplementary evidence. | - | 38% |
| Australia- Johnstone | Yes | No | Yes  **3** | DENV-4 in Innisfail in 2007  **5** | 2008 outbreak  **9** | *Ae. aegypti* present . News reports.  **2** | - | 63% |
| Australia- Torres | Yes | No | Yes  **3** | 2 cases of DHF in 2004 . Sero detection of residents in 2003 .  **6** | 2003/4 Outbreak .  **9** | - | **-** | 75% |
| Australia- Townsville | Yes | No | Yes  **3** | 1 case serologically confirmed in 2002 .  **4** | 2003/4 Outbreak .  **9** | - | - | 67% |
| Australia- Other states | No | No | No  **-6** | - | HE high.  **-9** | - | - | -100% |
| Austria | No | No | No  **-6** | - | HE high.  **-9** | - | - | -100% |
| Azerbaijan | No | No | No  **-6** | - | HE medium.  **-3** | - | - | -60% |
| Bahamas | No | Yes | Yes  **3** | 2 serologically confirmed cases in the 1995 Caribbean outbreak .  **4** | Major epidemic in 1977 (1420 confirmed cases). Since then smaller epidemics every 5-9 years. Latest outbreak (2011) appears to be more serious .  **9** | - | - | 67% |
| Bahrain | No | No | No  **-6** | - | HE high.  **-9** | - | - | -100% |
| Bangladesh | Yes | Yes | Yes  **6** | DENV-2 and DENV-3 in Bangladesh . Predominance of DENV-3 during 2000 outbreak . 24.4% seropositive for dengue in 2000 . DHF case description .  **7** | 637 cases in 2011 .  **9** | - | - | 92% |
| Barbados | Yes | Yes | Yes  **6** | PCR identification of dengue in DF patients . 31% seropositive in 1996 .  **7** | Outbreak confirmed in 1997 . Lower case numbers since then .  **6** | - | - | 79% |
| Belarus | No | No | No  **-6** | - | HE medium.  **-3** | **-** | - | -60% |
| Belize | Yes | Yes | Yes  **6** | DHF confirmed in 2009 .  **6** | Outbreaks reported in 2005, 2007 and 2009 in different regions of the country .  **9** | - | - | 88% |
| Belgium | No | No | No  **-6** | - | HE high.  **-9** | - | - | -100% |
| Benin | No | No | Yes  **-3** | Returning aid workers from Germany 1987-1993 tested positive (14.8%) . DENV-3 isolated . Travellers from France returning with dengue .  **5.66** | HE low.  **3** | Other arboviruses present . *Ae. aegypti* present . News reports. Travel advisory.  **6** | - | 39% |
| Bermuda | No | No | Yes  **-3** | - | HE high.  **-9** | - | - | -80% |
| Bhutan | Yes | Yes | Yes  **6** | PCR isolation of multiple strains of dengue virus . Review of dengue status in Bhutan including PCR isolation .  **7** | Reports of low case numbers in towns near the Pakistan Border in 2006 , 2007 and 2009 .  **9** | - | - | 92% |
| Bolivia | Yes | Yes | Yes  **6** | PCR isolation of dengue . Vertical transmission among *Ae. aegypti* during 2007 outbreak demonstrated using PCR . Identification of DENV-2 from Santa Cruz .  **6.66** | Severe outbreak 2011 26,019 cases (circa 6,000 lab confirmed) and 36 deaths, viruses 1,2 and 3 circulating .  **9** | - | - | 90% |
| Bosnia and Herzegovina | No | No | No  **-6** | - | HE high.  **-9** | - | - | -100% |
| Botswana | No | No | No  **-6** | - | HE high.  **-9** | - | - | -100% |
| Bouvet Island | No | No | No  **-6** | - | HE high.  **-9** | - | - | -100% |
| Brazil | Yes | Yes | Yes  **6** | Multiple lineages within Rio de Janeiro 11.9% seroprevalence in Belo Horrizonte 2006 . Significant proportion of imported cases to Germany .  **8.67** | DF cases at 16,240 (Aug 2011) already. Many states countrywide reporting dengue cases daily .  **9** | - | - | 99% |
| British Indian Ocean Territory | No | No | No  **-6** | - | HE high.  **-9** | - | - | -100% |
| Brunei Darussalam | No | Yes | Yes  **3** | Detailed study of dengue infection in Brunei including PCR isolation . Genetic characterisation of DENV-1 .  **6** | Dengue outbreaks reported in recent years, but no case numbers given .  **9** | - | - | 75% |
| Bulgaria | No | No | No  **-6** | - | HE medium.  **-3** | - | - | -60% |
| Burkina Faso | Yes | Yes | Yes  **6** | Virus identified in *Ae. aegypti* 1983-1986 . Dengue in sylvatic cycles . 9.2% aid workers returning from Burkina Faso seropositive for dengue . 36.5% seropositive in urban areas .  **6.25** | 2006 outbreak, 683 people Ouagadougou, Nouna .  **9** | - | - | 89% |
| Burundi | No | No | No  **-6** | - | HE low.  **3** | Travel advisory. Multiple other circulating arboviruses .  **2** | - | -5% |
| Cambodia | Yes | Yes | Yes  **6** | Transmission of all 4 DENV . 50% seropositive for dengue in rural village .  **7** | Consistent case load every year, outbreaks in 2009 and 2011, very high death rate .  **9** | - | - | 92% |
| Cameroon | No | Yes | Yes  **3** | Detection of first indigenous cases and suggestion of *Ae. albopictus* as the vector . 12.5% rural adults positive for DENV-2 amongst other arboviruses . Serosurvey of 2000 people in Garoua, Yaounde and Douala, results unknown (Personnel communication) . Dengue cases reported in European travellers 1999-2002 . DENV-1 case detected in French military personnel 2010 .  **6.2** | No outbreaks recorded, but not in the list of diseases routinely checked for by health centres . Sporadic case + HE low.  **6** | - | **+3** for certainty and seroprevalence data for both urban and rural settings confirming dengue presence. | 76% |
| Canada | No | No | No  **-6** | - | HE high.  **-9** | - | - | -100% |
| Cape Verde | Yes | Yes | Yes  **6** | DENV-3 in Cape Verde 2009 . DENV-3 isolated from 5 French military personnel in 2010.  **6.5** | 2009 outbreak with 21,304 cases including DHF and deaths, subsequent lower case load in 2010 .  **9** | - | - | 90% |
| Cayman Islands | No | Yes | Yes  **3** | - | Consistent case numbers yearly since 2001, minor outbreaks in 2007 and 2010, including confirmed indigenous cases .  **9** | News reports of dengue cases both imported and native. Presence of *Ae. aegypti* and *Ae. albopictus* despite control programs in the wake of increased dengue cases in 2005 . Travel advisory.  **4** | - | 69% |
| Central African Republic | No | No | No  **-6** | Dengue serologically detected from 1 French soldier serving in 1995 . Dengue detected in a 11.5% of samples in a seroprevalence survey .  **3.5** | HE low.  **3** | Antibodies detected towards multiple arboviruses (including dengue) in population sample . *Ae. albopictus* detected for first time in domestic habitats, very wide intervals for epidemic risk indices .  Travel advisory.  **4** | - | 15% |
| Chad | No | No | No  **-6** | Dengue serologically diagnosed in 28 French soldiers from 1998-2010 .  **5** | HE low but sporadic cases.  **6** | Travel advisory. Other arboviruses present . *Ae. aegypti* present .  **4** | **+3** for repeated dengue infection over a long time period. | 40% |
| Chile- Easter Island | Yes | Yes | Yes  **6** | DENV-1 isolated . DENV-4 in 2009 .  **6.5** | Outbreak in 2002 . 2009 outbreak .  **9** | - | - | 90% |
| Chile-  Other states | No | No | No  **-6** | - | HE high.  **-9** | - | - | -100% |
| China- Fujian | Yes | Yes | Yes  **6** | DENV-1 isolated 1987 .  **5** | Outbreak 2004 at least 100 cases . 39 confirmed cases in 2007 with control efforts in response .  **9** | - | - | 83% |
| China- Guangdong | Yes | Yes | Yes  **6** | 71% clinical samples positive for DENV-1 (using PCR) in 2006 . DENV-2 isolated in 2001 .  **7** | Last outbreak Guangzhou 2003 .  **6** | - | - | 79% |
| China- Guangxi | Yes | No | Yes  **3** | DENV-3 isolated in 2001 . Dengue virus isolated from bats . 0.52% Chinese residents seropositive .  **6.66** | HE medium.  **-3** | News reports. *Ae. aegypti* present . Other arboviruses present .  **4** | **+3** for natural reservoir and sharing a rural land border with a highly endemic country. | 46% |
| China- Hainan | Yes | Yes | Yes  **6** | DHF detailed case description 1986 . 74% seropositive for dengue after the 1980 outbreak . 17% of wild bats seropositive for dengue in 1998 .  **3.33** | Big outbreak reported in 1980 including DHF, since then no reported outbreaks.  **-6** | News reports. *Ae. argypti* present . Other arboviruses present .  **4** | **+3** for natural reservoir persisting after latest outbreak. | 34% |
| China- Taiwan | Yes | Yes | Yes  **6** | DHF and case data investigation from 2001 outbreak . 9.23% seropositive for dengue in 1989 . DENV-2 isolated during 2002 outbreak .  **6.33** | Outbreaks reported every 2-3 years e.g. 2008 .  **9** | - | - | 89% |
| China- Yunnan | No | No | Yes  **-3** | 10.9% seropositive for dengue in 2010 . DENV-4 isolated from *Ae. albopictus* in 1975 .  **6.5** | 68 confirmed cases reported in 2008 with 20 indigenous cases from Burma .  **9** | - | **+3** for recent high seroprevalence. | 65% |
| China- Zhejiang | No | Yes | Yes  **3** | DENV-2 isolated from 2004 outbreak .  **7** | 83 cases reported in 2004 associated with an imported case from Thailand .  **9** | - | - | 79% |
| China-  Other states | No | No | No  **-6** | - | HE medium.  **-3** | - | - | -60% |
| Christmas Island | No | No | No  **-6** | - | HE high.  **-9** | - | - | -100% |
| Cocos (Keeling) Islands | No | No | No  **-6** | - | HE high.  **-9** | - | - | -100% |
| Colombia | Yes | Yes | Yes  **6** | Prolonged cocirculation of 2 DENV-3 lineages in Colombia . 23.3% residents seropositive in 2004 .  **7.5** | Dengue cases yearly (10,000-180,000) including 2011 including a high DHF and mortality rate .  **9** | - | - | 94% |
| Comoros | No | Yes | Yes  **3** | DENV-3 in returning European travellers . Seroprevalence study (overall 22.7%) .  **7** | Notable outbreaks reported in 1992-1993 and 2010 .  **9** | - | - | 79% |
| Congo | No | No | No  **-6** | - | HE low + News reports of outbreaks of dengue-like-disease (DLD).  **6** | News reports. Travel advisory.  **2** | - | 10% |
| Congo (Democratic Republic of) | Yes | Yes | No  **3** | Detection of dengue in seroprevalence surveys . Dengue isolated from a European traveller 1999-2002 .  **5** | HE low + Sporadic cases.  **6** | Multiple other arboviruses circulating . Travel advisory. *Ae. aegypti* present .  **4** | - | 60% |
| Cook Islands | No | No | Yes  **-3** | DENV-3 serologically detected in 1997 .  **4** | Outbreaks 1976, 2002, 2006 with (1000-200 cases) then a smaller outbreak in 2009 (60 cases), not confirmed.  **9** | Presence of *Ae. albopictus* and *Ae. aegypti* and speculation on their cause of dengue epidemics . Travel advisory. News reports. Other arboviruses in the Cook Islands .  **6** | **+2** for recent outbreaks not being officially reported. | 60% |
| Costa Rica | Yes | Yes | Yes  **6** | All 4 types detected in Costa Rica . 36.9% seropositive for dengue in coastal regions .  **7** | Epidemics in 1994 , 2009 , 2011 with thousands of cases including DHF and deaths. High number of cases reported yearly .  **9** | - | - | 92% |
| Cote d'Ivoire | Yes | Yes | Yes  **6** | Isolation of DENV-1 from a single patient . Imported cases to Japan and France . 28 DENV-2 isolates from 4 possible vectors . Serosurvey of 800 people in Abidjan on-going (commenced December 2011) .  **7** | 2008 outbreak in Abidjan .  **9** | - | - | 92% |
| Croatia- Korcula | No | No | No  **-6** | Dengue fever contracted autochthonously by a German tourist in Croatia .  **6** | HE high.  **-9** | - | - | -38% |
| Croatia- other states | No | No | No  **-6** | - | HE high.  **-9** | - | - | -100% |
| Cuba | Yes | Yes | Yes  **6** | Retrospective serological detection from 1981 epidemic . DHF in Cuba 1997 . DENV-2 identified in 1997 outbreak .  **5.33** | Dengue free until 1981 epidemic since then urban outbreaks ever 3-4 years .  **9** | - | - | 85% |
| Cyprus | No | No | No  **-6** | - | HE high.  **-9** | - | - | -100% |
| Czech Republic | No | No | No  **-6** | - | HE high.  **-9** | - | - | -100% |
| Denmark | No | No | No  **-6** | - | HE high.  **-9** | - | - | -100% |
| Djibouti | No | Yes | Yes  **3** | PCR isolation of DENV-1 from 1 patient . PCR isolations of DENV-2 . 164 cases reported amongst French military personnel serving in the country between 1998 and 2008 .  **6** | 1991-1993 outbreak with 12000 cases , outbreak amongst French military personnel in 2005 with 123 cases .  **9** | - | - | 75% |
| Dominica | Yes | Yes | Yes  **6** | DENV-4 PCR isolation in 1981 Caribbean epidemic . Serological confirmation of 9 dengue cases 1995 .  **5** | Reported case numbers have been steadily on the rise since 2005 including DHF cases. Outbreak declared (Sept-2011) for this year .  **9** | - | - | 83% |
| Dominican Republic | Yes | Yes | Yes  **6** | 91% adults seropositive . Cases detected in returning US travellers . DENV-1-4 detected .  **8.33** | Major epidemic in 2009 .  **9** | - | - | 97% |
| Ecuador | Yes | Yes | Yes  **6** | PCR identification of dengue in 5.3% of febrile illness patients . Present in 30% of wild bats . DHF in 2003 .  **7** | Epidemic declared in 2009 .  **9** | - | - | 92% |
| Egypt | No | No | Yes  **-3** | Dengue reported in Italian travellers to the area . 4 samples seropositive for dengue in 1975 .  **4** | HE low.  **3** | Ae. aeypti in Egypt . Other arboviruses in Egypt .  **2** | - | 20% |
| El Salvador | Yes | Yes | Yes  **6** | 9.8% seropositive for recent dengue infection . Characterisation of DSS in El Salvador . DENV-2 in El Salvador .  **7.33** | Consistently high case numbers since 1978, 4 outbreaks reported in the last 10 years e.g. 2007 including DHF and deaths.  **9** | - | - | 93% |
| Equatorial Guinea | No | Yes | No  **-3** | Dengue reported in a European traveller 1999-2002 .  **4** | HE high.  **-9** | Ae. albopictus present . Travel advisory. Other arboviruses present .  **4** | **+3** for high weight of HE expenditure despite some suggestions of dengue presence. | -3% |
| Eritrea | No | Yes | Yes  **3** | Dengue reported in Norwegian travellers to Eritrea .  **5** | Sporadic cases reported by News agencies + HE low.  **6** | - | - | 58% |
| Estonia | No | No | No  **-6** | - | HE high.  **-9** | - | - | -100% |
| Ethiopia | Yes | Yes | Yes  **6** | Dengue isolated from a returning European traveller 1999-2002 .  **4** | Sporadic cases reported + HE low.  **6** | - | - | 67% |
| Falkland Islands (Malvinas) | No | No | No  **-6** | - | HE high.  **-9** | - | - | -100% |
| Faroe Islands | No | No | No  **-6** | - | HE high.  **-9** | - | - | -100% |
| France- Bouches-du Rhone | No | No | No  **-6** | First cases of autochthonous dengue and Chikungunya infections within the established *albopictus* populations in Marseille .  **6** | HE high.  **-9** | **-** | - | -38% |
| France- other states | No | No | No  **-6** | - | HE high.  **-9** | **-** | - | -100% |
| French Guiana | Yes | Yes | Yes  **6** | 23.5% seropositive for dengue . DHF in 1992 . DENV-1-4 isolated and seasonal patterns identified .  **7** | Major epidemic 2009  **9** | - | - | 92% |
| French Polynesia | Yes | No | Yes  **3** | DENV-1 replaced by DENV-4 . Isolation of DENV-1 and evaluation of the incidence of DHF during the 2001 outbreak .  **6** | Outbreaks in 1971 and 1989, lower case numbers in the 1990’s, outbreaks reported in 2001, 2006, 2009 (20,425 cases including DHF and deaths)  **9** | - | - | 75% |
| French Southern Territories | No | No | No  **-6** | - | HE high.  **-9** | - | - | -100% |
| Finland | No | No | No  **-6** | - | HE high.  **-9** | - | - | -100% |
| Fiji | Yes | No | Yes  **3** | 13 deaths and detailed case data for 1997 outbreak . Epidemiological study of DENV-1 outbreak and DHF in 1989-90 . Cases serologically detected in 2011 .  **5.66** | Epidemics reported 1989, 1997, 2008 (1,000-3,500 cases) .  **9** | - | - | 74% |
| Gabon | Yes | Yes | No  **3** | Serological detection of dengue during an outbreak of chikungunya and dengue in 2007 . Dengue isolated from returning European travellers 1999-2002 . DENV-1 isolated from a French soldier in 2010 . PCR detection of multiple dengue cases from the 2007 outbreak and their differentiation from Chikungunya .  **7.25** | 2006-2007 outbreak 17,618 cases Chikungunya, only 618 dengue .  **9** | - | **+3** for questionnaire return detailing the extensive detection of dengue and specifically methods for ruling out chikungunya. DENV-1,2 and 3 are now present in Gabon. | 93% |
| The Gambia | Yes | Yes | No  **3** | Dengue reported in returning U.K. traveller .  **4** | HE low.  **3** | Travel advisory. *Ae. aegypti* and other arboviruses present .  **4** | - | 47% |
| Ghana | Yes | Yes | Yes  **6** | DENV-2 isolated from Finnish traveller to Ghana .  **6** | Numerous sporadic cases reported + HE low.  **6** | - | - | 75% |
| Gibraltar | No | No | No  **-6** | - | HE high.  **-9** | - | - | -100% |
| Georgia | No | No | No  **-6** | - | HE medium.  **-3** | - | - | -60% |
| Germany | No | No | No  **-6** | - | HE high.  **-9** | - | - | -100% |
| Greece | No | No | No  **-6** | - | HE high  **-9** | **-** | - | -100% |
| Grenada | Yes | Yes | Yes  **6** | 93% tested seropositive in a sample study . Widespread seropositive cases in 2009 .  **6** | Epidemics reported in 2001-2002 and 2010 .  **9** | - | - | 88% |
| Greenland | No | No | No  **-6** | - | HE high.  **-9** | - | - | -100% |
| Guadeloupe | Yes | Yes | Yes  **6** | DENV-4 isolation from patient . DHF reported 1998 .  **6.5** | 3 outbreaks since2005 with increasing case numbers including DHF and deaths .  **9** | - | - | 90% |
| Guam | No | No | Yes  **-3** | - | Official government bodies declaring Guam dengue free. + HE high.  **-9** | - | - | -80% |
| Guatemala | Yes | Yes | Yes  **6** | Isolation of DENV-2 and DENV-4 from 2007 Guatemalan epidemic .  **7** | Background cases since 1996, major epidemics in 2005 and 2010 .  **9** | - | - | 92% |
| Guernsey | No | No | No  **-6** | - | HE high.  **-9** | - | - | -100% |
| Guinea | Yes | Yes | Yes  **6** | Dengue accounts for 2% of febrile illness . Some dengue serologically detected in a wide viral survey . DENV-2 isolated in 1981 .  **3.67** | HE low.  **3** | - | **+2** for consistent findings of multiple surveys despite their age. | 61% |
| Guinea-Bissau | No | No | No  **-6** | Dengue reported in returning Spanish traveller .  **6** | Dengue outbreaks have occurred recently in Guinea-Bissau and at least 2 expats working in the country have presented with dengue back in Scandinavia .  **9** | *Aedes spp.* present . News reports of dengue aid from Cuba. Other arboviruses in Guinea-Bissau . Travel advisory.  **6** | **+3** for questionnaire response confirming dengue. | 60% |
| Guyana | Yes | Yes | Yes  **6** | 45% of febrile illness patients tested positive for dengue .  **4** | Steady case numbers with outbreaks in 2006 and 2010 .  **9** | - | - | 79% |
| Haiti | Yes | Yes | Yes  **6** | 65% of infants seropositive for dengue by 36 months . Dengue reported in American travellers returning from Haiti [[193](#_ENREF_193)][[188](#_ENREF_188)][[188](#_ENREF_188)][[187](#_ENREF_187)][[187](#_ENREF_187)][[187](#_ENREF_187)][[186](#_ENREF_186)][[180](#_ENREF_180)] DENV-1,2 and 4 isolated in 1994-1996.  **7.66** | A lack of reporting of DHF cases before 2000 played down the scale of the problem until 3144 cases including 10 deaths were reported during the epidemic of that year . Yearly epidemics since .  **9** | - | - | 94% |
| Heard Island and McDonald Islands | No | No | No  **-6** | - | HE high.  **-9** | - | - | -100% |
| Holy See (Vatican City State) | No | No | No  **-6** | - | HE high.  **-9** | - | - | -100% |
| Honduras | Yes | Yes | Yes  **6** | Isolation of DENV-2 and DENV-4 from 2007 epidemic . Dengue detected in 0.3% of asymptomatic Honduran blood donors .  **7.5** | Major epidemic in 2009 .  **9** | - | - | 94% |
| Hong Kong | Yes | Yes | Yes  **6** | Serologically detected cases on Ma island in 2001 . 8% of dengue cases are indigenous .  **5** | Consistent case load year on year and first autochthonous outbreak (10cases) in 2010.(below threshold, HE medium) .  **-3** | Travel advisory, News reports of confirmed cases, *Ae. albopictus* present .  **4** | **+3** for on going low level detection of indigenous dengue. | 50% |
| Hungary | No | No | No  **-6** | - | HE high.  **-9** | - | - | -100% |
| Iceland | No | No | No  **-6** | - | HE high.  **-9** | - | - | -100% |
| India- Arunachal Pradesh | Yes | Yes | Yes  **6** | Dengue case description from 1963 .  **2** | HE low.  **3** | News reports. *Ae. aegypti* and *Ae. albopictus* both present . Other arboviruses present .  **4** | **-3** for no detection of cases during 2011 outbreak. | 50% |
| India- Assam | Yes | Yes | Yes  **6** | 10% of febrile illness patients seropositive for dengue (3.6% also positive for chikungunya) .  **5** | 117 cases reported in 2010 with 20 deaths .  **9** | - | **-3** for uncertainty over seroprevalence results conducted during a chikungunya outbreak. | 71% |
| India- Chhattisgarh | Yes | Yes | Yes  **6** | - | 43 cases with 3 deaths in October 2011 .  **9** | News reports. Other arboviruses present . *Ae. aegypti* present .  **4** | **-3** for co-detection during the chikungunya outbreak in 2011. | 76% |
| India- Himachal Pradesh | Yes | Yes | Yes  **6** | 2 patients from Kangra admitted to a hospital in Ludhiana (200km away) in 2005 with suspected dengue .  **4** | HE low.  **3** | News reports. *Ae. aegypti* present .  **2** | - | 50% |
| India- Jammu and Kashmir | No | Yes | No  **-3** | - | 1 case reported in 2011 . HE low.  **3** | - | **-3** for seronegative evidence and minimal cases despite poor surveillance. | -20% |
| India- Jharkhand | Yes | Yes | Yes  **6** | Linking 2006 outbreak with 1000 cases (13 confirmed) to high larval indices .  **4** | 16 cases reported (some confirmed) in 2011 , none before 2011 (below threshold)  HE low.  **3** | News reports. *Ae. aegypti* present . Other arboviruses present .  **4** | - | 57% |
| India- Meghalaya | Yes | Yes | Yes  **6** | - | HE low.  **3** | News reports. *Ae. aegypti* and *Ae. albopictus* both present. Other arboviruses present .  **4** | **-3** No cases reported in 2011 epidemic. | 48% |
| India- Mizoram | Yes | Yes | Yes  **6** | - | HE low.  **3** | *Ae. aegypti* and *Ae. albopictus* present . Other arboviruses present .  **2** | - | 52% |
| India- Nagaland | Yes | Yes | Yes  **6** | DENV-2 in Nagaland in 1995 , DHF characterised in 1995 .  **5** | HE low.  **3** | News reports. *Ae. albopictus* present . Other arboviruses present .  **4** | **-3** No cases reported in 2011 epidemic. | 50% |
| India- Orissa | Yes | Yes | Yes  **6** | 14 patients with serological detection of dengue 1995 .  **4** | 1811 cases reported in 2011 .  **9** | - | **-3** for uncertainty over case numbers in 2011 in light of likely mis-diagnosis of dengue when chikungunya is present. | 67% |
| India- Sikkim | Yes | Yes | No  **3** | - | 2 cases reported October-November 2011 but below threshold and HE low.  **3** | No supplementary information available. | - | 40% |
| India- Tripura | Yes | Yes | Yes  **6** | - | HE low.  **3** | *Ae. aegypti* and *Ae. albopictus* present . Other arboviruses present .  **2** | - | 52% |
| India- other states | Yes | Yes | Yes  **6** | 7% of undiagnosed febrile illness patients have dengue fever . Evidence of all 4 DENV types circulating in a single epidemic . 19% of Austrian imported cases come from India .  **8.66** | Almost yearly e.g. 2009 epidemics spread over all parts of the country often with very high casualties in urban areas .  **9** | - | - | 99% |
| Indonesia | Yes | Yes | Yes  **6** | Up to 69% of schoolchildren seropositive for dengue . DENV-3 isolated 2006 . 15% of imported cases into Austria come from Indonesia .  **8.33** | 1 or 2 notable outbreaks each decade e.g. 2009 with high incidences of DHF and deaths .  **9** | - | - | 97% |
| Iran | No | No | No  **-6** | - | First imported case only detected in 2008 +HE medium.  **-3** | - | **+1** for uncertainty over accuracy of case data. | -53% |
| Iraq | No | No | No  **-6** | - | HE medium.  **-3** | - | - | -60% |
| Ireland | No | No | No  **-6** | - | HE high.  **-9** | - | - | -100% |
| Isle of Man | No | No | No  **-6** | - | HE high.  **-9** | - | - | -100% |
| Israel | No | No | No  **-6** | - | HE high.  **-9** | - | - | -100% |
| Italy | No | No | No  **-6** | - | HE high.  **-9** | - | **+3** for recent establishment of *Aedes albopictus* and a chikungunya outbreak in invaded albopictus population in 2007 . | -80% |
| Jamaica | Yes | Yes | Yes  **6** | Review of DEV types isolated in Jamaica . Detailed case description .  **5.5** | 2007 Outbreak .  **9** | - | - | 85% |
| Japan | No | No | No  **-6** | - | HE high.  **-9** | - | - | -100% |
| Jersey | No | No | No  **-6** | - | HE high.  **-9** | - | - | -100% |
| Jordan | No | No | No  **-6** | - | HE medium.  **-3** | - | - | -60% |
| Kazakhstan | No | No | No  **-6** | - | HE medium.  **-3** | - | - | -60% |
| Kenya | Yes | Yes | Yes  **6** | Multidistrict seroprevalence study reporting 14% seropositive for dengue in adults . DENV-2 isolated 1999 .  **7.5** | Outbreaks reported 1982 and 2011 .  **9** | - | - | 94% |
| Kiribati | Yes | No | Yes  **3** | Isolation of DENV-4 from 2008 epidemic .  **7** | Outbreaks in 1980 and 2008 (both around 800 cases and DHF) .  **9** | - | **-2** for long intervals between recorded outbreaks. | 71% |
| Korea (Democratic People's Republic of) | No | No | No  **-6** | - | HE assumed low.  **3** | - | **-3** although HE is low in North Korea, its climate is highly unsuitable for Aedes and it is surrounded by countries that have proven dengue absences along their border with North Korea. | -40% |
| Korea (Republic of)-Gyeongsangnam-do | No | No | No  **-6** | One confirmed case with a travel history to Indonesia (39 days before symptoms), but long outside the latency period for dengue (14 days) suggesting autochthonous transmission. Mosquitoes were tested for presence of the virus, but no positive results were found .  **5** | HE high.  **-9** | - | **-3** for the lack of certainty surrounding whether this documents an imported or autochthonous case. | -51% |
| Korea, (Republic of)- All other states | No | No | No  **-6** | - | HE high.  **-9** | - | - | -100% |
| Kuwait | No | No | No  **-6** | 14% seropositive for dengue antibodies .  **3** | HE high.  **-9** | - | - | -50% |
| Kyrgyzstan | No | No | No  **-6** | - | HE low.  **3** | No supplemental evidence found. | - | -20% |
| Lao People's Democratic Republic | Yes | Yes | Yes  **6** | Cross-sectional survey revealed 13% seropositive to dengue 2008 . DENV-2 isolated in 2002 .  **7** | Consistent case numbers and deaths, outbreaks in 1994 and 2010 (21,509 cases) .  **9** | **-** | - | 92% |
| Latvia | No | No | No  **-6** | - | HE high.  **-9** | - | - | -100% |
| Lebanon | No | No | No  **-6** | - | HE high.  **-9** | - | - | -100% |
| Lesotho | No | No | No  **-6** | - | HE low.  **3** | - | **-3** despite low HE dengue cases would likely be detected in neighbouring South Africa. | -40% |
| Liberia | No | No | No  **-6** | - | Small outbreaks reported + HE low.  **6** | Travel advisory. News reports of confirmed dengue cases. Other arboviruses present in Liberia . *Ae. aegypti* present .  **6** | - | 29% |
| Libyan Arab Jamahiriya | No | No | No  **-6** | - | HE medium.  **-3** | - | - | -60% |
| Liechtenstein | No | No | No  **-6** | - | HE high.  **-9** | - | - | -100% |
| Lithuania | No | No | No  **-6** | - | HE high.  **-9** | - | - | -100% |
| Luxembourg | No | No | No  **-6** | - | HE high.  **-9** | - | - | -100% |
| Macao | Yes | Yes | Yes  **6** | 48% of native Macao residents seropositive for dengue .  **5** | Low levels of cases yearly, an outbreak declared in 2001 and higher case numbers in 2004 and 2009. DHF used to be present, but no cases since 2001 .  **9** | - | - | 83% |
| Macedonia, the former Yugoslav Republic of | No | No | No  **-6** | - | HE medium.  **-3** | - | - | -60% |
| Madagascar | Yes | Yes | Yes  **6** | Seroprevalence surveys confirmed dengue on 2 islands in Madagascar . Serological and entomological survey of 2006 outbreak revealing *Ae. albopictus* as the vector .  **5** | 2006 outbreak .  **9** | **-** | - | 83% |
| Malawi | No | No | No  **-6** | - | Sporadic cases reported +HE low.  **6** | Reference to multiple circulating arboviruses in Malawi . *Ae. aegypti* present . Travel advisory.  **4** | Personal communication with Palwasha Khan reveals there is no dengue detecting infrastructure of any kind in Malawi, therefore uncertainty remains +0. | 19% |
| Malaysia | Yes | Yes | Yes  **6** | 91.6% of the population seropositive . PCR isolation of DENV . 10% imported dengue cases into Austria involve travel to Malaysia .  **8.66** | Outbreaks 2-3 time a decade e.g. 2009, 24,534 cases and consistently high case and death numbers .  **9** | - | - | 99% |
| Maldives | No | Yes | Yes  **3** | Description of dengue cases . 6% seropositive for dengue .  **5** | Steady yearly case loads since 1998. Outbreaks reported in 1988, 1998, 2006 , 2008 , 2010 and 2011 including high rates of DHF and deaths .  **9** | - | - | 71% |
| Mali | Yes | Yes | Yes  **6** | Seroprevalence survey reveals 93% of febrile illness patients are seropositive for dengue . DENV-3 in suspected 2008 outbreak . Imported case into France 2008 .  **7.33** | 2008 suspected outbreak with 70 unconfirmed cases .  **9** | - | - | 93% |
| Malta | No | No | No  **-6** | - | HE high.  **-9** | - | - | -100% |
| Marshall Islands | Yes | No | Yes  **3** | DENV-4 detected by PCR in 2011 .  **7** | Outbreak December 2011 in Ebeye. 101 suspected cases, 46 confirmed .  **9** | **-** | - | 79% |
| Martinique | Yes | Yes | Yes  **6** | Case reports of DSS . DENV-3 in Martinique .  **6.5** | High case numbers (>100) since 1995. Outbreaks reported 1997, 2001, 2005, 2007, 2008 with growing case and death numbers .  **9** | - | - | 90% |
| Mauritania | No | No | No  **-6** | - | HE low.  **3** | Ae. aegypti present . Multiple other circulating arboviruses .  **2** | - | -5% |
| Mauritius | No | Yes | Yes  **-3** | DENV-2 isolated . 3.8% seropositive in Mauritius (Pre2009 outbreak) .  **7.5** | 2009 outbreak where 252 cases were reported in a short epidemic.  **9** | News reports. Travel advisory. *Ae. albopictus* present . Other arboviruses present .  **6** | - | 65% |
| Mayotte | No | Yes | Yes  **3** | 22.7% seropositive for dengue . Chikungunya and dengue seropositivity identified in household studies post 2005-6 chikungunya .outbreak . DENV-1 isolated from a French soldier in 2009 .  **6** | Dengue epidemic reported in 2005-6 chikungunya outbreak .  **9** | - | - | 75% |
| Mexico | Yes | Yes | Yes  **6** | Seroprevalence survey revealed 2% of blood donors and 7.5% of the population of Northern Mexico were seropositive for dengue . PCR isolation of DENV . 4% imported cases into Austria come from Mexico .  **8.33** | 7 outbreaks reported in the last 20 years including 1100 cases in 2008, very high rates of DHF reported .  **9** | - | - | 97% |
| Micronesia, Federated States of | No | No | Yes  **-3** | DHF/DSS described in 2004 . DENV-1 isolated from travellers to Yap state in 2004 .  **7** | Epidemics in Yap State in 2011 .  **9** | - | **+3** for lack of health bodies keeping up with case data in an isolated country. | 67% |
| Moldova, Republic of | No | No | No  **-6** | - | HE medium.  **-3** | - | - | -60% |
| Monaco | No | No | No  **-6** | - | HE high.  **-9** | - | - | -100% |
| Mongolia | No | No | No  **-6** | - | HE low.  **3** | - | **-3** although HE is low in Mongolia, its climate is highly unsuitable for Aedes and it is surrounded by countries that have proven dengue absences along their border with Mongolia. | -40% |
| Montenegro | No | No | No  **-6** | - | HE high.  **-9** | - | - | -100% |
| Montserrat | Yes | Yes | Yes  **6** | PCR isolation of DENV-4 from 1994 outbreak .  **4** | Outbreak in 1994-5 (around 1000 cases), sporadic cases since then every few years .  **3** | News reports. Travel advisory.  **2** | - | 50% |
| Morocco | No | No | No  **-6** | - | HE medium.  **-3** | - | - | -60% |
| Mozambique | Yes | Yes | Yes  **6** | DENV-3 transmission documented in 1984 outbreak . Dengue isolated from a European traveller 1999-2002 . DENV-3 isolate in 1985 .  **5** | Outbreak 1984, 2 fatal cases .  **-3** | Travel advisory. *Ae. albopictus* detected . Other arboviruses detected .  **4** | - | 40% |
| Myanmar | Yes | Yes | Yes  **6** | DENV-1 PCR isolation . Review of DHF cases in Myanmar . 10-60% seropositive dependent of region .  **6.33** | Outbreaks declared 1970, 2001, 2009 , 2010, 2011. Very high death rate .  **9** | - | - | 89% |
| Namibia | No | No | No  **-6** | Dengue isolated from a returning European traveller 1999-2002 .  **4** | HE medium.  **-3** | - | **-3** for 2 serosurveys finding no dengue and very low levels of other arboviruses . | -33% |
| Nauru | Yes | No | Yes  **3** | DENV-1 isolated by PCR from 1974 outbreak .  **4** | Outbreaks reported in 1972, 1974 and 1984 .  **-3** | Travel advisory. *Ae. aegypti* present .  **2** | - | 20% |
| Netherlands | No | No | No  **-6** | - | HE high.  **-9** | - | - | -100% |
| Nepal | Yes | Yes | Yes  **6** | Seroprevalence 30% among DLD . Identification of all DENV serotypes in Nepal . Report on 2010 outbreak including DHF and identifying *Ae. aegypti* as the vector .  **7.66** | No cases until 2006 . Outbreak in 2010 (7,000 cases), still cases being reported in 2011 .  **9** | - | - | 94% |
| New Caledonia | Yes | Yes | Yes  **6** | Displacement of DENV-1 by DENV-4 in 2007 . description of DSS and DHF .  **7** | Outbreaks 1-2 times a decade e.g. 2009 , consistent case numbers yearly, deaths in epidemic years .  **9** | - | - | 92% |
| New Zealand | No | No | No  **-6** | - | HE high  **-9** | - | - | -100% |
| Nicaragua | Yes | Yes | Yes  **6** | DENV-2 serologically detected in 75% of febrile illness cases . 12% seroprevalence among school children .  **6** | Outbreaks in 1985, 1994, 1998, 2009 , 2010 (up to 20,000 cases including DHF and deaths) .  **9** | - | - | 88% |
| Niger | No | No | No  **-6** | - | HE low.  **3** | Travel advisory. *Ae. aegypti* present . Multiple arboviruses present .  **4** | - | 5% |
| Nigeria | Yes | Yes | Yes  **6** | Seroprevalence to DENV-2 was 46% in Kainji lake area 63% seroprevalence to multiple arboviruses . DENV-3 isolated from Nigeria . Dengue isolated from European travellers 1999-2002 .  **5.75** | Sporadic cases reported + HE low.  **6** | *Ae. albopictus* identified in Nigeria in 1991 . Many other arboviruses also present . Travel advisory.  **6** | - | 79% |
| Niue | Yes | No | Yes  **3** | PCR identification of DENV-2 from 1972 outbreak . Serological detection of DENV-3 during 1986 epidemic .  **3.5** | Big epidemics in 1972, 1980 and 1986, no more than 2 cases a year since 1988 , until 2012 outbreak with 20 cases to date (Apr 2012) .  **9** | - | **-** | 65% |
| Norfolk Island | No | No | No  **-6** | **-** | HE high.  **-9** | **-** | - | -100% |
| Northern Mariana Islands | Yes | No | Yes  **3** | 3 children without a history of international travel tested seropositive for dengue .  **4** | 1,418 cases reported during 2001 outbreak. None since .  **6** | - | - | 54% |
| Norway | No | No | No  **-6** | - | HE high.  **-9** | - | - | -100% |
| Oman | No | Yes | No  **-3** | - | 8 imported cases 2001-2004 (2 autochthonous) . All below threshold HE medium.  **-3** | - | **+3** for uncertainty surrounding unidentified imported cases. | -20% |
| Pakistan- Azad Kashmir | Yes | Yes | No  **3** | - | HE low.  **3** | News reports. Arboviruses present . *Ae. aegypti* present .  **4** | - | 48% |
| Pakistan- Baluchistan | No | No | Yes  **-3** | 45 construction workers seropositive for dengue in 1995 .  **3** | HE low.  **3** | News reports. *Ae. aegypti* present . Other arboviruses present .  **4** | - | 23% |
| Pakistan- Gilgit-Baltistan | Yes | No | No  **-3** | - | HE low.  **3** | **-** | **-3** for remote mountain region unlikely to have case data therefore suffers disproportionately in case score category. | -80% |
| Pakistan- Islamabad | Yes | No | No  **-3** | Description of 825 cases in 2006 . 5 serologically confirmed cases in Islamabad in 2009 .  **4.5** | December 2011 outbreak .  **9** | News reports. No other supplementary evidence found. | **+3** for growing amount of confirmed cases, but no recognition at the official health body level. | 56% |
| Pakistan- Khyber Pakhtunkhwa | Yes | No | No  **-3** | Dengue case data from 2006 . 7 people diagnosed with dengue died in 2009- suspected outbreak, but lack of conformation makes confirming it difficult .  **4** | Reported dengue deaths in 2009 and 2012 and 220 confirmed cases reported in 2011 .  **9** | News reports. *Ae. aegypti* present .  **2** | **+3** for growing importance and recognition not yet acknowledged by health bodies. | 50% |
| Pakistan- Punjab | Yes | Yes | Yes  **6** | Case description from 2008 . Case description from 2009 .  **4** | 2011 outbreak .  **9** | - | - | 79% |
| Pakistan- Sindh | Yes | Yes | Yes  **6** | Detailed case report of DHF and DSS in Karachi 2005 . DENV-2 and DENV-3 isolated in 2006 .  **7** | 2012 outbreak .  **9** | - | - | 92% |
| Pakistan- Tribal areas | Yes | No | No  **-3** | Patients serologically diagnosed in 2010 .  **5** | HE low.  **3** | News reports. No other supplementary evidence. | **+3** for neighbouring cases and poor public health surveillance. | 33% |
| Palau | Yes | Yes | Yes  **6** | Review of risk factors during 2000 outbreak and isolation of DENV-4 virus .  **5** | Outbreaks reported in 1995 and 2000, steady case numbers since 1995 .  **6** | - | - | 71% |
| Panama | Yes | Yes | Yes  **6** | Clinical study of children during the 2001 and 2005 epidemics including detailed description of DHF . DENV-3 isolated by PCR from Ae*. albopictus* in 1994 epidemic .  **6** | Outbreaks in 1994, 2004 , 2011, consistent case numbers yearly including DHF and deaths .  **9** | - | - | 88% |
| Papua New Guinea | Yes | Yes | Yes  **6** | Dengue diagnosed in 8% of febrile illness patients negative for malaria . Multiple cases of dengue imported into Queensland from Papua New Guinea . DENV-1 and 2 identified .  **6.33** | Outbreaks reported in 1971 and 1976 , elevated case numbers through the 1980’s, a few reported cases since .  **-6** | Travel advisory. News reports PNG residents being diagnosed with dengue in Australia. *Ae. aegypti* present . Other arboviruses present .  **6** | **+2** for what appears to be a decline in reporting ability as there has been no decline in cases imported from Papua New Guinea. | 48% |
| Paraguay | Yes | Yes | Yes  **6** | 26% dengue seropositive in 2007 . PCR identification of DENV-3 in 2002 outbreak . Dengue cases imported to Argentina from Paraguay (ELISA diagnosed) .  **7.37** | Cases / observation of cases began with 1999 outbreak, 4 outbreaks since (2011 26,792 cases with 62 deaths) .  **9** | - | - | 97% |
| Peru | Yes | Yes | Yes  **6** | 26% of febrile illness patients tested positive for dengue . DENV-4 in north-eastern Peru .  **8** | Outbreaks 1991, 2001 , 2010 including DHF and some deaths .  **9** | - | - | 96% |
| Philippines | Yes | Yes | Yes  **6** | DENV-3 isolated and identified as the predominant type between 2007 and 2009 . 94.9% of febrile illness patients tested seropositive for dengue . 19% imported cases into Austria from the Philippines .  **8.66** | Consistently high case numbers yearly including DHF and deaths, 4 outbreaks reported in the last 10 years (up to 120,000 cases) e.g., 2009 .  **9** | **-** | - | 99% |
| Pitcairn | No | No | No  **-6** | - | HE high.  **-9** | - | - | -100% |
| Poland | No | No | No  **-6** | - | HE high.  **-9** | - | - | -100% |
| Portugal | No | No | No  **-6** | - | HE high.  **-9** | - | - | -100% |
| Puerto Rico | Yes | Yes | Yes  **6** | Clinical and Epidemiological observations from the 1986 outbreak including isolation of DENV1 and 4. . Serological survey revealed 37% seropositive for DENV . Re-emergence of DENV-2 in Puerto Rico .  **8.33** | 2-3 outbreaks per decade e.g. 2007 , case numbers in the thousands including DHF and deaths  **9** | - | - | 97% |
| Qatar | No | No | No  **-6** | - | HE high.  **-9** | - | - | -100% |
| Reunion | No | No | Yes  **-3** | Case descriptions from 1977 and 2004 epidemics . Isolation of DENV-2 . Case description of 1996 dengue outbreak .  **3** | Outbreak reported in 1977. Dengue cases have been detected during chikungunya outbreaks in 2004 and 2010  **9** | Travel advisory. News reports. *Ae. albopictus* present and other circulating arboviruses .  **6** | Evidence for difficulty of detection during Chikungunya outbreaks given a -2 to reflect the uncertainty in the case detection data. | 43% |
| Romania | No | No | No  **-6** | - | HE high.  **-9** | - | - | -100% |
| Russian Federation | No | No | No  **-6** | - | HE high.  **-9** | - | - | -100% |
| Rwanda | No | No | No  **-6** | Dengue detected in 1 of 33 German aid workers .  **4** | HE low.  **3** | Travel advisory. No other supplementary evidence. | - | 4% |
| Saint Helena | No | No | No  **-6** | - | HE high.  **-9** | - | - | -100% |
| Saint Kitts and Nevis | Yes | Yes | Yes  **6** | Serologically confirmed cases in 1995 .  **4** | Consistent number of cases with peaks every decade e.g. 2008 (49 cases) .  **9** | - | - | 79% |
| Saint Lucia | Yes | Yes | Yes  **6** | Serological survey after 1978 Caribbean epidemic . DENV-2 isolated .  **6** | 2008 outbreak .  **9** | - | - | 88% |
| Saint Pierre and Miquelon | No | No | No  **-6** | - | HE high.  **-9** | - | - | -100% |
| Saint Vincent and the Grenadines | Yes | Yes | Yes  **6** | - | Between 1 and 200 cases reported every year, some confirmed, no case histories taken .  **9** | *Ae aegypti* present. Travel advisory.  **2** | **-3** As imported cases are not ruled out of the low annual case score, therefore most recent epidemic difficult to identify. | 47% |
| Samoa | Yes | No | Yes  **3** | DENV-2 isolated by PCR from 1971 outbreak . Traveller to Samoa introduced DENV-1 to Hawaii . DENV-3 confirmed 1996 .  **4.33** | Consistent case numbers with outbreaks every 5-10 years e.g. 1997 . Most recent outbreak 2001.  **6** | - | **+3** for presence evidence over long time scales in case data and literature. | 68% |
| San Marino | No | No | No  **-6** | - | HE high.  **-9** | - | - | -100% |
| Sao Tome and Principe | No | Yes | No  **-6** | - | HE low.  **3** | Travel advisory. No other supplemental evidence. | - | -20% |
| Saudi Arabia- Jizan | Yes | No | Yes  **3** | Case description from 2010 .  **4** | 2010 outbreak .  **9** | - | - | 67% |
| Saudi Arabia- Makkah | No | Yes | Yes  **3** | 49% suspected dengue cases serologically confirmed in 2005 . DENV-2 and 3 serologically identified in 2004 .  **5** | Outbreaks reported in 2004 , 2009 and 2010.  **9** | - | - | 71% |
| Saudi Arabia- Madinah | No | Yes | No  **-3** | - | HE high.  **-9** | - | - | -80% |
| Saudi Arabia- Baha | No | Yes | No  **-3** | - | HE high.  **-9** | - | - | -80% |
| Saudi Arabia-  Other states | No | No | No  **-6** | - | HE high.  **-9** | - | - | -100% |
| Senegal | Yes | Yes | Yes  **6** | DENV-2 isolated from 1990 outbreak . DENV-2 isolated from a variety of *Aedes* vectors . Isolation of DENV-2 and 4 and serological evidence that dengue is widespread . First report of DHF in West Africa .  **6.25** | Outbreaks reported 2009 .  **9** | - | - | 89% |
| Serbia | No | No | No  **-6** | - | HE medium.  **-3** | - | - | -60% |
| Seychelles | No | Yes | Yes  **3** | Identification of DENV-2 during 1976 outbreak .  **3** | Outbreak 1976-1979. Cases also reported in 1997 (706) and 2004 (405)  **9** | - | **-** | 63% |
| Sierra Leone | Yes | Yes | Yes  **6** | Blood donors found seropositive for dengue among many other arboviruses .  **3** | Sporadic cases have been reported+ HE low.  **6** | - | **-1** for likely cross reactivity of seroprevalence survey in the presence of many other arboviruses. | 58% |
| Singapore | Yes | Yes | Yes  **6** | 59% seropositive for dengue . Confirmed cases of DHF and dengue deaths . Review of the 2007 outbreak including PCR virus isolation .  **7.67** | 4 outbreaks in the last 10 years e.g. 2007 with rising case numbers and deaths .  **9** | - | - | 94% |
| Slovakia | No | No | No  **-6** | - | HE high.  **-9** | - | - | -100% |
| Slovenia | No | No | No  **-6** | - | HE high.  **-9** | - | - | -100% |
| Solomon Islands | Yes | Yes | Yes  **6** | 39% of sample population seropositive for dengue .  **4** | Low case numbers (less than 40 a year) detected since 1995 .  **3** | *Ae. albopictus* present . Travel advisory. Other arboviruses present .  **4** | **+2** for likely underreporting. | 63% |
| Somalia | Yes | Yes | Yes  **6** | DENV-1 and DENV-2 isolated from febrile soldiers . 39% seropositive in a refugee camp in Somalia . Dengue fever documented in an American soldier visiting Somalia .  **5** | 2011 outbreak (122 cases) reported among Ugandan soldiers serving in Somalia .  **9** | - | - | 83% |
| South Africa | No | No | No  **-6** | - | Sporadic imported cases with no autochthonous activity.  Only imported cases of dengue since 1980 . HE medium.  **-6** | - | - | -80% |
| South Georgia and the South Sandwich Islands | No | No | No  **-6** | - | HE high.  **-9** | - | - | -100% |
| South Sudan | No | Yes | Yes  **3** | DENV-2 in South Sudan, dengue present in both urban and sylvatic cycles .  **4** | HE low + sporadic cases.  **6** | - | - | 67% |
| Spain | No | No | No  **-6** | - | HE high.  **-9** | - | - | -100% |
| Sri Lanka | Yes | Yes | Yes  **6** | DENV-2 and 3 in Sri Lanka . DHF in Sri Lanka . 67% patients with acute febrile illness positive for dengue .  **7.33** | High case numbers and deaths yearly, outbreaks declared in 1986, 2009 , 2010 and 2011 .  **9** | - | - | 93% |
| Sudan | Yes | Yes | Yes  **6** | DENV-1 and 2 isolated using PCR from 1985-6 outbreak . DHF reported in Port Sudan . Serological evidence of past dengue infection .  **7** | Outbreaks reported in 2004 and 2005 and 2010 , sporadic cases reported.  **9** | - | - | 92% |
| Suriname | Yes | Yes | Yes  **6** | DHF in Suriname . DENV-4 isolated from Suriname .  **6** | Consistent case and DHF numbers since 1986 including elevated case numbers every 5-10 years (including 2011-12) .  **9** | - | - | 88% |
| Svalbard and Jan Mayen | No | No | No  **-6** | - | HE high.  **-9** | - | - | -100% |
| Swaziland | No | No | No  **-6** | - | HE low.  **3** | - | **-3** for surrounding South Africa not documenting cases despite active case detection. | -40% |
| Sweden | No | No | No  **-6** | - | HE high.  **-9** | - | - | -100% |
| Switzerland | No | No | No  **-6** | - | HE high.  **-9** | - | - | -100% |
| Syrian Arab Republic | No | No | No  **-6** | - | HE low.  **3** | Appearance of *Ae. albopictus* for the first time . Other arboviruses found (WNV) .  **2** | **-3** for lack of dengue evidence despite uncertainty surrounding Syria’s recent increase in dengue suitability and their ability to detect dengue cases. | -19% |
| Tajikistan | No | No | No  **-6** | - | HE low.  **3** | No supplementary evidence found. | - | -20% |
| Tanzania (United Republic of) | Yes | Yes | Yes  **6** | Returning travellers reporting dengue . 7.7% of residents on Zanzibar and 1.8% in Tosamaganga on the mainland seropositive for dengue . DENV-3 isolated in 2010 .  **7** | Sporadic cases reported in recent years +HE low.  **6** | **-** | - | 79% |
| Thailand | Yes | Yes | Yes  **6** | PCR isolation of dengue . 71% of school children seropositive . 44% of imported cases to Austria come from Thailand .  **8.33** | Predictable outbreaks every 2 to 4 years, well defined risk categories .  **9** | - | - | 97% |
| Timor-Leste | Yes | Yes | Yes  **6** | Molecular and clinical analysis of isolates from the 2005 outbreak . Returning soldiers contracted dengue in East Timor .  **7** | Outbreaks reported 2000 , 2005 (1,128 cases, high number of DHF and deaths) and 2012 .  **9** | - | - | 92% |
| Togo | Yes | No | No  **-3** | Dengue detected in French returning traveller .  **5** | HE low.  **3** | Seroprevalence test revealed presence of multiple arboviruses . Travel advisory. *Ae. aegypti* present .  **4** | - | 30% |
| Tokelau | Yes | No | Yes  **3** | - | Outbreaks in 1989 and 2001 .  **6** | - | - | 60% |
| Tonga | Yes | No | Yes  **3** | Identification of DENV-1 and 2 . Returning Taiwanese travellers diagnosed with dengue after Tonga visit .  **5** | Consistent case numbers with high case numbers in 1980, 1990 and 2004 . Many reports from 2007-8 outbreak including reports of DHF and deaths .  **9** | - | - | 71% |
| Trinidad and Tobago | Yes | Yes | Yes  **6** | DENV-4 in Trinidad and Tobago . 98% pregnant women seropositive for dengue and linking disease outbreaks to *Ae. aegypti* . Detailed case description of DHF .  **7.33** | Consistent case numbers including DHF yearly, outbreaks reported in 1998, 2009 and 2010 .  **9** | - | - | 93% |
| Tunisia | No | No | No  **-6** | - | HE medium.  **-3** | - | - | -60% |
| Turkey | No | No | No  **-6** | Dengue detected in 0.9% of blood donors in central Anatolia after excluding travel and Yellow fever vaccinations including IgM positive results . 12.6% of Aegean residents seropositive for dengue .  **4** | HE high.  **-9** | - | **+3** for uncertainty in seroprevalence surveys. Currently there is a lack of explanation of why there are IgG+ individuals in central Turkey. Until this is done there will be no consensus. | -33% |
| Turkmenistan | No | No | No  **-6** | - | HE low.  **3** | No supplementary evidence found. | - | -20% |
| Turks and Caicos Islands | No | Yes | Yes  **3** | One case detected in 2005 .  **4** | No outbreaks reported since 1978, all imported cases case investigated . 9 indigenous cases reported in 2011, but below threshold. HE high.  **-9** | Travel advisory. News reports.  **2** | **+3** for recent confirmed indigenous transmission and on-going vector control even if the scale is uncertain. | 10% |
| Tuvalu | Yes | No | Yes  **3** | Description of DHF cases .  **4** | Outbreak in 1992, sporadic smaller outbreaks every few years, no cases since 1998 .  **3** | *Ae. aegypti* present on the island . Travel advisory.  **2** | **-3** for lack of seroprevalence or PCR data to back up the older case data. | 30% |
| Uganda | No | No | No  **-6** | Dengue isolated from a returning European traveller 1999-2002 . DENV-2 isolated in 1993 .  **4** | HE low.  **3** | Travel advisory. Other arboviruses present . Ae. aegypti present .  **4** | **-** | 17% |
| Ukraine | No | No | No  **-6** | - | HE medium.  **-3** | - | - | -60% |
| United Arab Emirates | No | Yes | No  **-3** | - | HE high.  **-9** | - | - | -80% |
| United Kingdom | No | No | No  **-6** | - | HE high.  **-9** | - | - | -100% |
| United States Minor Outlying Islands | No | No | No  **-6** | - | HE high.  **-9** | - | - | -100% |
| Uruguay- Salto | No | No | No  **-6** | 1 confirmed case of indigenous dengue detected in 2007, the first non-imported dengue case.  **5** | HE high  **-9** | **-** | - | -42% |
| Uruguay- all other states | No | No | No  **-6** | - | Only a few cases since 1997 and all case investigated to rule out indigenous transmission + HE high.  **-9** | **-** | - | -100% |
| U.S.A. Texas-Cameron | Yes | No | Yes  **3** | DENV-1 serologically identified in 1980 . 4 cases in 1986 . DHF in 2005 . 25% seropositive following an spill over outbreak . 0.9% southern Texan residents seropositive in 1989 .  **5** | Although no officially declared epidemic the seroprevalence surveys suggest recent outbreaks.  **9** | - | - | 71% |
| U.S.A. Texas- Hidalgo | Yes | No | Yes  **3** | DENV-1 serologically identified . 0.9% southern Texan residents seropositive in 1989 .  **3** | HE high.  **-9** | News reports. No other supplementary evidence. | - | -13% |
| U.S.A. Texas- Webb | Yes | No | Yes  **3** | DENV-1 serologically identified . 2 cases serologically identified in 1986 .  **3** | HE high.  **-9** | News reports. No other supplementary evidence. | - | -13% |
| U.S.A. Texas- Maverick | Yes | No | Yes  **3** | DENV-1 serologically identified .  **3** | HE high.  **-9** | News reports. No other supplementary evidence. | - | -13% |
| U.S.A. Texas- Bee | Yes | No | Yes  **3** | DENV-1 serologically identified .  **3** | HE high.  **-9** | News reports. No other supplementary evidence. | - | -13% |
| U.S.A. Texas- Travis | Yes | No | Yes  **3** | DENV-1 serologically identified .  **3** | HE high.  **-9** | News reports. No other supplementary evidence. | - | -13% |
| U.S.A. Texas- Nueces | Yes | No | Yes  **3** | 3 cases serologically identified in 1986 .  **3** | HE high.  **-9** | News reports. No other supplementary evidence. | - | -13% |
| U.S.A. Florida- Monroe | Yes | Yes | Yes  **6** | 27 serologically confirmed dengue cases in Key West in 2009, 1 from a New York resident on holiday . 5.4% seroprevalence among Key West residents following the 2009 outbreak .  **7** | 2010 outbreak .  **9** | - | - | 92% |
| U.S.A. Florida- Miami-Dade | No | No | No  **-6** | 1 indigenous case serologically diagnosed in 2010 . 2 indigenous cases serologically diagnosed in 2011 .  **5** | No outbreaks, HE high.  **-9** | *Ae. aegypti* present . News reports.  **2** | **+ 3** for consecutive years transmission. | -17% |
| U.S.A. Florida- Broward County | No | No | No  **-6** | 1 indigenous case serologically diagnosed in 2010 .  **5** | No outbreaks, HE high.  **-9** | *Ae. aegypti* present . News reports.  **2** | - | -27% |
| U.S.A. Florida, Palm Beach County | No | No | No  **-6** | 2 indigenous cases serologically diagnosed in 2011 .  **5** | No outbreaks, HE high.  **-9** | - | - | -42% |
| U.S.A. Florida, Martin County | No | No | No  **-6** | 1 indigenous case serologically diagnosed in 2011 .  **5** | No outbreaks, HE high.  **-9** | - | - | -42% |
| U.S.A. Florida, Hillsborough County | No | No | No  **-6** | 1 indigenous case serologically diagnosed in 2011 .  **5** | No outbreaks, HE high.  **-9** | News reports of the 1 indigenous case. No other supplementary evidence. | - | -42% |
| U.S.A.-  Hawaii | Yes | No | Yes  **3** | Serological detection of dengue cases in 2002 . DENV-1 and DENV-2 introduced in 2000 . Dengue in returning German traveller in 1995 .  **5.66** | Low case numbers yearly with outbreaks every 3-6 years since 2001 .  **9** | - | - | 74% |
| United States of America- other states | No | No | No  **-6** | - | HE high.  **-9** | - | - | -100% |
| Uzbekistan | No | No | No  **-6** | - | HE low.  **3** | No supplementary evidence found. | - | -20% |
| Vanuatu | Yes | Yes | Yes  **6** | Serologically confirmed cases . Serologically confirmed case numbers .  **4.5** | Consistent case numbers yearly with elevated case numbers and outbreaks reported roughly every 5 years e.g. 1998 .  **9** | - | - | 81% |
| Venezuela | Yes | Yes | Yes  **6** | 41.9% of sample population seropositive for dengue . Descriptive study of DHF cases . Detection of DENV-1,3 and 4 in wild *Ae. aegypti* populations .  **7.33** | Outbreaks reported once or twice a decade e.g. 2009 with 15,000-100,000 cases including DHF and deaths, persistently high case numbers yearly .  **9** | - | - | 93% |
| Vietnam | Yes | Yes | Yes  **6** | 4.6% children seropositive for dengue . DENV-2 and DENV-4 coinfection . 18% imported cases into Austria from Vietnam .  **8.67** | Outbreaks almost yearly with 20,000 to 100,000 cases including high rates of DHF and deaths e.g. 2009 .  **9** | - | - | 99% |
| Virgin Islands, British | Yes | Yes | Yes  **6** | DENV-1 detected among community aid workers returning from the BVI . Outbreak reported among American tourists visiting BVI .  **5** | Sporadic outbreaks (up to 16 cases) reported every few years. Last outbreak 1996 .  **3** | - | - | 58% |
| Virgin Islands, U.S. | Yes | Yes | Yes  **6** | Serological detection of cases during 2005 outbreak . DHF case in a returning traveller to the U.S. Virgin Islands . Circulation of DENV-1,2 and 4 .  **6.33** | Case numbers reduced since 1993, but still outbreaks every few years including 2005, 2007 and 2010 . Program for prevention and control of dengue, no eradication .  **9** | - | - | 89% |
| Wallis and Futuna | Yes | No | Yes  **3** | DENV-2 isolated 1998 . DENV-4 isolated by PCR in 2009 .  **5** | Outbreaks reported once a decade (with confirmed cases) e.g. most recent outbreak:1998 with sporadic cases in intermediate years.  **6** | News reports. *Ae. aegypti* on Wallis and Futuna . Other arboviruses present . Travel advisory.  **6** | - | 67% |
| Western Sahara | No | No | No  **-6** | - | HE medium.  **-3** | - | - | -60% |
| Yemen | Yes | Yes | Yes  **6** | Dengue isolated from a returning traveller in 1984 . Dengue case imported into Italy (2010) . DENV-3 isolated from 2005 outbreak . Description of 2005 outbreak including detailed description of DHF .  **7** | Cases reported in 2003 and 2005. Official outbreaks confirmed 2008 , 2009, 2010 (1000-2000 cases) .  **9** | - | - | 92% |
| Zambia | No | No | No  **-6** | 1 seropositive returning aid worker .  **4** | HE low.  **3** | *Ae. aegypti* present . Travel advisory.  **2** | - | 10% |
| Zimbabwe | No | No | No  **-6** | 1 seropositive returning aid worker .  **4** | HE assumed low.  **3** | Outbreak of other arboviruses (chikungunya in 1963) . Travel advisory. *Ae. aegypti* present .  **4** | - | 17% |

**References**
